# Synthesis, In Silico and In Vitro Evaluation of Some Flavone Derivatives for Acetylcholinesterase and BACE-1 Inhibitory Activity

**DOI:** 10.3390/molecules25184064

**Published:** 2020-09-05

**Authors:** Thai-Son Tran, Thanh-Dao Tran, The-Huan Tran, Thanh-Tan Mai, Ngoc-Le Nguyen, Khac-Minh Thai, Minh-Tri Le

**Affiliations:** 1 Department of Medicinal Chemistry, Faculty of Pharmacy, University of Medicine and Pharmacy at Ho Chi Minh City, Ho Chi Minh City 700000, Vietnam; ttson@huemed-univ.edu.vn (T.-S.T.); tranthanhdao@uphcm.edu.vn (T.-D.T.); mthtan@ump.edu.vn (T.-T.M.); 2 Department of Pharmaceutical Chemistry, Faculty of Pharmacy, College of Medicine and Pharmacy, Hue University, Hue City 530000, Vietnam; tthuan@hueuni.edu.vn; 3 Department of Pharmaceutics and Industrial Pharmacy, Faculty of Pharmacy, South Can Tho University, Can Tho City 640000, Vietnam; dsngocle1984@gmail.com; 4 School of Medicine, Vietnam National University Ho Chi Minh City, Ho Chi Minh City 700000, Vietnam

**Keywords:** in silico, in vitro, QSAR, docking, flavone, acetylcholinesterase, β-secretase

## Abstract

Acetylcholinesterase (AChE) and β-secretase (BACE-1) have become attractive therapeutic targets for Alzheimer’s disease (AD). Flavones are flavonoid derivatives with various bioactive effects, including AChE and BACE-1 inhibition. In the present work, a series of 14 flavone derivatives was synthesized in relatively high yields (35–85%). Six of the synthetic flavones (**B4**, **B5**, **B6**, **B8**, **D6** and **D7**) had completely new structures. The AChE and BACE-1 inhibitory activities were tested, giving pIC**_50_** 3.47–4.59 (AChE) and 4.15–5.80 (BACE-1). Three compounds (**B3**, **D5** and **D6**) exhibited the highest biological effects on both AChE and BACE-1. A molecular docking investigation was conducted to explain the experimental results. These molecules could be employed for further studies to discover new structures with dual action on both AChE and BACE-1 that could serve as novel therapies for AD.

## 1. Introduction

Alzheimer’s disease (AD) is an irreversible neurodegenerative disorder with the defects in nervous system and cognitive function [1]. In this ailment, there is an impairment of the cerebral cortex with complex processes; involving the production of senile plaques, neurofibrillary tangles, synapse loss and neuroinflammation; leading to the progressive cognitive decline and memory loss [2,3]. At the present time, about 50 millions of people worldwide suffer from the disease, which creates a heavy burden on the health care systems of many countries [4,5,6]. Therefore, discovery of novel drugs for AD is an urgent task.

The exact pathogenesis of AD is currently not fully know and this disease might be referred to as a neurological disorder caused by multiple factors, such as: (i) low concentrations of acetylcholine (ACh) in synaptic clefts; (ii) the accumulation of intracellular neurofibrillary tangles with hyperphosphorylated Tau protein and extracellular amyloid plaques; (iii) oxidative stress; (iv) the homeostasis dysregulation of biometals [2,7,8,9]. The multifactorial nature of AD has transformed the paradigm of AD drug development from single to multitarget-directed ligands [10]. Acetylcholinesterase (AChE) and β-secretase (BACE-1) are the two drug targets that have attracted much attention among others. They are two critical enzymes in Alzheimer’s pathogenesis, responsible for the defects in the cholinergic signaling pathway [11] and the formation of β-amyloid plaques [12].

During the last few years, flavonoids, including flavones, have attracted much interest because of their diverse bioactivities [13,14,15,16,17,18,19,20,21,22,23,24]. Recent studies have indicated the abilities of flavones in inhibiting many enzymes [25,26,27,28,29], especially AChE [30] and BACE-1 [31,32]. Studies on the bioactivity of flavone derivatives against AChE and BACE-1 are thus promising for the discovery of novel therapeutic agents for AD.

In this study, a series of flavone derivatives was synthesized and evaluated for AChE and BACE-1 inhibitory activities with the aim of discovering new small-molecule inhibitors with dual action on both enzymes. The produced compounds could also be used in an external validation set to assess the two-dimensional quantitative structure–activity relationship (2D-QSAR) models developed for inhibitors of these enzymes. A molecular docking investigation was conducted to analyze the molecular binding abilities of these substances with the enzymes to obtain more detailed information on these influences on the bioactivity. 

## 2. Results

### 2.1. Chemistry

**Scheme 1:**
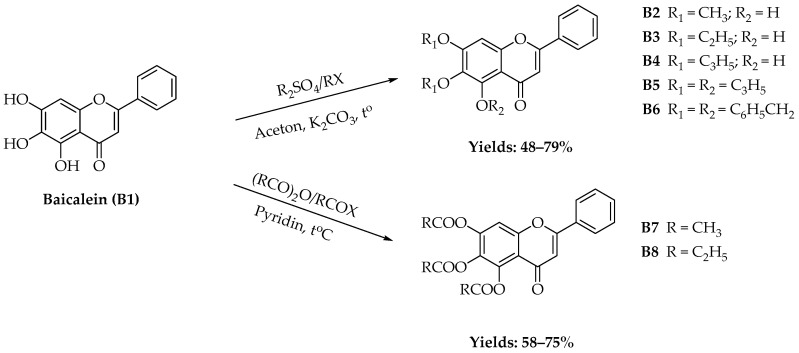
Synthesis of flavone derivatives from baicalein.

**Scheme 2:**
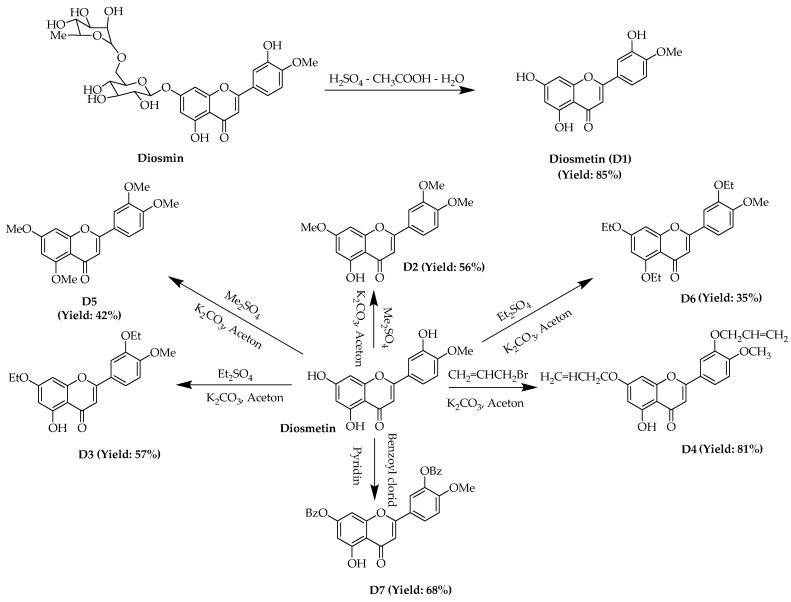
Synthesis of diosmetin and its derivatives.

Etherification (with acetone as solvent and K**_2_**CO**_3_** as catalyst) and esterification (in pyridine) reactions were conducted on baicalein (**B1**) to produce the derivatives **B2**–**B8** in relatively high yields (48–79%, Scheme 1). A hydrolysis process was carried out with diosmin to make diosmetin (**D1**, yield 85%), from which the other compounds **D2**–**D7** were synthesized via etherification and esterification reactions (Scheme 2) with yields of 35–81%. The structures of the synthetic substances were elucidated by ultraviolet (UV), infrared (IR), high-resolution mass spectrometry (HRMS), proton nuclear magnetic resonance (^1^H-NMR), and carbon-13 nuclear magnetic resonance (^13^C-NMR) spectroscopy as described in the Materials and Methods section and six of them (**B4**, **B5**, **B6**, **B8**, **D6** and **D7**) were identified as completely new compounds [33].

### 2.2. Enzyme Inhibition and 2D-QSAR Analysis

The results of the in vitro assays on AChE and BACE-1 inhibition are indicated in Table 1. From the biological evaluation, the inhibitory activities of synthesized substances against the two enzymes gave pIC**_50_** (−logIC**_50_**, negative common logarithm of the half maximal inhibitory concentration) values of 3.47–4.59 (IC**_50_** 340.09–25.51 µM) for AChE and 4.15–5.80 (IC**_50_** 70.79–1.58 µM) for BACE-1. Among the synthetic molecules, **B3**, **D5** and **D6** exhibited the highest bioactivities against both enzymes. Galantamine and quercetin were used as reference substances. Galantamine is a well-known AChE inhibitor used for the treatment of cognitive decline in mild to moderate AD. Quercetin is a flavonoid reported to have inhibitory activity against BACE-1. These two compounds have been used as references in many studies on AChE and BACE-1 inhibition. The results showed that the biological activities of the positive controls in this study are comparative to previously published values [34,35,36].

**Figure 1:**
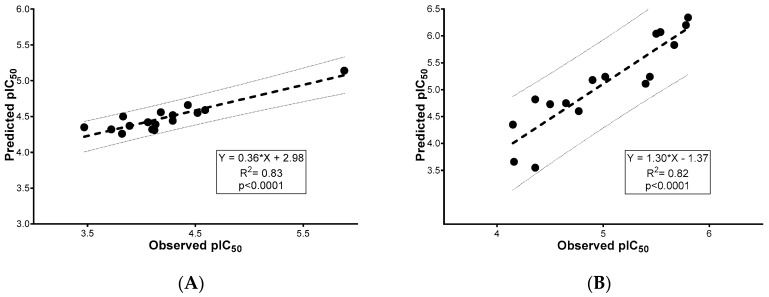
The linear regression between observed and predicted activity of synthesized flavone derivatives and positive control against (**A**) acetylcholinesterase (AChE) and (**B**) β-secretase (BACE-1).

In this study, the synthesized substances were used in an external validation set for the 2D-QSAR models published in a previous work [37] (Table 2). By using these 2D-QSAR models, the predicted values of pIC**_50_** for AChE and BACE-1 of the synthesized flavone derivatives were calculated and are indicated in Table 1. Correlations between observed and predicted activities of synthesized flavones molecules and positive controls againts AChE and BACE-1 were found (Figure 1) with good values of the squared correlation coefficient (R^2^) and root-mean-square error (RMSE) (R^2^ = 0.83 and RMSE = 0.44 for AChE; and R^2^ = 0.82 and RMSE = 0.40 for BACE-1). These results indicate that the reported models were statistically significant and sufficiently robust to predict the biological activities of new ligands. Selected molecular descriptors used for building 2D-QSAR models are described in detail in Table S1, and their values used in the prediction of pIC**_50_** of the synthesized flavone derivatives are provided in Tables S2 and S3.

### 2.3. Molecular Docking

**Figure 2:**
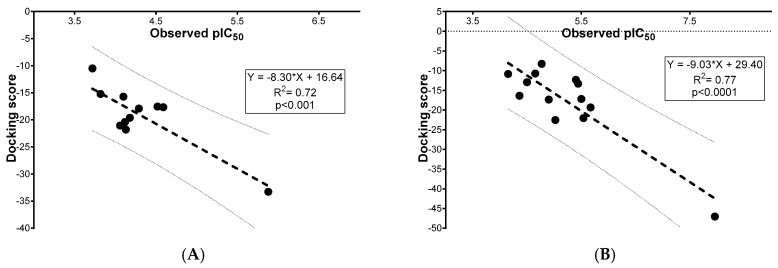
The linear regression between observed activity and docking score against (**A**) acetylcholinesterase (AChE) (values from **B2**, **B3, B5–8, D2, D4–6** and galantamine) and (**B**) β-secretase (BACE-1) (values from **B2–8, D2–4, D7**, umibecestat and quercetin).

**Figure 3:**
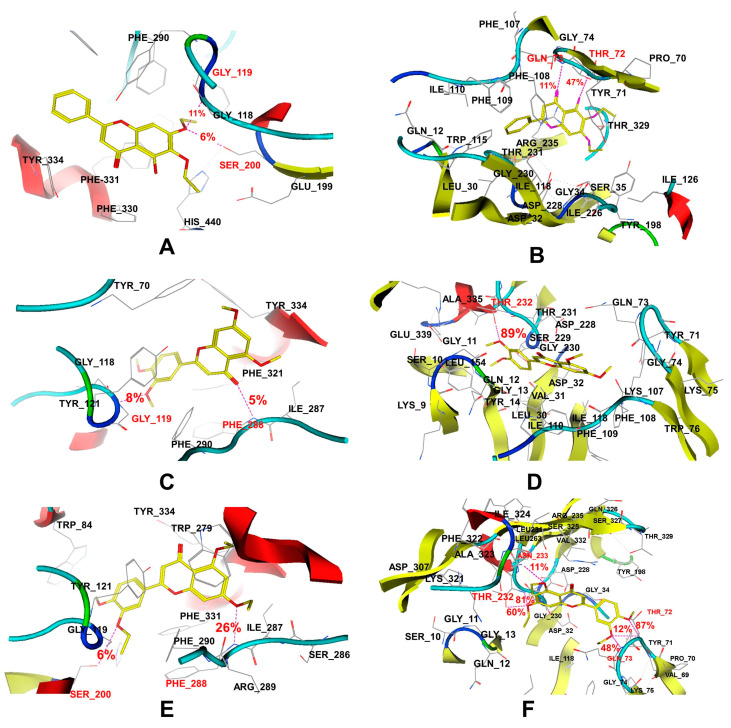
Interactions with AChE (left) and BACE-1 (right) of flavone derivatives **B3** (**A**,**B**), **D5** (**C**,**D**) and **D6** (**E**,**F**).

The results of the molecular docking study are reported in Table 1, Figure 2 and Figure 3, and in the Supporting Information (Tables S4–S6). The interactions of three compounds with the highest pIC**_50_** (**B3**, **D5** and **D6**) on both enzyme AChE and BACE-1 are indicated in Figure 3. In this study, the co-crystallized complexes employed for AChE was 1DX6 (resolution 2.30 Å) and for BACE-1 was 6EQM (resolution 1.35 Å). These were the complexes with high resolutions and the co-crystallized ligands were the drugs used in clinical (1DX6: galantamine) or in clinical development (6EQM: umibecestat). With this selection, the probability of docked compounds to be reached further optimization would be high [40]. The results indicated that all studied substances were successfully docked into the binding pockets of AChE and BACE-1 with the docking scores of (−10.36)–(−27.06) kJ·mol^−1^ (AChE) and (−8.29)–(−22.07) kJ·mol^−1^ (BACE-1). Linear correlations between docking scores and observed values of pIC**_50_** on AChE and BACE-1 of synthesized flavone derivatives were revealed (Figure 2). The ligand interactions between chemical molecules and their targets will be analysed more detailed in the Discussion section.

## 3. Discussion

In this study, a series of fourteen flavone molecules was synthesized in relatively high yields (35–85%). The structures of these substances were elucidated by UV, IR, HRMS, ^1^H-NMR and ^13^C-NMR spectroscopy, and six of them (**B4**, **B5**, **B6**, **B8**, **D6** and **D7**) were completely new. The bioactivities of these compounds were examined with pIC**_50_** 3.47–4.59 (AChE) and 4.15–5.80 (BACE-1). The synthetic molecules could be divided into two groups of derivatives, **B2**–**B8** (from baicalein, **B1**) and **D2**–**D7** (from diosmetin, **D1**). **B2**, **B3**, **D2**, **D3**, **D5** and **D6** had better AChE inhibiting effects than the respective parent compounds. Compared to baicalein and diosmetin, the synthesized derivatives of these two substances (except for **B4**) had the improved bioactivities against BACE-1. The results indicated that the derivatives of diosmetin had the higher inhibiting effects on BACE-1 than those of baicalein. **B3**, **D5** and **D6** exhibited the highest biological effects on both enzymes among the synthesized compounds.

A molecular docking study showed that all the synthesized flavone derivatives were successfully docked into the binding pocket of AChE (1DX6). Docking scores and pIC**_50_** values in 12 among studied substances (**B2**, **B3**, **B5–8**, **D2**, **D4–6** and galantamine) were quite correlated with R^2^ = 0.72 (Figure 2). In addition, the differences in the observed pIC**_50_** values could be partly explained by the analysis of the interactions between flavone molecules with AChE. These interactions include: (i) arene–arene interactions with Trp84, Trp279, Tyr334; (ii) arene–cation interaction with Trp84; (iii) hydrogen bonding with Asp72, Glu199. These are residues of the active site-gorge of the enzyme where Trp84 and Tyr334 are exposed on the surface, particularly Asp72 on the top, Glu199 on the bottom, and Trp279 is on the entrance of the active-site gorge [41]. Compared to galantamine, the flavone derivatives were all lower in bioactivity. This could be explained by the fact that galantamine could create an arene–cation interaction with Phe330, a strong hydrogen bond to Glu199 (score: 36%, length: 1.85 Å), and a hydrogen bond (score: 22%, length: 3.07 Å) to Ser200 (a residue in catalytic triad). **B1** and **B2** had the same mode of interaction with the enzyme but the docking score of **B1** was higher than that of **B2**. This could be due to the hydrogen bonds made by **B1** with Tyr130 (score: 68%, length: 2.61 Å) and Gly117 (score: 20%, length: 1.82 Å) were stronger than those of **B2** with these two residues (Tyr130: score 8%, length 3.14 Å; and Gly117: score 1%, length: 3.52 Å). In addition, the interaction between **B1** and Phe330 was an arene–arene interaction (proposed to consist of van der Waals, hydrophobic, and electrostatic forces) while this interaction seen with **B2** was a van der Waals force in general. However, the AChE inhibiting effect of **B2** was higher than that of **B1**. This could be due to **B1** lacked the van der Waals interactions with two important residues (seen in the **B2** case), including His440 of the catalytic triad and Asp72 of the active site-gorge. The docking results also indicated that **B3** could form only a weak but important hydrogen bond with Ser200 (score: 6%, length: 3.65 Å) and van der Waals interactions with His440. This could explain why **B3** had a stronger biological activity than **B1** on AChE, although the docking score of **B3** is lower. In the series of diosmetin derivatives, a hydrogen bond between the ligand molecule with either Gly119 or Ser200 could be needed for the improved activities of these substances than the parent compound (**D1**).

Molecular docking results on BACE-1 expressed that all the synthesized flavone derivatives were successfully docked into the binding pocket of this enzyme (6EQM). The interactions with catalytic dyad residues (Asp32 and Asp228) could be necessary for high inhibiting effect against BACE-1. The interaction models in Table S6 revealed that umibecestat could form strong hydrogen bonds with the catalytic dyad (Asp32: score 59%, length 1.82 Å; score 68%, length 1.75 Å, and Asp228: score 13%, length 2.48 Å; score 30%, length 1.96 Å) in addition to a hydrogen bond with Gly230 (score 21%, length 1.91 Å). This mode of interaction could explain the higher bioactivity against BACE-1 of umibecestat (observed pIC**_50_** 7.96). None of the studied flavone derivatives form any hydrogen bonds with the catalytic dyad residues. They interacted only with these amino acids through van der Waals forces. **B3, D2–D7** and quercetin (pIC**_50_** 5.02–5.80) were exposed to Asp32, Asp228, and Gly230 in the radius of 4.5–5.0 Å. Two substances with the lowest BACE-1 inhibitory activities, **B1** (pIC**_50_** 4.16) and **B4** (pIC_50_ 4.15), did not produce any interactions with Asp32, Asp228, and Gly230 (**B4**) or formed only a weak interaction with Gly230 at a distance >5.5 Å (**B1**). The remaining substances (**B2**, **B5–8** and **D1**) had pIC**_50_** values of 4.36–4.90. These molecules could form medium van der Waals interactions with Asp32, Asp228, and Gly230 (within a distance of 5.0–5.5 Å) or interacted only with one residue of the catalytic dyad. **D5** (pIC**_50_** 5.78) and **D6** (pIC**_50_** 5.80) formed strong hydrogen bonds with Thr232 (**D5**: score 89%, length 2.69 Å; **D6**: score 81%, length 2.49 Å; score 60%, length 2.49 Å). These interactions could explain for the higher activities against BACE-1 of those two compounds. In this study, a correlation between docking score and observed pIC_50_ for BACE-1 was found for most of the studied substances (**B2–8**, **D2–4**, **D7**, umibecestat and quercetin) with an R^2^ value of 0.77 (Figure 2). However, this correlation (as in the case of AChE above) was not a conclusion but an interesting result from the present work. In general, molecular docking (with a specified scoring function) is better in pose (spatial orientation of a ligand in the active site of an enzyme) than affinity (free binding energy) prediction. Free binding energy is related to the inhibition constant (K_i_). The more negative the free binding energy, the lower the K_i_ value. While K_i_ is a constant value for a given compound with an enzyme, an IC**_50_** (or pIC**_50_**) is a relative value, whose magnitude depends upon the concentration of substrate used in the assay. The relation between K_i_ and IC**_50_** could be obtained only if the mode of inhibition and the concentration of substrate used in the assay are reported [42,43,44].

## 4. Materials and Methods 

### 4.1. Materials and Instruments

All chemicals were obtained from commercial suppliers and used without further purification. Melting points were determined on open capillary tubes and are uncorrected (using a Stuart apparatus SMP20, Cole-Parmer, Staffordshire, UK). UV, IR, HRMS, and ^1^H-NMR and ^13^C-NMR spectra were recorded on V-730 UV-Vis (Jasco International Co. Ltd., Tokyo, Japan), IR Prestige-21 (Shimadzu, Kyoto, Japan), 6200 Series TOF and 6500 Series Q-TOF LC/MS (Agilent, Santa Clara, CA, USA) and AV500 (500 MHz, 125 MHz) (Bruker, Billerica, MA, USA) spectrometers, respectively. Chemical shifts were reported in parts per million (ppm) downfield relative to tetramethylsilane as an internal standard. Peak splitting patterns were abbreviated as m (multiplet), s (singlet), bs (broad singlet), d (doublet), bd (broad doublet), t (triplet) and dd (doublet of doublets), respectively. All computational processes were performed on a computer system equipped with an Intel^®^ Core^TM^ i&-7700 CPU @ 3.60 Hz processor, 16.0 GB of RAM, an NVIDIA GeForce GT 1030 2GB Visual Graphics Card and the 64 bit Windows 10 operating system (Microsoft, Redmond, WA, USA).

### 4.2. Chemistry

A series of 14 flavone derivatives were synthesized as indicated in Scheme 1 and Scheme 2, as follows:

#### 4.2.1. Synthesis of **B2**–**B6**

Baicalein (**B1**) was dissolved in anhydrous acetone. K**_2_**CO**_3_**, and a dialkyl sulfate or an alkyl bromide were then added. The reaction mixture was heated at 40–45 °C and monitored by thin layer chromatography eluting with the appropriate solvents. At the end of the reaction, the mixture was filtered to remove insoluble solids. The obtained solution was evaporated under reduced pressure and the crude product was purified by column chromatography with the appropriate solvent systems.

*5-Hydroxy-6,7-dimethoxyflavone* (**B2**). Yield: 65%. MP: 160–161 °C. UV (λ_max_ nm, MeOH): 210, 272, 314. IR (ν cm^−1^, KBr): 3453, 3064, 2941, 1662, 1616, 1456, 1128. MS: [M + H]^+^ *m*/*z* = 299.0917. ^1^H-NMR (DMSO-*d*_6_) δ (ppm): 12.76 (s, 1H, OH), 8.09 (dd, *J*_1_ = 1.5 Hz, *J*_2_ = 8 Hz, 2H, Ar-H), 7.63–7.57 (m, 3H, Ar-H), 7.02 (s, 1H, Ar-H), 6.97 (s, 1H, Ar-H), 3.94 (s, 3H, OCH**_3_**), 3.75 (s, 3H, OCH**_3_**). ^13^C-NMR (DMSO-*d*_6_) δ (ppm): 182.3, 163.4, 158.8, 152.7, 151.0, 132.1, 132.0, 130.6, 129.1, 126.3, 105.3, 104.9, 91.7, 60.0, 56.4.

*6,7-Diethoxy-5-hydroxyflavone* (**B3**). Yield: 50%. MP: 140–141 °C. UV (λ_max_ nm, MeOH): 210, 272, 315. IR (ν cm^−1^, KBr): 3447, 3071, 2982, 1661, 1614, 1454, 1125. MS: [M + H]^+^ *m*/*z* = 327.1230. ^1^H-NMR (DMSO-*d*_6_) δ (ppm): 12.72 (s, 1H, OH), 8.09 (dd, *J*_1_ = 1.5 Hz, *J*_2_ = 8 Hz, 2H, Ar-H), 7.64–7.56 (m, 3H, Ar-H), 7.00 (s, 1H, Ar-H), 6.94 (s, 1H, Ar-H), 4.19 (q, *J* = 7 Hz, 2H, CH_2_), 3.99 (q, *J* = 7 Hz, 2H, CH**_2_**), 1.40 (t, *J* = 7 Hz, 3H, CH**_3_**), 1.26 (t, *J* = 7 Hz, 3H, CH**_3_**). ^13^C-NMR (DMSO-*d*_6_) δ (ppm): 182.3, 163.3, 158.3, 152.6, 152.2, 132.0, 130.9, 130.6, 129.0, 126.3, 105.2, 104.9, 92.1, 67.8, 64.6, 15.3, 14.3.

*6,7-Diallyloxy-5-hydroxyflavone* (**B4**). Yield: 69%. MP: 132–133 °C. UV (λ_max_ nm, MeOH): 214, 273, 315. IR (ν cm^−1^, KBr): 3449, 3071, 2922, 1661, 1612, 1456, 1132. MS: [M + H]^+^ *m*/*z* = 351.1224. ^1^H-NMR (DMSO-*d*_6_) δ (ppm): 12.79 (s, 1H, OH), 8.09 (dd, *J*_1_ = 1,5 Hz, *J*_2_ = 8.5 Hz, 2H, Ar-H), 7.63–7.57 (m, 3H, Ar-H), 7.03 (s, 1H, Ar-H), 6.98 (s, 1H, Ar-H), 6.13–6.01 (m, 2H, -CH=), 5.48 (dd, *J*_1_ = 1.5 Hz, *J*_2_ = 17.5 Hz, 1H, =CH_trans_), 5.33 (dd, *J*_1_ = 1.5 Hz, *J*_2_ = 17 Hz, 1H, =CH_trans_), 5.33 (dd, *J*_1_ = 1.5 Hz, *J*_2_ = 10.5 Hz, 1H, =CH_cis_), 5.18 (dd, *J*_1_ = 1.5 Hz, *J*_2_ = 10.5 Hz, 1H, =CH_cis_), 4.75 (d, *J* = 5 Hz, 2H, CH**_2_**), 4.51 (d, *J* = 5.5 Hz, 2H, CH**_2_**). ^13^C-NMR (DMSO-*d*_6_) δ (ppm): 182.3, 163.4, 157.8, 152.6, 152.3, 134.4, 132.7, 132.0, 130.6, 130.5, 129.1, 126.3, 117.8, 117.5, 105.3, 104.9, 92.5, 73.0, 69.2.

*5,6,7-Triallyloxyflavone* (**B5**). Yield: 48%. MP: 117–118 °C. UV (λ_max_ nm, MeOH): 215, 264, 307. IR (ν cm^−1^, KBr): 3053, 2978, 1638, 1603, 1453, 1117. MS: [M + H]^+^ *m*/*z* = 391.1549. ^1^H-NMR (DMSO-*d*_6_) δ (ppm): 8.05 (dd, *J*_1_ = 2 Hz, *J*_2_ = 8 Hz, 2H, Ar-H), 7.59–7.55 (m, 3H, Ar-H), 7.23 (s, 1H, Ar-H), 6.79 (s, 1H, Ar-H), 6.17–6.02 (m, 3H, -CH=), 5.49 (dd, *J*_1_ = 1.5 Hz, *J*_2_ = 17 Hz, 1H, =CH_trans_), 5.37 (dd, *J*_1_ = 1.5 Hz, *J*_2_ = 17 Hz, 1H, =CH_trans_), 5.36 (dd, *J*_1_ = 1.5 Hz, *J*_2_ = 17 Hz, 1H, =CH_trans_), 5.34 (dd, *J*_1_ = 1.5 Hz, *J*_2_ = 10.5 Hz, 1H, =CH_cis_), 5.20 (dd, *J*_1_ = 1.5 Hz, *J*_2_ = 10.5 Hz, 2H, =CH_cis_), 4.76 (d, *J* = 5 Hz, 2H, CH**_2_**), 4.54 (d, *J* = 5.5 Hz, 2H, CH**_2_**), 4.50 (d, *J* = 5.5 Hz, 2H, CH**_2_**). ^13^C-NMR (DMSO-*d*_6_) δ (ppm): 175.7, 160.1, 156.4, 153.8, 150.4, 138.8, 134.6, 134.2, 132.6, 131.4, 130.9, 129.0, 125.9, 117.8, 117.4, 117.2, 112.4, 107.5, 98.2, 75.0, 73.8, 69.2.

*5,6,7-Tribenzyloxyflavone* (**B6**). Yield: 79%. MP: 126–127 °C. UV (λ_max_ nm, MeOH): 264, 308. IR (ν cm^−1^, KBr): 3032, 2940, 1643, 1601, 1450, 1117. MS: [M + H]^+^ *m*/*z* = 541.2011. ^1^H-NMR (DMSO-*d*_6_) δ (ppm): 8.08 (dd, *J*_1_ = 2 Hz, *J*_2_ = 8 Hz, 2H, Ar-H), 7.61–7.45 (m, 8H, Ar-H), 7.43 (s, 1H, Ar-H), 7.41–7.26 (m, 10H, Ar-H), 6.85 (s, 1H, Ar-H), 5.33 (s, 2H, CH_2_), 5.03 (s, 2H, CH**_2_**), 4.97 (s, 2H, CH**_2_**). ^13^C-NMR (DMSO-*d*_6_) δ (ppm): 175.8, 160.2, 156.7, 154.0, 150.6, 139.2, 137.3, 137.1, 135.9, 131.5, 130.9, 129.0, 128.5, 128.3, 128.2, 128.0, 128.0, 127.9, 127.9, 127.8, 126.0, 112.6, 107.6, 98.6, 75.6, 74.9, 70.6.

#### 4.2.2. Synthesis of **B7** and **B8**

Baicalein (**B1**) was dissolved in pyridine and an anhydride acid or an acid chloride was then added. The reaction mixture was heated at 70–80 °C and monitored by thin layer chromatography with an appropriate solvent system. When the reaction was complete, the mixture was poured into cold water and stirred vigorously. The precipitate was then filtered and recrystallized from an appropriate solvent to yield final product.

*5,6,7-Triacetoxyflavone* (**B7**). Yield: 58%. MP: 196–197 °C. UV (λ_max_ nm, MeOH): 256, 294. IR (ν cm^−1^, KBr): 3065, 2936, 1784, 1645, 1452, 1175. MS: [M + H]^+^ *m*/*z* = 397.0916. ^1^H-NMR (DMSO-*d*_6_) δ 8.08 (dd, *J*_1_ = 1.5 Hz, *J*_2_ = 8.5 Hz, 2H, Ar-H), 7.83 (s, 1H, Ar-H), 7.62–7.57 (m, 3H, Ar-H), 6.94 (s, 1H, Ar-H), 2.37 (s, 3H, CH**_3_**), 2.37 (s, 3H, CH**_3_**), 2.3 (s, 3H, CH**_3_**). ^13^C-NMR (DMSO-*d*_6_) δ 175.3, 167.8, 167.5, 167.4, 162.0, 153.6, 146.7, 141.2, 132.6, 132.0, 130.4, 129.1, 126.4, 115.1, 111.1, 107.5, 20.4, 20.3, 19.7.

*5,6,7-Tripropionoxyflavone* (**B8**). Yield: 75%. MP: 167–168 °C. UV (λ_max_ nm, MeOH): 256, 295. IR (ν cm^−1^, KBr): 3063, 2984, 2943, 1782, 1643, 1454, 1111. MS: [M + H]^+^ *m*/*z* = 439.1384. ^1^H-NMR (DMSO-*d*_6_) δ 8.08 (dd, *J*_1_ = 1.5 Hz, *J*_2_ = 8.5 Hz, 2H, Ar-H), 7.83 (s, 1H, Ar-H), 7.62–7.57 (m, 3H, Ar-H), 6.93 (s, 1H, Ar-H), 2.68 (q, *J* = 7.5 Hz, 2H, CH**_2_**), 2.67 (q, *J* = 7.5 Hz, 2H, CH**_2_**), 2.66 (q, *J* = 7.5 Hz, 2H, CH**_2_**), 1.18 (t, *J* = 7.5 Hz, 3H, CH**_3_**), 1.16 (t, *J* = 7.5 Hz, 3H, CH**_3_**), 1.16 (t, *J* = 7.5 Hz, 3H, CH**_3_**). ^13^C-NMR (DMSO-*d*_6_) δ 175.3, 171.0, 170.8, 170.7, 162.0, 153.6, 146.8, 141.2, 132.5, 132.0, 130.4, 129.1, 126.3, 115.1, 111.0, 107.5, 26.7, 26.6, 26.2, 9.0, 8.7, 8.6.

#### 4.2.3. Synthesis of Diosmetin (**D1**)

Diosmin was placed in a two-necked flask with a solution of sulfuric acid-acetic acid-water. The reaction mixture was heated at 95–105 °C and monitored by thin layer chromatography with an appropriate solvent system. At the end of the reaction, the mixture was poured into cold water and stirred vigorously. The precipitate formed was then filtered and recrystallized from an appropriate solvent to yield the final product.

*5*,*7*,*3’-Trihydroxy-4’-methoxyflavone* (**D1**). Yield: 85%. MP: 277–278 °C. UV (λ_max_ nm, MeOH): 252, 269, 345. IR (ν cm^−^^1^, KBr): 3387, 3089, 2940, 2844, 1652, 1610, 1507, 1166. MS: [M + H]^+^ *m*/*z* = 301.0709. ^1^H-NMR (DMSO-*d*_6_) δ 12.92 (s, 1H, OH), 10.82 (s, 1H, OH), 9.43 (s, 1H, OH), 7.52 (dd, *J*_1_ = 8.5 Hz, *J*_2_ = 2.5 Hz, 1H, Ar-H), 7.42 (d, *J* = 2.5 Hz, 1H, Ar-H), 7.07 (d, *J* = 8.5 Hz, 1H, Ar-H), 6.73 (s, 1H, Ar-H), 6.46 (d, *J* = 2 Hz, 1H, Ar-H), 6.19 (d, *J* = 2 Hz, 1H, Ar-H), 3.86 (s, 3H, OCH**_3_**). ^13^C-NMR (DMSO-*d*_6_) δ 181.6, 164.1, 163.5, 161.4, 157.3, 151.1, 146.7, 123.0, 118.6, 112.9, 112.1, 103.7, 103.5, 98.8, 93.8, 55.7.

#### 4.2.4. Synthesis of **D2**–**D6**

Derivatives **D2**–**D6** were synthesized from diosmetin (**D1**) through the same reaction processes as described in the synthesis of substances B2–B6 from baicalein (**B1**).

*5-Hydroxy-3’,4’,7-trimethoxyflavone* (**D2**). Yield: 56%. MP: 200–201 °C. UV (λ_max_ nm, MeOH): 242, 269, 340. IR (ν cm^−^^1^, KBr): 3444, 3001, 2951, 2835, 1662, 1589, 1502, 1168. MS: [M + H]^+^ *m*/*z* = 329.1015. ^1^H-NMR (DMSO-*d*_6_) δ 12.92 (s, 1H, OH), 7.72 (dd, *J*_1_ = 8.5 Hz, *J*_2_ = 2 Hz, 1H, Ar-H), 7.60 (d, *J* = 2 Hz, 1H, Ar-H), 7.14 (d, *J* = 8.5 Hz, 1H, Ar-H), 7.00 (s, 1H, Ar-H), 6.82 (d, *J* = 2 Hz, 1H, Ar-H), 6.45 (d, *J* = 2 Hz, 1H, Ar-H), 3.89 (s, 3H, OCH**_3_**), 3.88 (s, 3H, OCH**_3_**), 3.86 (s, 3H, OCH**_3_**). ^13^C-NMR (DMSO-*d*_6_) δ 182.0, 165.2, 163.6, 161.1, 157.3, 152.2, 149.0, 122.7, 120.1, 111.7, 109.5, 104.7, 104.0, 98.0, 92.7, 56.0, 55.9, 55.7.

*3’,7-Diethoxy-5-hydroxy-4’-methoxyflavone* (**D3**). Yield: 57%. MP: 173–174 °C. UV (λ_max_ nm, MeOH): 269, 338. IR (ν cm^−^^1^, KBr): 3452, 3057, 2920, 2835, 1654, 1622, 1510, 1168. MS: [M + H]^+^ *m*/*z* = 357.1327. ^1^H-NMR (DMSO-*d*_6_) δ 12.90 (s, 1H, OH), 7.70 (dd, *J*_1_ = 8.5 Hz, *J*_2_ = 2.5 Hz, 1H, Ar-H), 7.58 (d, *J* = 2.5 Hz, 1H, Ar-H), 7.13 (d, *J* = 8.5 Hz, 1H, Ar-H), 7.00 (s, 1H, Ar-H), 6.69 (d, *J* = 2.5 Hz, 1H, Ar-H), 6.45 (d, *J* = 2.5 Hz, 1H, Ar-H), 4.16 (q, *J* = 7 Hz, 2H, CH**_2_**), 4.15 (q, *J* = 7 Hz, 2H, CH**_2_**), 3.86 (s, 3H, OCH**_3_**), 1.37 (t, *J* = 7 Hz, 3H, CH**_3_**), 1.36 (t, *J* = 7 Hz, 3H, CH**_3_**). ^13^C-NMR (DMSO-*d*_6_) δ 181.9, 164.4, 163.6, 161.1, 157.3, 152.4, 148.2, 122.7, 120.1, 111.8, 110.5, 104.6, 103.9, 98.3, 93.1, 64.2, 64.1, 55.7, 14.6, 14.3.

*3’,7-Diallyloxy-5-hydroxy-4’-methoxyflavone* (**D4**). Yield: 81%. MP: 126–127 °C. UV (λ_max_ nm, MeOH): 243, 269, 339. IR (ν cm^−^^1^, KBr): 3446, 3082, 2931, 2841, 1662, 1608, 1498, 1168. MS: [M + H]^+^ *m*/*z* = 381.1333. ^1^H-NMR (DMSO-*d*_6_) δ 12.90 (s, 1H, OH), 7.71 (dd, *J*_1_ = 8.5 Hz, *J*_2_ = 2 Hz, 1H, Ar-H), 7.61 (d, *J* = 2 Hz, 1H, Ar-H), 7.15 (d, *J* = 8.5 Hz, 1H, Ar-H), 6.99 (s, 1H, Ar-H), 6.82 (d, *J* = 2 Hz, 1H, Ar-H), 6.40 (d, *J* = 2 Hz, 1H, Ar-H), 6.11–6.04 (m, 2H, -CH=), 5.45 (dd, *J*_1_ = 17 Hz, *J*_2_ = 1.5 Hz, 1H, =CH_trans_), 5.44 (dd, *J*_1_ = 17 Hz, *J*_2_ = 1.5 Hz, 1H, =CH_trans_), 5.31 (dd, *J*_1_ = 10 Hz, *J*_2_ = 1.5 Hz, 1H, =CH_cis_), 5.29 (dd, *J*_1_ = 10 Hz, *J*_2_ = 1.5 Hz, 1H, =CH_cis_), 4.71–4.69 (m, 4H, CH_2_), 3.87 (s, 3H, OCH_3_). ^13^C-NMR (DMSO-*d*_6_) δ 181.9, 164.0, 163.5, 161.2, 157.2, 152.5, 147.8, 133.6, 132.8, 122.7, 120.4, 118.1, 117.9, 112.0, 111.1, 104.8, 104.0, 98.4, 93.4, 69.2, 68.9, 55.8.

*3’,4’,5,7-Tetramethoxyflavone* (**D5**). Yield: 42%. MP: 196–197 °C. UV (λ_max_ nm, MeOH): 242, 265, 331. IR (ν cm^−^^1^, KBr): 3001, 2938, 2841, 1605, 1514, 1018. MS: [M + H]^+^ *m*/*z* = 343.1179. ^1^H-NMR (DMSO-*d*_6_) δ 7.61 (dd, *J*_1_ = 8.5 Hz, *J*_2_ = 2 Hz, 1H, Ar-H), 7.51 (d, *J* = 2 Hz, 1H, Ar-H), 7.08 (d, *J* = 8.5 Hz, 1H, Ar-H), 6.83 (d, *J* = 2 Hz, 1H, Ar-H), 6.74 (s, 1H, Ar-H), 6.48 (d, *J* = 2 Hz, 1H, Ar-H), 3.90 (s, 3H, OCH**_3_**), 3.88 (s, 3H, OCH**_3_**), 3.84 (s, 3H, OCH_3_), 3.82 (s, 3H, OCH**_3_**). ^13^C-NMR (DMSO-*d*_6_) δ 175.6, 163.5, 160.1, 159.5, 159.0, 151.5, 148.9, 123.0, 119.2, 111.5, 109.0, 108.2, 106.9, 96.0, 93.3, 55.9, 55.8, 55.7, 55.6.

*3′,5,7-Triethoxy-4′-methoxyflavone* (**D6**). Yield: 35%. MP: 167–168 °C. UV (λ_max_ nm, MeOH): 242, 266, 234. IR (ν cm^−^^1^, KBr): 3082, 2977, 2929, 1601, 1514, 1115. MS: [M + H]^+^ *m*/*z* = 385.1645. ^1^H-NMR (DMSO-*d*_6_) δ 7.62 (dd, *J*_1_ = 8.5 Hz, *J*_2_ = 2 Hz, 1H, Ar-H), 7.51 (d, *J* = 2 Hz, 1H, Ar-H), 7.10 (d, *J* = 8.5 Hz, 1H, Ar-H), 6.82 (d, *J* = 2 Hz, 1H, Ar-H), 6.69 (s, 1H, Ar-H), 6.45 (d, *J* = 2 Hz, 1H, Ar-H), 4.18 (q, *J* = 7 Hz, 2H, CH**_2_**), 4.14 (q, *J* = 7 Hz, 2H, CH**_2_**), 4.08 (q, *J* = 7 Hz, 2H, CH**_2_**), 3.85 (s, 3H, OCH_3_), 1.37 (t, *J* = 7 Hz, 9H, CH**_3_**). ^13^C-NMR (DMSO-*d*_6_) δ 175.5, 162.6, 159.4, 159.3, 159.0, 151.6, 148.1, 123.1, 119.1, 111.6, 109.9, 108.3, 106.9, 97.1, 93.6, 64.2, 64.0, 63.9, 55.5, 14.6, 14.3, 14.3. 

#### 4.2.5. Synthesis of **D7**

Diosmetin (**D1**) was dissolved in pyridine and benzoyl chloride was then added. The reaction mixture was heated at 40–45 °C and monitored by thin layer chromatography with the appropriate solvent system. At the end of the reaction, the mixture was poured into cold water and stirred vigorously. The precipitate formed was then filtered off and recrystallized in the appropriate solvent to yield the final product.

*3’,7-Dibenzoyloxy-5-hydroxy-4’-methoxyflavone* (**D7**). Yield: 68%. MP: 227–228 °C. UV (λ_max_ nm, MeOH): 232, 270, 324. IR (ν cm^–1^, KBr): 3066, 1742, 1608, 1503, 1250, 1054. MS: [M + H]^+^ *m*/*z* = 509.1226. ^1^H-NMR (DMSO-*d*_6_) δ 12.93 (s, 1H, OH), 8.16–8.11 (m, 6H, Ar-H), 7.79–7.76 (m, 2H, Ar-H), 7.64–7.61 (m, 4H, Ar-H), 7.39 (d, *J* = 9 Hz, 1H, Ar-H), 7.29 (d, *J* = 2 Hz, 1H, Ar-H), 7.14 (s, 1H, Ar-H), 6.84 (d, *J* = 2 Hz, 1H, Ar-H), 3.89 (s, 3H, OCH**_3_**). ^13^C-NMR (DMSO-*d*_6_) δ 182.4, 163.8, 163.7, 163.2, 160.7, 156.1, 155.8, 154.3, 139.6, 134.3, 134.2, 129.9, 129.8, 129.0, 128.9, 128.4, 128.3, 126.2, 122.7, 121.5, 113.3, 108.2, 105.3, 104.6, 101.7, 56.3.

All spectra of the synthesized substances are provided in the Supporting Information (Table S7).

### 4.3. In Vitro Assays

AChE inhibitory activities of the synthesized flavone derivatives were determined by Ellman’s method [45]. Purified acetylcolinesterase from *Electrophorus electricus* (electric eel), acetylthiocholine iodide (ATCI, substrate), and galantamine (positive control) were purchased from Sigma–Aldrich (St. Louis, MO, USA) and used in this study. The assay was performed as described earlier [46] in 96-well microtiter plates using the EMR-500 ELISA system (Labomed, Los Angeles, CA, USA). Each well of the plate contained a mixture of sodium phosphate buffer pH 8, sample (studied compounds or reference), acetylcholinesterase, ATCI, and 5,5′-dithio-bisnitrobenzoic acid (DTNB). The absorbance was recorded at 405 nm. All samples were assayed in triplicate.

A fluorescence β-Secretase (BACE-1) Activity Detection Kit was purchased from Sigma–Aldrich and used to determine the effect of the synthesized flavone derivatives on β-secretase activity. The assay was carried out according to the manufacturer’s protocol as described earlier [47,48]. The enzyme solution was reacted with the substrate (7-methoxycumarin-4-acetyl-(Asn670, Lue671)-amyloid β/A4 precursor protein 770 fragment 667–676-(2,4-dinitrophenyl))Lys-Arg-Arg amide trifluoroacetate salt and sulfated polysaccharide samples in a fluorescence assay buffer in different wells. Baseline readings were measure immediately on an F-7000 fluorescence spectrophotometer (Hitachi, Tokyo, Japan; excitation: 320 nm; emission: 405 nm) and repeated after 2 h incubation at 37 °C. Quercetin was used as the positive control.

### 4.4. Molecular Docking

Ligand molecules were prepared directly in Sybyl X 2.0 [49]. Complexes 1DX6 of AChE (acetylcolinesterase from *Electrophorus electricus* in complex with (−)-galantamine, resolution 2.3 Å) and 6EQM of BACE-1 (human BACE-1 in complex with umibecestat, resolution 1.35 Å) were downloaded from Protein Data Bank [50]. Docking protocols were firstly validated by the method of pose selection [51] on three conformations of co-crystallized ligands. The root-mean-square deviation (RMSD) value between re-docked conformations and the original bound ligand in the co-crystallized complex which was ≤1.5 Å would indicate the reliability of the binding ability prediction of new ligands. In this study, FlexX program in BioSolveIT LeadIt [52] was used with default settings. This program applied a flexible-based docking methodology, an incremental construction algorithm for the seach of ligand conformations, and empirical scoring functions to score and rank the docking poses [53,54]. The interactions between flavone molecules and their targets, including hydrogen bonds, π–π, cation–π, ionic and van der Waals, were rendered and analyzed in the MOE 2008.10 program [55].

## 5. Conclusions

In this study, 14 flavone derivatives divided into two groups were synthesized and tested for AChE and BACE-1 inhibition, giving pIC**_50_** values of 3.47–4.59 (AChE) and 4.15–5.80 (BACE-1). The synthesized substances were also used in an external validation set to evaluate the developed 2D-QSAR model for AChE and BACE-1 inhibitors with relatively high correlations between the observed and estimated bioactivities (R^2^ = 0.83 and RMSE = 0.44 for AChE, and R^2^ = 0.82 and RMSE = 0.40 for BACE-1). Six of the synthesized derivatives (**B4**, **B5**, **B6**, **B8**, **D6** and **D7**) were new. The molecular docking model developed in the present work could be used to explain the biological activity observed in the in vitro assays. Among the studied flavone derivatives, **B3**, **D5** and **D6** were the three derivatives with the strongest inhibitory activities against both AChE and BACE-1. These substances could be used in the further studies.

## Figures and Tables

**Table 1:** Acetylcholinesterase (AChE) and β-secretase (BACE-1) inhibitory activities and docking scores of the synthesized flavone derivatives.

Compound	AChE				BACE-1			
	IC_50_ (µM)	Obs. pIC_50_	Pre. pIC_50_ *	Docking Score (kJ·mol^−1^) **	IC_50_ (µM)	Obs. pIC_50_	Pre. pIC_50_ *	Docking Score (kJ·mol^−1^) **
B1 (Baicalein)	37.15	4.43	4.66	−27.06	69.18	4.16	3.66	−17.64
B2	25.51	4.59	4.59	−17.67	43.65	4.36	4.82	−16.37
**B3**	**30.01**	**4.52**	**4.55**	**−17.55**	**3.98**	**5.40**	**5.11**	**−** **12.35**
B4	51.81	4.29	4.44	−10.36	70.79	4.15	4.35	−10.84
B5	191.47	3.72	4.32	−10.49	16.98	4.77	4.60	−8.29
B6	80.32	4.10	4.32	−15.71	12.59	4.90	5.18	−17.35
B7	51.29	4.29	4.52	−17.91	31.62	4.50	4.73	−12.95
B8	66.24	4.18	4.56	−19.61	22.39	4.65	4.75	−10.75
D1 (Diosmetin)	147.91	3.83	4.50	−22.86	43.65	4.36	3.55	−19.32
D2	87.10	4.06	4.42	−21.05	2.14	5.67	5.83	−19.33
D3	128.82	3.89	4.37	−20.90	3.16	5.50	6.04	−17.18
D4	151.36	3.82	4.26	−15.22	3.60	5.44	5.24	–13.31
**D5**	**73.82**	**4.13**	**4.39**	**−21.80**	**1.66**	**5.78**	**6.20**	**−** **13.46**
**D6**	**75.91**	**4.12**	**4.31**	**−20.39**	**1.58**	**5.80**	**6.34**	**−** **12.02**
D7	340.09	3.47	4.35	−25.35	2.86	5.54	6.07	−22.07
Galanthamine	1.31	5.88	5.14	−33.28	-	-	-	-
Umibecestat	-	-	-	-	0.01	7.96 [38]	7.67	−47.04
Quercetin	-	-	-	-	9.55	5.02	5.24	−22.55
		R^2^ = 0.83, RMSE = 0.44				R^2^ = 0.82, RMSE = 0.40		

Obs.: Observed, Pre.: Predicted, R^2^ (squared correlation coefficient), RMSE (root-mean-square error), IC**_50_**: the half maximal inhibitory concentration, pIC**_50_**: -logIC**_50_**. * pIC**_50_** values predicted using 2D-QSAR models in the previous published work [37]. ** Molecular docking results were reported using the co-crystallized 1DX6 and 6EQM for AChE and BACE-1, respectively. **B3**, **D5** and **D6** (highlighted in bold) exhibited the highest bioactivities against both enzymes.

**Table 2:** Two-dimensional quantitative structure–activity relationship models (2D-QSAR) for AChE and BACE-1 inhibitors reported in the previous published work [37].

AChE *												
pIC**_50_** = −0.928 + (2.348 × BCUT_SLOGP_3) − (0.150 × *reactive*) − (0.002 × SlogP_VSA2) − (0.004 × PEOE_VSA+1) − (0.004 × SMR_VSA2) − (0.005 × PEOE_VSA–3)												
***Internal Validation***					***External Validation***							
N	RMSE	R^2^	RMSE_LOO_	Q^2^_LOO_	N	RMSE	R^2^	R^2^_(PRED)_	$r_{m}^{2}$	$\bar{r_{m}^{2}}$	$\Delta r_{m}^{2}$	CCC
50	0.18	0.70	0.22	0.57	22	0.16	0.78	0.78	0.65	0.69	0.11	0.88
BACE-1 *												
pIC**_50_** = 1.268 + (6.370 × BCUT_PEOE_1) + (3.305 × *a_ICM*) + (0.870 × *petitjean*) + (0.157 × *a_Nn*) + (0.085 × *rings*) + (0.022 × PEOE_VSA–6) + (0.006 × PEOE_VSA–0) + (0.009 × SlogP_VSA3) + (0.009 × SlogP_VSA5) – (0.478 × *chiral_u*) − (0.260 × *logS*)												
***Internal Validation***					***External Validation***							
N	RMSE	R^2^	RMSE_LOO_	Q^2^_LOO_	N	RMSE	R^2^	R^2^_(PRED)_	$r_{m}^{2}$	$\bar{r_{m}^{2}}$	$\Delta r_{m}^{2}$	CCC
150	0.37	0.80	0.40	0.77	65	0.41	0.83	0.81	0.79	0.76	0.05	0.91

N: number of compounds, RMSE_LOO_ (cross-validated root-mean-square error), Q^2^_LOO_ (cross-validated squared correlation coefficient), R^2^_(PRED)_ (predicted R-squared), CCC (concordance correlation coefficient), and $r_{m}^{2},\;\bar{r_{m}^{2}},\;\Delta r_{m\;}^{2}$(validation metrics suggested by Roy et al. [39]). * Selected molecular descriptors used for building 2D-QSAR models are described in detail in Table S1.

## Supplementary Materials

The following are available online at https://www.mdpi.com/1420-3049/25/18/4064/s1, Table S1: Selected descriptors used for building 2D-QSAR models reported in the previous published work, Table S2: Values of selected descriptors used in the prediction of pIC**_50_** of the synthesized flavone derivatives (AChE), Table S3: Values of selected descriptors used in the prediction of pIC**_50_** of the synthesized flavone derivatives (BACE-1), Table S4: Results of re-docking (RMSD in Å), Table S5: Docking results and ligand interactions (Co-crystallized 1DX6), Table S6: Docking results and ligand interactions (Co-crystallized 6EQM), Table S7: Spectra of the synthesized flavone derivatives.
