# Supplementary material for: Synthesis, In Silico and In Vitro Evaluation of Some Flavone Derivatives for Acetylcholinesterase and BACE-1 Inhibitory Activity
Source: Molecules. 2020 Sep 5;25(18):4064. doi: 10.3390/molecules25184064 (PMC7570966; doi:10.3390/molecules25184064)
Supplement: Supplementary file 1 [file molecules-25-04064-s001.pdf]

SUPPORTING INFORMATION

Synthesis, In Silico and In Vitro Evaluation of Some Flavone Derivatives for Acetylcholinesterase and BACE-1 Inhibitory Activity

Thai-Son Tran [1,2], Thanh-Dao Tran [1], The-Huan Tran [2], Thanh-Tan Mai [1], Ngoc-Le Nguyen [3], Khac-Minh Thai [1,*] and Minh-Tri Le [1,4,*]

[1] Department of Medicinal Chemistry, Faculty of Pharmacy, University of Medicine and Pharmacy at Ho Chi Minh City, Ho Chi Minh City 700000, Vietnam; ttson@huemed-univ.edu.vn (T.-S.T.); tranthanhdao@uphcm.edu.vn (T.-D.T.); mthtan@ump.edu.vn (T.-T.M.)

[2] Department of Pharmaceutical Chemistry, Faculty of Pharmacy, College of Medicine and Pharmacy, Hue University, Hue City 530000, Vietnam; tthuan@hueuni.edu.vn

[3] Department of Pharmaceutics and Industrial Pharmacy, Faculty of Pharmacy, South Can Tho University, Can Tho City 640000, Vietnam; dsngocle1984@gmail.com

[4] School of Medicine, Vietnam National University Ho Chi Minh City, Ho Chi Minh City 700000, Vietnam

* Correspondence: leminhtri@ump.edu.vn (M.-T.L.); thaikhacminh@gmail.com or thaikhacminh@uphcm.edu.vn (K.-M.T.); Tel.: +84-28-3855-2225 or +84-909-680-385 (K-M.T.); +84-903-718-190 (M.-T.L.); Fax: +84-28-3822-5435 (K.-M.T.)

Table S1. Selected descriptors used for building 2D-QSAR models reported in the previous published work

Table S2. Values of selected descriptors used in the prediction of pIC_{50} of the synthesized flavone derivatives (AChE)

Table S3. Values of selected descriptors used in the prediction of pIC_{50} of the synthesized flavone derivatives (BACE-1)

Table S4. Results of re-docking (RMSD in Å)

Table S5. Docking results and ligand interactions (Co-crystallized 1DX6)

Table S6. Docking results and ligand interactions (Co-crystallized 6EQM)

Table S7. Spectra of the synthesized flavone derivatives

Table S1. Selected descriptors used for building 2D-QSAR models reported in the previous published work [1]

Code	Category	Description
BCUT_SlogP_3	Adjacency and distance matrix	A Burden's parameter using atomic contribution to logP (using the Wildman and Crippen SlogP method [2]) instead of partial charge.
BCUT_PEOE_1	Adjacency and distance matrix	A descriptor relating topological shape and partial charges
petitjean	Adjacency and distance matrix	Value of (diameter - radius) / diameter.
reactive	Physical property	An indicator of the presence of reactive groups. A non-zero value indicates that the molecule contains a reactive group. The table of reactive groups is based on the Oprea set [3] and includes metals, phospho-, N/O/S-N/O/S single bonds, thiols, acyl halides, Michael Acceptors, azides, esters, etc.
logS	Physical property	The log of the aqueous solubility (mol/L).
PEOE_VSA-0, PEOE_VSA+1, PEOE_VSA-3, PEOE_VSA-6	Partial charge	Sum of the proximate accessible *van der Waals* surface area (Å^2), v_i, calculation for each atom over all the atoms *i*, such that partial charge of atom *i* is in a specified range.
SlogP_VSA2, SlogP_VSA3, SlogP_VSA5	Subdivided surface areas	Sum of the proximate accessible *van der Waals* surface area (Å^2), v_i, calculated for each atom over all the atoms, such that partition coefficient for atom *i* is in a specified range
SMR_VSA2	Subdivided surface areas	Sum of the proximate accessible *van der Waals* surface area (Å^2), v_i, calculation for each atom over all the atoms *i*, such that molar refractivity for atom *i* is in a specified range.
a_ICM	Atom counts and bond counts	The entropy of the element distribution in the molecule (including implicit hydrogens but not lone pair pseudo-atoms)
chiral_u	Atom counts and bond counts	The number of unconstrained chiral centers.
rings	Atom counts and bond counts	The number of rings
a_Nn	Atom counts and bond counts	The number of nitrogen atoms.

Table S2. Values of selected descriptors used in the prediction of pIC_{50} of the synthesized flavone derivatives (AChE)

Compound	BCUT_SLOGP_3	reactive	PEOE_VSA+1	PEOE_VSA-3	SlogP_VSA2	SMR_VSA2	**Predicted pIC_{50}**
B1 (Baicalein)	2.52	1	48.98	0.00	0.00	0.00	**4.66**
B2	2.53	1	74.58	0.00	0.00	0.00	**4.59**
B3	2.54	1	95.95	0.00	0.00	0.00	**4.55**
B4	2.53	1	121.54	0.00	0.00	0.00	**4.44**
B5	2.54	1	157.82	0.00	0.00	0.00	**4.32**
B6	2.58	1	183.41	0.00	0.00	0.00	**4.32**
B7	2.52	1	48.98	0.00	71.59	0.00	**4.52**
B8	2.54	1	48.98	0.00	71.59	0.00	**4.56**
D1 (Diosmetin)	2.49	1	74.43	0.00	0.00	0.00	**4.50**
D2	2.49	1	100.02	0.00	0.00	0.00	**4.42**
D3	2.50	1	121.40	0.00	0.00	0.00	**4.37**
D4	2.50	1	146.99	0.00	0.00	0.00	**4.26**
D5	2.50	1	112.82	0.00	0.00	0.00	**4.39**
D6	2.51	1	144.88	0.00	0.00	0.00	**4.31**
D7	2.49	1	117.08	0.00	0.00	0.00	**4.35**
Galanthamine	2.77	0	114.49	0.00	12.94	0.00	**5.14**

Table S3. Values of selected descriptors used in the prediction of pIC_{50} of the synthesized chalcone derivatives (BACE-1)

Compound	petitjean	BCUT_PEOE_1	a_ICM	chiral_u	rings	a_nN	PEOE_VSA-0	PEOE_VSA-6	logS	SlogP_VSA3	SlogP_VSA5	**Predicted pIC_{50}**
B1 (Baicalein)	0.50	-0.72	1.46	0	3	0	0.00	25.81	-3.46	0.00	0.00	**3.66**
B2	0.45	-0.62	1.44	0	3	0	0.00	15.28	-4.29	0.00	70.77	**4.82**
B3	0.50	-0.58	1.41	0	3	0	37.50	15.28	-4.94	41.85	0.00	**5.11**
B4	0.46	-0.67	1.39	0	3	0	0.00	15.28	-5.28	41.85	0.00	**4.35**
B5	0.46	-0.66	1.36	0	3	0	0.00	10.02	-6.19	62.78	0.00	**4.60**
B6	0.47	-0.65	1.29	0	6	0	6.63	10.02	-10.00	0.00	0.00	**5.18**
B7	0.50	-0.57	1.49	0	3	0	0.00	2.50	-5.61	0.00	0.00	**4.73**
B8	0.46	-0.57	1.46	0	3	0	0.00	2.50	-6.21	0.00	0.00	**4.75**
D1 (Diosmetin)	0.45	-0.87	1.48	0	3	0	61.27	28.31	-3.51	0.00	35.38	**3.55**
D2	0.50	-0.60	1.46	0	3	0	61.27	17.78	-4.34	0.00	106.15	**5.83**
D3	0.46	-0.56	1.43	0	3	0	98.77	17.78	-4.99	41.85	35.38	**6.04**
D4	0.47	-0.66	1.42	0	3	0	61.27	17.78	-5.33	41.85	35.38	**5.24**
D5	0.50	-0.59	1.44	0	3	0	61.27	12.52	-4.75	0.00	141.54	**6.20**
D6	0.46	-0.56	1.40	0	3	0	117.52	12.52	-5.73	62.78	35.38	**6.34**
D7	0.47	-0.65	1.42	0	5	0	98.04	12.78	-8.47	0.00	35.38	**6.07**
Umibecestat	0.50	-0.56	2.09	2	3	5	74.26	9.29	-5.92	20.93	16.79	**7.67**
Quercetin	0.50	-0.63	-1.52	0	3	0	61.27	41.34	-2.77	25.39	5.24	**5.24**

Table S4. Results of re-docking (RMSD in Å)

Ligand	AChE	BACE-1
	1DX6	6EQM
1	0.49	0.75
2	0.50	0.80
3	0.89	0.92

- Ligand 1: separated from the complex (native form, not prepared).

- Ligand 2: separated from the complex and re-prepared using appropriate procedure indicated in the section of Materials and Methods

- Ligand 3: built and prepared from the beginning.

Table S5: Docking results and ligand interaction (Co-crystallized 1DX6)

Compound	Observed pIC50	Docking score (kJ.mol⁻¹)	Interactions
B1 (Baicalein)	4.43	–27.06	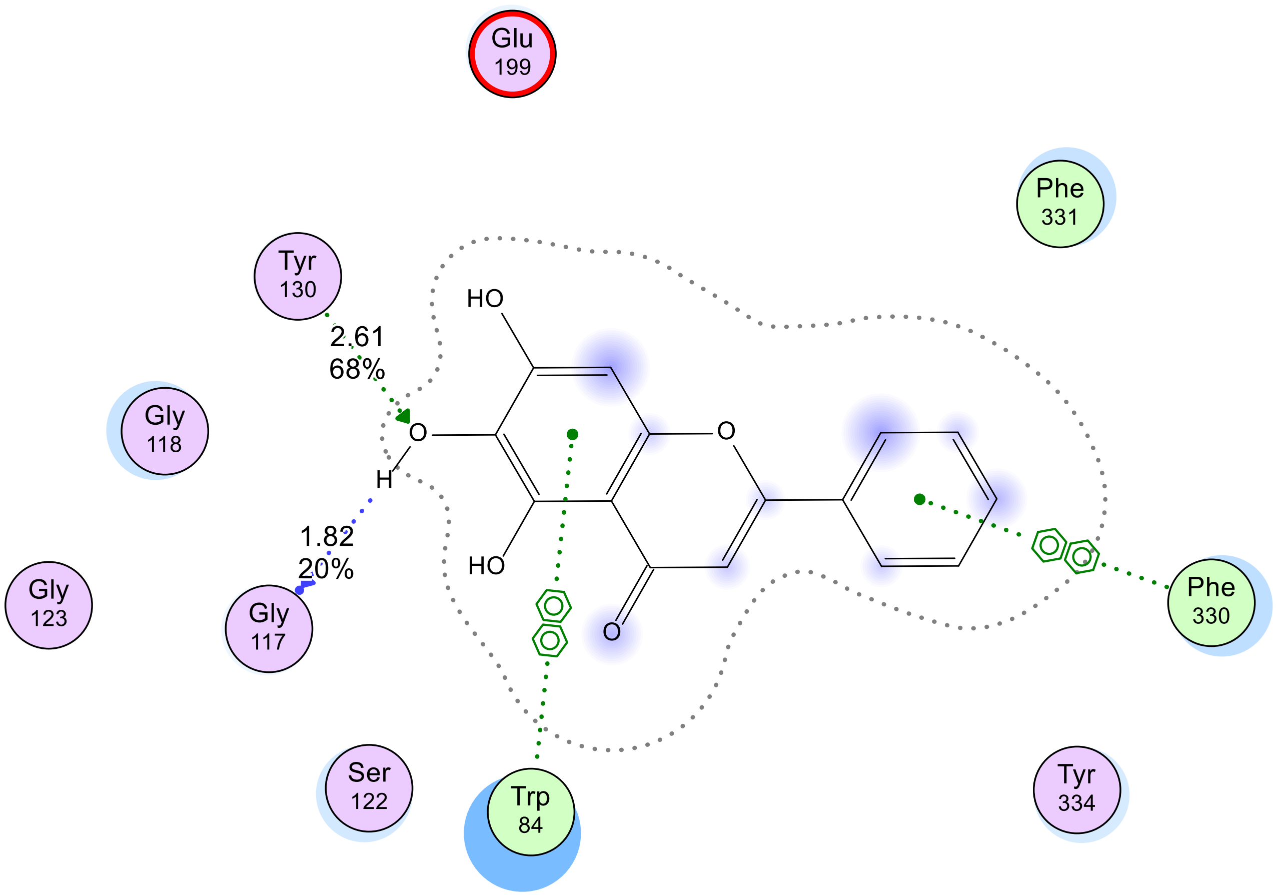

B2	4.59	−17.67	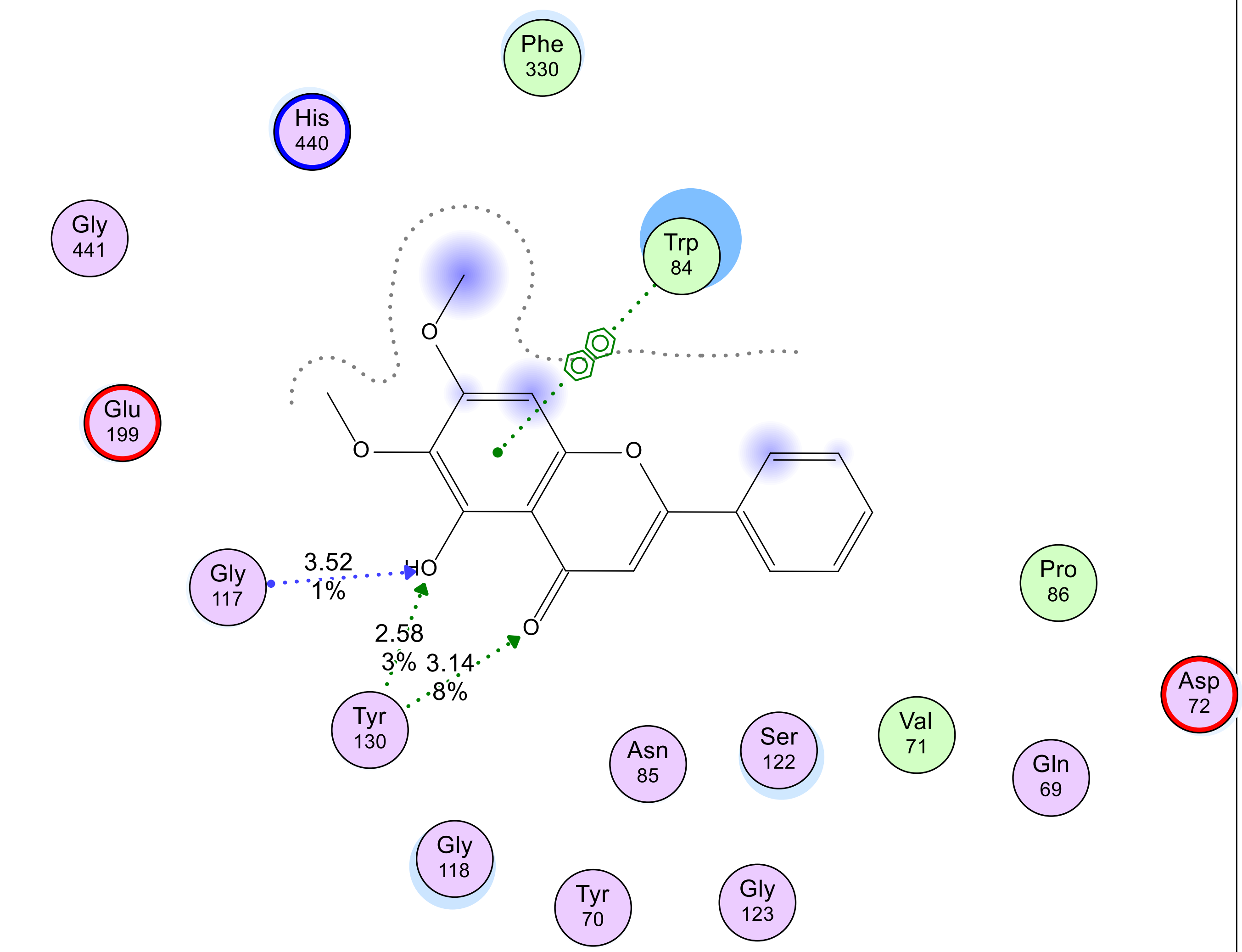 Phe 330 His 440 Gly 441 Trp 84 Glu 199 O O O O Gly 117 3.52 1% HO 2.58 3% 3.14 8% O Pro 86 Asp 72 Tyr 130 Asn 85 Ser 122 Val 71 Gln 69 Gly 118 Tyr 70 Gly 123

B3	4.52	−17.55	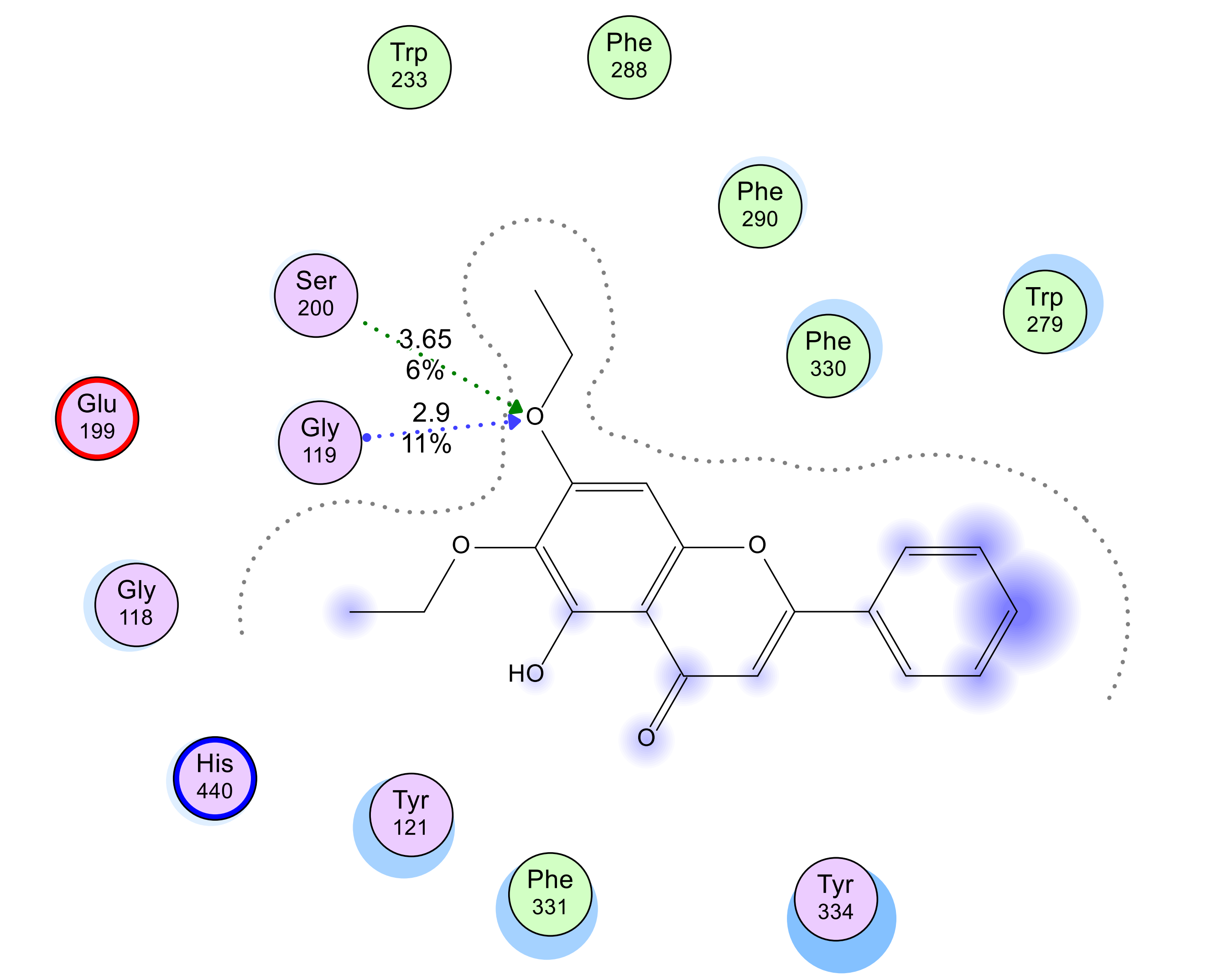Trp 233
Phe 288
Phe 290
Ser 200
Phe 330
Trp 279
3.65
6%
Glu 199
Gly 119
2.9
11%
O
O
O
Gly 118
HO
O
His 440
Tyr 121
Phe 331
Tyr 334

B4	4.29	−10.36	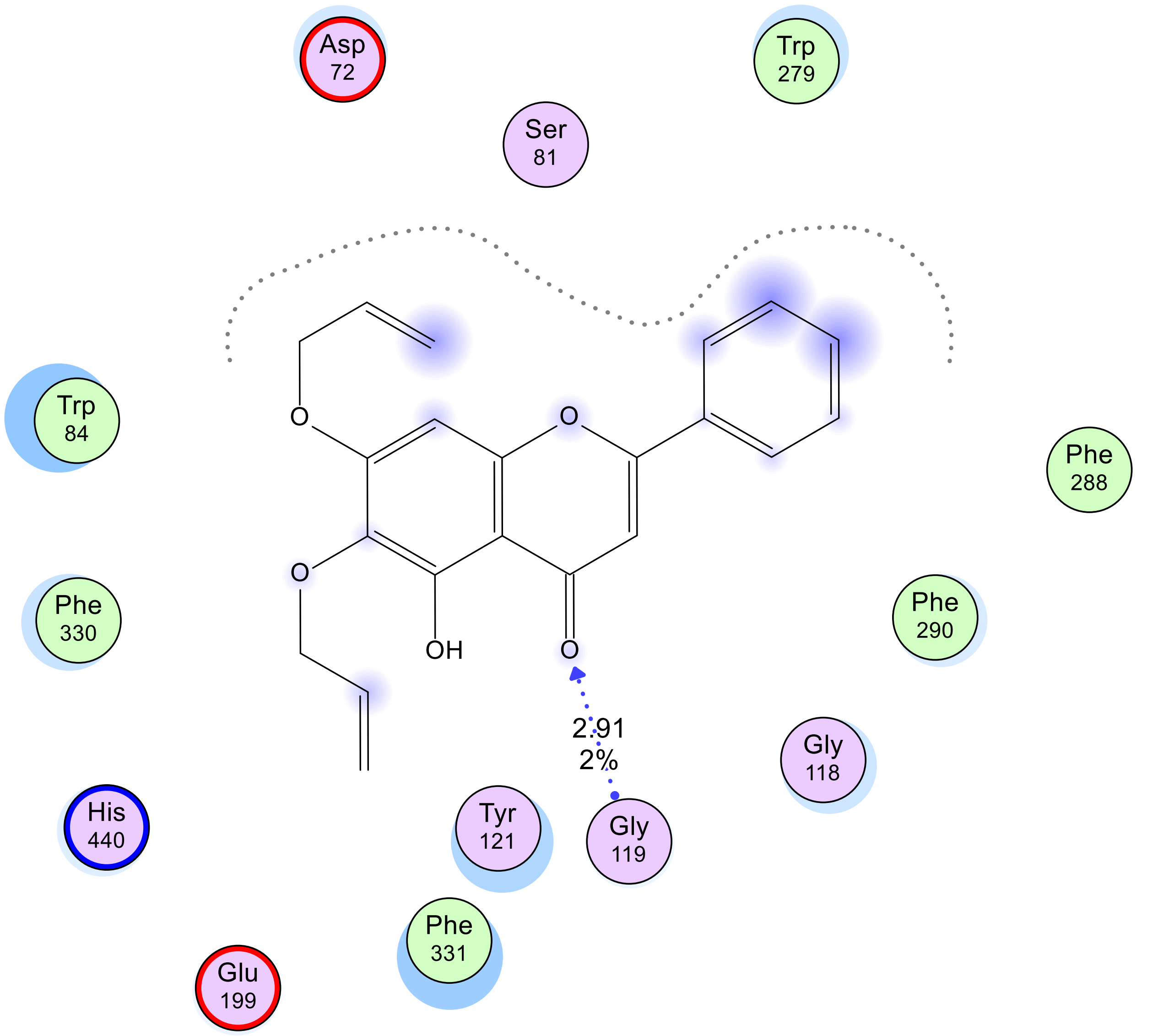Asp<br>72<br>Trp<br>279<br>Ser<br>81<br>Trp<br>84<br>Phe<br>288<br>Phe<br>330<br>Phe<br>290<br>O<br>O<br>O<br>OH<br>O<br>2.91<br>2%<br>Gly<br>118<br>His<br>440<br>Tyr<br>121<br>Gly<br>119<br>Phe<br>331<br>Glu<br>199

B5	3.72	−10.49	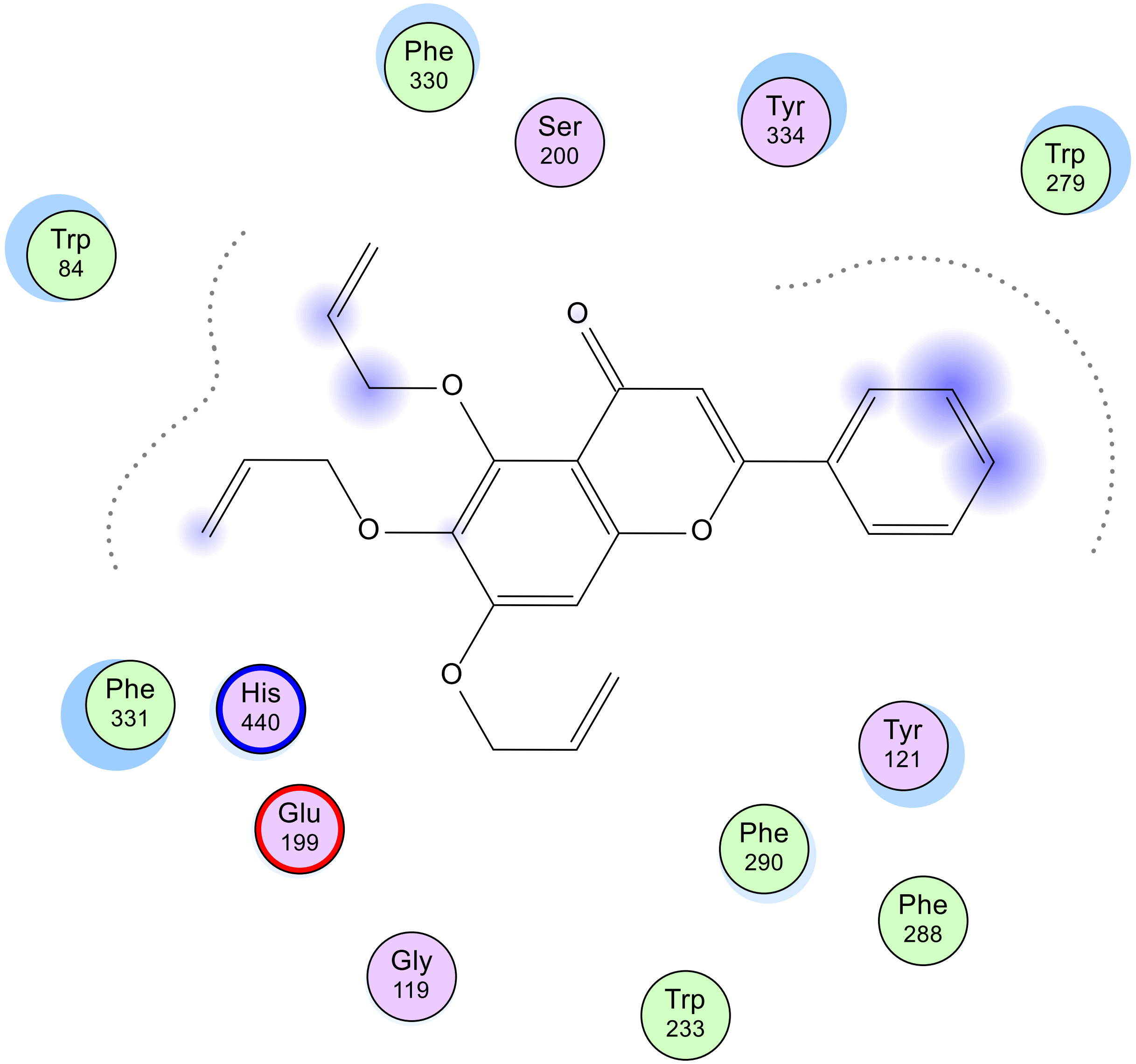Phe 330<br>Ser 200<br>Tyr 334<br>Trp 279<br>Trp 84<br>O<br>O<br>O<br>O<br>O<br>Phe 331<br>His 440<br>Glu 199<br>Tyr 121<br>Phe 290<br>Phe 288<br>Gly 119<br>Trp 233

B6	4.10	–15.71	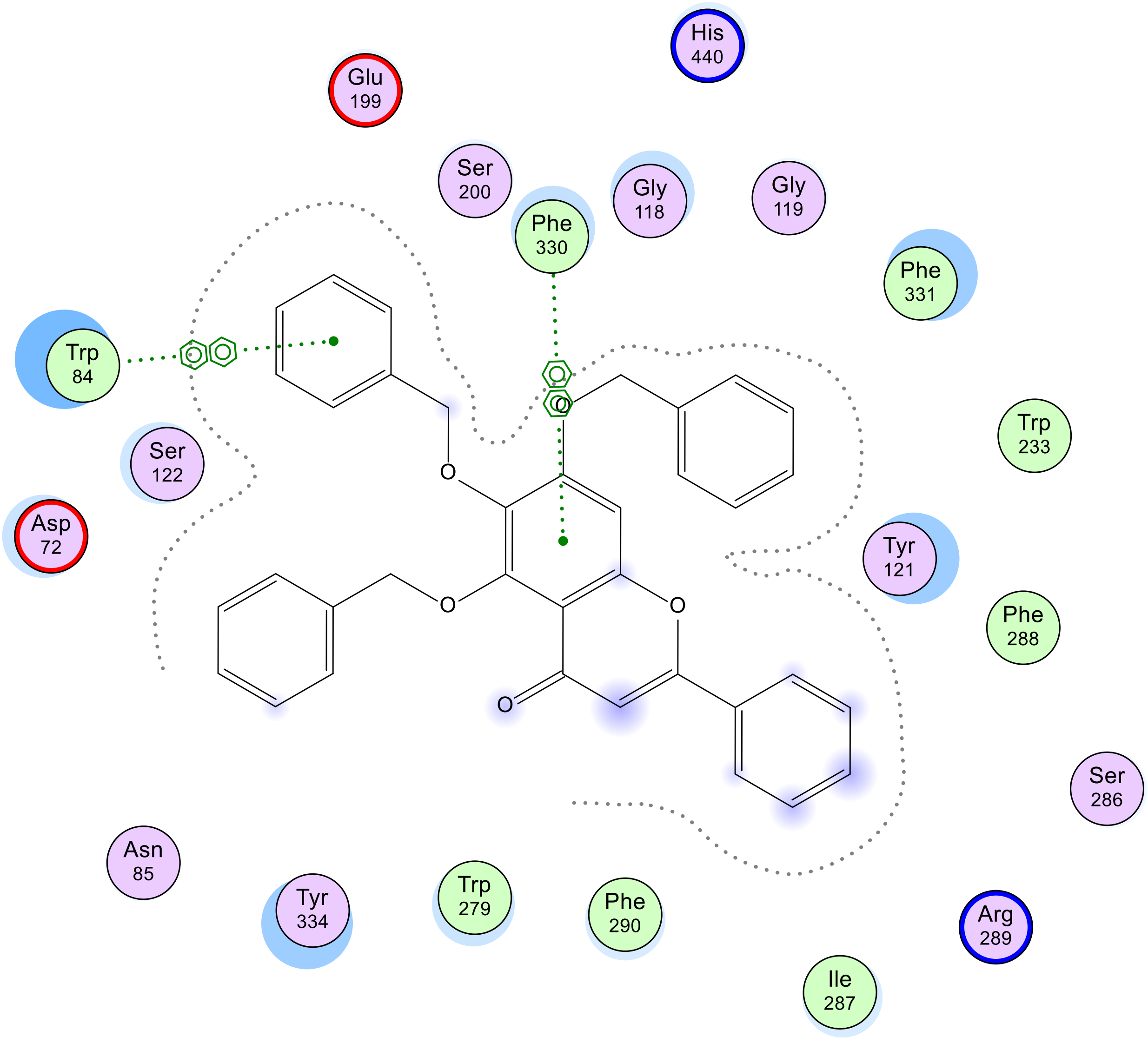His 440<br>Glu 199<br>Ser 200<br>Gly 118<br>Gly 119<br>Phe 330<br>Phe 331<br>Trp 84<br>Ser 122<br>Asp 72<br>Trp 233<br>Tyr 121<br>Phe 288<br>Ser 286<br>Asn 85<br>Tyr 334<br>Trp 279<br>Phe 290<br>Arg 289<br>Ile 287<br>O<br>O<br>O<br>O<br>O

B7	4.29	−17.91	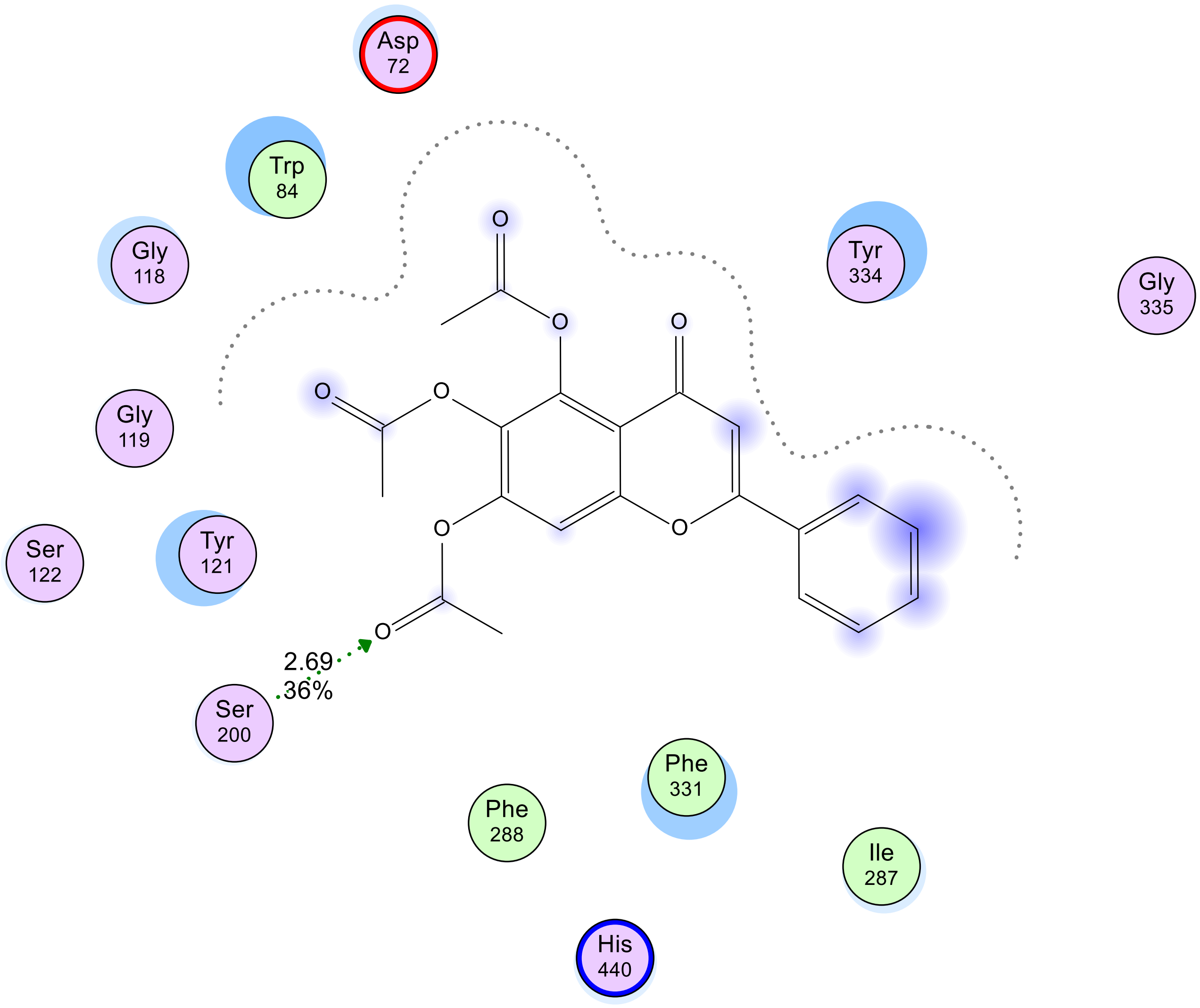

Asp 72
Trp 84
Gly 118
Gly 119
Ser 122
Tyr 121
Tyr 334
Gly 335
2.69
36%
Ser 200
Phe 331
Phe 288
Ile 287
His 440

B8	4.18	−19.61	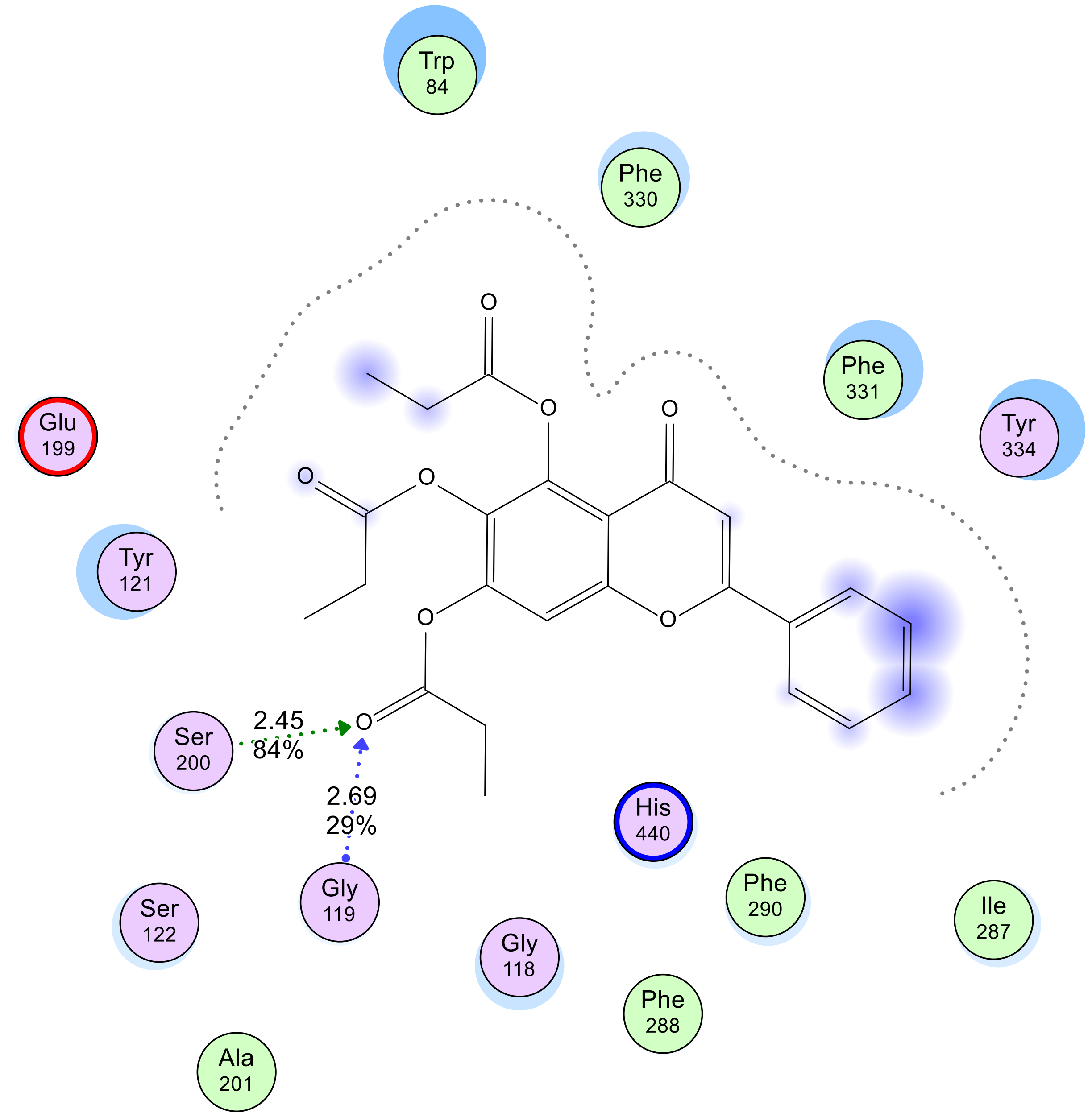Trp 84<br>Phe 330<br>Phe 331<br>Tyr 334<br>Glu 199<br>Tyr 121<br>O<br>O<br>O<br>O<br>O<br>O<br>O<br>O<br>Ser 200<br>2.45<br>84%<br>2.69<br>29%<br>His 440<br>Ser 122<br>Gly 119<br>Gly 118<br>Phe 290<br>Ile 287<br>Phe 288<br>Ala 201

D1 (Diosmetin)	3.83	−22.86	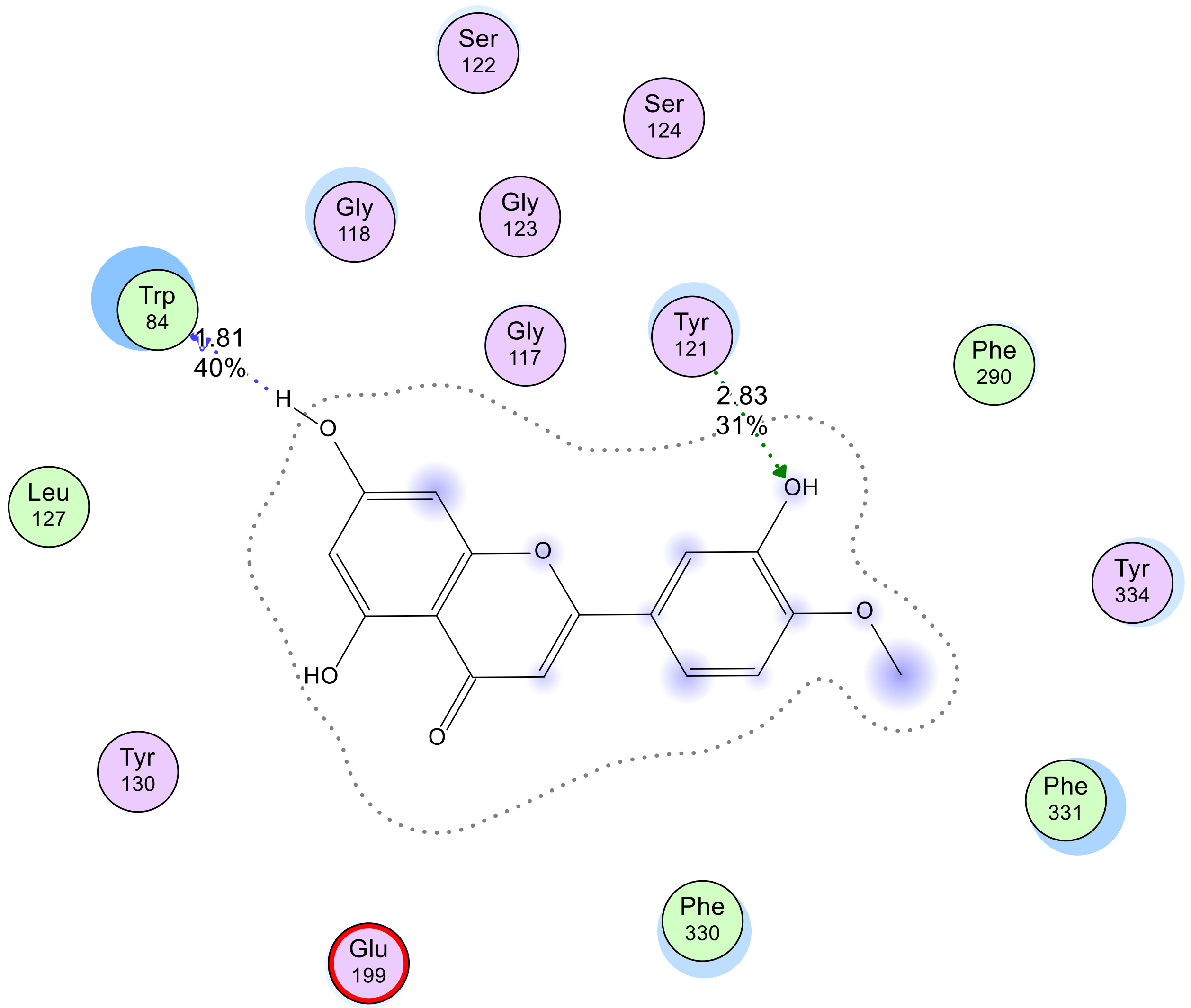

D2	4.06	−21.05	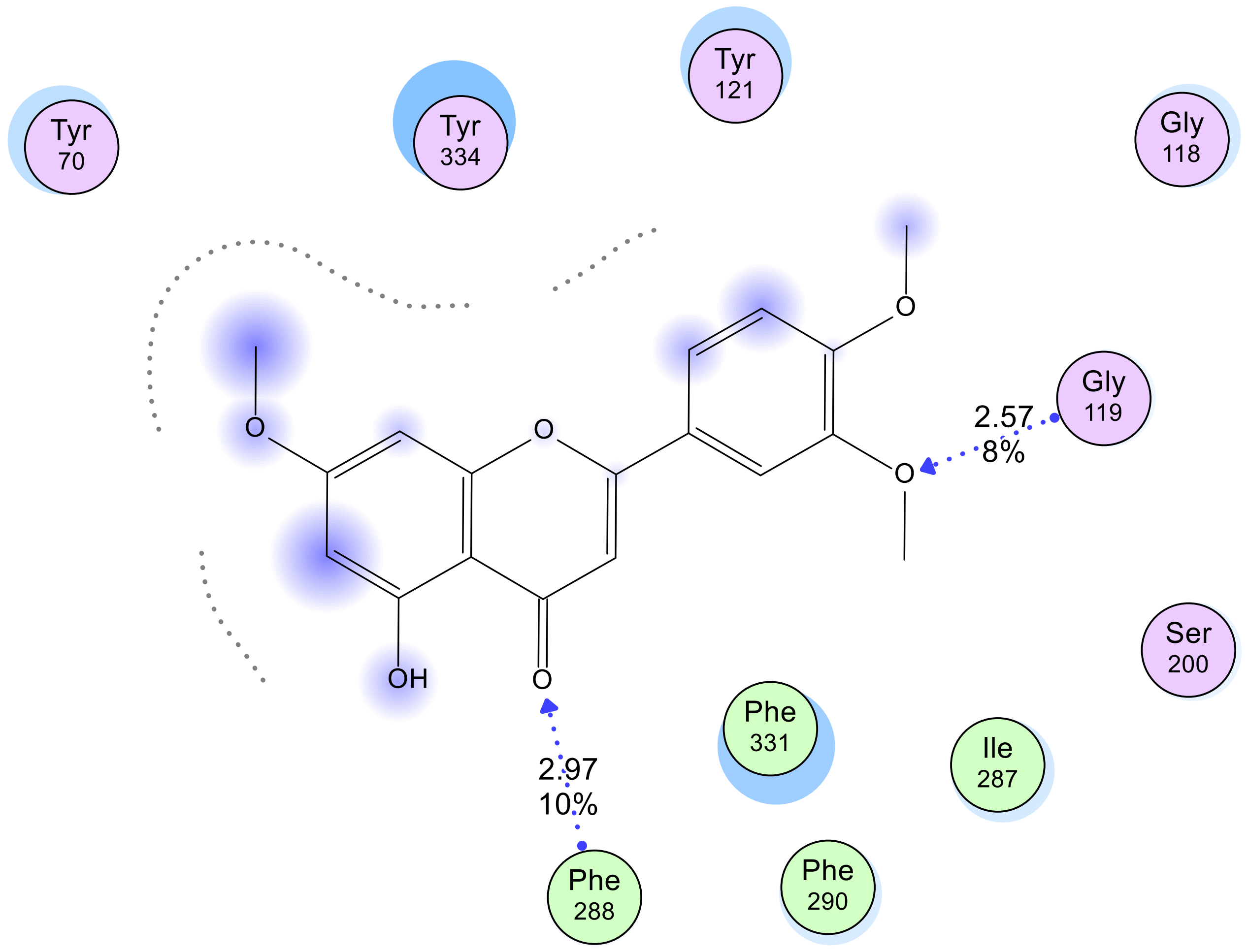Tyr 70
Tyr 334
Tyr 121
Gly 118
Gly 119
2.57
8%
O
O
O
O
OH
O
2.97
10%
Phe 331
Ile 287
Ser 200
Phe 288
Phe 290

D3	3.89	−20.90	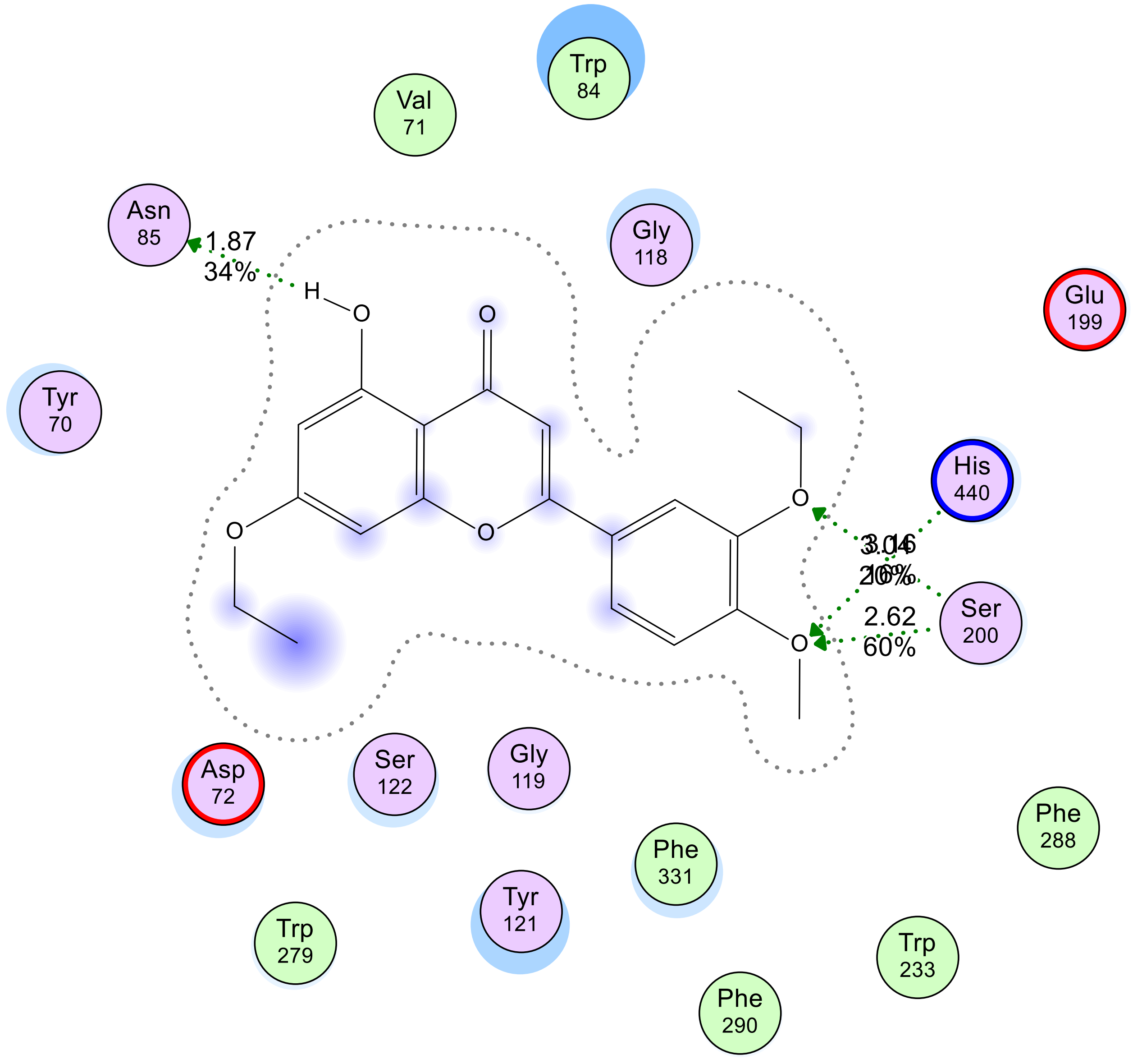

Trp 84
Val 71
Asn 85
1.87 34%
Gly 118
Glu 199
Tyr 70
His 440
Ser 200
2.62 60%
Asp 72
Ser 122
Gly 119
Phe 288
Phe 331
Tyr 121
Trp 279
Trp 233
Phe 290

D4	3.82	−15.22	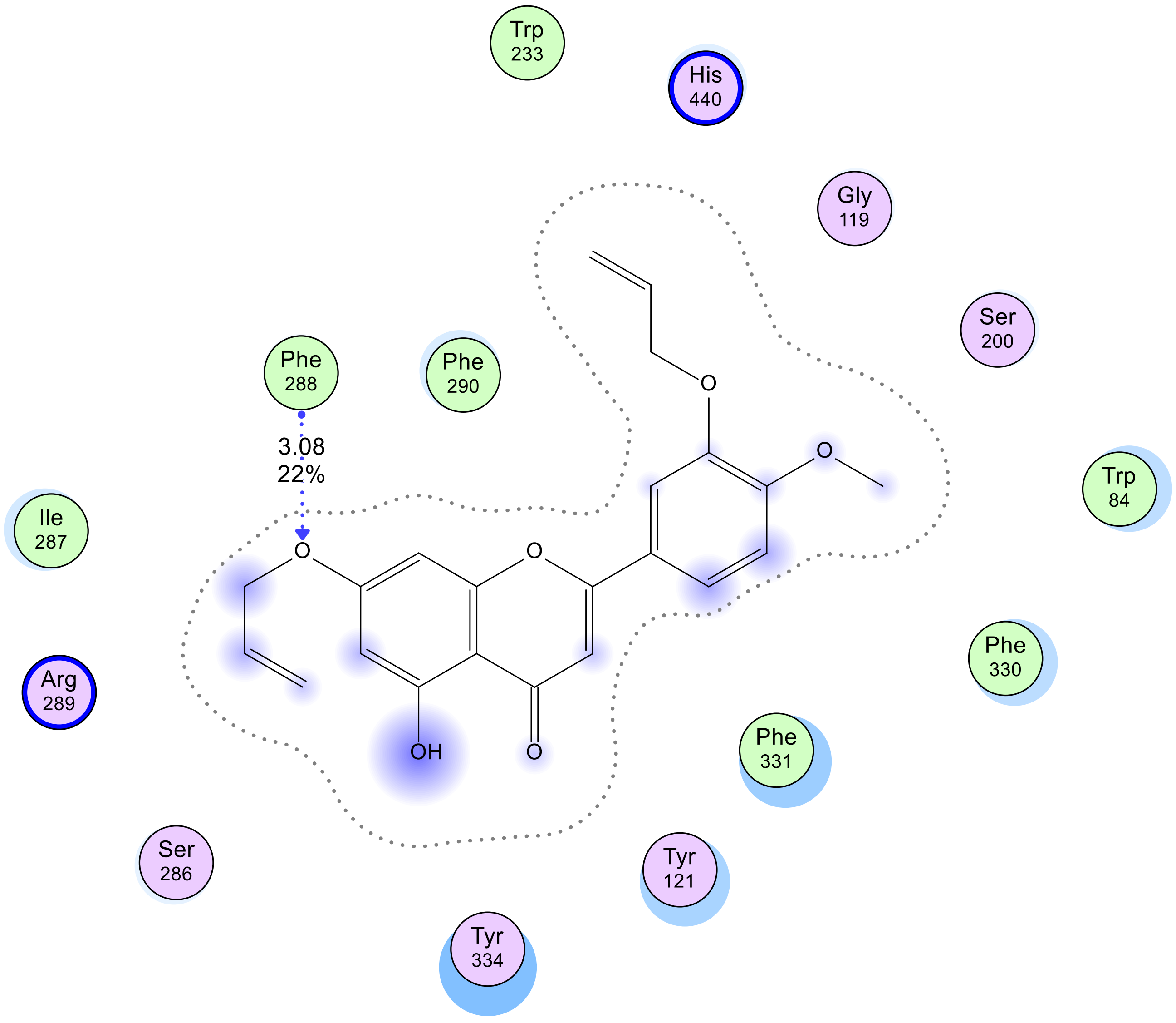Trp 233
His 440
Gly 119
Ser 200
Trp 84
Phe 288
Phe 290
3.08
22%
Ile 287
Arg 289
O
O
O
O
OH
O
Phe 330
Phe 331
Ser 286
Tyr 121
Tyr 334

D5	4.13	−21.80	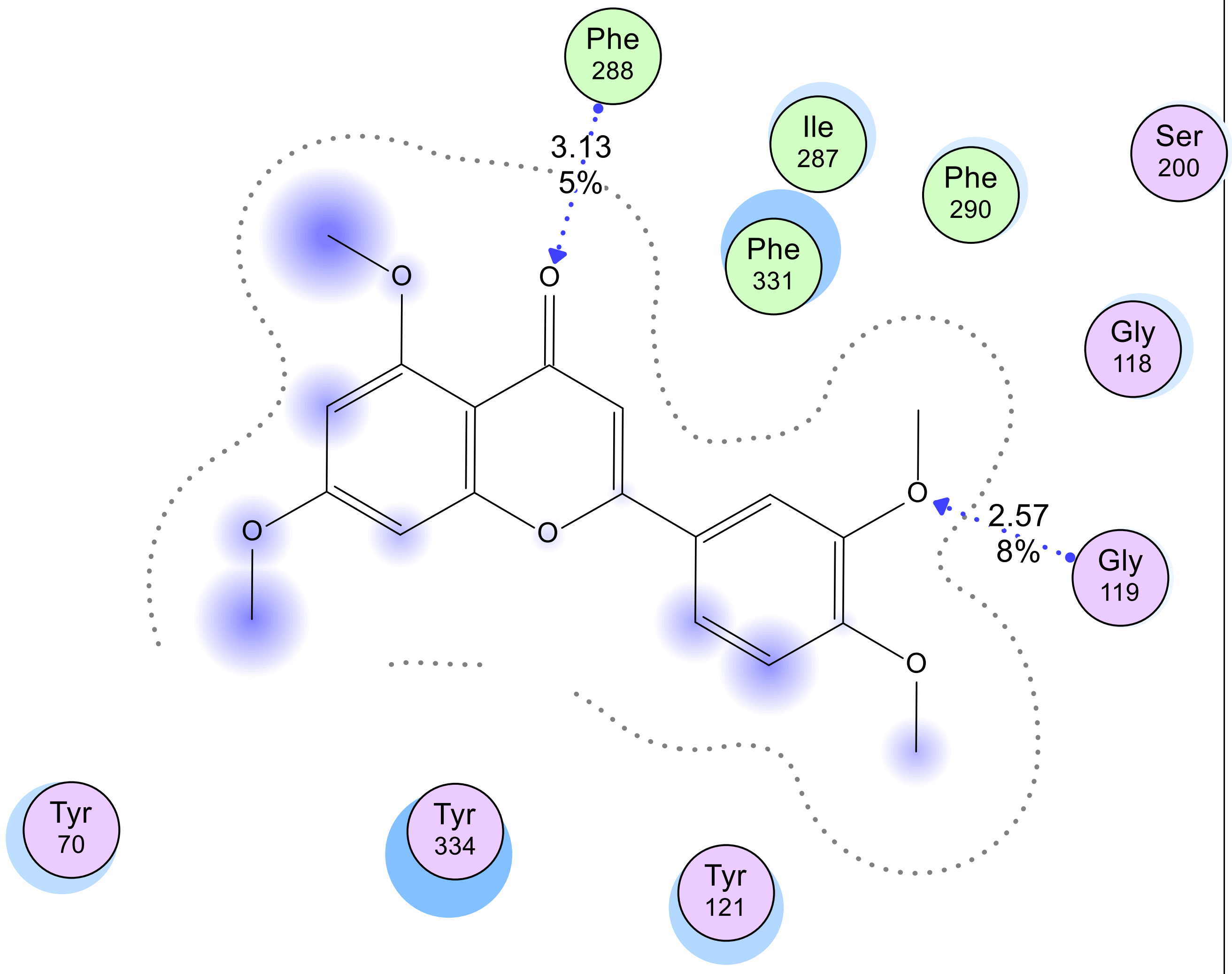Phe 288 3.13 5% Ile 287 Phe 290 Ser 200 Phe 331 Gly 118 O O O O O O 2.57 8% Gly 119 Tyr 70 Tyr 334 Tyr 121

D6	4.12	−20.39	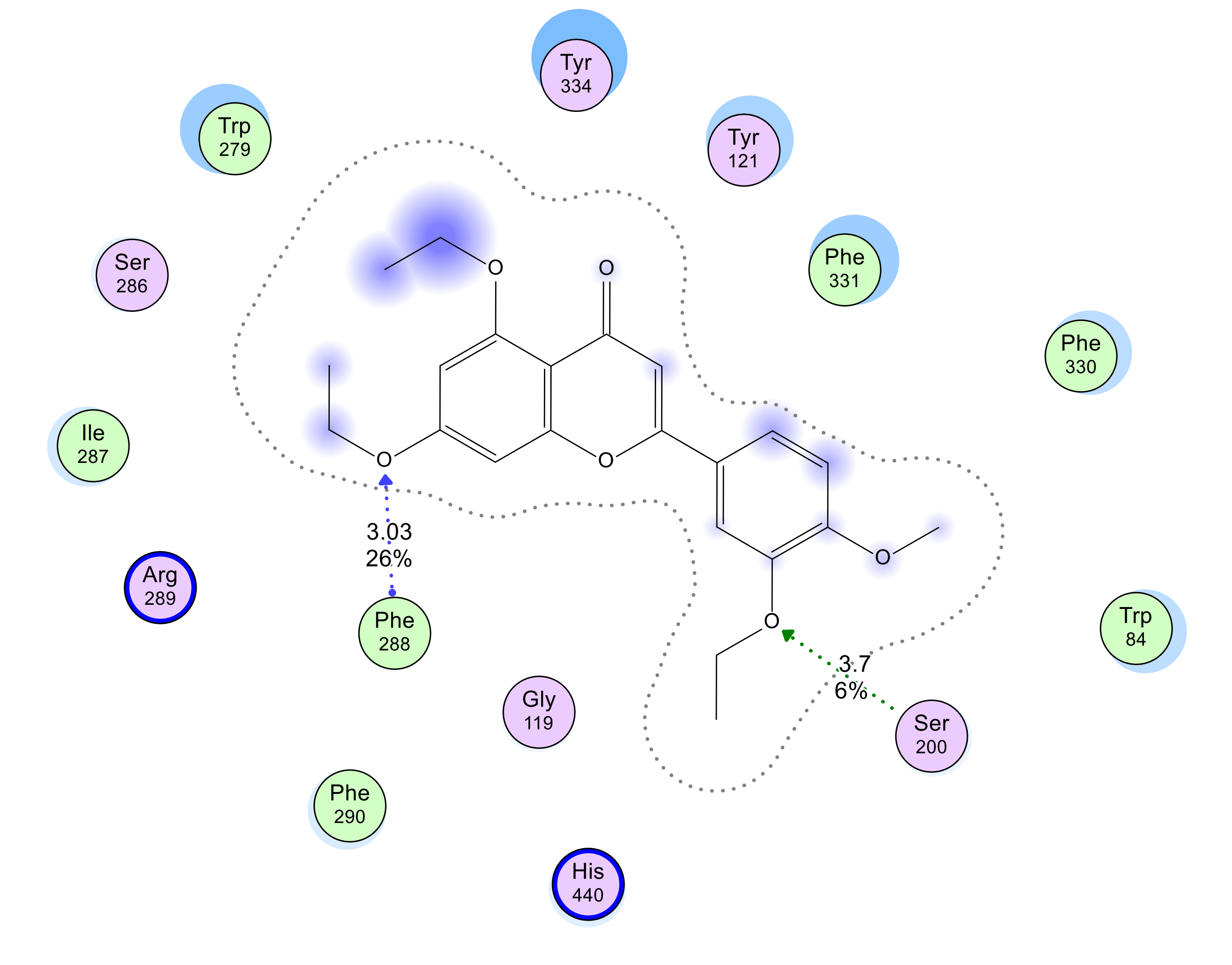

Tyr 334
Trp 279
Tyr 121
Ser 286
Phe 331
Phe 330
Ile 287
Arg 289
3.03
26%
Phe 288
Trp 84
3.7
6%
Gly 119
Ser 200
Phe 290
His 440
O

D7	3.47	−25.35	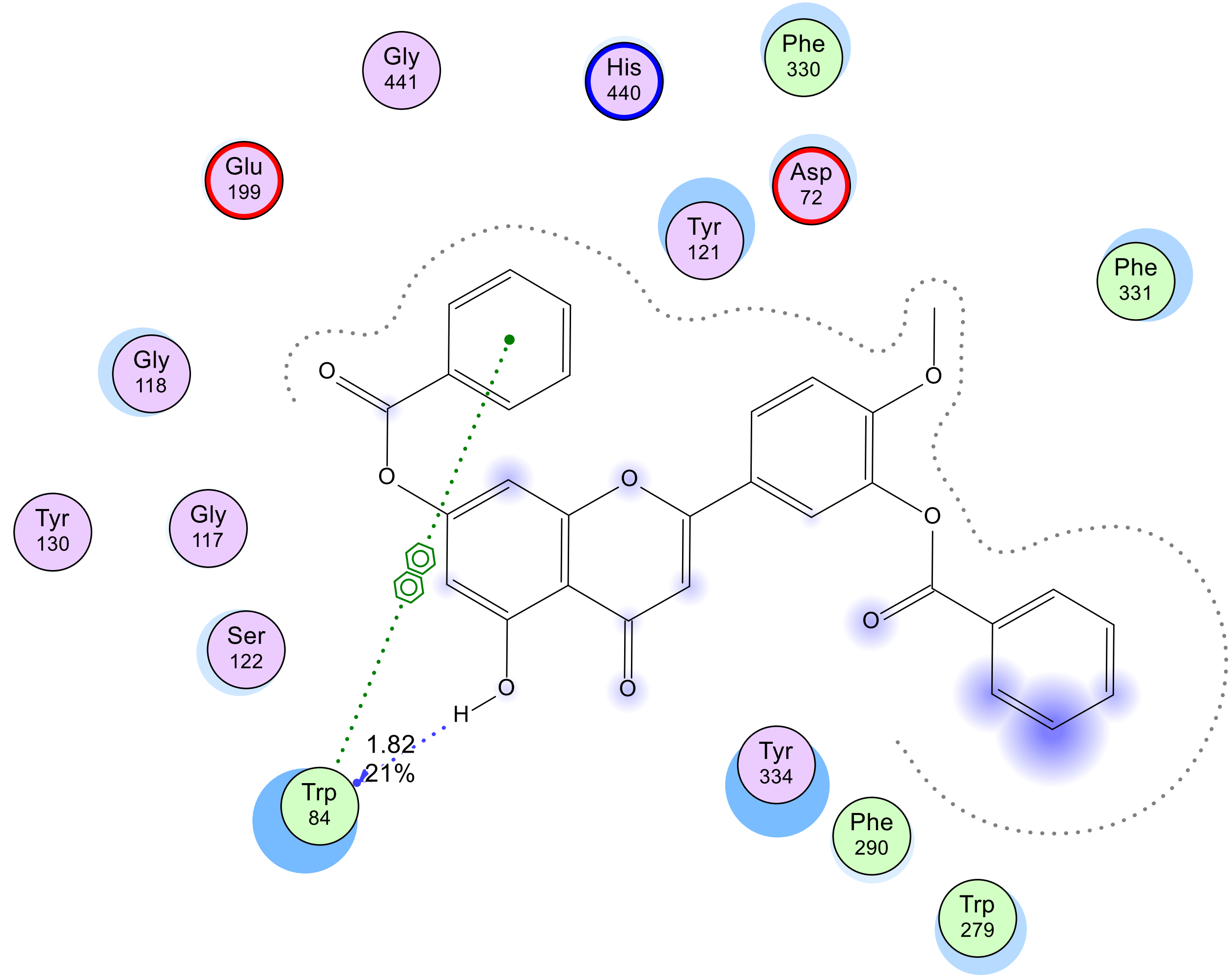Gly 441
His 440
Phe 330
Glu 199
Asp 72
Tyr 121
Phe 331
Gly 118
Tyr 130
Gly 117
Ser 122
Tyr 334
Phe 290
Trp 84
Trp 279
1.82
21%

Galanthamine	5.88	–33.28	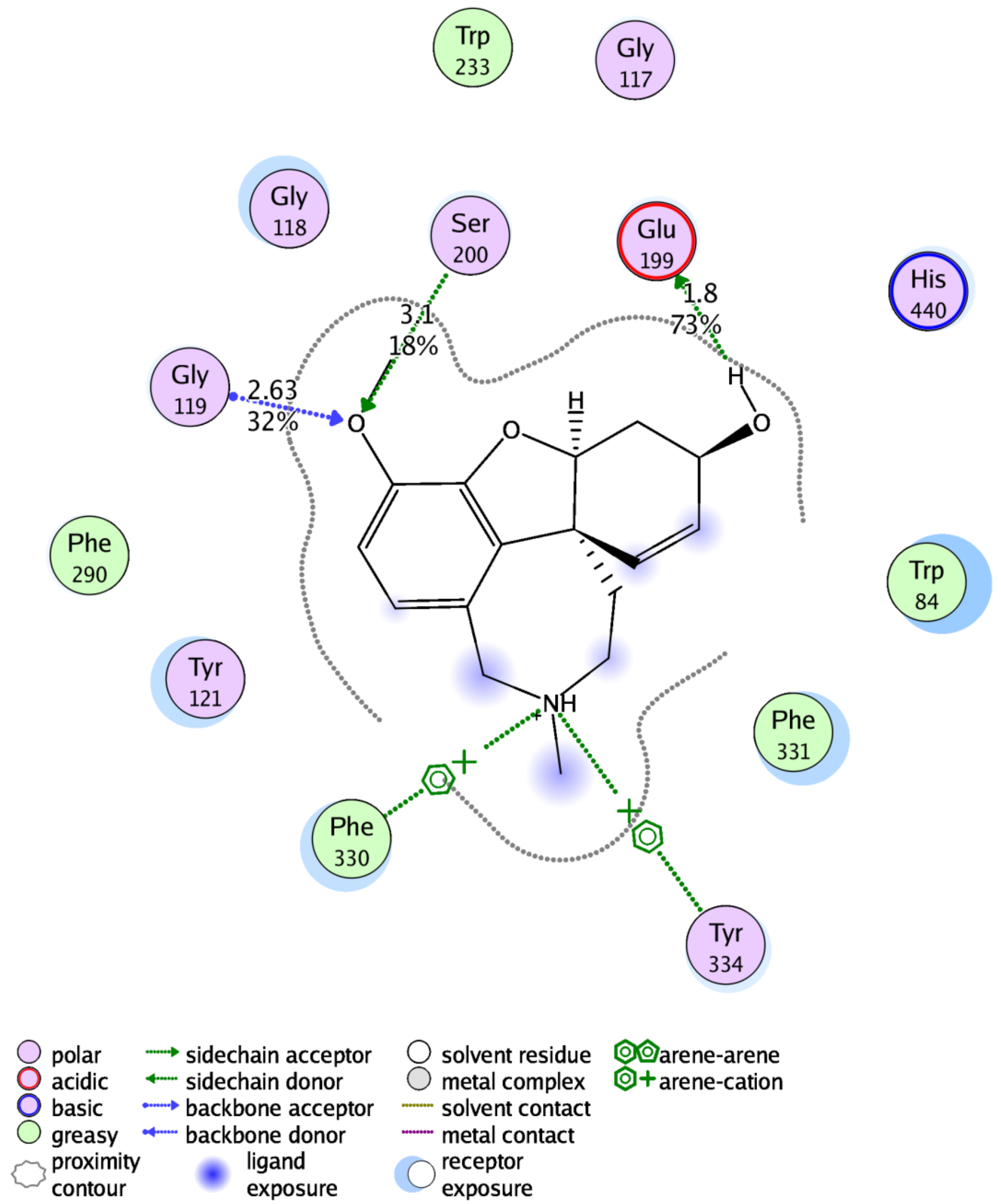

Table S6. Docking results and ligand interaction (Co-crystallized 6EQM)

Compound	Observed pIC50	Docking score (kJ.mol⁻¹)	Interactions
B1 (Baicalein)	4.16	–17.64	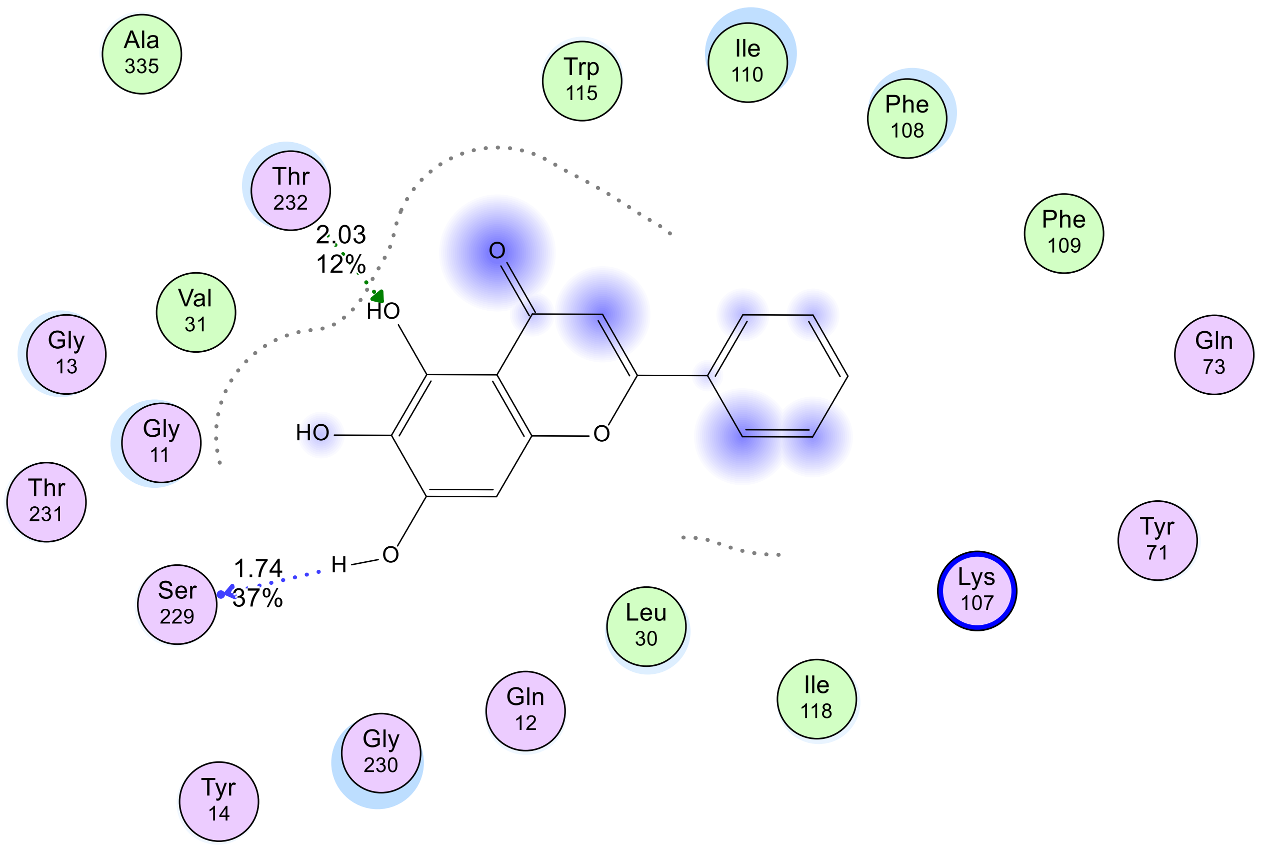

B2	4.36	−16.37	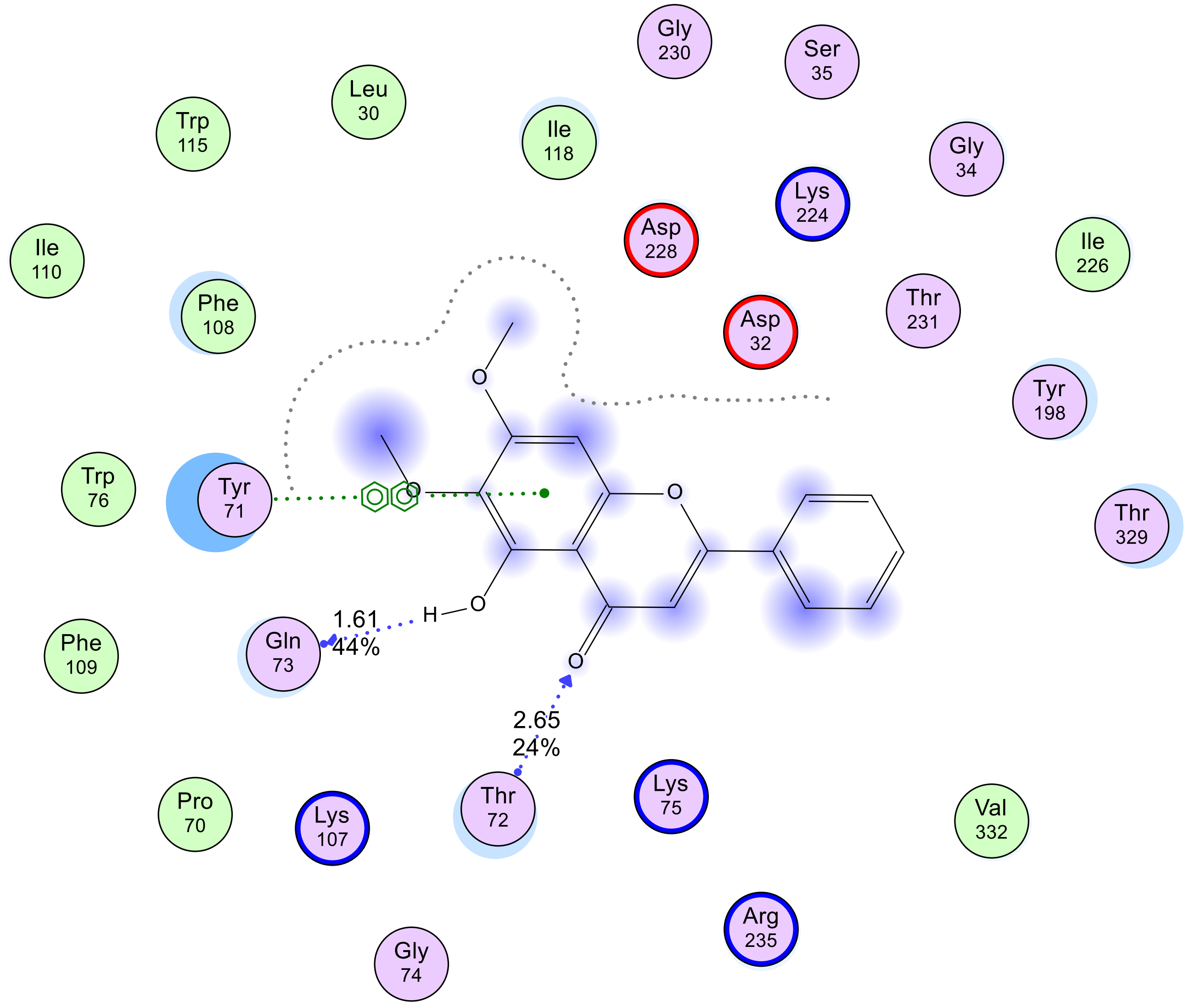

Gly 230
Ser 35
Leu 30
Trp 115
Ile 118
Gly 34
Lys 224
Asp 228
Ile 110
Ile 226
Phe 108
Thr 231
Asp 32
Tyr 198
Trp 76
Tyr 71
O
O
O
Thr 329
Phe 109
Gln 73
1.61
44%
H
O
O
2.65
24%
Pro 70
Lys 107
Thr 72
Lys 75
Val 332
Arg 235
Gly 74

B3	5.40	−12.35	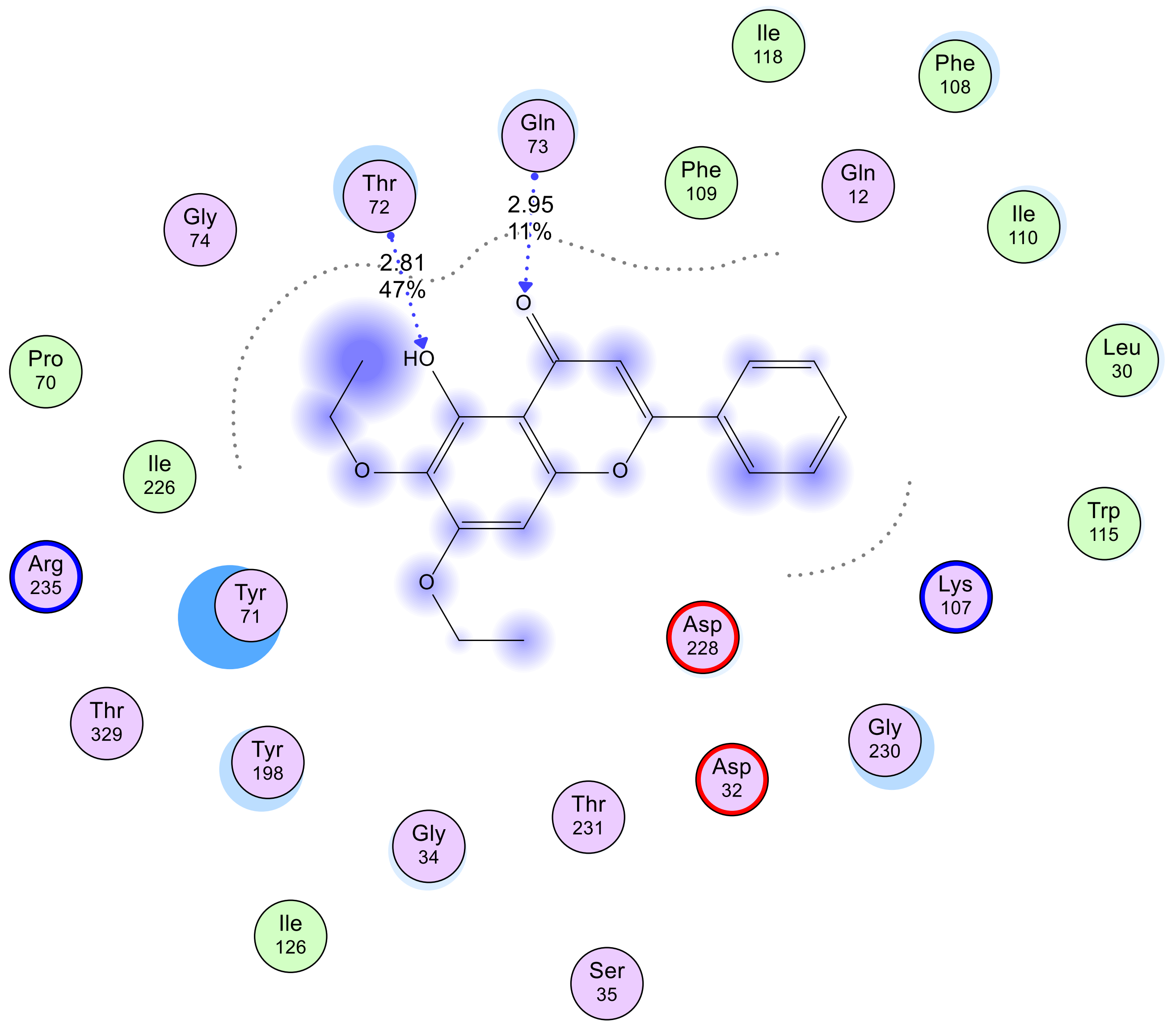

Ile 118
Phe 108
Gln 73
Thr 72
Phe 109
Gln 12
Gly 74
2.95
11%
Ile 110
2.81
47%
O
HO
Pro 70
Leu 30
O
O
Ile 226
Trp 115
Arg 235
Tyr 71
O
Lys 107
Asp 228
Thr 329
Tyr 198
Gly 230
Asp 32
Thr 231
Gly 34
Ile 126
Ser 35

B4	4.15	−10.84	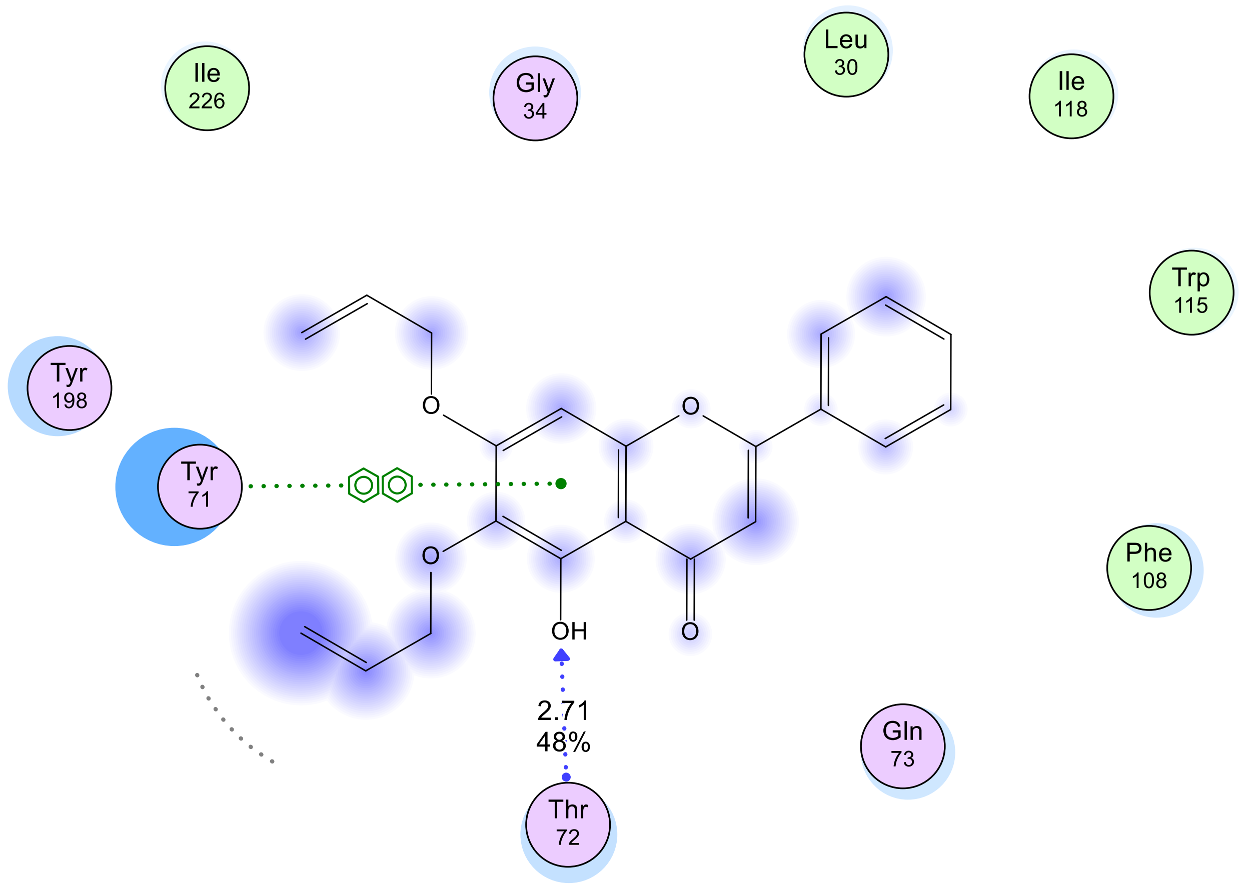

Ile 226
Gly 34
Leu 30
Ile 118
Trp 115
Tyr 198
Tyr 71
O
O
O
O
OH
2.71
48%
Phe 108
Gln 73
Thr 72

B5	4.77	−8.29	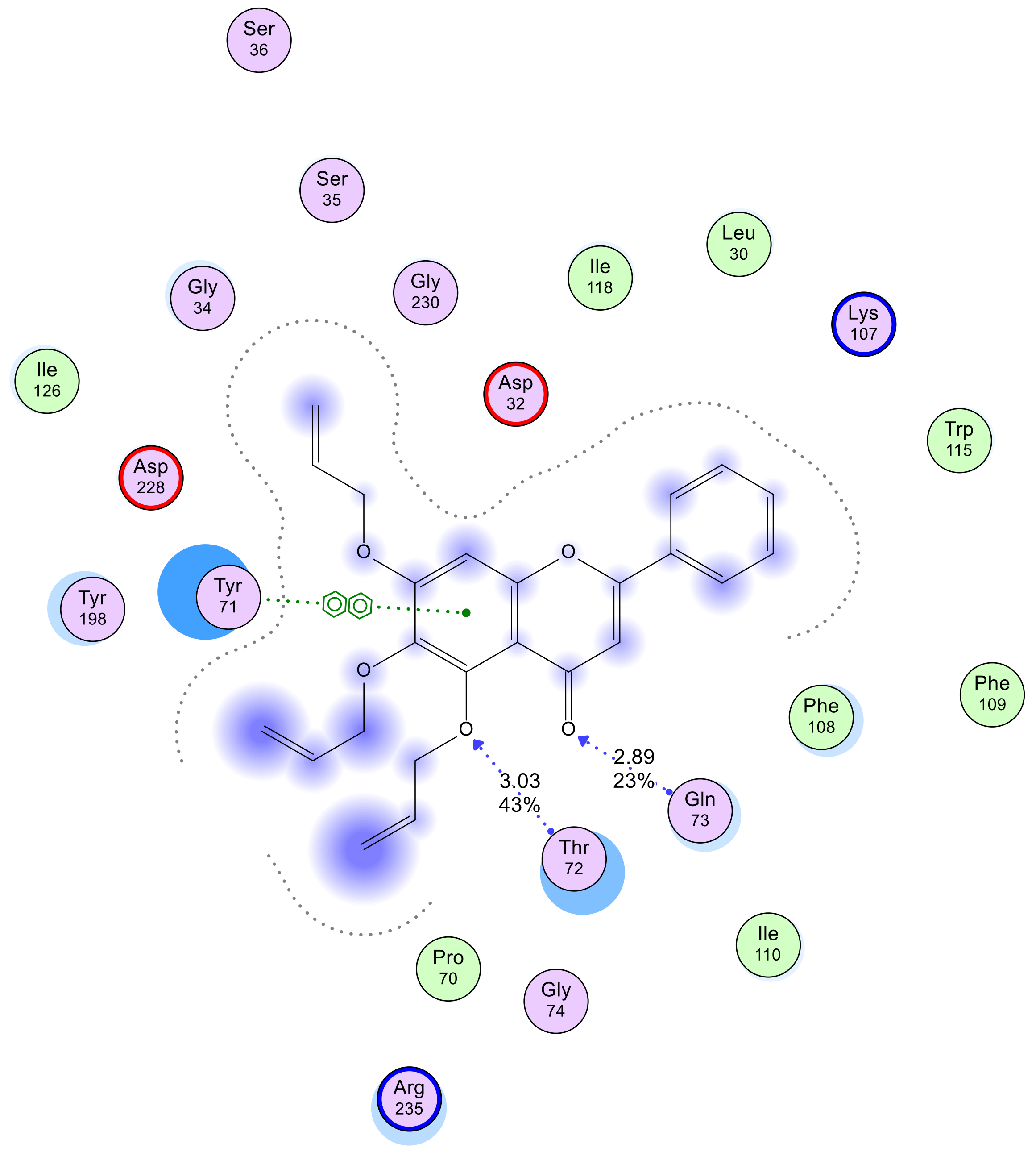

Ser 36
Ser 35
Gly 34
Gly 230
Ile 118
Leu 30
Lys 107
Ile 126
Asp 32
Asp 228
Trp 115
Tyr 198
Tyr 71
O
O
O
O
O
Phe 108
Phe 109
2.89 23%
3.03 43%
Gln 73
Thr 72
Ile 110
Pro 70
Gly 74
Arg 235

B6	4.90	−17.35	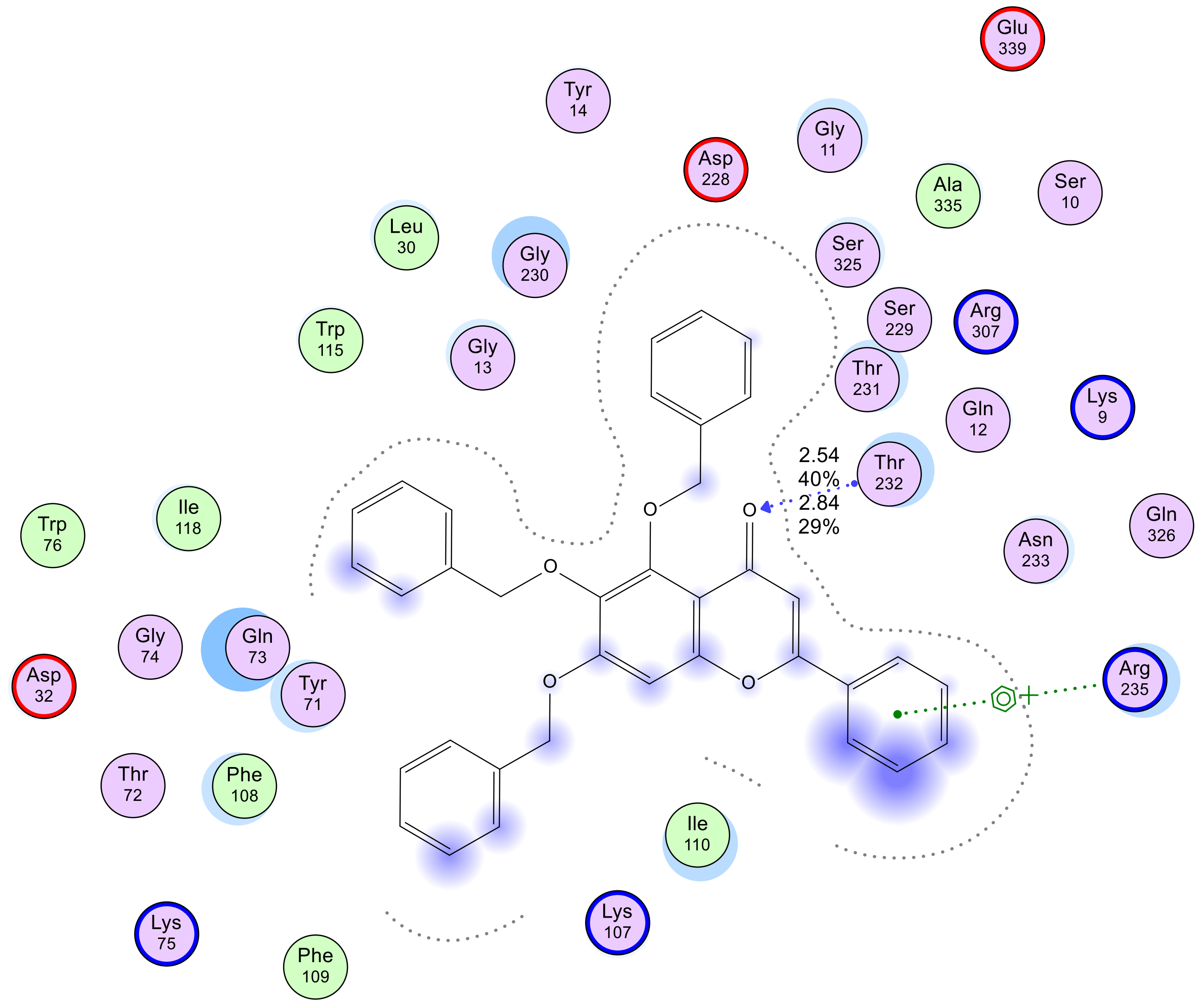

Glu 339
Tyr 14
Gly 11
Asp 228
Ala 335
Ser 10
Leu 30
Gly 230
Ser 325
Ser 229
Arg 307
Trp 115
Gly 13
Thr 231
Gln 12
Lys 9
2.54
40%
2.84
29%
Thr 232
Trp 76
Ile 118
Asn 233
Gln 326
Gly 74
Gln 73
Asp 32
Tyr 71
Arg 235
Thr 72
Phe 108
Ile 110
Lys 75
Phe 109
Lys 107

B7	4.50	−12.95	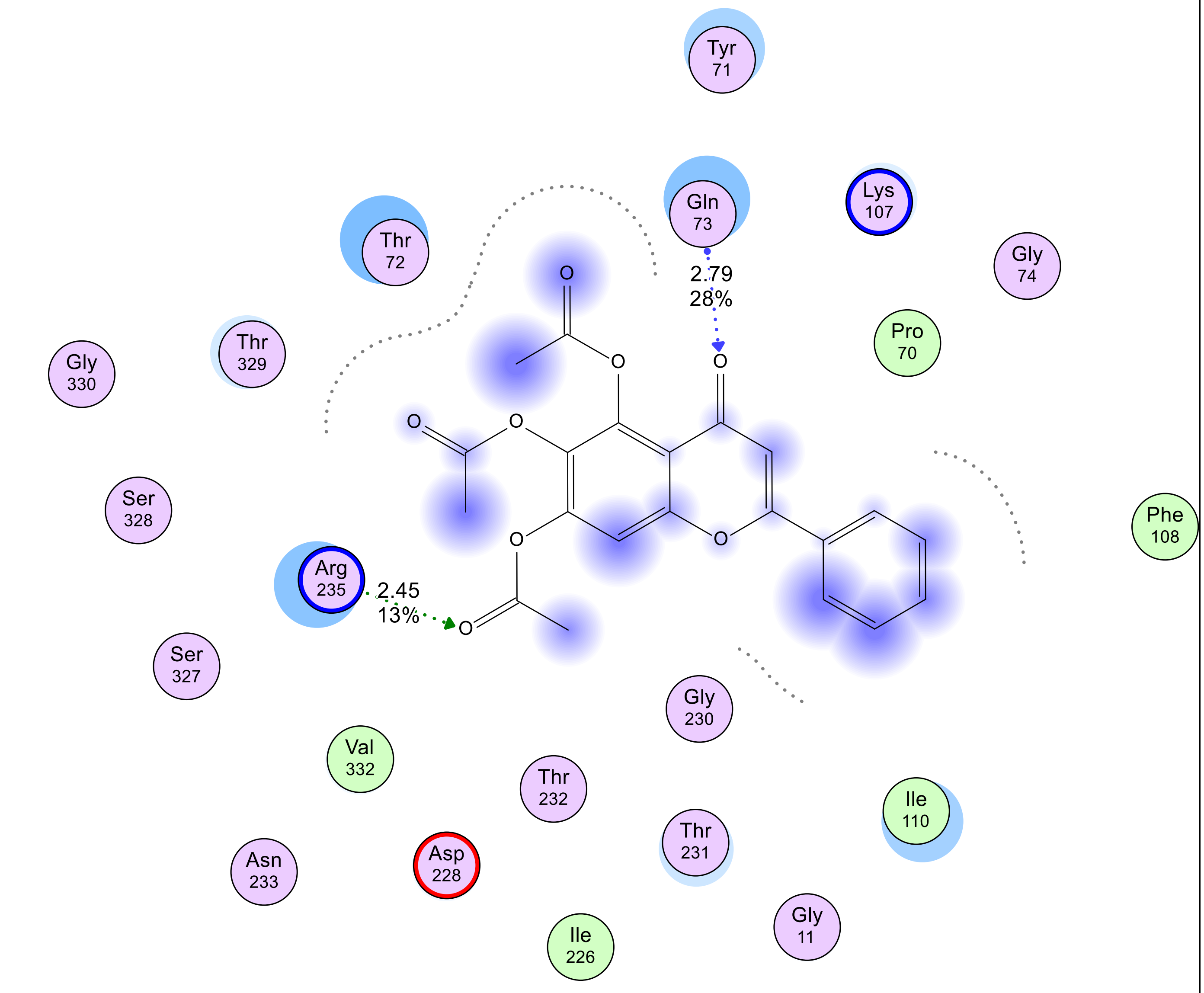Tyr 71<br>Gln 73<br>Lys 107<br>Gly 74<br>Thr 72<br>2.79<br>28%<br>Pro 70<br>Gly 330<br>Thr 329<br>O<br>Ser 328<br>Phe 108<br>Arg 235<br>2.45<br>13%<br>Ser 327<br>Gly 230<br>Val 332<br>Thr 232<br>Ile 110<br>Thr 231<br>Asn 233<br>Asp 228<br>Ile 226<br>Gly 11

B8	4.65	−10.75	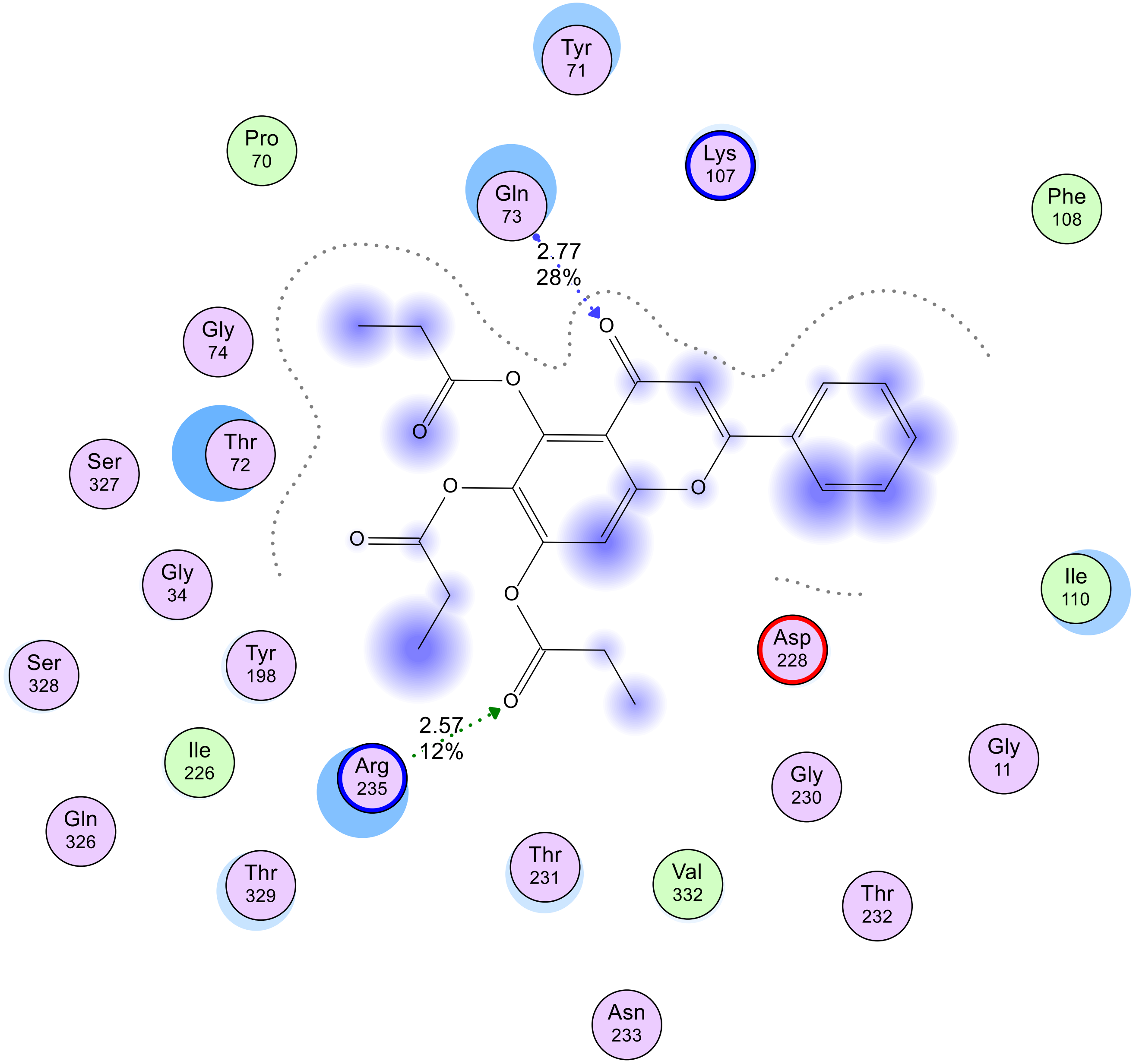

Tyr 71
Pro 70
Gln 73
Lys 107
Phe 108
2.77
28%
Gly 74
Thr 72
Ser 327
Gly 34
Ile 110
Ser 328
Tyr 198
Asp 228
2.57
12%
Ile 226
Arg 235
Gly 11
Gly 230
Gln 326
Thr 329
Thr 231
Val 332
Thr 232
Asn 233
O

D1 (Diosmetin)	4.36	−19.32	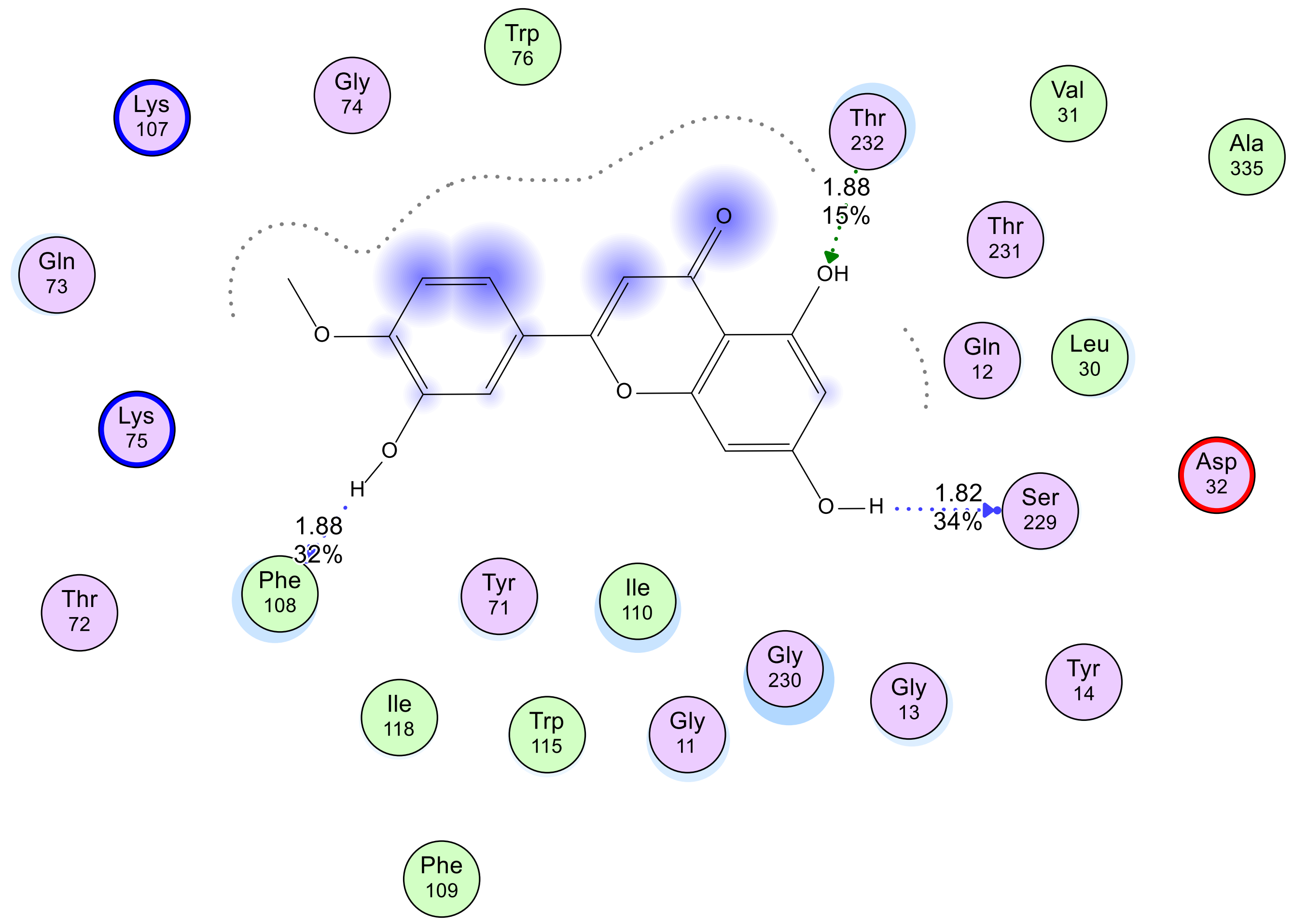

D2	5.67	−19.33	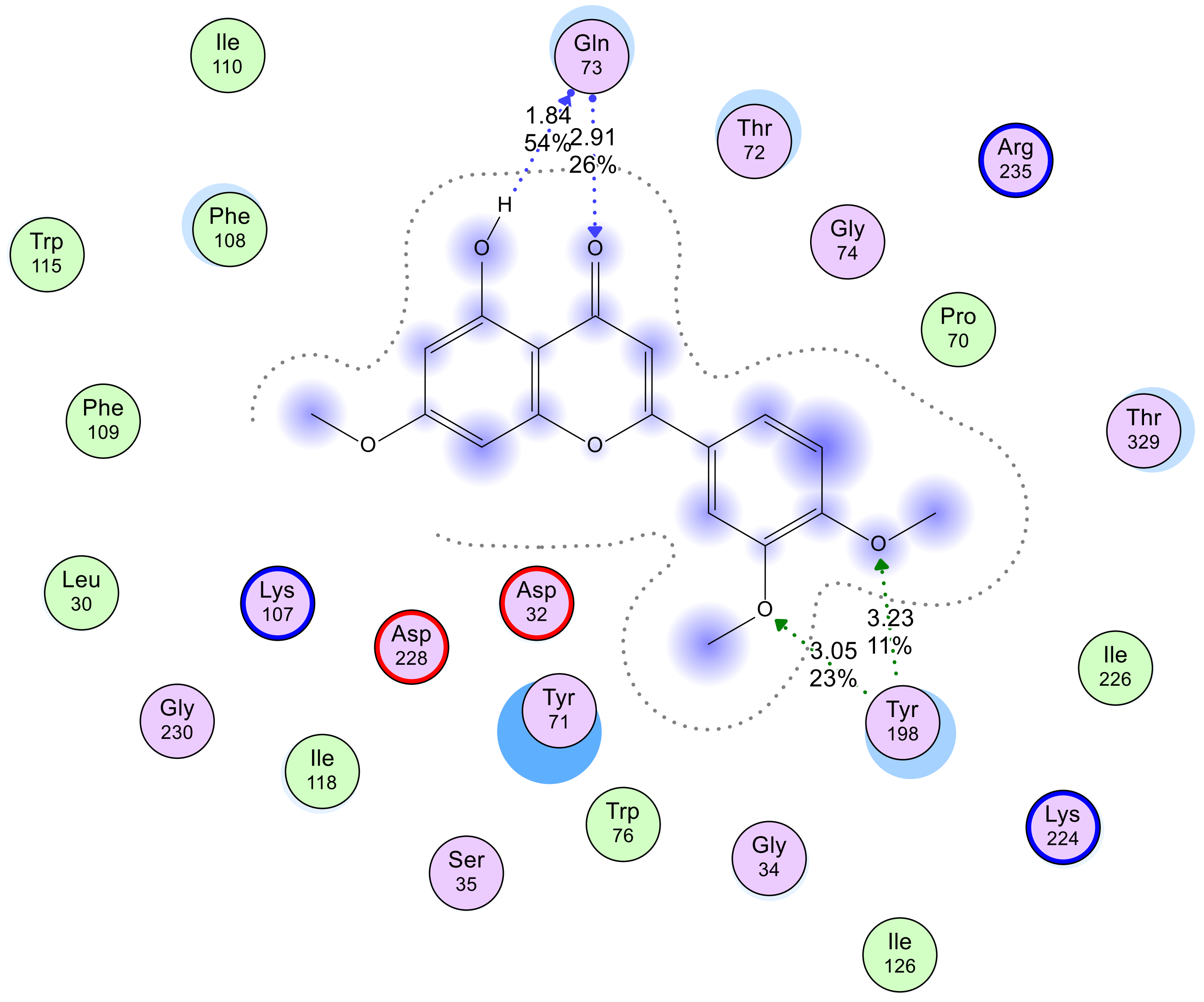

Ile 110
Gln 73
1.84
54%
2.91
26%
Thr 72
Arg 235
Trp 115
Phe 108
H
O
O
Gly 74
Pro 70
Phe 109
O
O
Thr 329
O
Leu 30
Lys 107
Asp 32
Asp 228
O
3.23
11%
3.05
23%
Ile 226
Gly 230
Tyr 71
Tyr 198
Ile 118
Trp 76
Lys 224
Ser 35
Gly 34
Ile 126

D3	5.50	−17.18	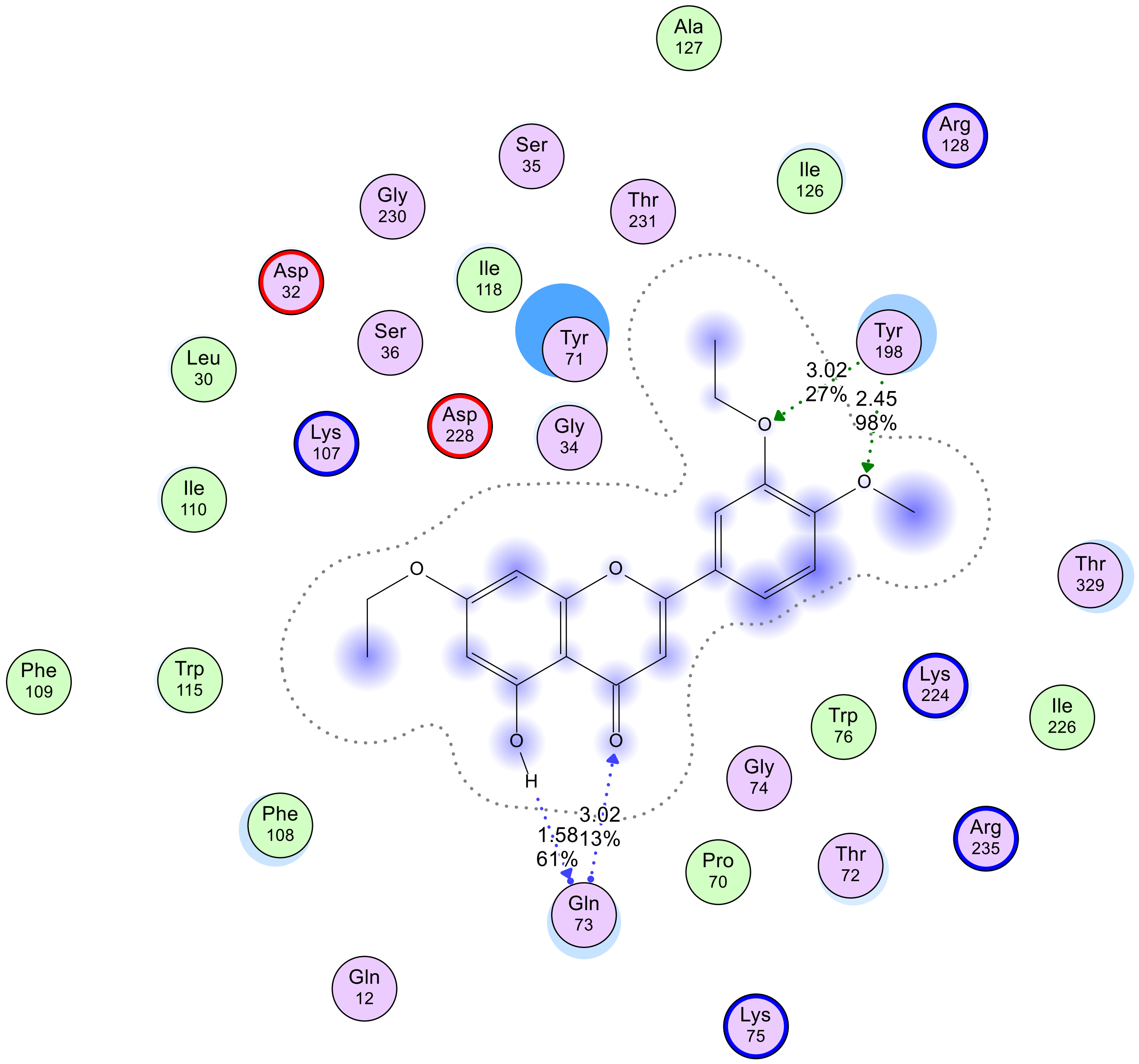

Ala 127
Arg 128
Ser 35
Gly 230
Thr 231
Ile 126
Asp 32
Ile 118
Ser 36
Tyr 71
Tyr 198
Leu 30
Lys 107
Asp 228
Gly 34
3.02 27%
2.45 98%
Ile 110
Thr 329
Phe 109
Trp 115
Lys 224
Ile 226
Trp 76
Gly 74
Phe 108
1.58 61%
3.02 13%
Arg 235
Pro 70
Thr 72
Gln 73
Gln 12
Lys 75

D4	5.44	–13.31	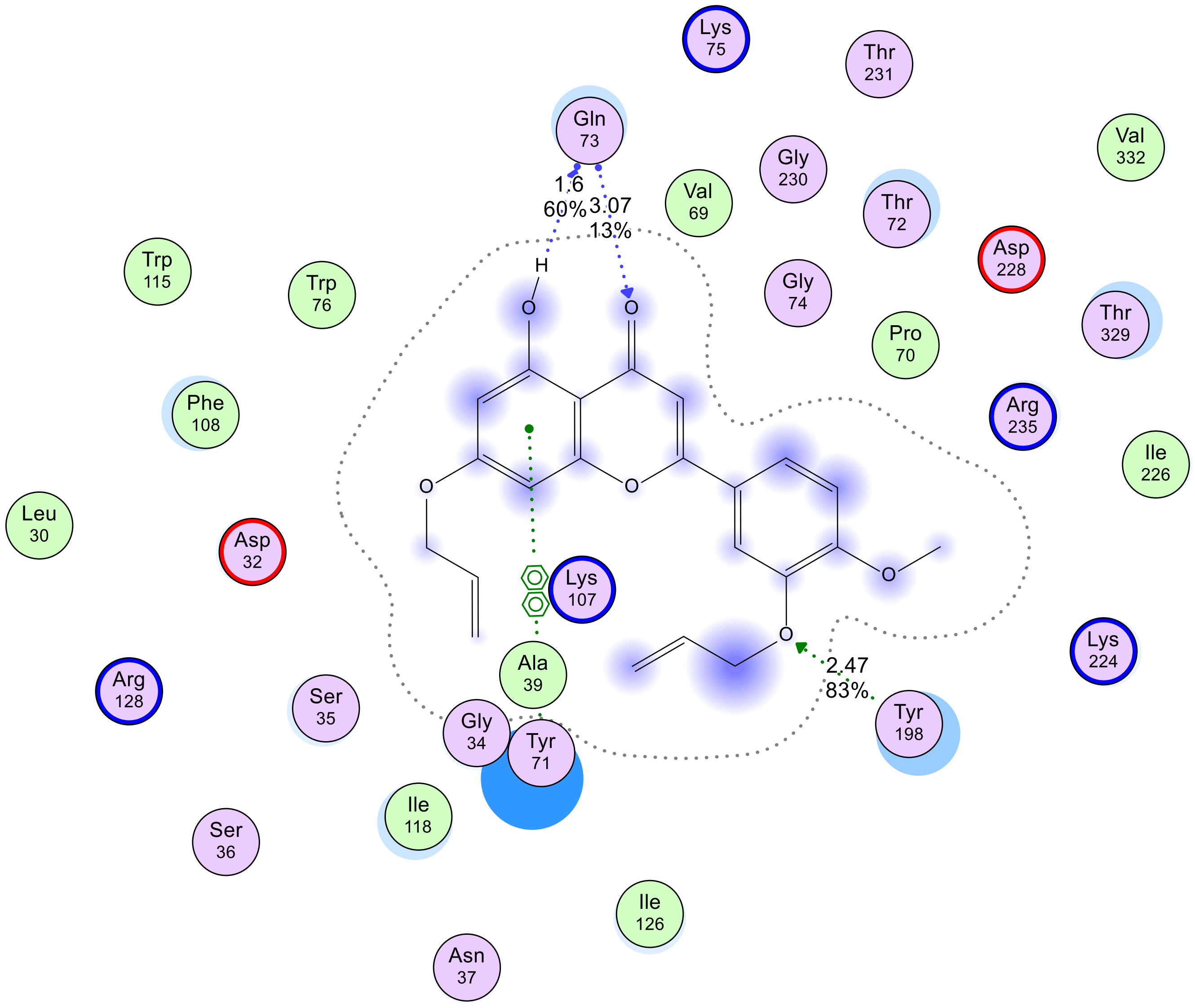

Lys 75
Thr 231
Gln 73
Val 332
Gly 230
1.6 60%
3.07 13%
Val 69
Thr 72
Asp 228
Trp 115
Trp 76
Gly 74
Thr 329
Pro 70
Phe 108
Arg 235
Ile 226
Leu 30
Asp 32
Lys 107
Lys 224
Ala 39
2.47 83%
Arg 128
Ser 35
Gly 34
Tyr 71
Tyr 198
Ile 118
Ser 36
Ile 126
Asn 37

D5	5.78	−13.46	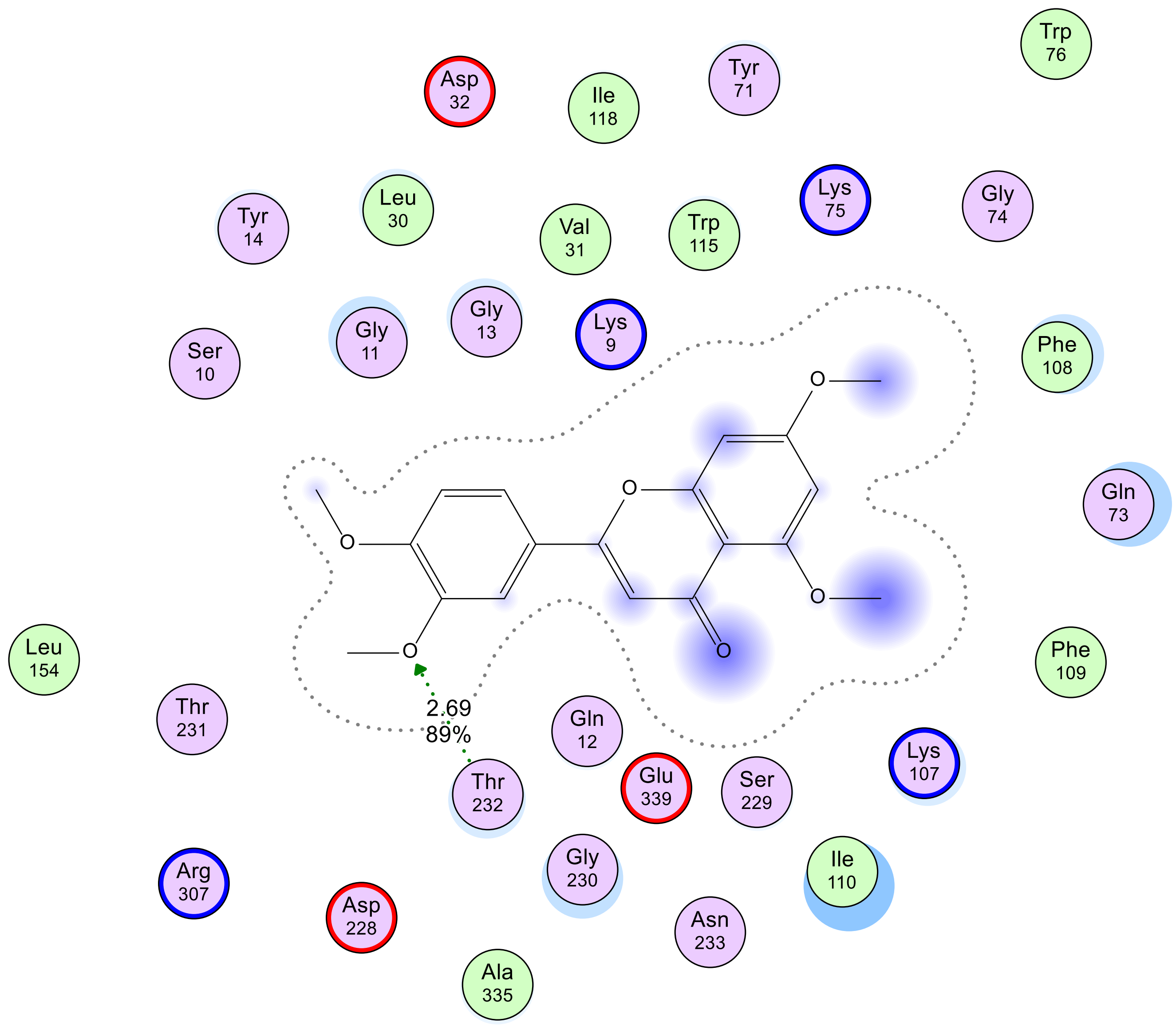Asp 32<br>Ile 118<br>Tyr 71<br>Trp 76<br>Tyr 14<br>Leu 30<br>Val 31<br>Trp 115<br>Lys 75<br>Gly 74<br>Ser 10<br>Gly 11<br>Gly 13<br>Lys 9<br>Phe 108<br>Gln 73<br>Leu 154<br>Phe 109<br>Thr 231<br>2.69<br>89%<br>Gln 12<br>Lys 107<br>Thr 232<br>Glu 339<br>Ser 229<br>Gly 230<br>Ile 110<br>Arg 307<br>Asp 228<br>Asn 233<br>Ala 335

D6	5.80	–12.02	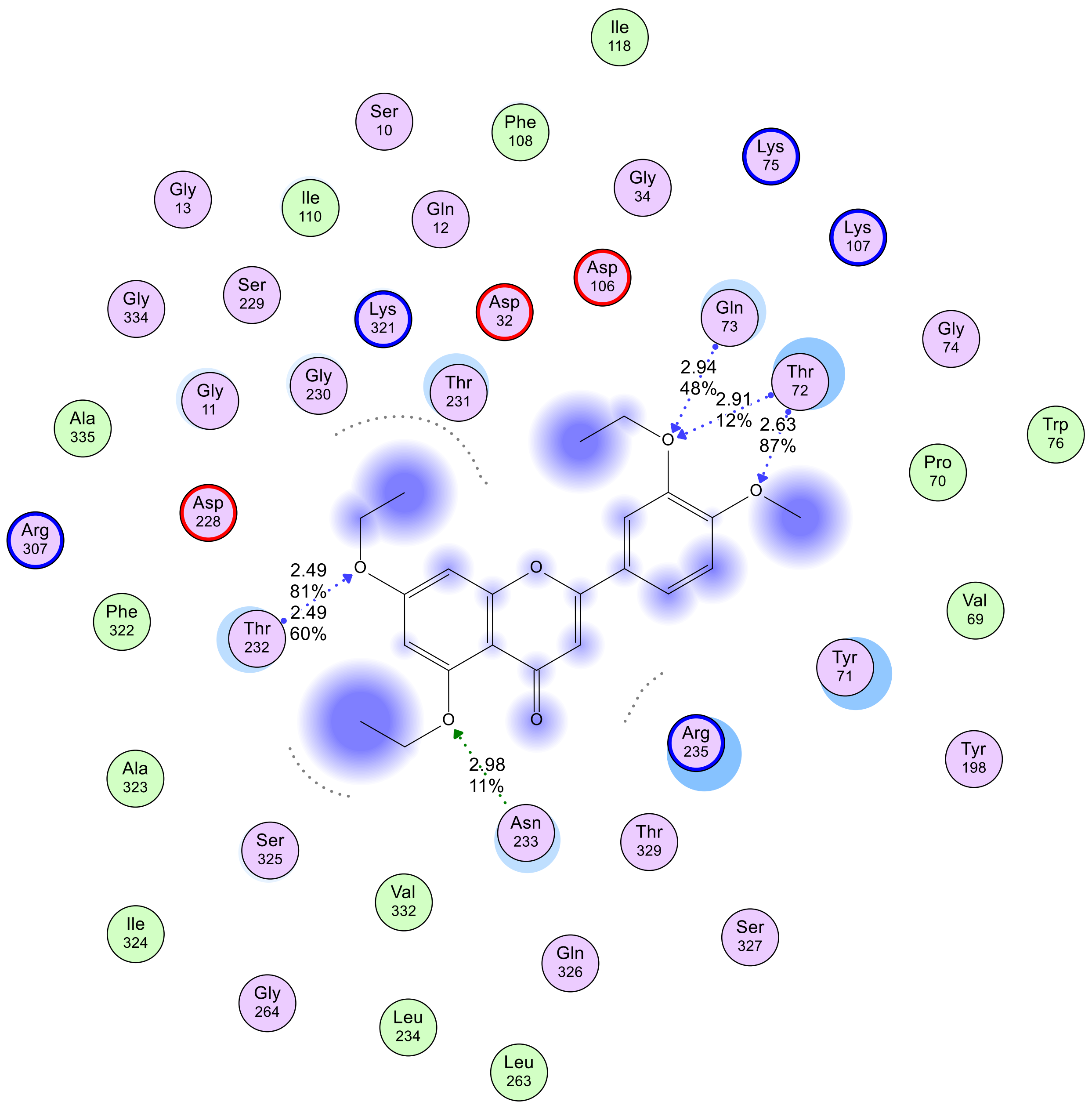

Ile 118
Ser 10
Phe 108
Lys 75
Gly 13
Ile 110
Gln 12
Gly 34
Lys 107
Asp 106
Gly 334
Ser 229
Lys 321
Asp 32
Gln 73
Gly 74
Thr 72
Gly 11
Gly 230
Thr 231
Ala 335
2.94
48%
2.91
12%
2.63
87%
Trp 76
Pro 70
Asp 228
Arg 307
O
O
O
2.49
81%
2.49
60%
Phe 322
Thr 232
Val 69
Tyr 71
O
O
Arg 235
Tyr 198
2.98
11%
Ala 323
Asn 233
Ser 325
Thr 329
Val 332
Ile 324
Ser 327
Gln 326
Gly 264
Leu 234
Leu 263

D7	5.54	−22.07	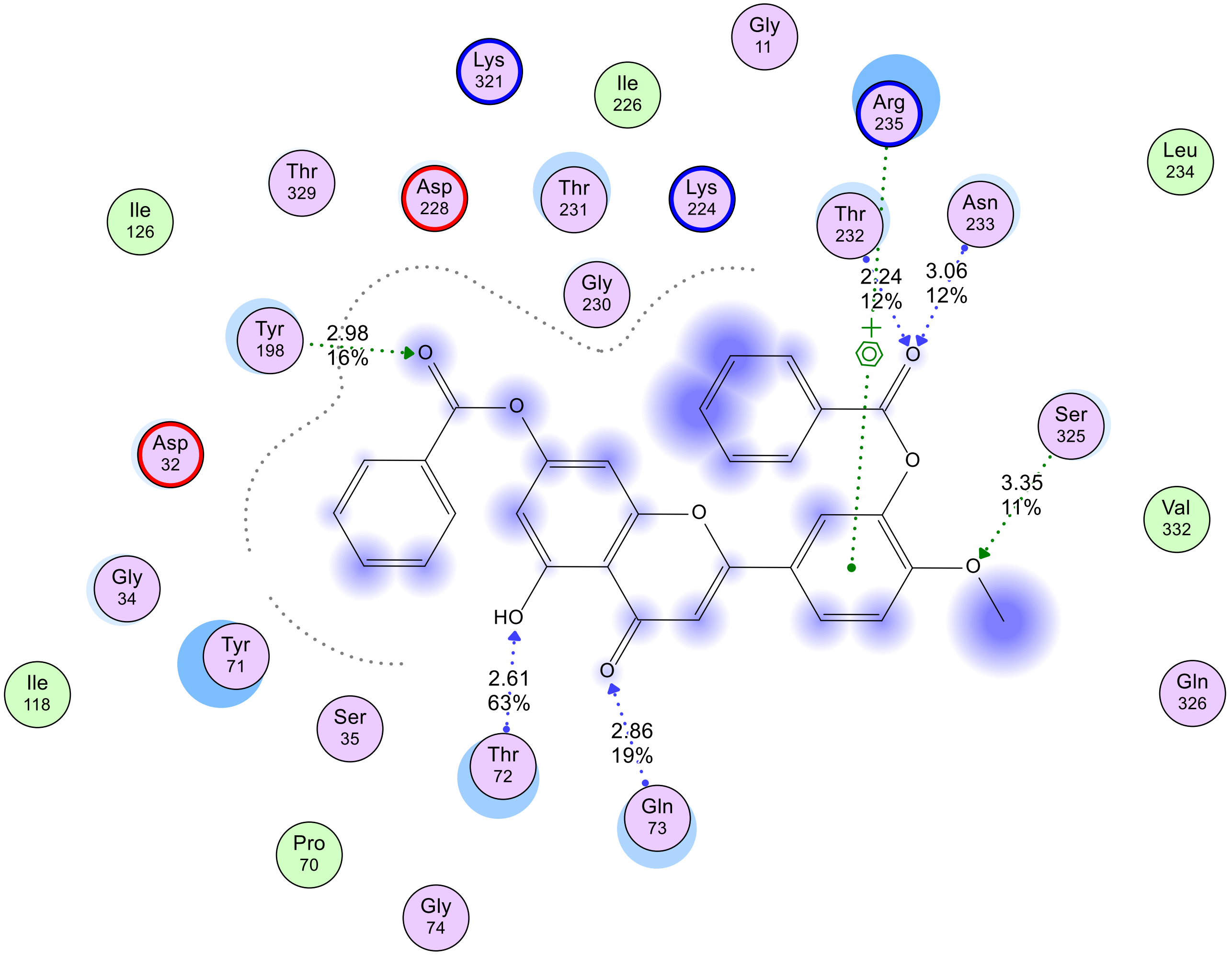

Gly 11
Lys 321
Ile 226
Arg 235
Leu 234
Thr 329
Asp 228
Thr 231
Lys 224
Thr 232
Asn 233
Ile 126
Gly 230
2.24 12%
3.06 12%
Tyr 198
2.98 16%
O
O
O
Asp 32
O
Ser 325
3.35 11%
Val 332
O
O
Gly 34
HO
Tyr 71
O
Ile 118
2.61 63%
Gln 326
Ser 35
2.86 19%
Thr 72
Gln 73
Pro 70
Gly 74

Quercetin	5.02	−22.55	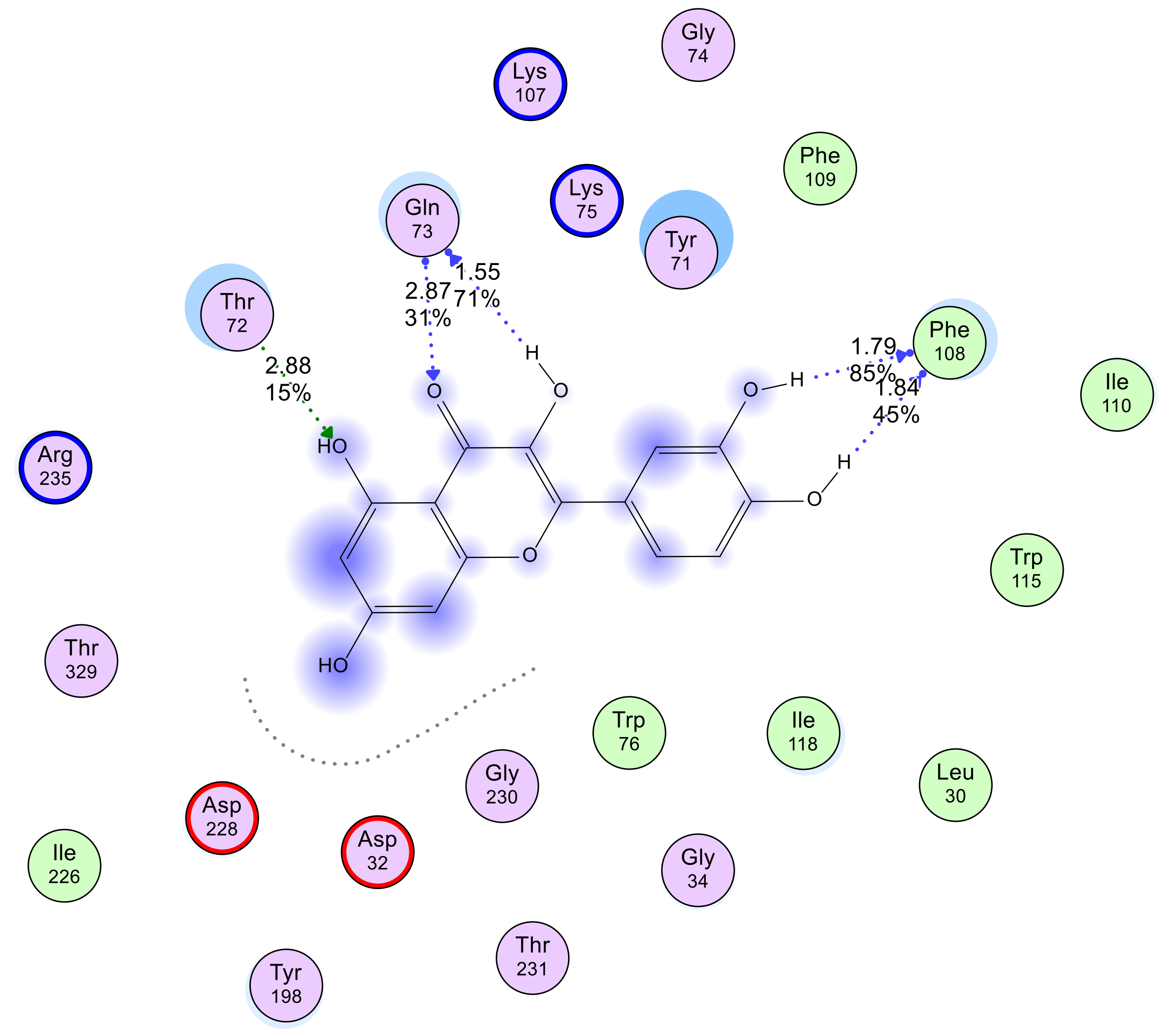

Umibecestat	7.96 [4]	−47.04	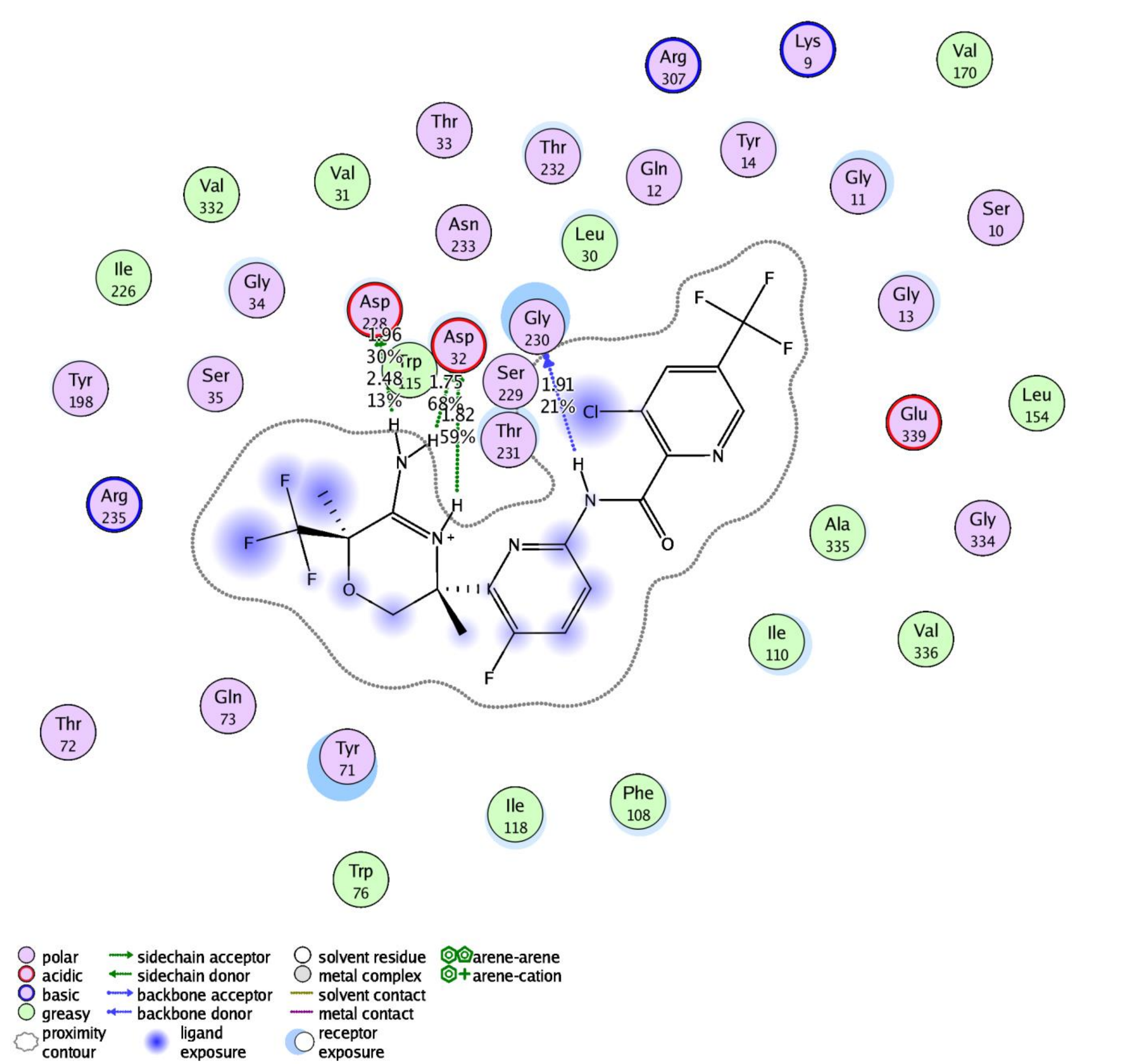

Table S7. Spectra of synthesized flavone derivatives

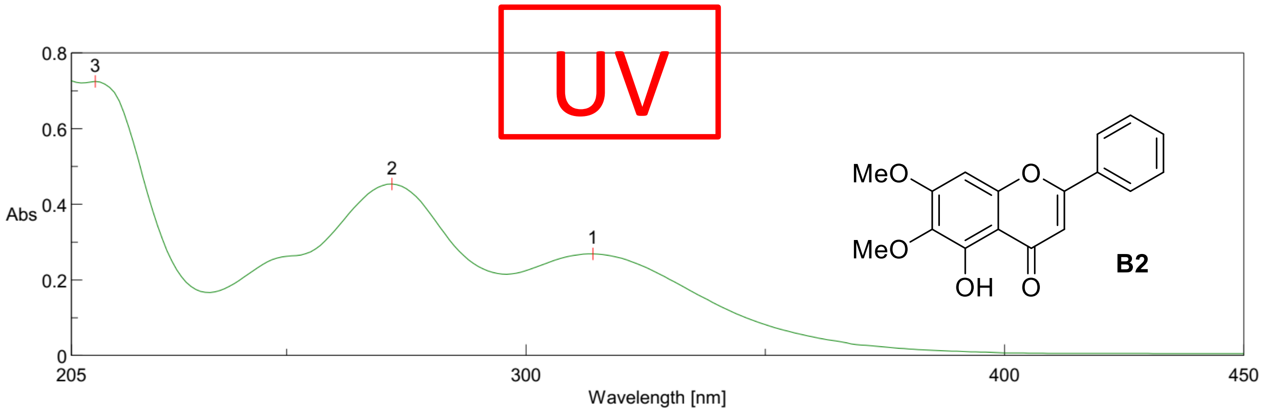

[Result of Peak Picking]

No.	Position	Intensity	No.	Position	Intensity	No.	Position	Intensity
1	314	0.268399	2	272	0.452919	3	210	0.724239

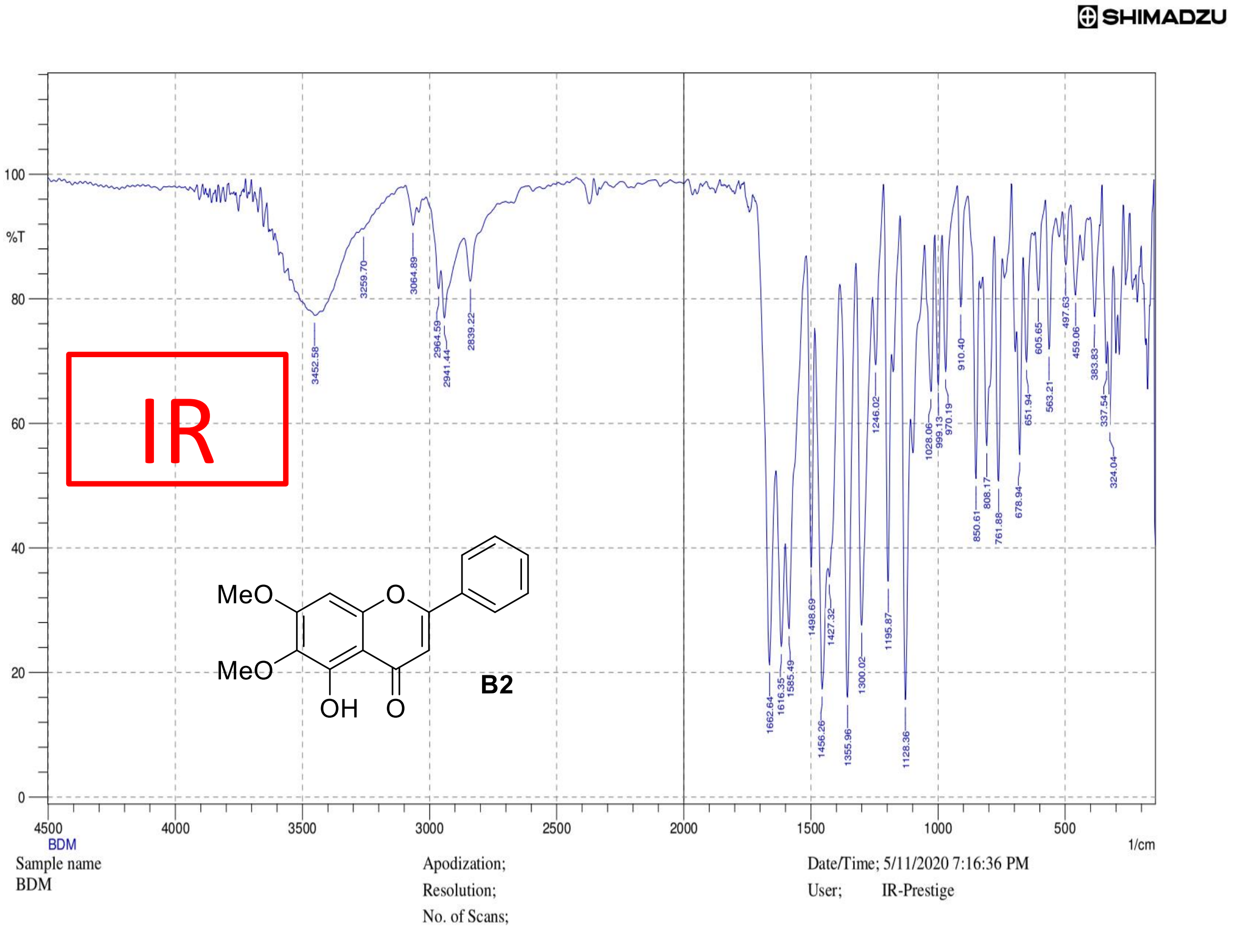

MS

Qualitative Analysis Report

**Data Filename**	BDM 2.d	**Sample Name**	BDM 2
**Sample Type**	Sample	**Position**	P2-C1
**Instrument Name**	Instrument 1	**User Name**	
**Acq Method**	Cot ngan - MSMS_Pos.m	**Acquired Time**	04/08/2020 8:02:09 PM
**IRM Calibration Status**	Success	**DA Method**	COTNGAN.M.m
**Comment**			

**Sample Group**		**Info.**	
**Stream Name**	LC 1	**Acquisition SW Version**	6200 series TOF/6500 series Q-TOF B.06.01 (B6172 SP1)

User Chromatograms

Fragmentor Voltage 140 **Collision Energy** 0 **Ionization Mode** ESI

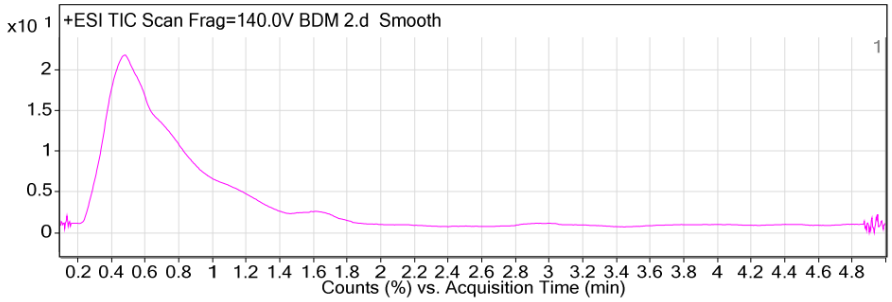

User Spectra

Fragmentor Voltage	Collision Energy	Ionization Mode
140	0	ESI

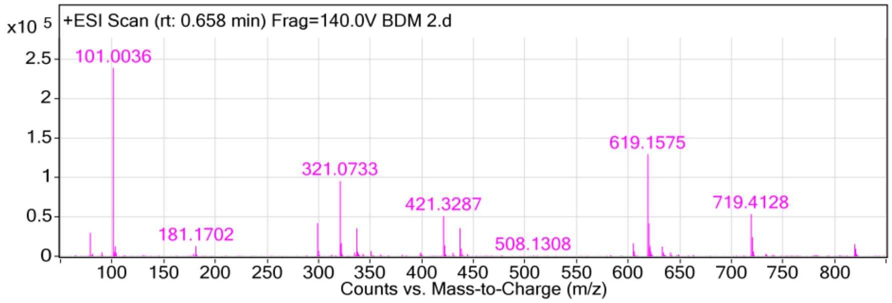

Peak List

m/z	z	Abund
79.0213	1	29725.18
101.0036	1	239362.81
299.0917	1	41948.91
321.0733	1	95620.17
337.0475	1	35516.83
421.3287	1	51010.1
437.3026	1	35858.05
619.1575	1	129711.47
620.1605	1	41771.26
719.4128	1	53729.01

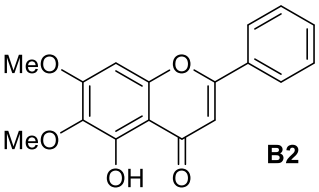

B2

--- End Of Report ---

MS

**Sample Name**	BDM 2	**Position**	P2-C1	**Instrument Name**	Instrument 1	**User Name**	
**Inj Vol**	2	**InjPosition**		**SampleType**	Sample	**IRM Calibration Status**	Success
**Data Filename**	BDM 2.d	**ACQ Method**	Cot ngan - MSMS_Pos.	**Comment**		**Acquired Time**	04/08/2020 8:02:09 PM

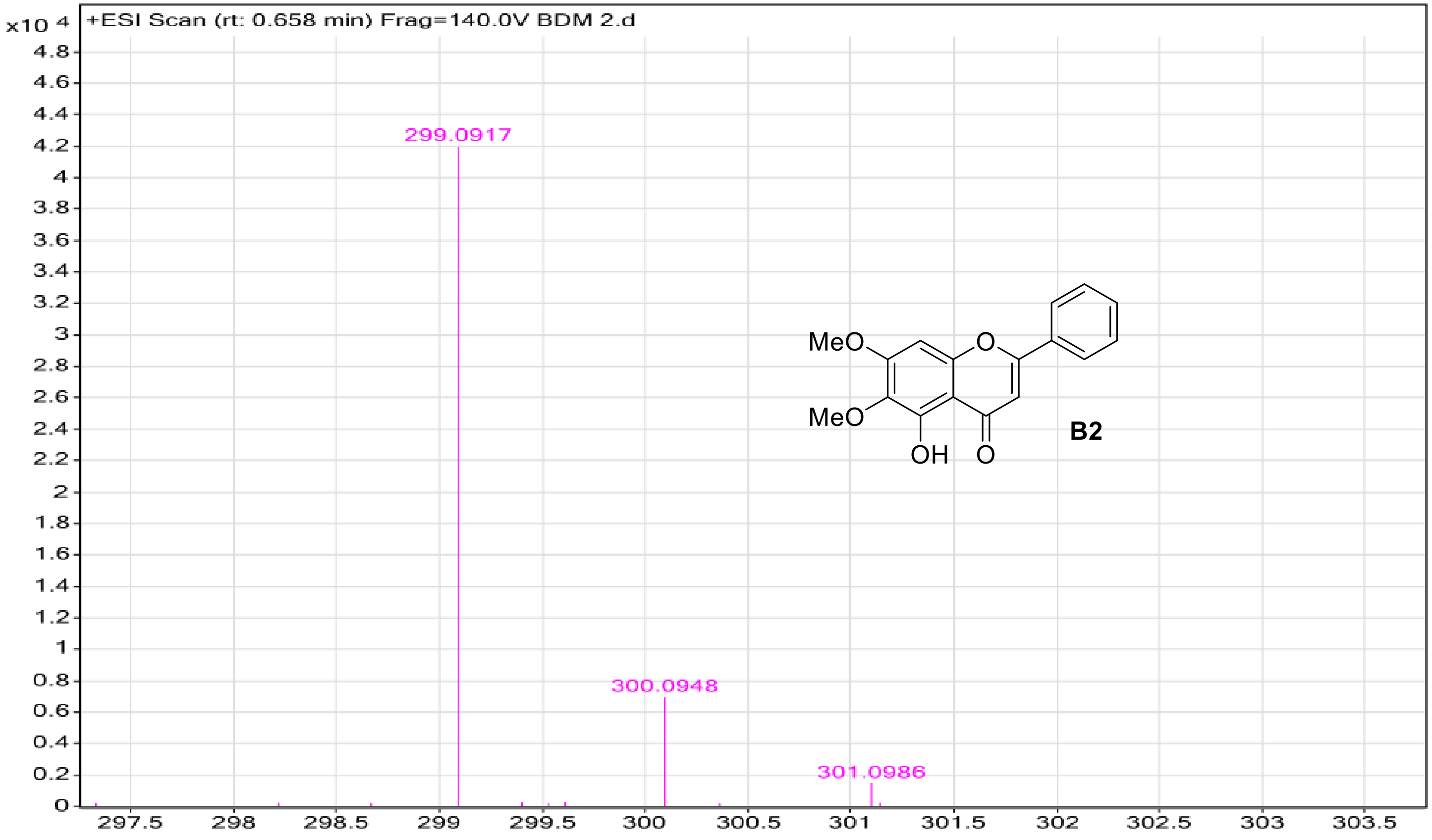

^{1}H-NMR

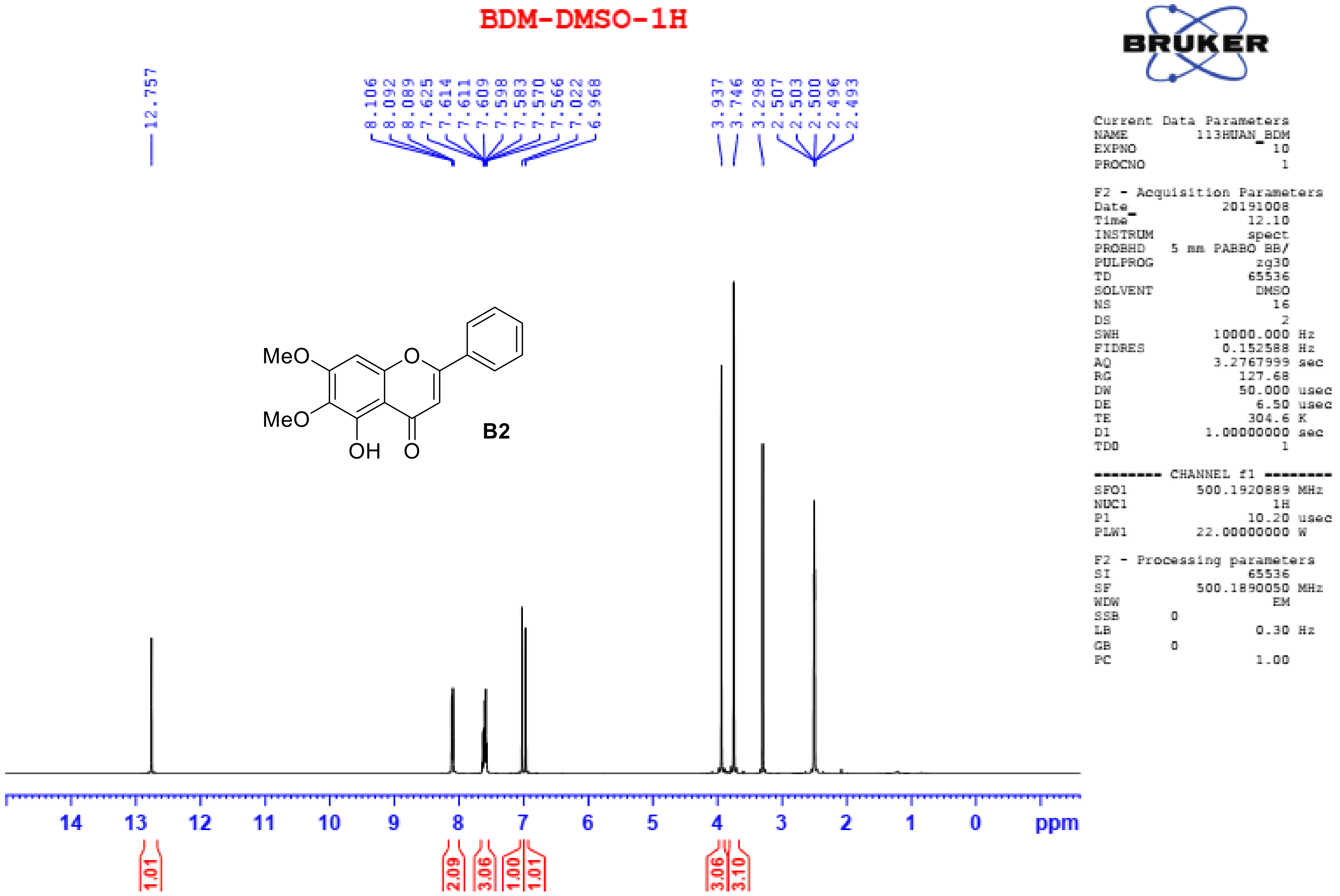

BDM-DMSO-1H
12.757
8.106 8.092 8.089 7.625 7.614 7.611 7.609 7.598 7.583 7.570 7.566 7.022 6.968
3.937 3.746 3.298 2.507 2.503 2.500 2.496 2.493
MeO
MeO
O
OH
O
B2
BRUKER
Current Data Parameters
NAME 113HUAN_BDM
EXPNO 10
PROCNO 1
F2 - Acquisition Parameters
Date_ 20191008
Time 12.10
INSTRUM spect
PROBHD 5 mm PABBO BB/
PULPROG zg30
TD 65536
SOLVENT DMSO
NS 16
DS 2
SWH 10000.000 Hz
FIDRES 0.152588 Hz
AQ 3.2767999 sec
RG 127.68
DW 50.000 usec
DE 6.50 usec
TE 304.6 K
D1 1.00000000 sec
TD0 1
======== CHANNEL f1 ========
SFO1 500.1920889 MHz
NUC1 1H
P1 10.20 usec
PLW1 22.00000000 W
F2 - Processing parameters
SI 65536
SF 500.1890050 MHz
WDW EM
SSB 0
LB 0.30 Hz
GB 0
PC 1.00
14 13 12 11 10 9 8 7 6 5 4 3 2 1 0 ppm
1.01 2.09 3.06 1.00 1.01 3.06 3.10

^{1}H-NMR

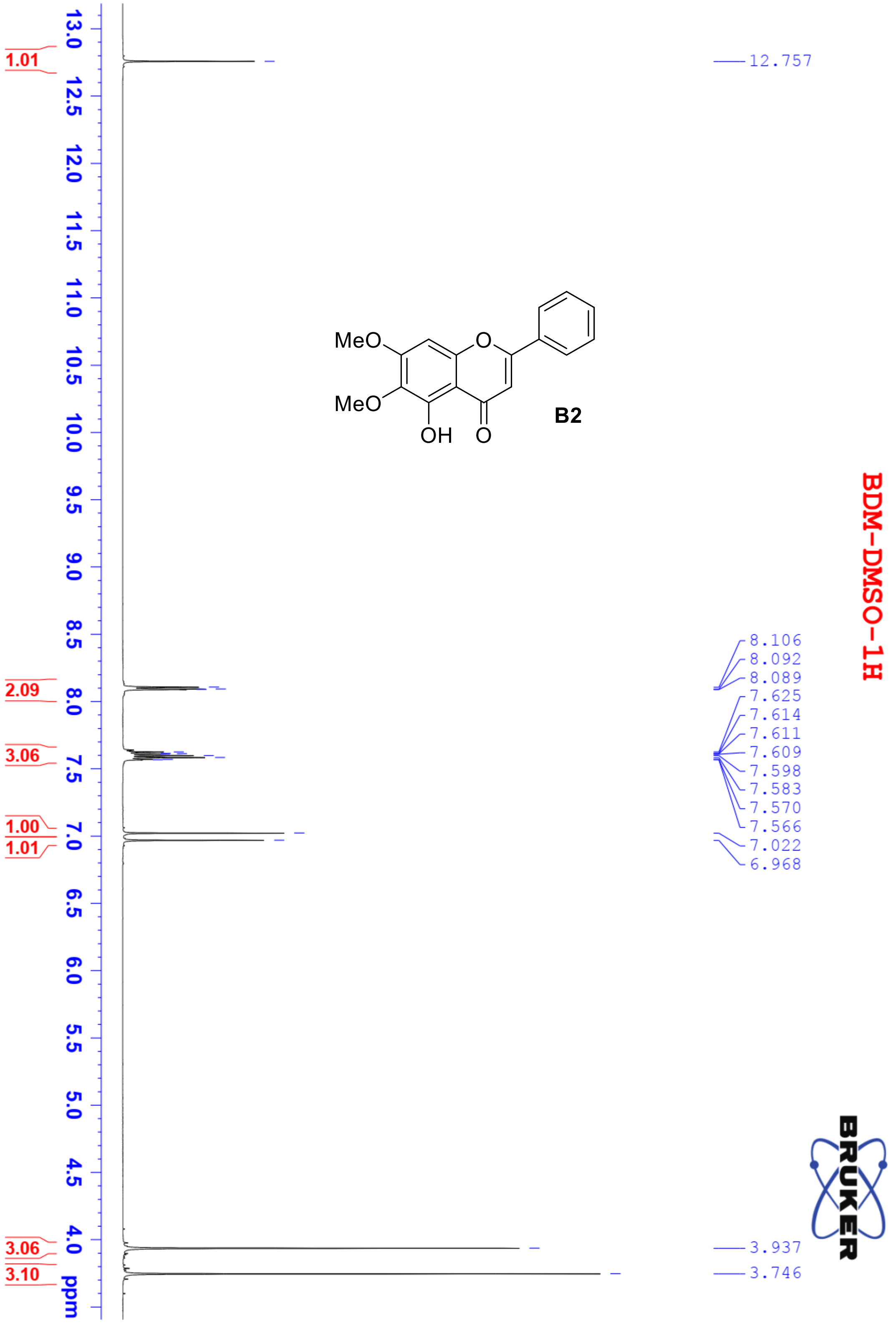

MeO
MeO
OH
O
O
B2
BDM-DMSO-1H
12.757
8.106
8.092
8.089
7.625
7.614
7.611
7.609
7.598
7.583
7.570
7.566
7.022
6.968
3.937
3.746
1.01
2.09
3.06
1.00
1.01
3.06
3.10
ppm

^{1}H-NMR

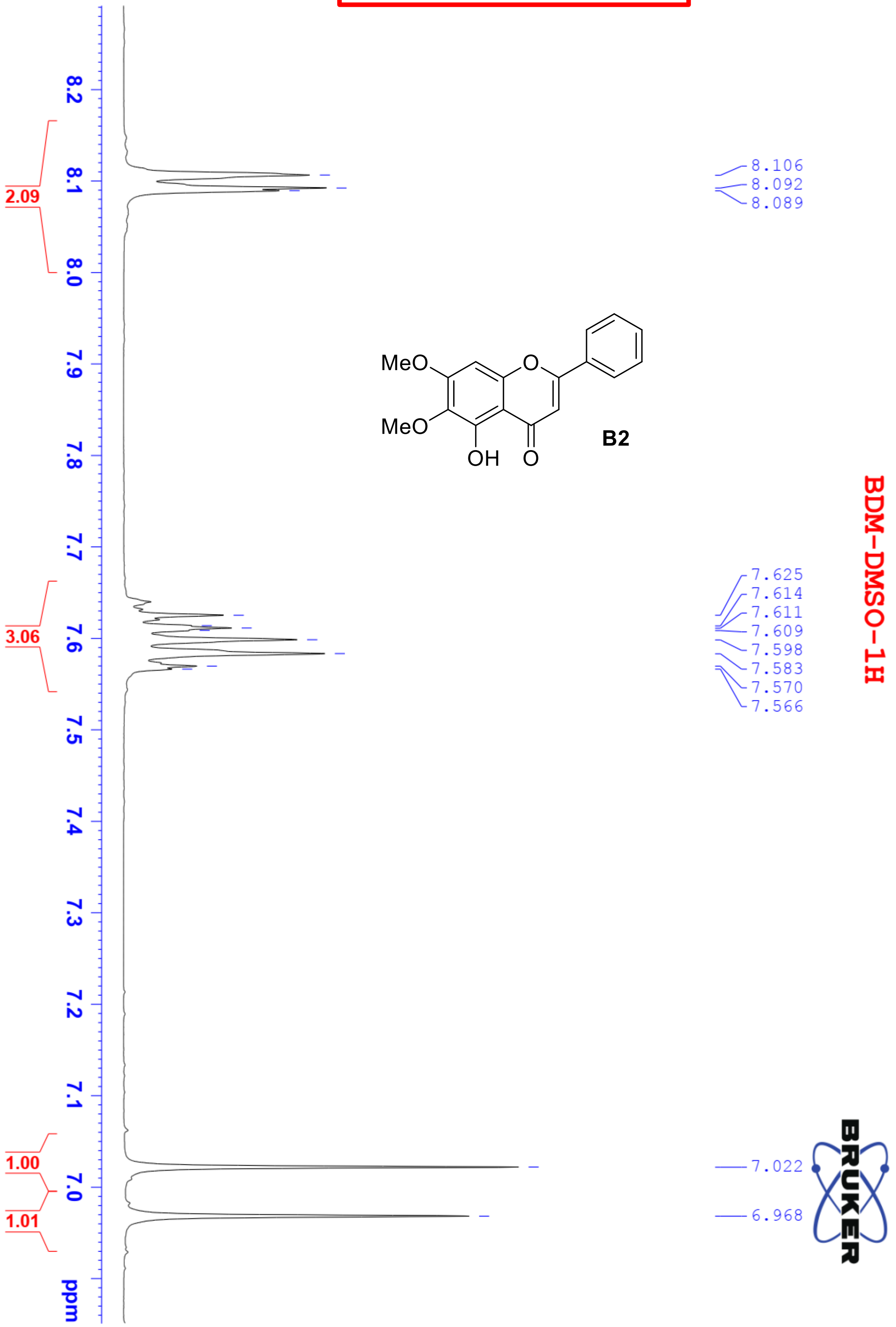

MeO
MeO
OH
O
O
B2
8.106
8.092
8.089
7.625
7.614
7.611
7.609
7.598
7.583
7.570
7.566
7.022
6.968
BDM-DMSO-1H
2.09
3.06
1.00
1.01
8.2
8.1
8.0
7.9
7.8
7.7
7.6
7.5
7.4
7.3
7.2
7.1
7.0
ppm

^{13}C-NMR

BDM-DMSO-C13CPD

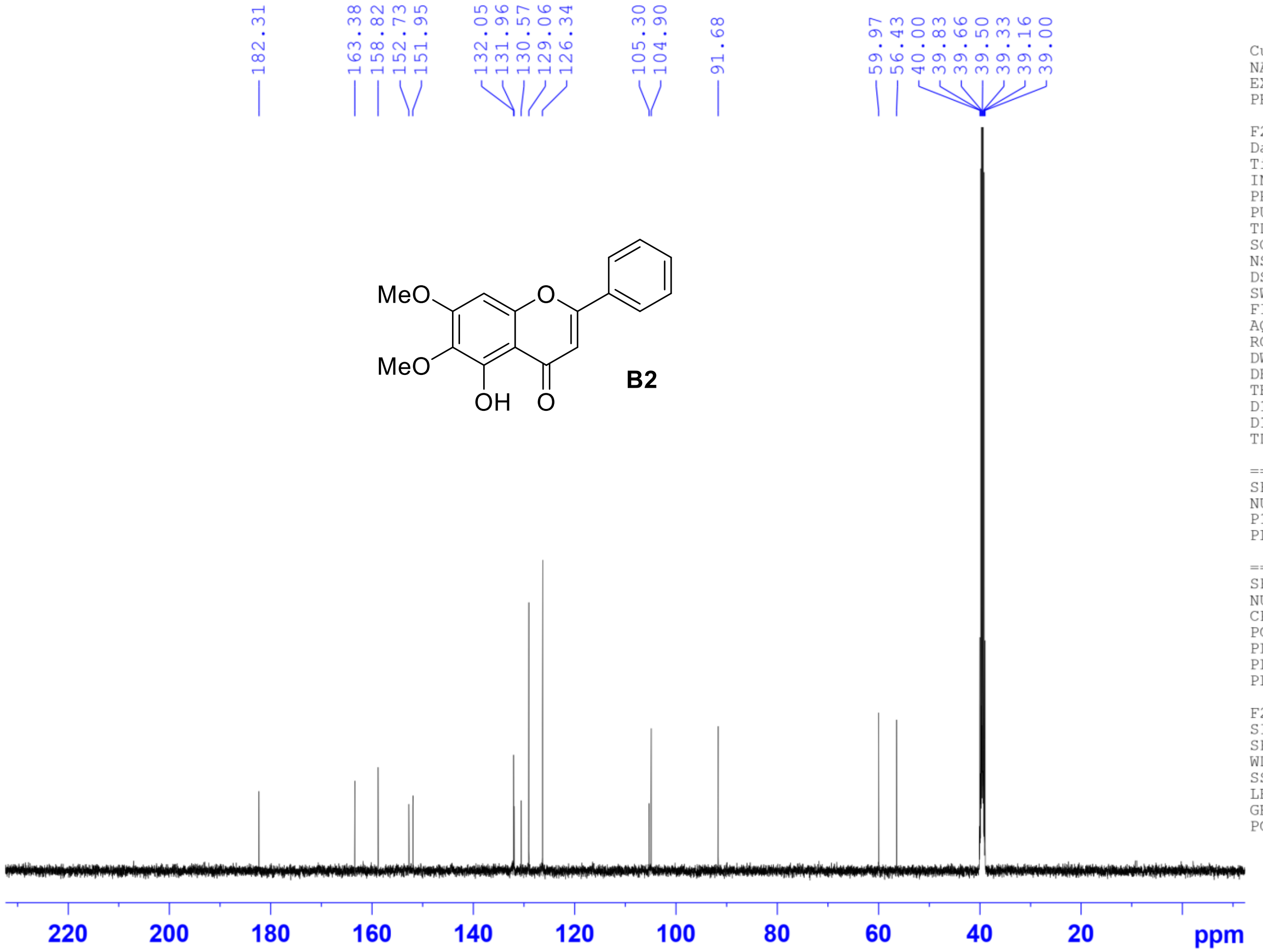


```
Current Data Parameters
NAME              113HUAN_BDM
EXPNO                       2
PROCNO                      1

F2 - Acquisition Parameters
Date_                20191008
Time                    16.40
INSTRUM                 spect
PROBHD      5 mm PABBO BB/
PULPROG               zgpg30
TD                      65536
SOLVENT                  DMSO
NS                        256
DS                          4
SWH                 31250.000 Hz
FIDRES               0.476837 Hz
AQ                  1.0485760 sec
RG                     198.57
DW                     16.000 usec
DE                       6.50 usec
TE                      304.1 K
D1                 2.00000000 sec
D11                0.03000000 sec
TD0                         1

======== CHANNEL f1 ========
SFO1             125.7864591 MHz
NUC1                      13C
P1                      10.00 usec
PLW1              88.00000000 W

======== CHANNEL f2 ========
SFO2             500.1910008 MHz
NUC2                       1H
CPDPRG[2              waltz16
PCPD2                   80.00 usec
PLW2              22.00000000 W
PLW12              0.35764000 W
PLW13              0.17989001 W

F2 - Processing parameters
SI                      32768
SF               125.7726928 MHz
WDW                        EM
SSB      0
LB                       1.00 Hz
GB       0
PC                       1.40
```

^{13}C-NMR

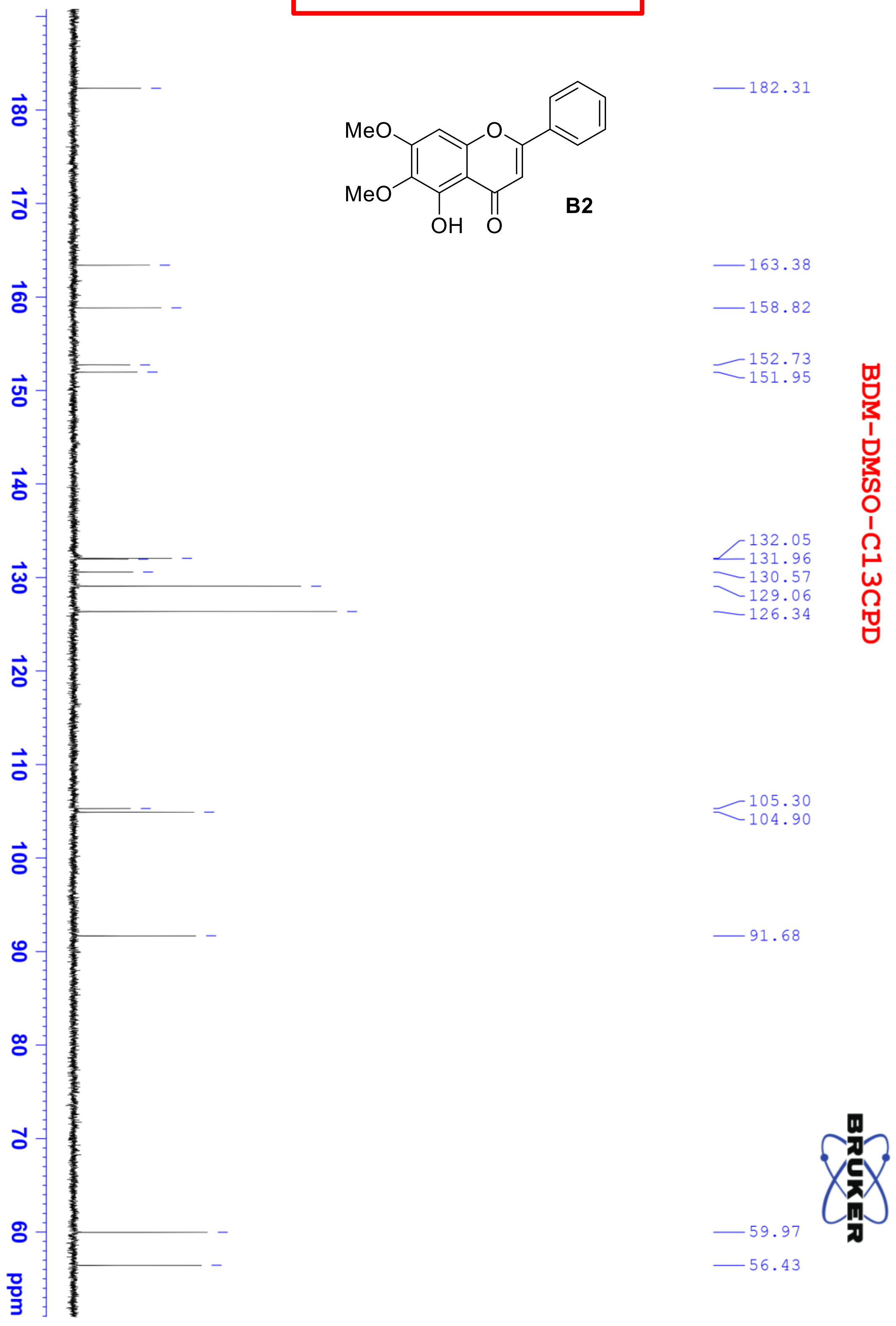

MeO
MeO
OH
O
O
B2
182.31
163.38
158.82
152.73
151.95
132.05
131.96
130.57
129.06
126.34
105.30
104.90
91.68
59.97
56.43
BDM-DMSO-C13CPD
180
170
160
150
140
130
120
110
100
90
80
70
60
ppm

^{13}C-NMR

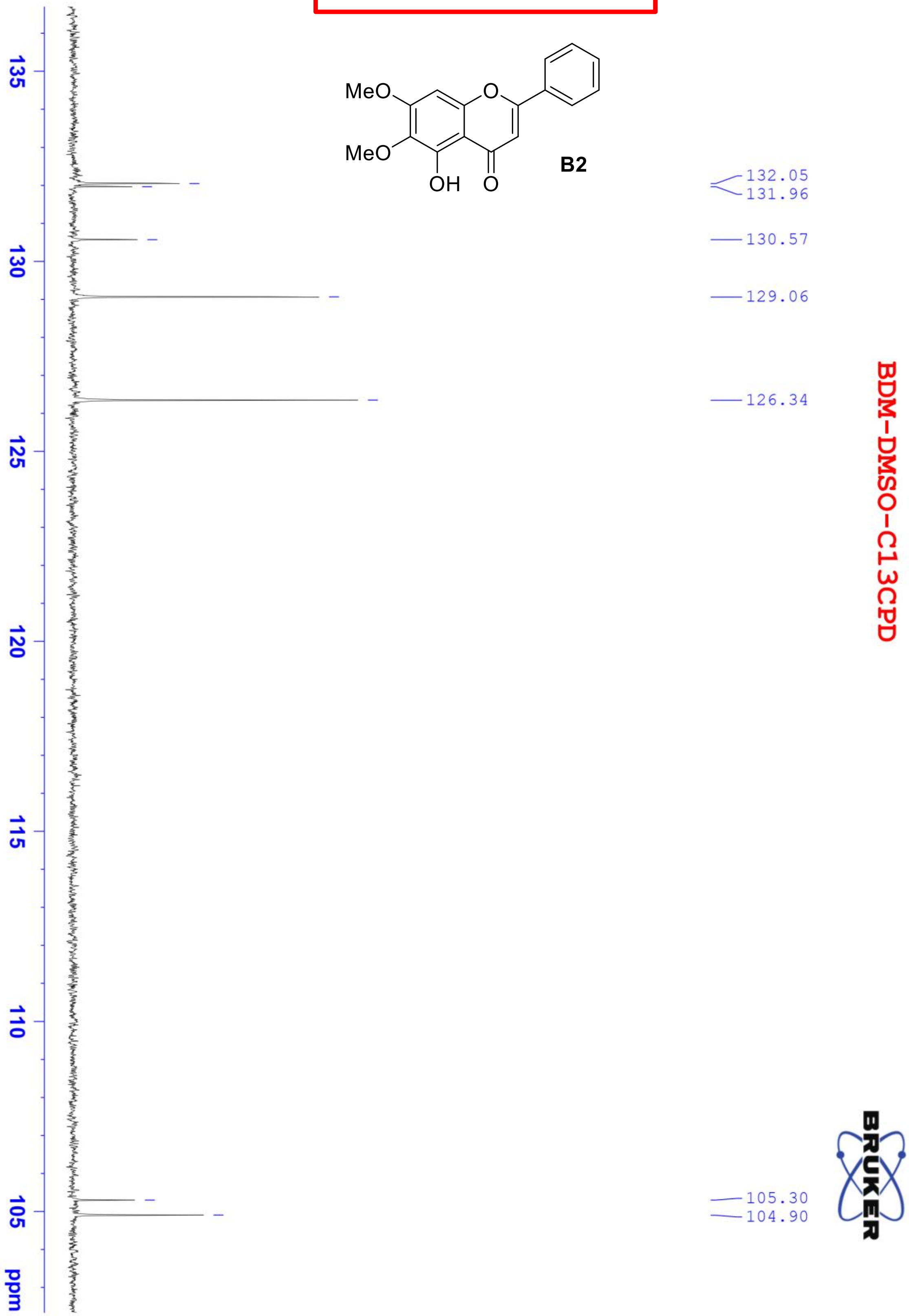
MeO
MeO
OH
O
O
B2
132.05
131.96
130.57
129.06
126.34
105.30
104.90
135
130
125
120
115
110
105
ppm
BDM-DMSO-C13CPD

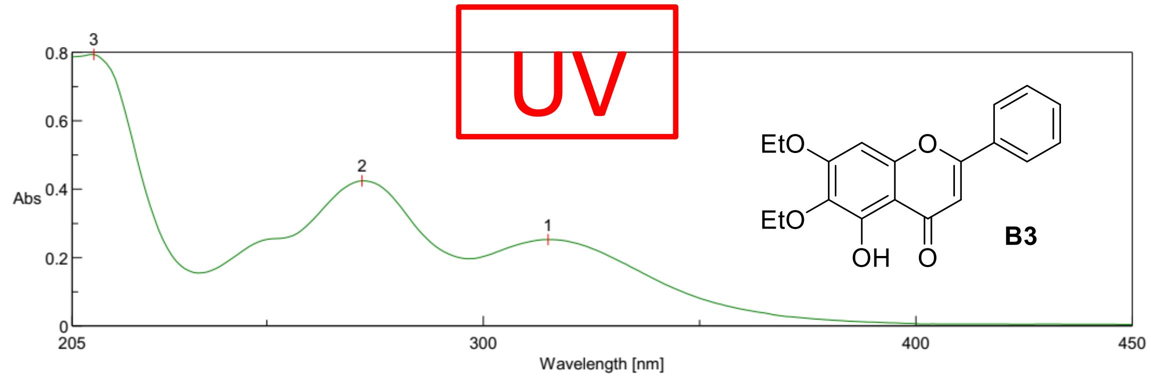

[Result of Peak Picking]

No.	Position	Intensity	No.	Position	Intensity	No.	Position	Intensity
1	315	0.252366	2	272	0.424071	3	210	0.793522

SHIMADZU

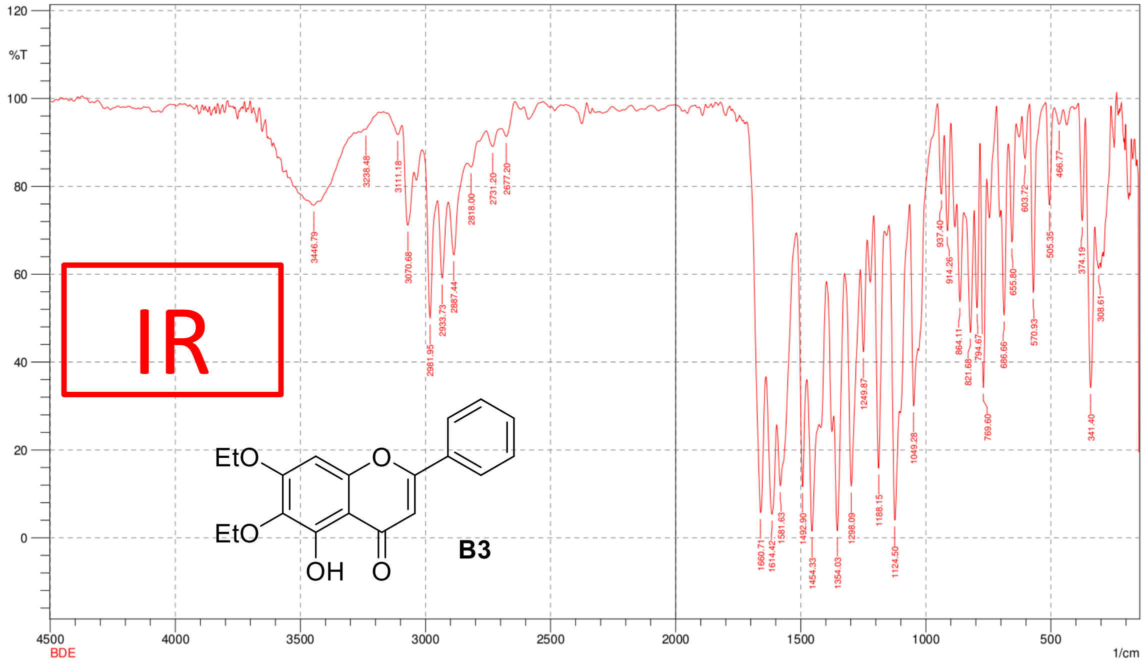

BDE
Sample name
BDE

Apodization;
Resolution;
No. of Scans;

Date/Time; 5/11/2020 5:52:21 PM
User; IR-Prestige

MS

Qualitative Analysis Report

**Data Filename**	BDE 4.d	**Sample Name**	BDE 4
**Sample Type**	Sample	**Position**	P2-B2
**Instrument Name**	Instrument 1	**User Name**	
**Acq Method**	Cot ngan - MSMS_Pos.m	**Acquired Time**	04/08/2020 7:17:38 PM
**IRM Calibration Status**	Success	**DA Method**	COTNGAN.M.m
**Comment**			

**Sample Group**		**Info.**	
**Stream Name**	LC 1	**Acquisition SW Version**	6200 series TOF/6500 series Q-TOF B.06.01 (B6172 SP1)

User Chromatograms

Fragmentor Voltage 140 **Collision Energy** 0 **Ionization Mode** ESI

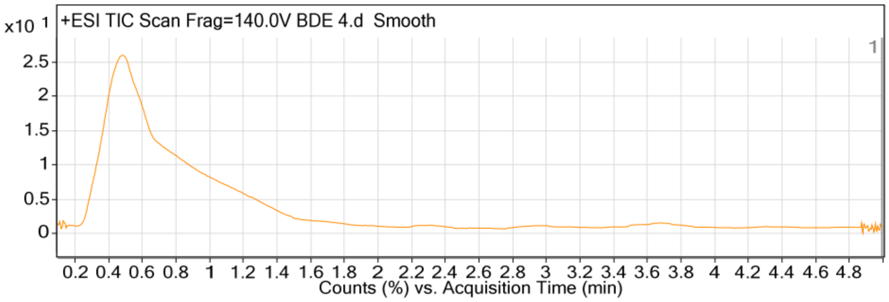

User Spectra

Fragmentor Voltage 140 **Collision Energy** 0 **Ionization Mode** ESI

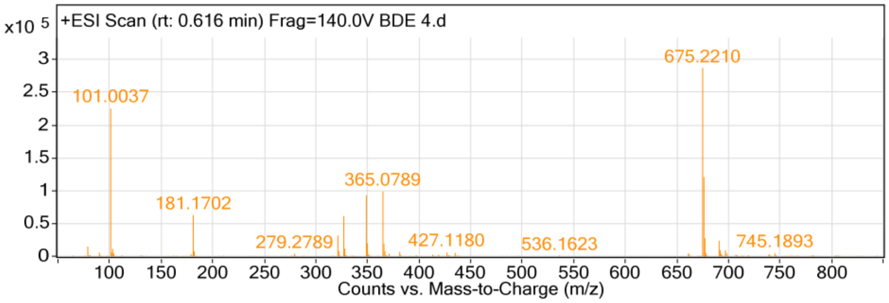

Peak List

m/z	z	Abund
101.0037	1	225364.16
181.1702	1	62704.9
321.3268	1	31494.73
327.123	1	61334.02
349.105	1	92764.2
365.0789	1	98738.83
675.221	1	287090.03
676.2238	1	120780.58
677.2262	1	27656.04
691.1934	1	23767.92

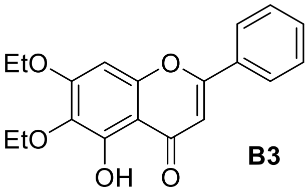

B3

--- End Of Report ---

MS

Sample Name	BDE 4	Position	P2-B2	Instrument Name	Instrument 1	User Name	
Inj Vol	2	InjPosition		SampleType	Sample	IRM Calibration Status	Success
Data Filename	BDE 4.d	ACQ Method	Cot ngan - MSMS_Pos.	Comment		Acquired Time	04/08/2020 7:17:38 PM

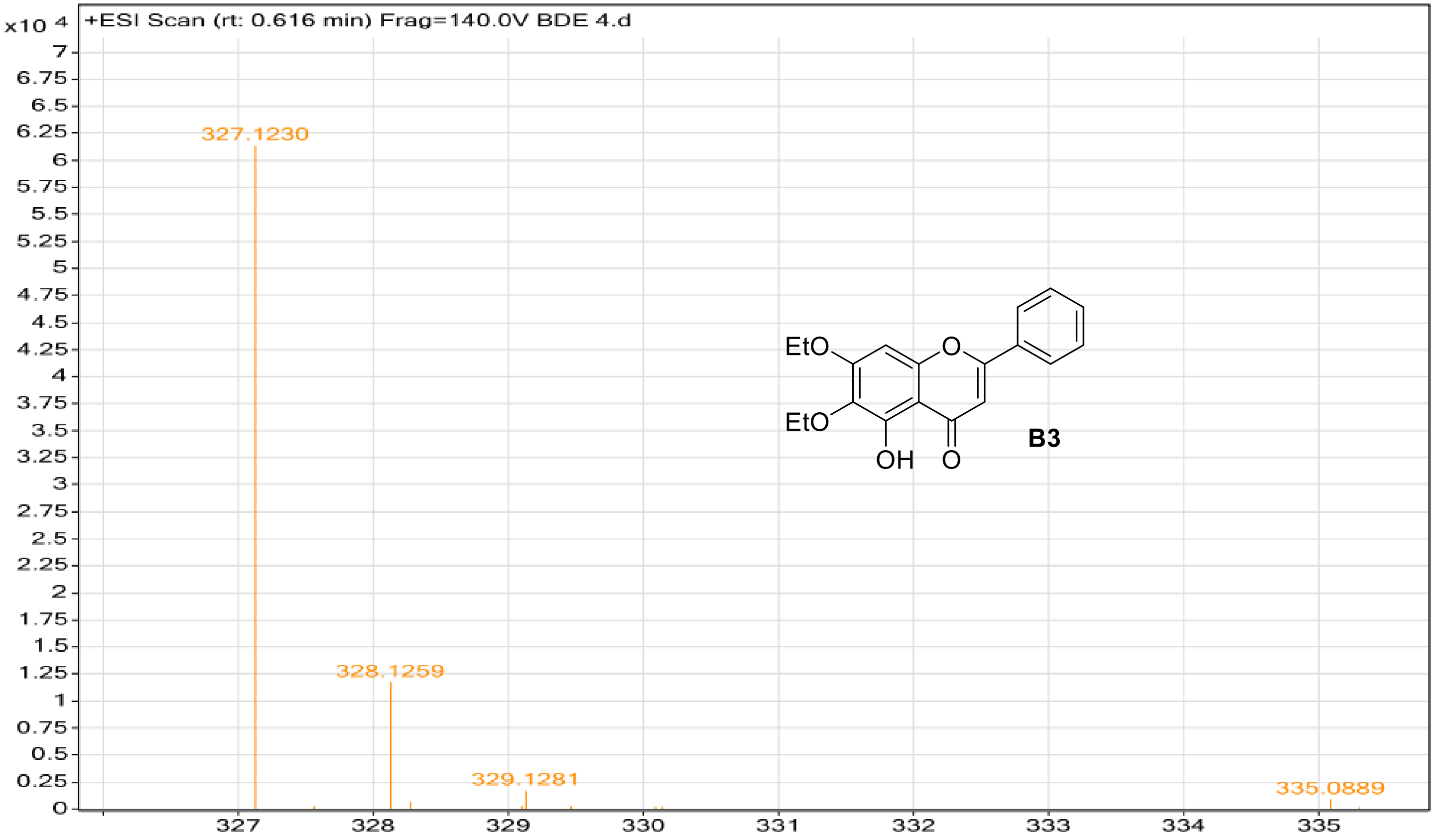

^{1}H-NMR

BDE-DMSO-1H

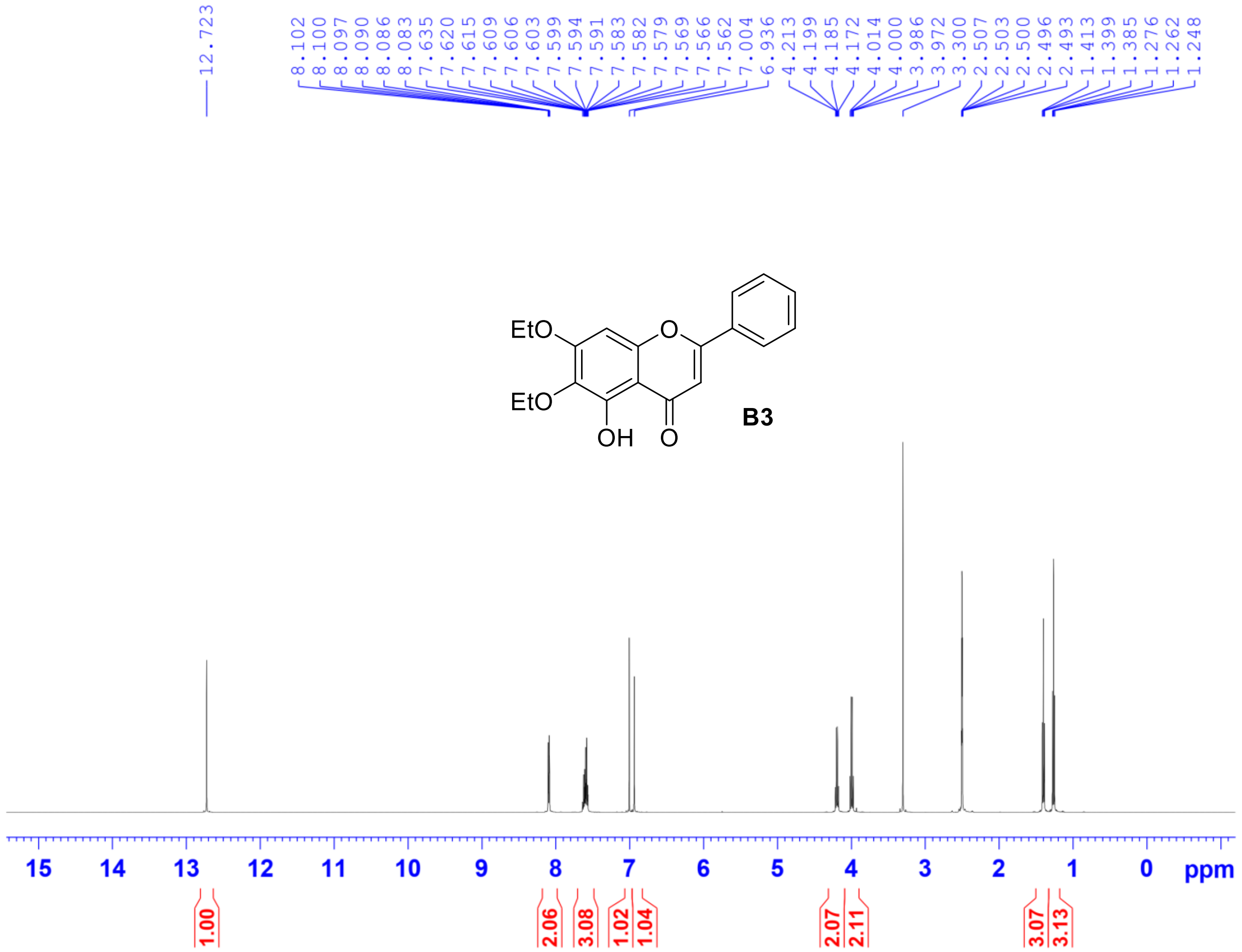


```
Current Data Parameters
NAME            110HUAN_BDE
EXPNO                    10
PROCNO                    1

F2 - Acquisition Parameters
Date_              20191126
Time                  11.33
INSTRUM               spect
PROBHD    5 mm PABBO BB/
PULPROG                zg30
TD                    65536
SOLVENT                DMSO
NS                       16
DS                        2
SWH              10000.000 Hz
FIDRES            0.152588 Hz
AQ               3.2767999 sec
RG                   111.09
DW                   50.000 usec
DE                     6.50 usec
TE                    303.9 K
D1              1.00000000 sec
TD0                       1

======== CHANNEL f1 ========
SFO1          500.1920889 MHz
NUC1                     1H
P1                    10.20 usec
PLW1            22.00000000 W

F2 - Processing parameters
SI                    65536
SF            500.1890047 MHz
WDW                      EM
SSB      0
LB                     0.30 Hz
GB       0
PC                     1.00
```

^{1}H-NMR

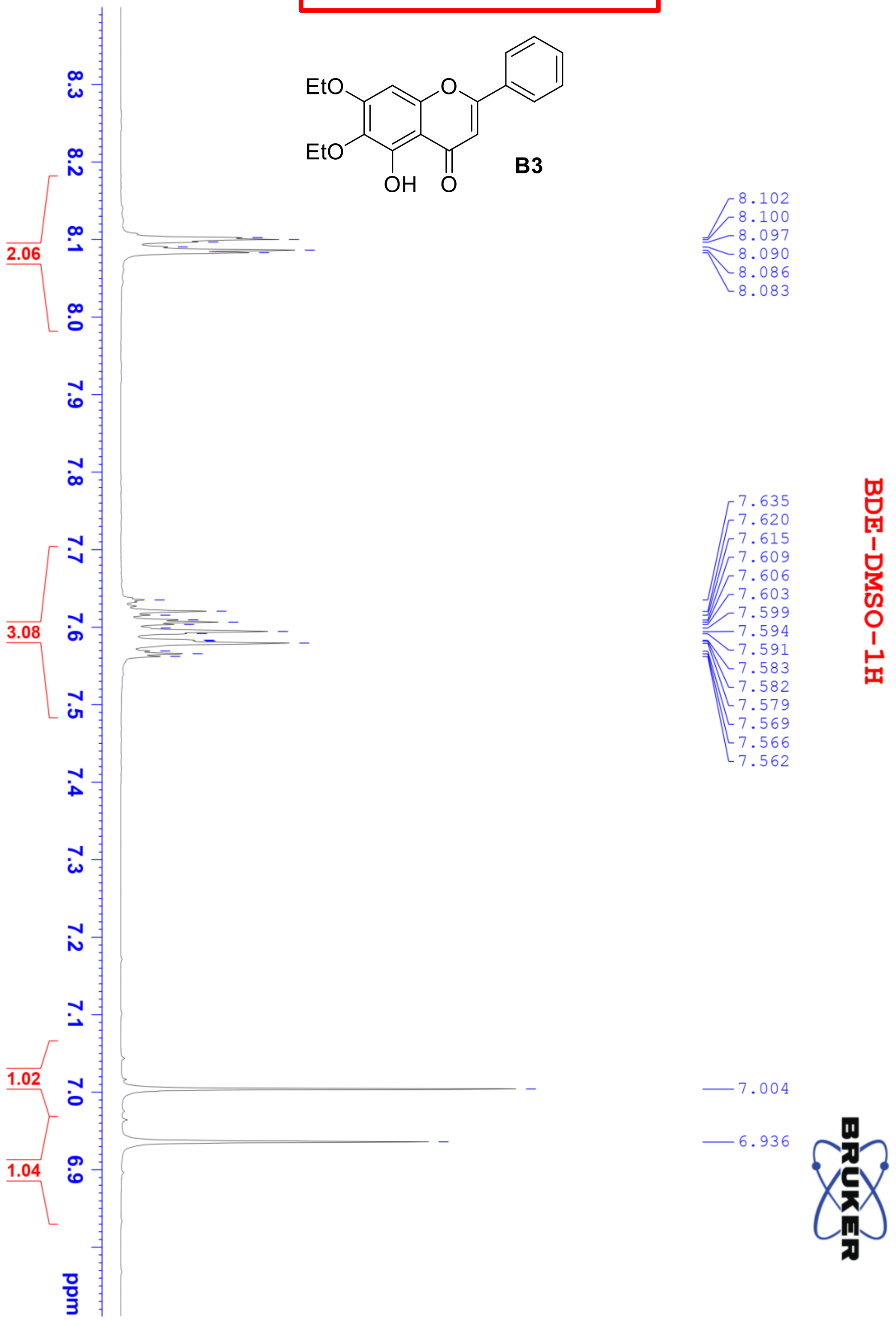

EtO
EtO
OH
O
O
8.102
8.100
8.097
8.090
8.086
8.083
7.635
7.620
7.615
7.609
7.606
7.603
7.599
7.594
7.591
7.583
7.582
7.579
7.569
7.566
7.562
7.004
6.936
2.06
3.08
1.02
1.04
8.3
8.2
8.1
8.0
7.9
7.8
7.7
7.6
7.5
7.4
7.3
7.2
7.1
7.0
6.9
ppm
BDE-DMSO-1H

B3

^{1}H-NMR

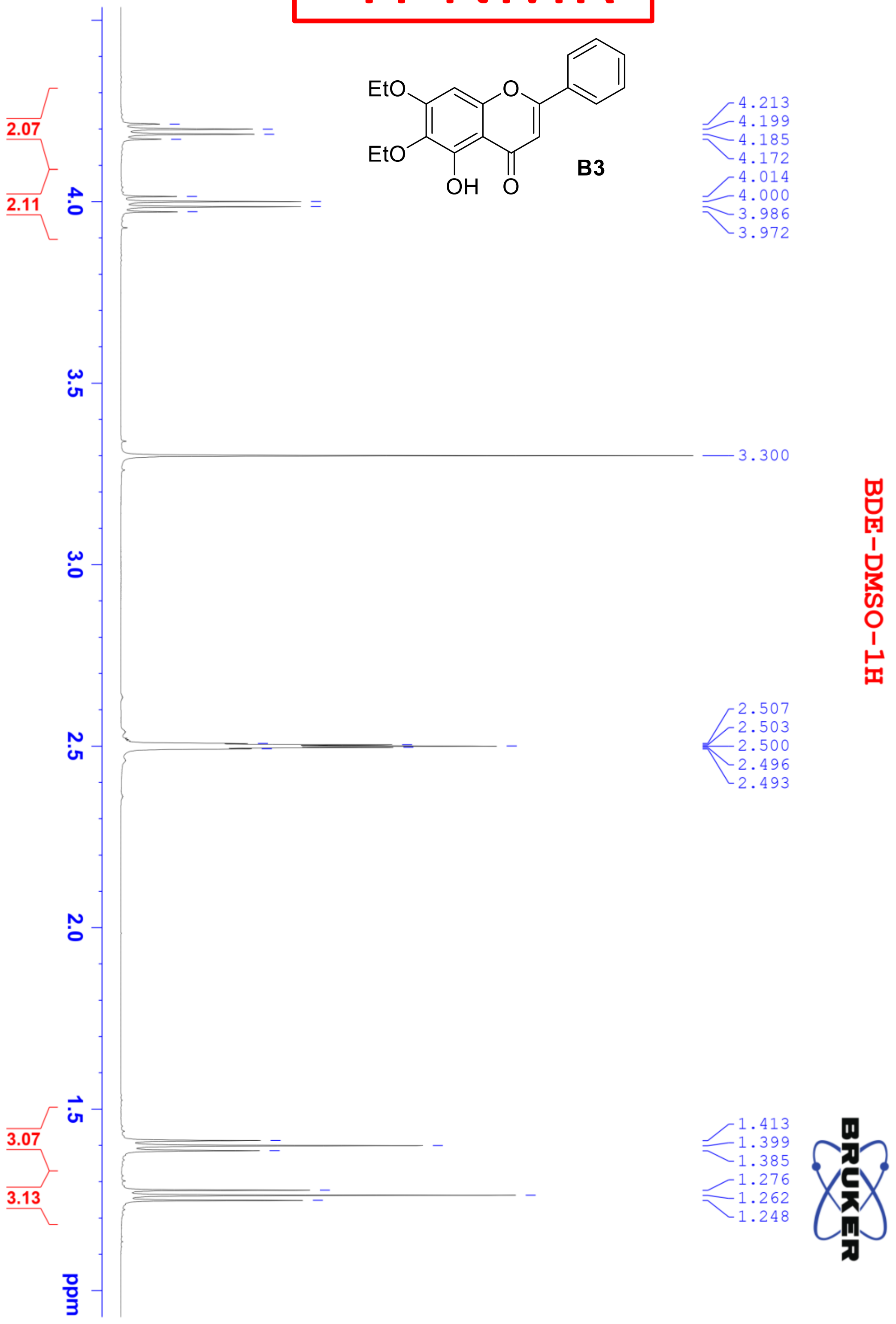

EtO
EtO
OH
O
O
B3
4.213
4.199
4.185
4.172
4.014
4.000
3.986
3.972
3.300
2.507
2.503
2.500
2.496
2.493
1.413
1.399
1.385
1.276
1.262
1.248
2.07
2.11
3.07
3.13
4.0
3.5
3.0
2.5
2.0
1.5
ppm
BDE-DMSO-1H

^{13}C-NMR

BDE-DMSO-C13CPD

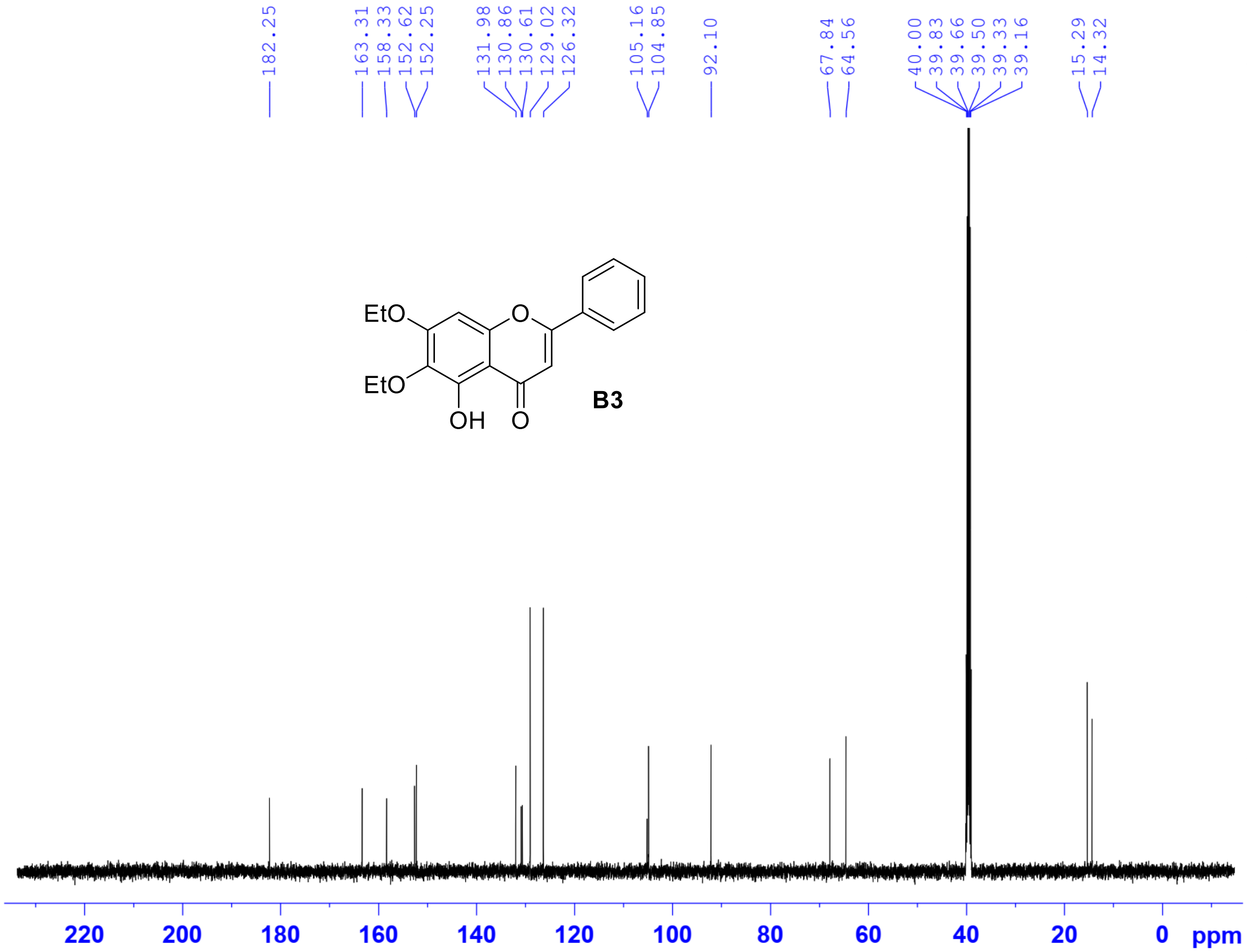


```
Current Data Parameters
NAME          110HUAN_BDE
EXPNO                   2
PROCNO                  1

F2 - Acquisition Parameters
Date_            20191126
Time                17.24
INSTRUM             spect
PROBHD   5 mm PABBO BB/
PULPROG           zgpg30
TD                 65536
SOLVENT             DMSO
NS                   256
DS                     4
SWH            31250.000 Hz
FIDRES          0.476837 Hz
AQ             1.0485760 sec
RG                198.57
DW                16.000 usec
DE                  6.50 usec
TE                 306.5 K
D1            2.00000000 sec
D11           0.03000000 sec
TD0                    1

======== CHANNEL f1 ========
SFO1        125.7864591 MHz
NUC1                 13C
P1                 10.00 usec
PLW1         88.00000000 W

======== CHANNEL f2 ========
SFO2        500.1910008 MHz
NUC2                  1H
CPDPRG[2         waltz16
PCPD2              80.00 usec
PLW2         22.00000000 W
PLW12         0.35764000 W
PLW13         0.17989001 W

F2 - Processing parameters
SI                 32768
SF          125.7726952 MHz
WDW                   EM
SSB      0
LB                  1.00 Hz
GB       0
PC                  1.40
```

^{13}C-NMR

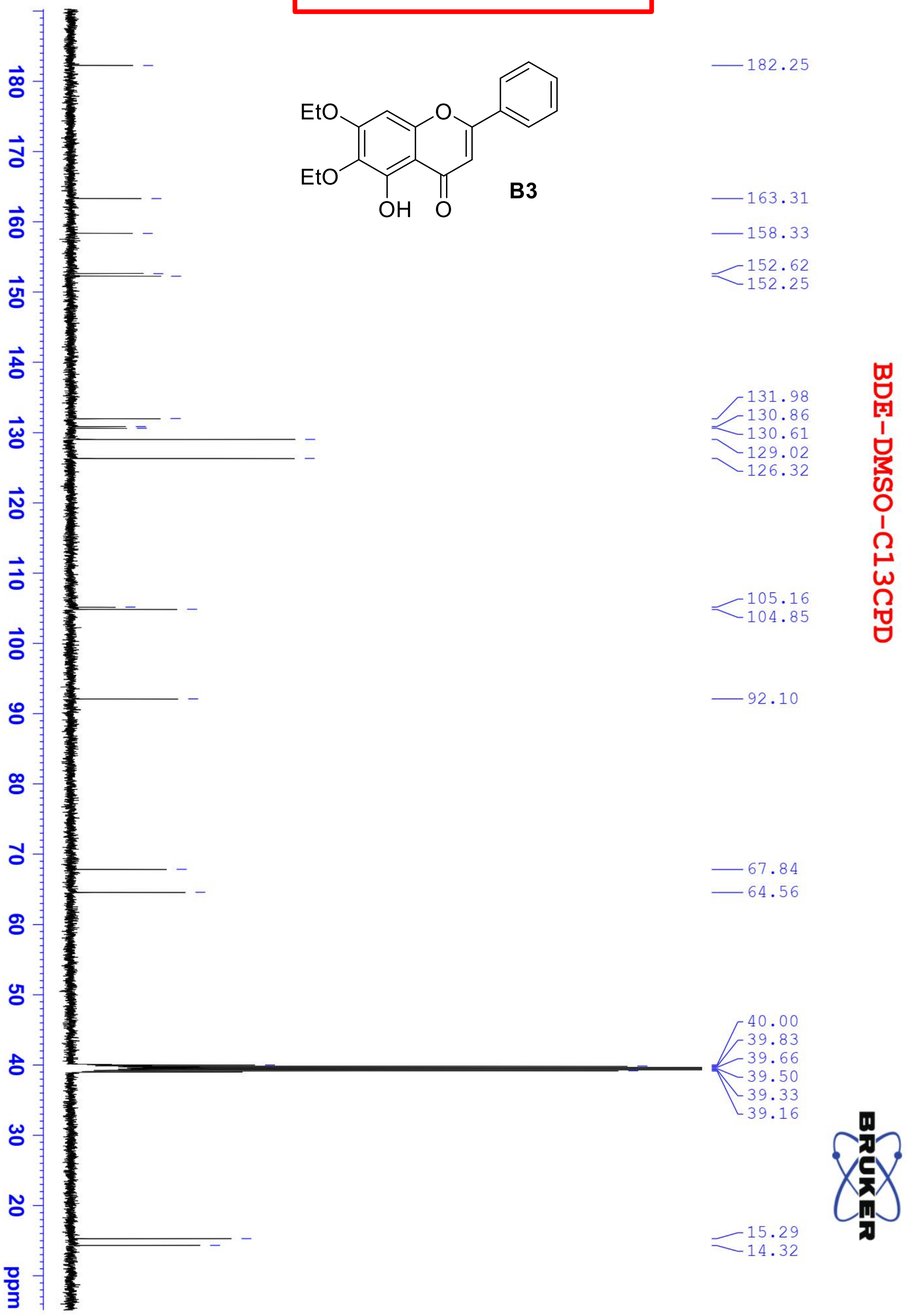

EtO
EtO
OH
O
O
B3
182.25
163.31
158.33
152.62
152.25
131.98
130.86
130.61
129.02
126.32
105.16
104.85
92.10
67.84
64.56
40.00
39.83
39.66
39.50
39.33
39.16
15.29
14.32
BDE-DMSO-C13CPD
BRUKER
ppm
180
170
160
150
140
130
120
110
100
90
80
70
60
50
40
30
20

^{13}C-NMR

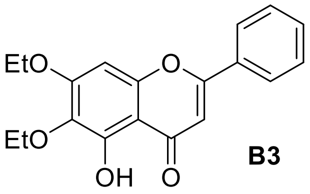

B3

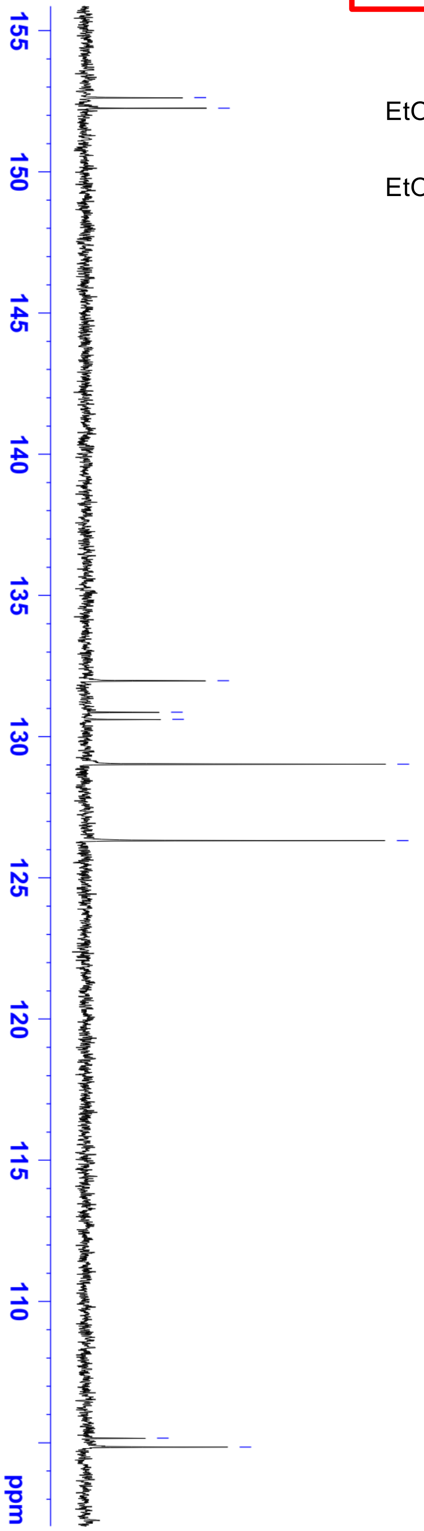

155
150
145
140
135
130
125
120
115
110
ppm
152.62
152.25
131.98
130.86
130.61
129.02
126.32
105.16
104.85
BDE-DMSO-C13CPD

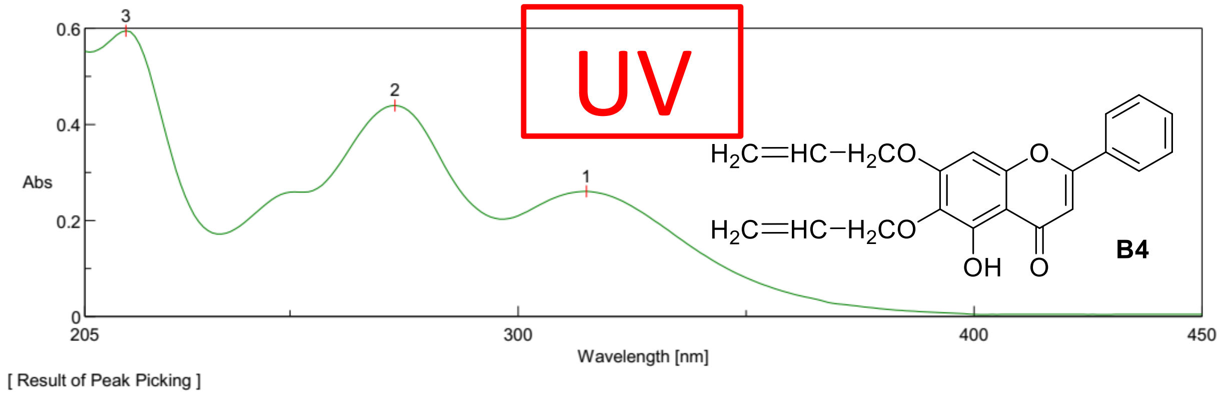

[Result of Peak Picking]

No.	Position	Intensity	No.	Position	Intensity	No.	Position	Intensity
1	315	0.260353	2	273	0.439249	3	214	0.594943

SHIMADZU

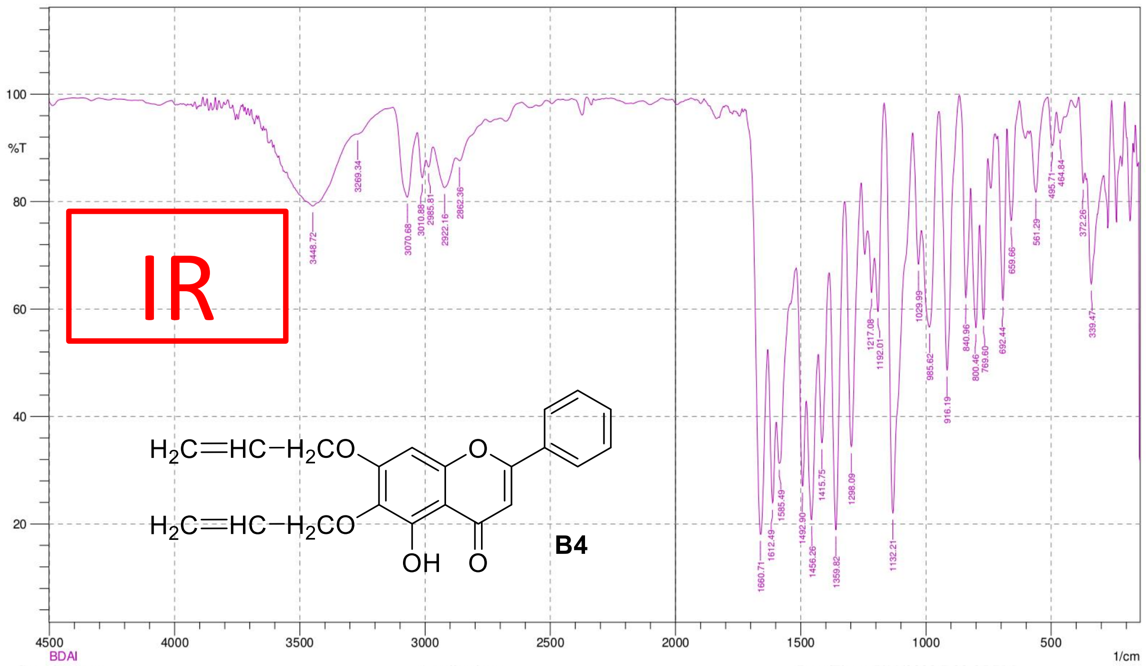

BDAI

Sample name
BDAl

Apodization;
Resolution;
No. of Scans;

Date/Time; 5/11/2020 7:32:35 PM
User; IR-Prestige

MS

Qualitative Analysis Report

**Data Filename**	BDAI 6.d	**Sample Name**	BDAI 6
**Sample Type**	Sample	**Position**	P2-C5
**Instrument Name**	Instrument 1	**User Name**	
**Acq Method**	Cot ngan - MSMS_Pos.m	**Acquired Time**	04/08/2020 8:24:25 PM
**IRM Calibration Status**	Success	**DA Method**	COTNGAN.M.m
**Comment**			

**Sample Group**		**Info.**	
**Stream Name**	LC 1	**Acquisition SW Version**	6200 series TOF/6500 series Q-TOF B.06.01 (B6172 SP1)

User Chromatograms

Fragmentor Voltage 140 **Collision Energy** 0 **Ionization Mode** ESI

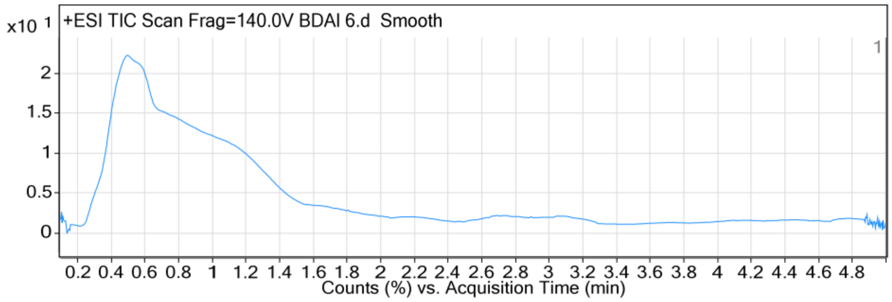

User Spectra

Fragmentor Voltage	Collision Energy	Ionization Mode
140	0	ESI

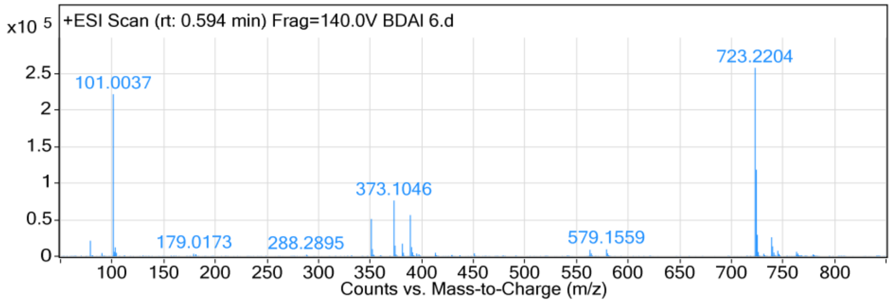

Peak List

m/z	z	Abund
79.0213	1	21162.16
101.0037	1	221657.94
351.1224	1	50865.48
373.1046	1	76269.85
381.2974	1	17324.84
389.0783	1	56522.68
723.2204	1	257544.19
724.223	1	118534.92
725.2257	1	29415.95
739.193	1	26186.25

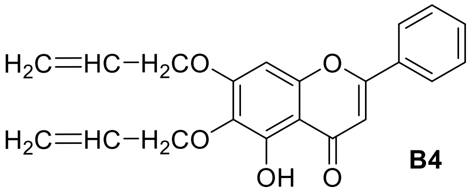

B4

--- End Of Report ---

MS

Sample Name	BDAl 6	Position	P2-C5	Instrument Name	Instrument 1	User Name	
Inj Vol	2	InjPosition		SampleType	Sample	IRM Calibration Status	Success
Data Filename	BDAl 6.d	ACQ Method	Cot ngan - MSMS_Pos.	Comment		Acquired Time	04/08/2020 8:24:25 PM

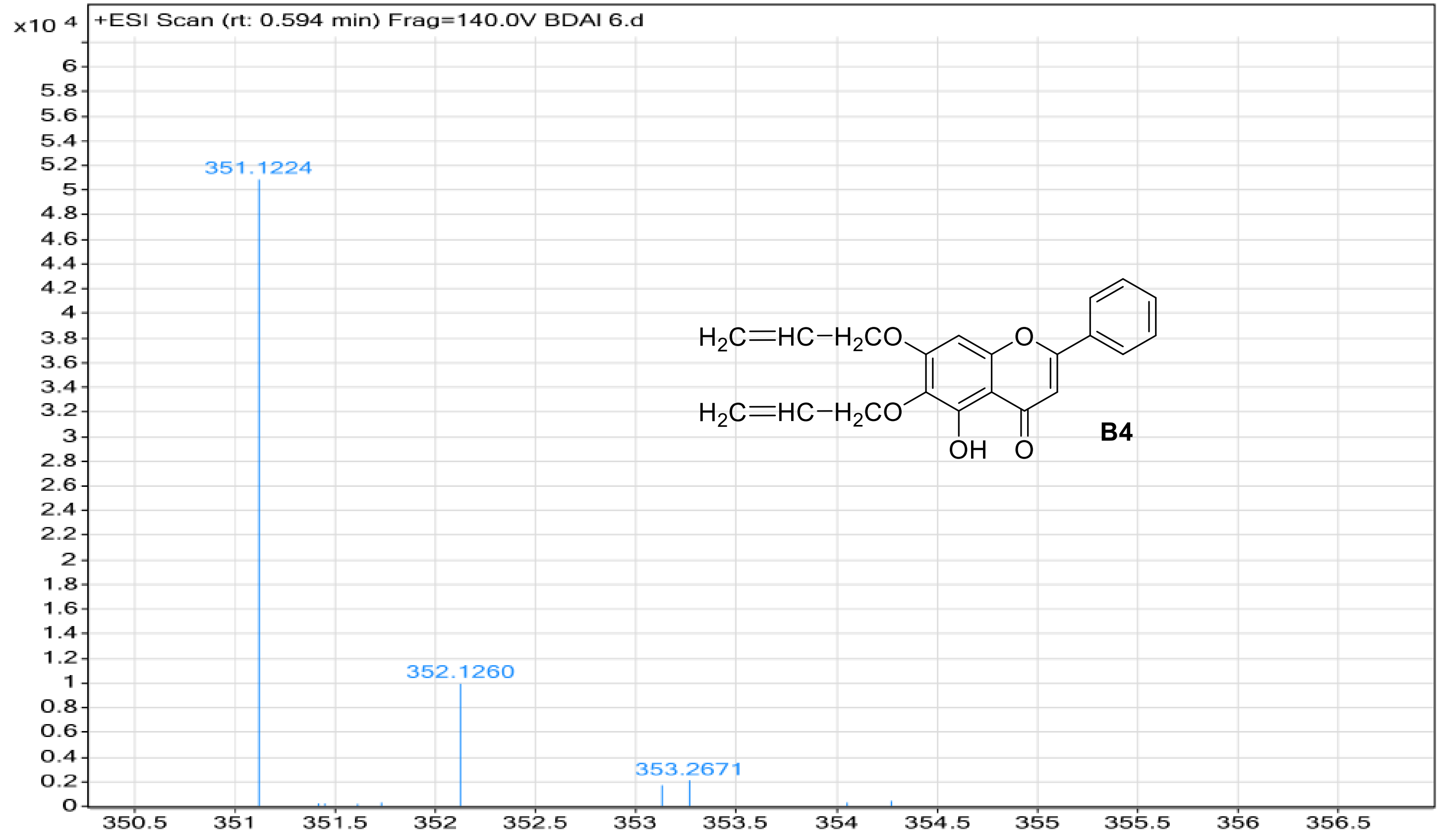

^{1}H-NMR

BDA1-DMSO-1H

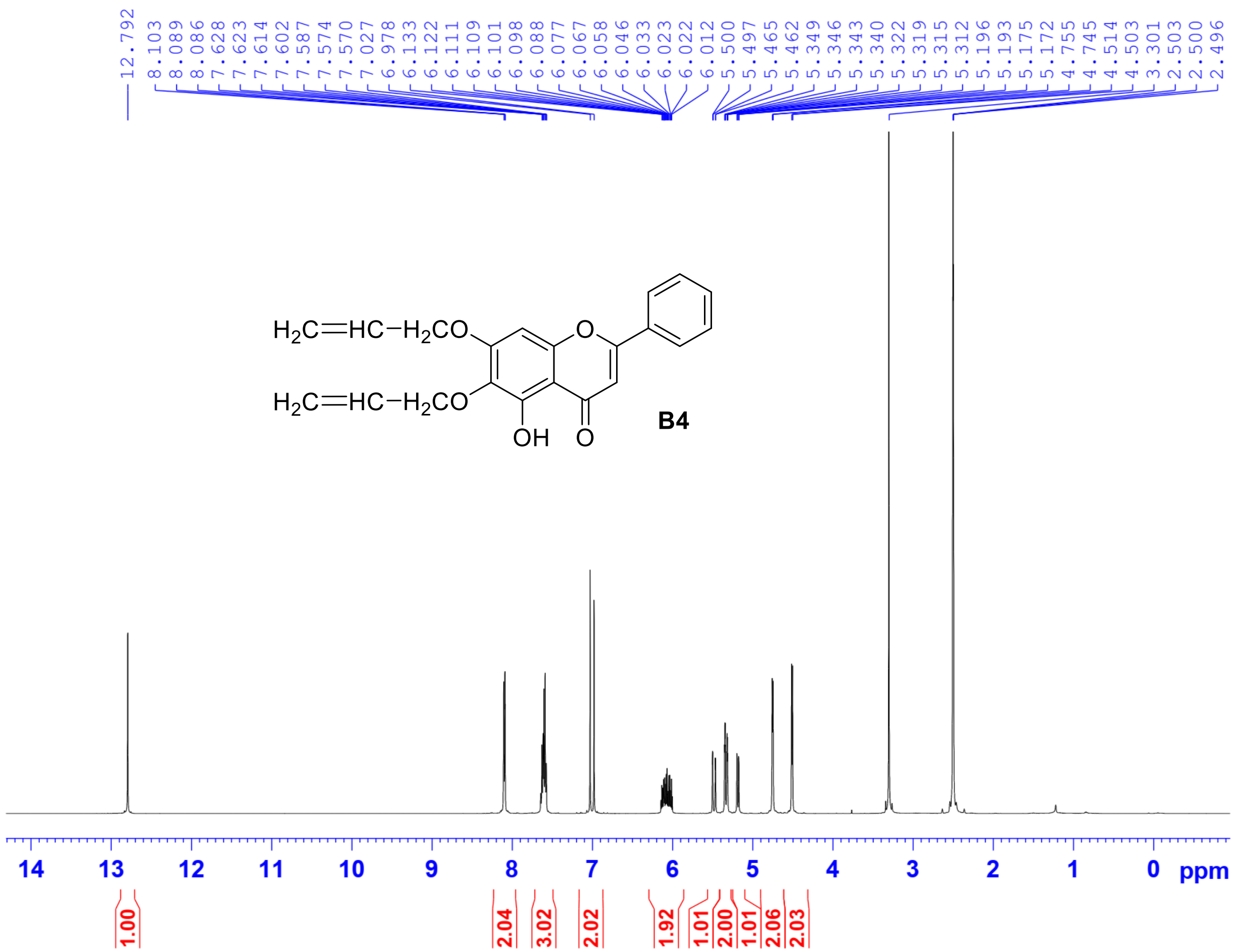


```
Current Data Parameters
NAME          110HUAN_BDA1
EXPNO                   10
PROCNO                   1

F2 - Acquisition Parameters
Date_             20191126
Time                 11.29
INSTRUM              spect
PROBHD   5 mm PABBO BB/
PULPROG               zg30
TD                   65536
SOLVENT               DMSO
NS                      16
DS                       2
SWH              10000.000 Hz
FIDRES            0.152588 Hz
AQ               3.2767999 sec
RG                  127.68
DW                  50.000 usec
DE                    6.50 usec
TE                   303.9 K
D1              1.00000000 sec
TD0                      1

======== CHANNEL f1 ========
SFO1           500.1920889 MHz
NUC1                    1H
P1                   10.20 usec
PLW1           22.00000000 W

F2 - Processing parameters
SI                   65536
SF             500.1890052 MHz
WDW                     EM
SSB      0
LB                    0.30 Hz
GB       0
PC                    1.00
```

^{1}H-NMR

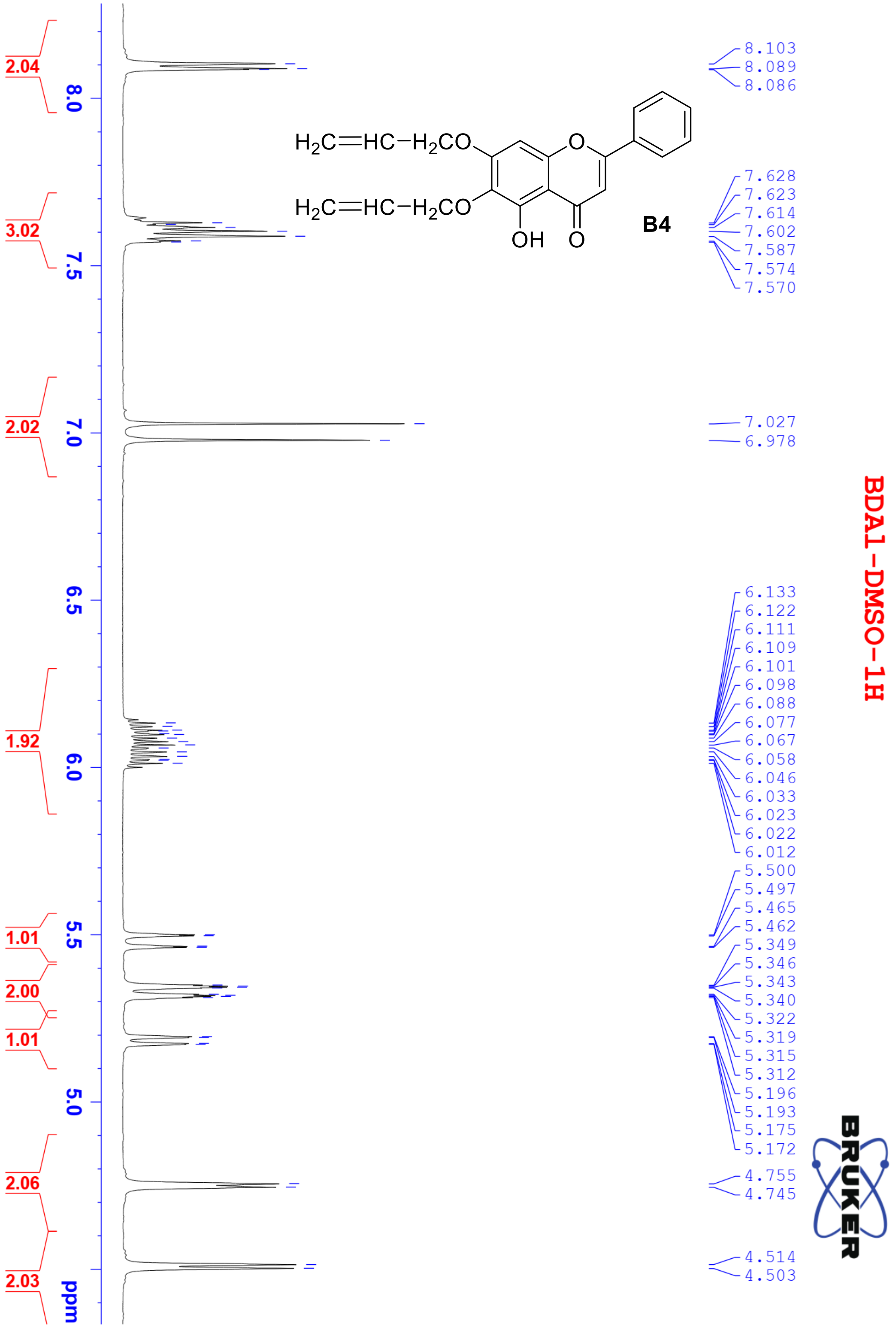

8.103
8.089
8.086
7.628
7.623
7.614
7.602
7.587
7.574
7.570
7.027
6.978
6.133
6.122
6.111
6.109
6.101
6.098
6.088
6.077
6.067
6.058
6.046
6.033
6.023
6.022
6.012
5.500
5.497
5.465
5.462
5.349
5.346
5.343
5.340
5.322
5.319
5.315
5.312
5.196
5.193
5.175
5.172
4.755
4.745
4.514
4.503
2.04
3.02
2.02
1.92
1.01
2.00
1.01
2.06
2.03
8.0
7.5
7.0
6.5
6.0
5.5
5.0
ppm
H2C=HC−H2CO
H2C=HC−H2CO
O
OH
O
B4
BDA1-DMSO-1H
BRUKER

^{13}C-NMR

BDA1-DMSO-C13CPD

BRUKER

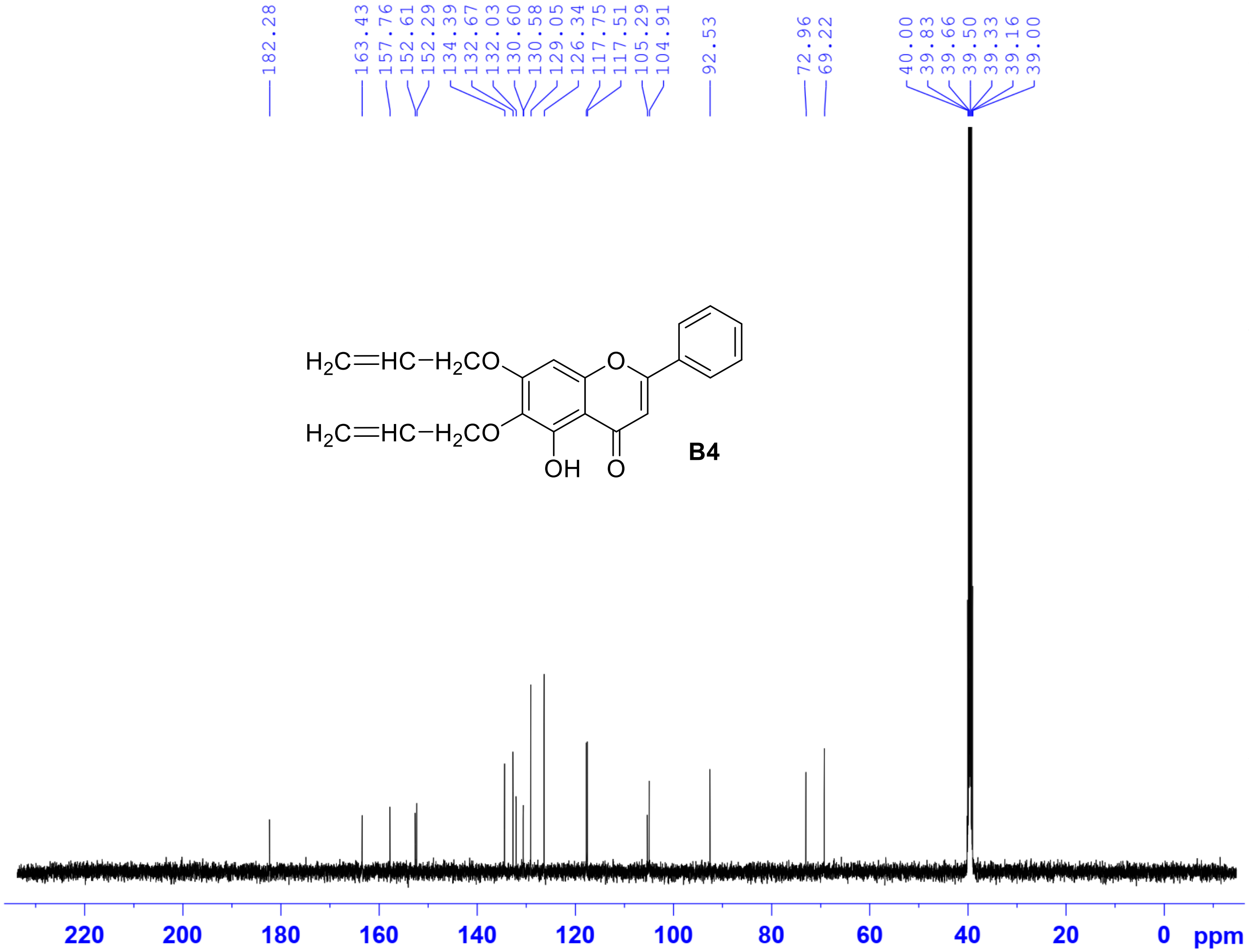

Current Data Parameters	
NAME	110HUAN_BDA1
EXPNO	2
PROCNO	1
F2 - Acquisition Parameters	
Date_	20191126
Time	17.15
INSTRUM	spect
PROBHD	5 mm PABBO BB/
PULPROG	zgpg30
TD	65536
SOLVENT	DMSO
NS	512
DS	4
SWH	31250.000 Hz
FIDRES	0.476837 Hz
AQ	1.0485760 sec
RG	198.57
DW	16.000 usec
DE	6.50 usec
TE	307.0 K
D1	2.00000000 sec
D11	0.03000000 sec
TD0	1
======== CHANNEL f1 ========	
SFO1	125.7864591 MHz
NUC1	13C
P1	10.00 usec
PLW1	88.00000000 W
======== CHANNEL f2 ========	
SFO2	500.1910008 MHz
NUC2	1H
CPDPRG[2	waltz16
PCPD2	80.00 usec
PLW2	22.00000000 W
PLW12	0.35764000 W
PLW13	0.17989001 W
F2 - Processing parameters	
SI	32768
SF	125.7726956 MHz
WDW	EM
SSB	0
LB	1.00 Hz
GB	0
PC	1.40

^{13}C-NMR

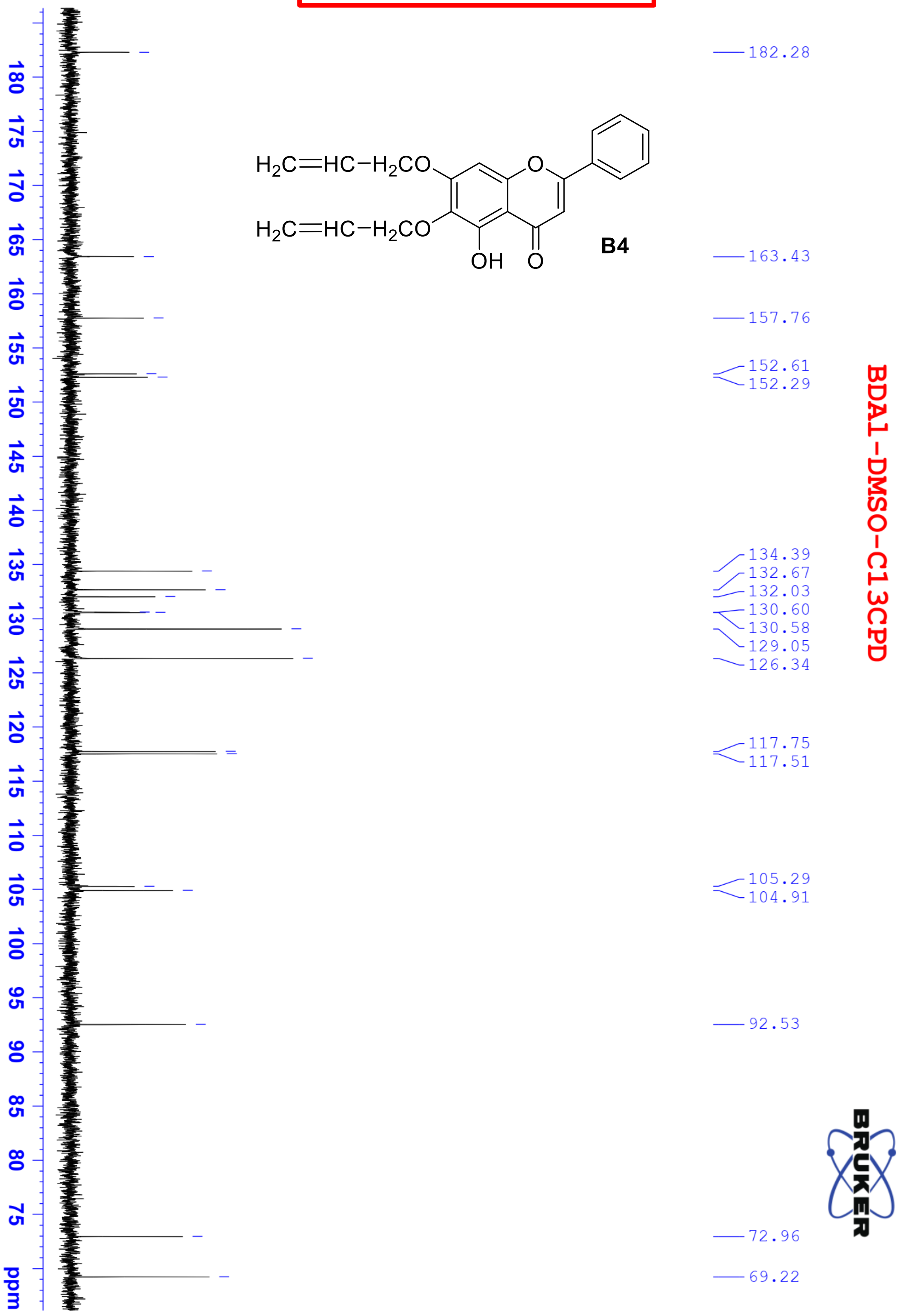

B4
182.28
163.43
157.76
152.61
152.29
134.39
132.67
132.03
130.60
130.58
129.05
126.34
117.75
117.51
105.29
104.91
92.53
72.96
69.22
BDA1-DMSO-C13CPD
ppm
180
175
170
165
160
155
150
145
140
135
130
125
120
115
110
105
100
95
90
85
80
75

^{13}C-NMR

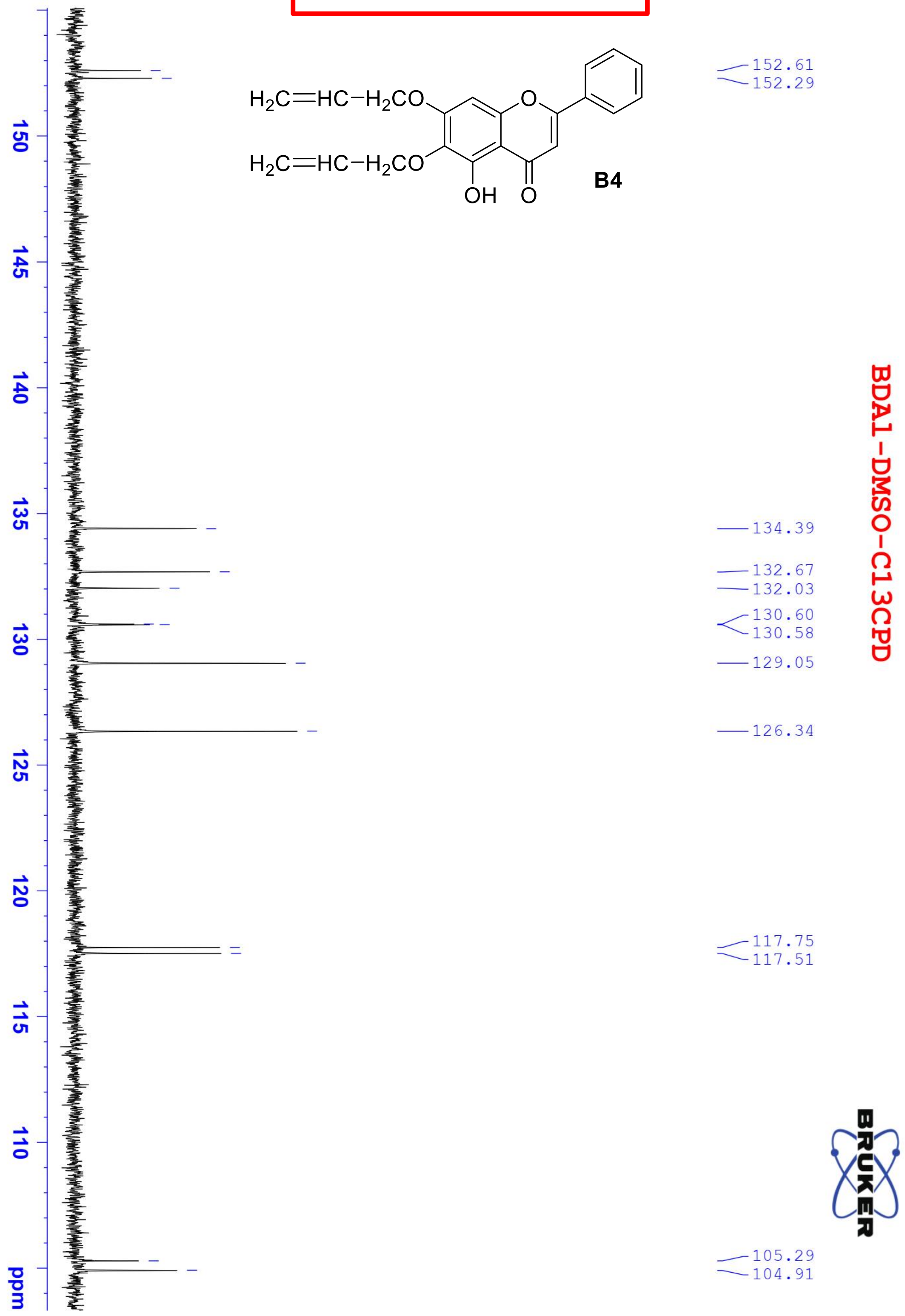

B4
152.61
152.29
134.39
132.67
132.03
130.60
130.58
129.05
126.34
117.75
117.51
105.29
104.91
150
145
140
135
130
125
120
115
110
ppm
BDA1-DMSO-C13CPD

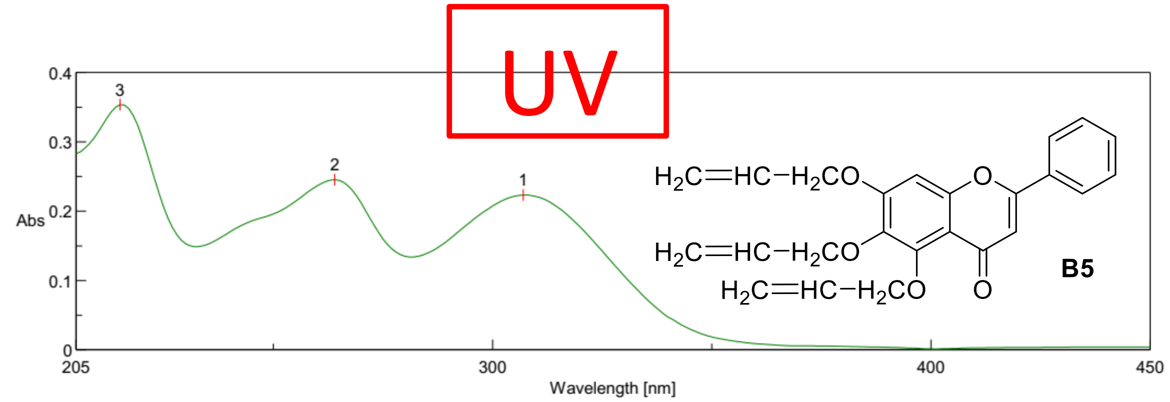

[Result of Peak Picking]

No.	Position	Intensity	No.	Position	Intensity	No.	Position	Intensity
1	307	0.223122	2	264	0.245246	3	215	0.353469

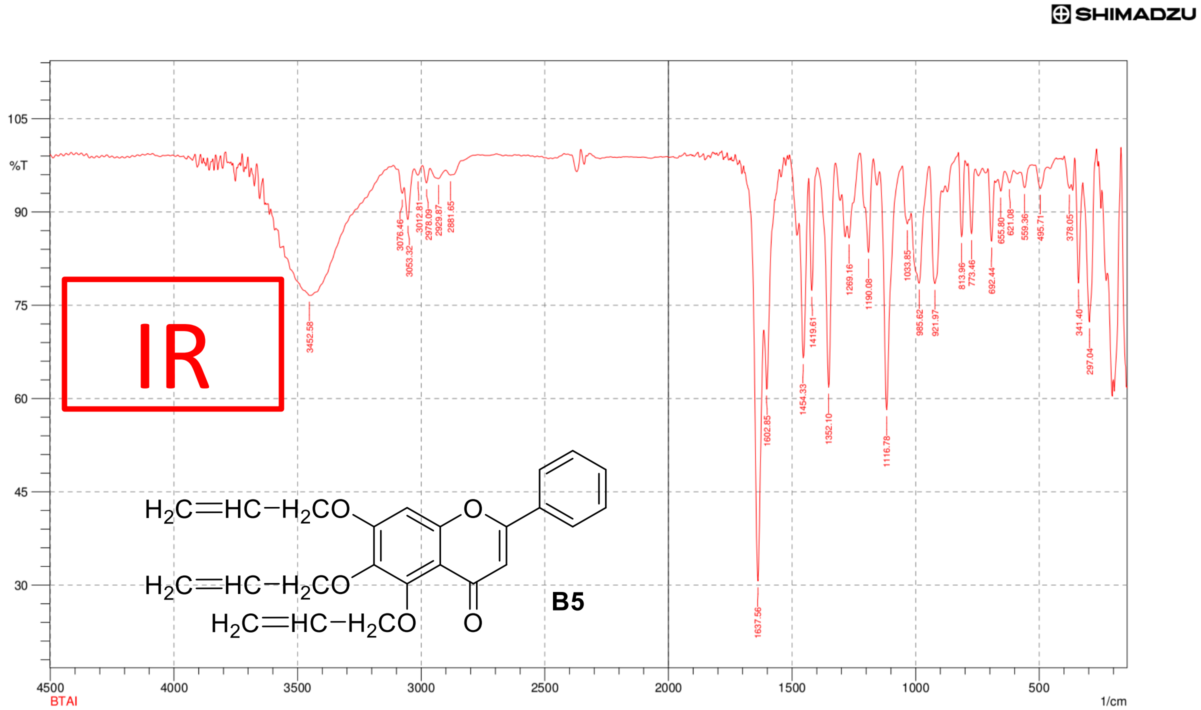

Sample name
BTAI

Apodization;
Resolution;
No. of Scans;

Date/Time; 5/8/2020 5:35:02 PM
User; IR-Prestige

MS

Qualitative Analysis Report

**Data Filename**	BTAI 7.d	**Sample Name**	BTAI 7
**Sample Type**	Sample	**Position**	P2-C3
**Instrument Name**	Instrument 1	**User Name**	
**Acq Method**	Cot ngan - MSMS_Pos.m	**Acquired Time**	04/08/2020 8:13:17 PM
**IRM Calibration Status**	Success	**DA Method**	COTNGAN.M.m
**Comment**			

**Sample Group**		**Info.**	
**Stream Name**	LC 1	**Acquisition SW Version**	6200 series TOF/6500 series Q-TOF B.06.01 (B6172 SP1)

User Chromatograms

Fragmentor Voltage 140 **Collision Energy** 0 **Ionization Mode** ESI

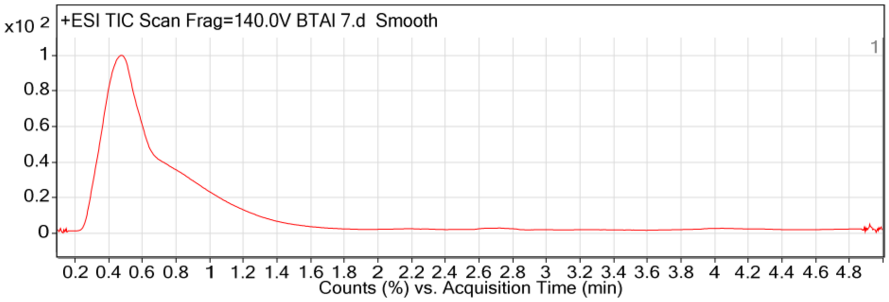

User Spectra

Fragmentor Voltage	Collision Energy	Ionization Mode
140	0	ESI

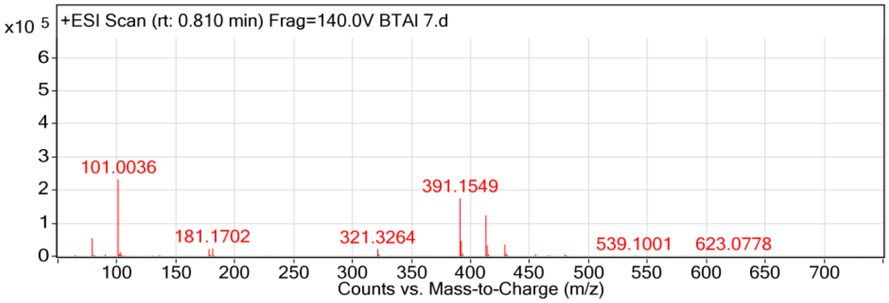

Peak List

m/z	z	Abund
79.0213	1	54313.98
101.0036	1	232908.31
391.1549	1	175124.36
392.1574	1	46811.03
413.1363	1	124046.12
803.2834	1	568929.69
804.2872	1	294397.13
805.2892	1	88924.84
819.257	1	166365.66
820.2596	1	80525.21

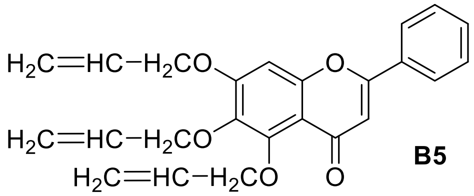

B5

--- End Of Report ---

MS

**Sample Name**	BTAl 7	**Position**	P2-C3	**Instrument Name**	Instrument 1	**User Name**	
**Inj Vol**	2	**InjPosition**		**SampleType**	Sample	**IRM Calibration Status**	Success
**Data Filename**	BTAl 7.d	**ACQ Method**	Cot ngan - MSMS_Pos.	**Comment**		**Acquired Time**	04/08/2020 8:13:17 PM

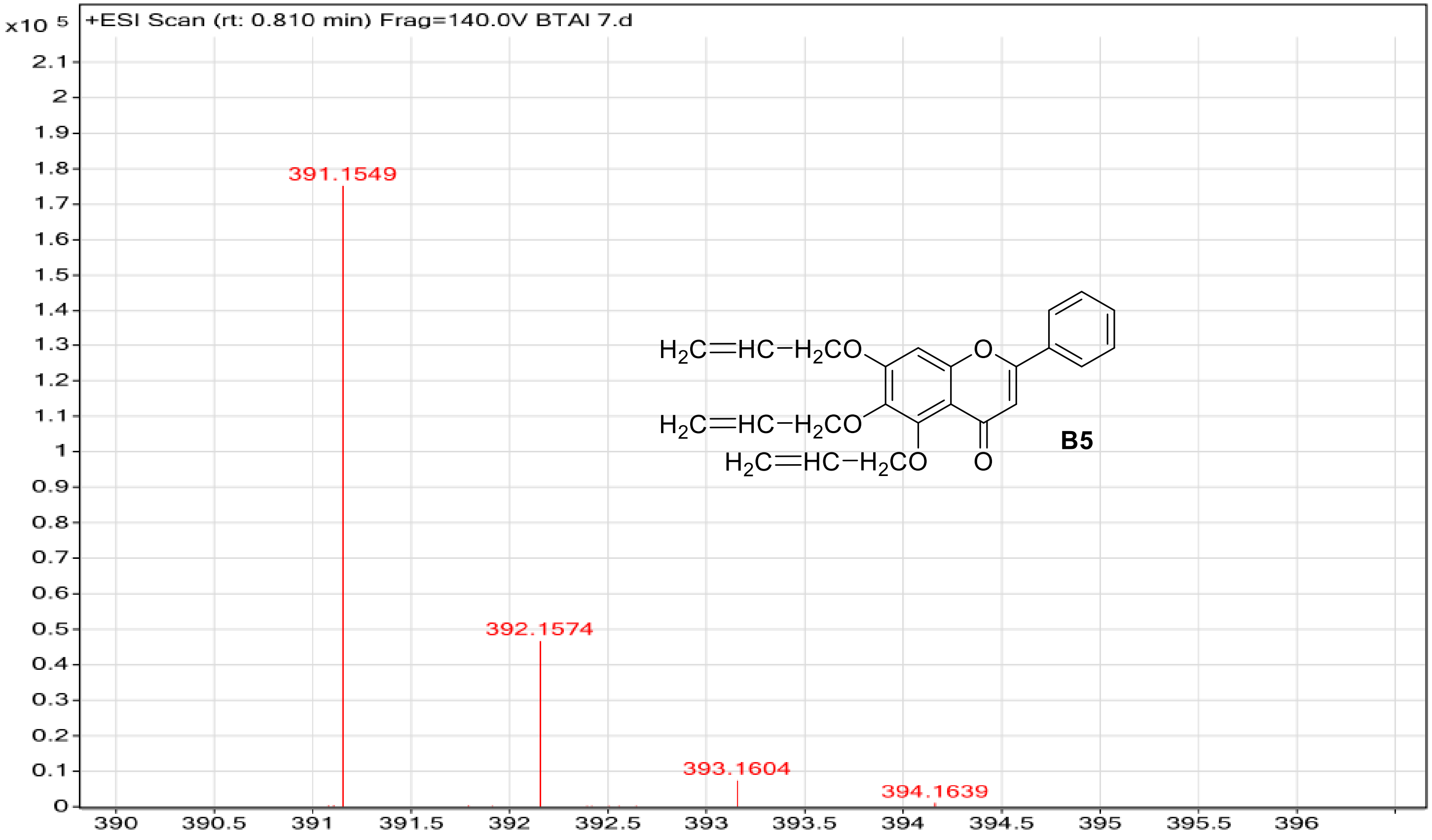

^{1}H-NMR

BTA1-DMSO-1H

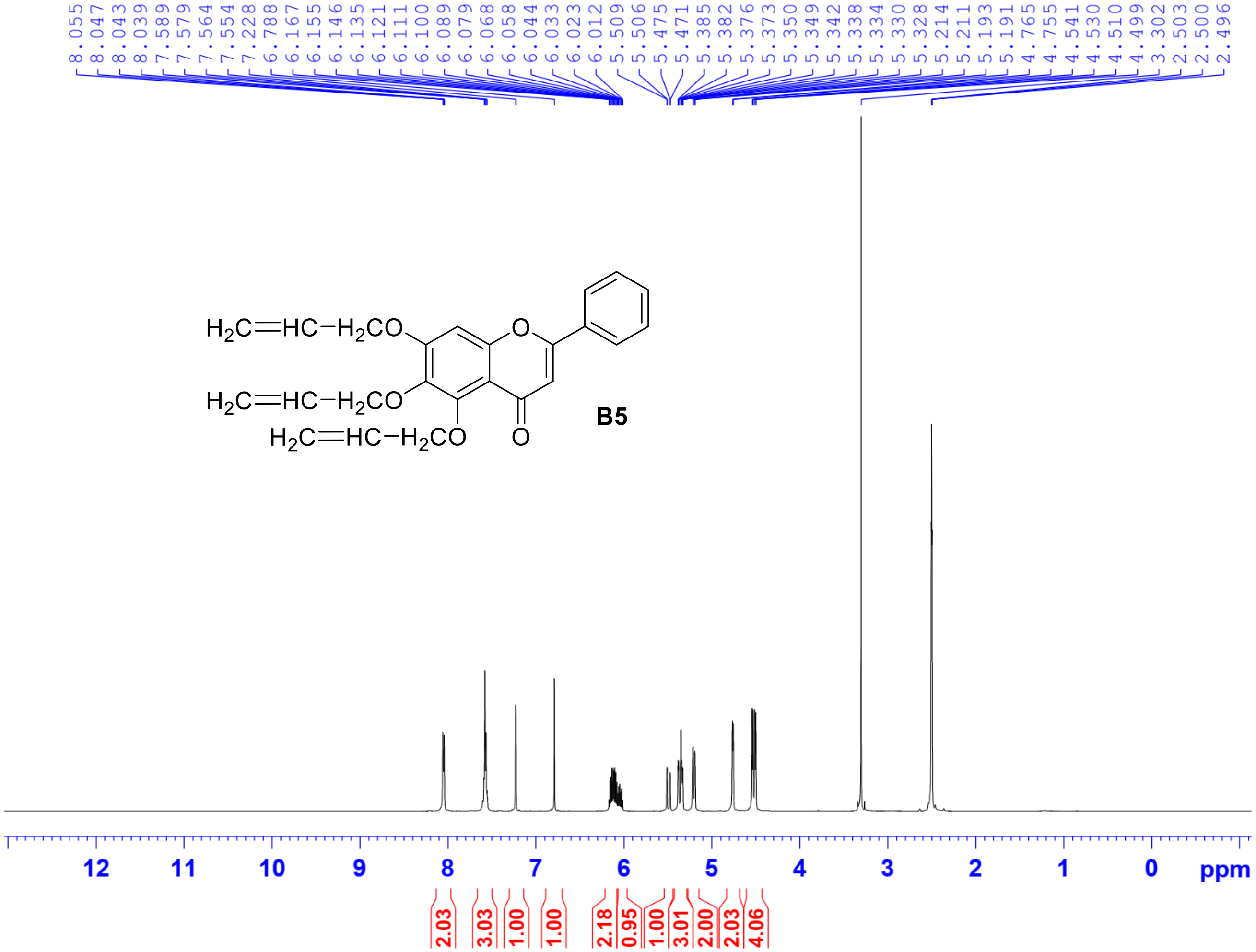

BRUKER

Current Data Parameters
NAME 110HUAN_BTA1
EXPNO 10
PROCNO 1

F2 - Acquisition Parameters
Date_ 20191126
Time 11.18
INSTRUM spect
PROBHD 5 mm PABBO BB/
PULPROG zg30
TD 65536
SOLVENT DMSO
NS 16
DS 2
SWH 10000.000 Hz
FIDRES 0.152588 Hz
AQ 3.2767999 sec
RG 111.09
DW 50.000 usec
DE 6.50 usec
TE 304.0 K
D1 1.00000000 sec
TD0 1

======== CHANNEL f1 ========
SFO1 500.1920889 MHz
NUC1 1H
P1 10.20 usec
PLW1 22.00000000 W

F2 - Processing parameters
SI 65536
SF 500.1890053 MHz
WDW EM
SSB 0
LB 0.30 Hz
GB 0
PC 1.00

^{1}H-NMR

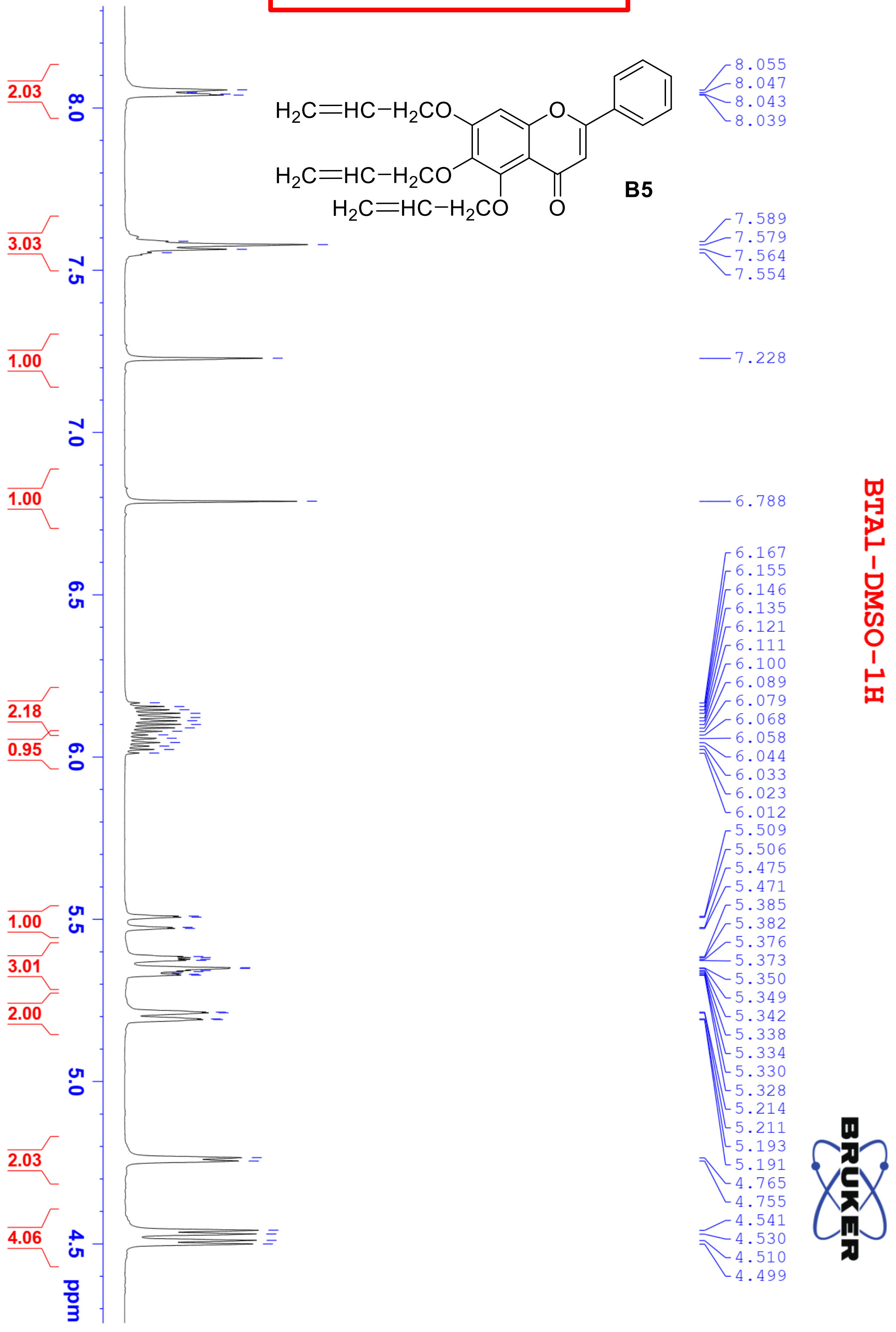

B5
8.055
8.047
8.043
8.039
7.589
7.579
7.564
7.554
7.228
6.788
6.167
6.155
6.146
6.135
6.121
6.111
6.100
6.089
6.079
6.068
6.058
6.044
6.033
6.023
6.012
5.509
5.506
5.475
5.471
5.385
5.382
5.376
5.373
5.350
5.349
5.342
5.338
5.334
5.330
5.328
5.214
5.211
5.193
5.191
4.765
4.755
4.541
4.530
4.510
4.499
2.03
3.03
1.00
1.00
2.18
0.95
1.00
3.01
2.00
2.03
4.06
8.0
7.5
7.0
6.5
6.0
5.5
5.0
4.5
ppm
BTA1-DMSO-1H

^{1}H-NMR

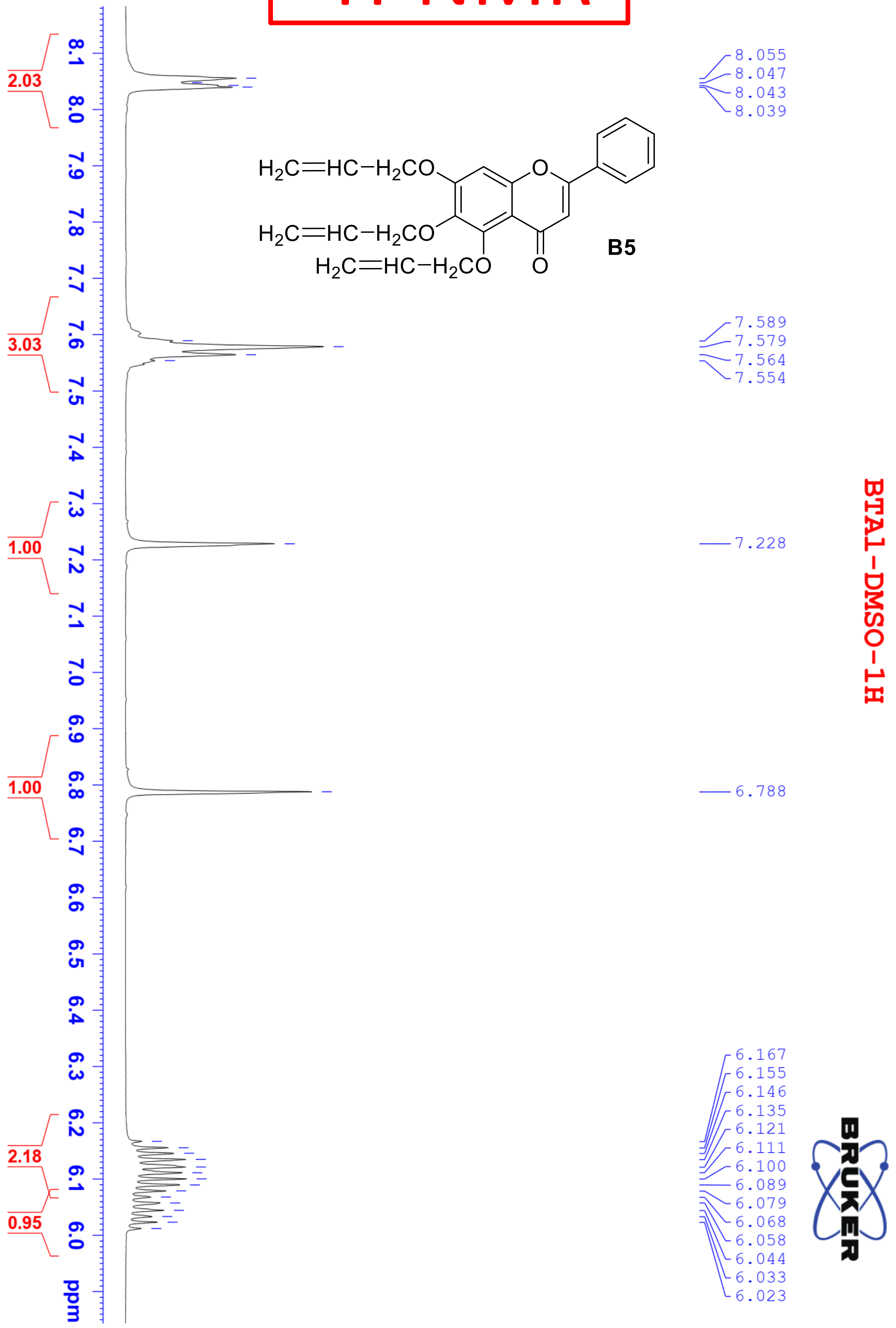

H2C=HC−H2CO
H2C=HC−H2CO
H2C=HC−H2CO
O
O
B5
8.055
8.047
8.043
8.039
7.589
7.579
7.564
7.554
7.228
6.788
6.167
6.155
6.146
6.135
6.121
6.111
6.100
6.089
6.079
6.068
6.058
6.044
6.033
6.023
2.03
3.03
1.00
1.00
2.18
0.95
8.1
8.0
7.9
7.8
7.7
7.6
7.5
7.4
7.3
7.2
7.1
7.0
6.9
6.8
6.7
6.6
6.5
6.4
6.3
6.2
6.1
6.0
ppm
BTA1-DMSO-1H

^{1}H-NMR

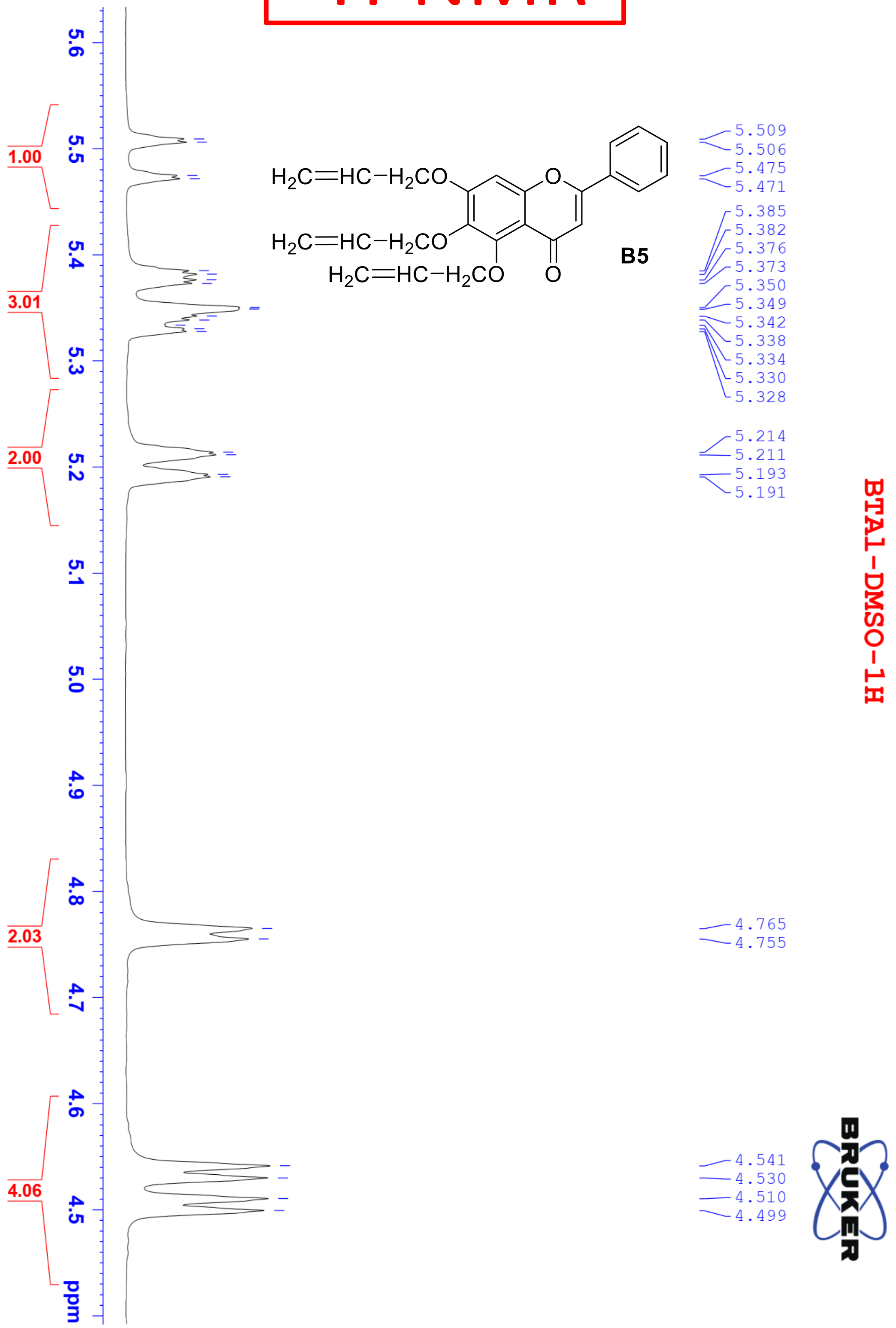

B5
BTA1-DMSO-1H
5.509
5.506
5.475
5.471
5.385
5.382
5.376
5.373
5.350
5.349
5.342
5.338
5.334
5.330
5.328
5.214
5.211
5.193
5.191
4.765
4.755
4.541
4.530
4.510
4.499
1.00
3.01
2.00
2.03
4.06
ppm

^{13}C-NMR

BTA1-DMSO-C13CPD

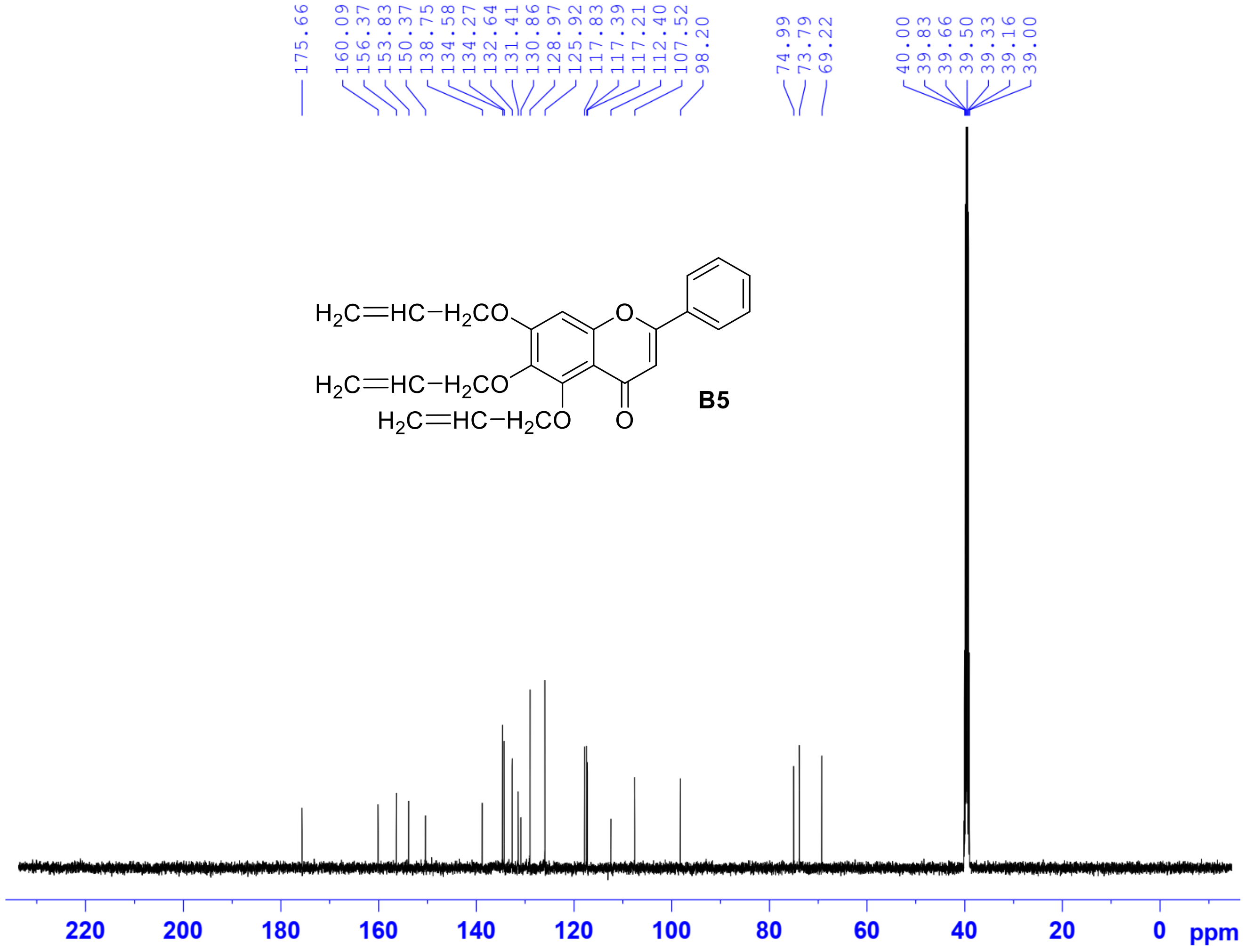

BRUKER

Current Data Parameters
NAME 110HUAN_BTA1
EXPNO 2
PROCNO 1

F2 - Acquisition Parameters
Date_ 20191126
Time 13.41
INSTRUM spect
PROBHD 5 mm PABBO BB/
PULPROG zgpg30
TD 65536
SOLVENT DMSO
NS 256
DS 4
SWH 31250.000 Hz
FIDRES 0.476837 Hz
AQ 1.0485760 sec
RG 198.57
DW 16.000 usec
DE 6.50 usec
TE 305.0 K
D1 2.00000000 sec
D11 0.03000000 sec
TD0 1

======== CHANNEL f1 ========
SFO1 125.7864591 MHz
NUC1 13C
P1 10.00 usec
PLW1 88.00000000 W

======== CHANNEL f2 ========
SFO2 500.1910008 MHz
NUC2 1H
CPDPRG[2 waltz16
PCPD2 80.00 usec
PLW2 22.00000000 W
PLW12 0.35764000 W
PLW13 0.17989001 W

F2 - Processing parameters
SI 32768
SF 125.7726941 MHz
WDW EM
SSB 0
LB 1.00 Hz
GB 0
PC 1.40

^{13}C-NMR

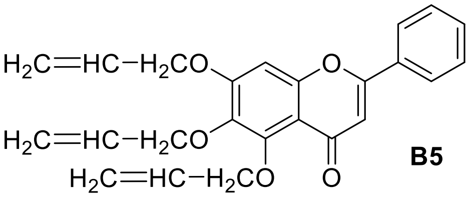

B5

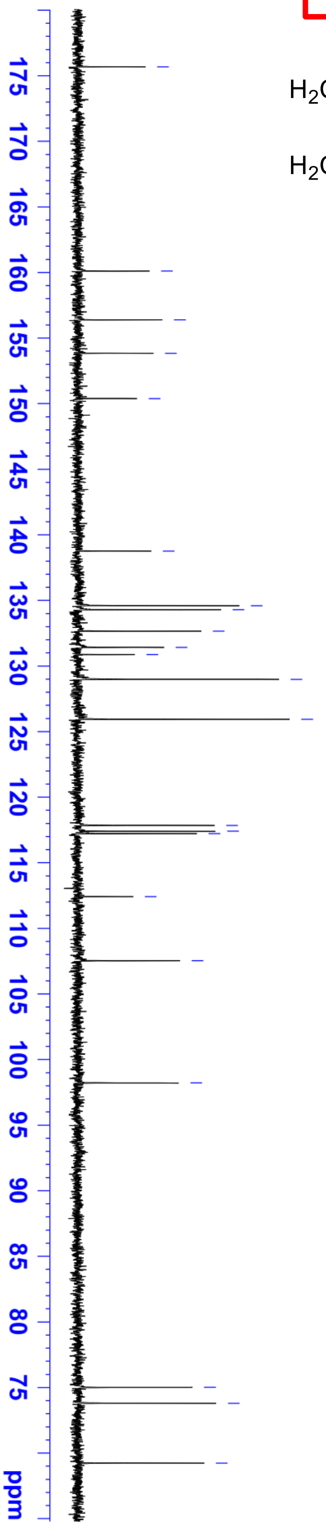
175.66
160.09
156.37
153.83
150.37
138.75
134.58
134.27
132.64
131.41
130.86
128.97
125.92
117.83
117.39
117.21
112.40
107.52
98.20
74.99
73.79
69.22
BTA1-DMSO-C13CPD
ppm

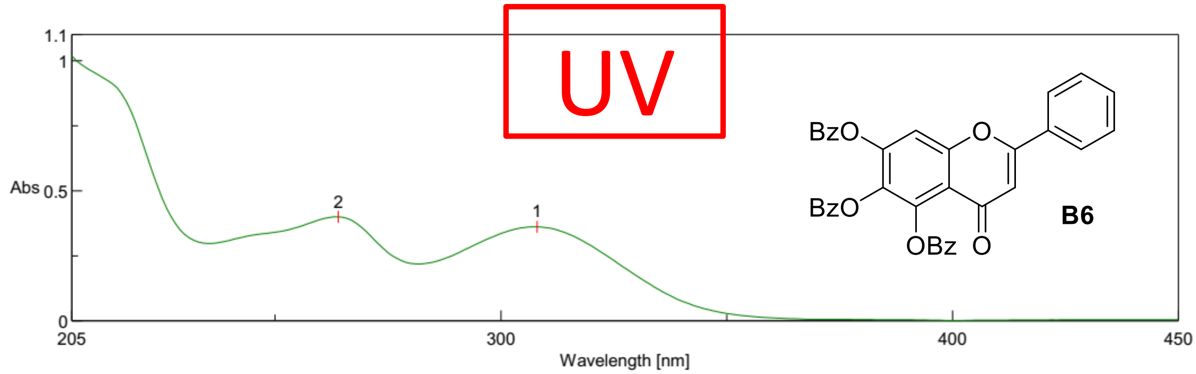

[Result of Peak Picking]

No.	Position	Intensity	No.	Position	Intensity	No.	Position	Intensity
1	308	0.361351	2	264	0.399643			

SHIMADZU

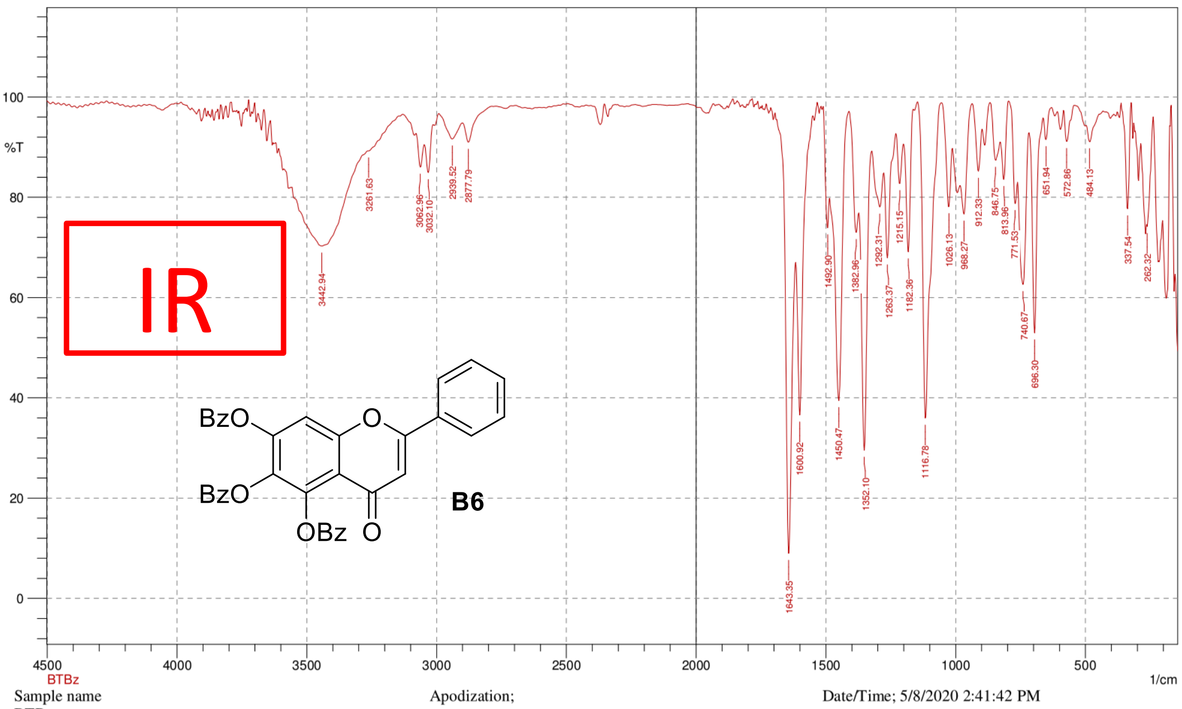

BTBz

Sample name
BTBz

Apodization;
Resolution;
No. of Scans;

Date/Time; 5/8/2020 2:41:42 PM
User; IR-Prestige

MS

Qualitative Analysis Report

**Data Filename**	BTBZ 9.d	**Sample Name**	BTBZ 9
**Sample Type**	Sample	**Position**	P2-C2
**Instrument Name**	Instrument 1	**User Name**	
**Acq Method**	Cot ngan - MSMS_Pos.m	**Acquired Time**	04/08/2020 8:07:43 PM
**IRM Calibration Status**	Success	**DA Method**	COTNGAN.M.m
**Comment**			

**Sample Group**		**Info.**	
**Stream Name**	LC 1	**Acquisition SW Version**	6200 series TOF/6500 series Q-TOF B.06.01 (B6172 SP1)

User Chromatograms

Fragmentor Voltage 140 **Collision Energy** 0 **Ionization Mode** ESI

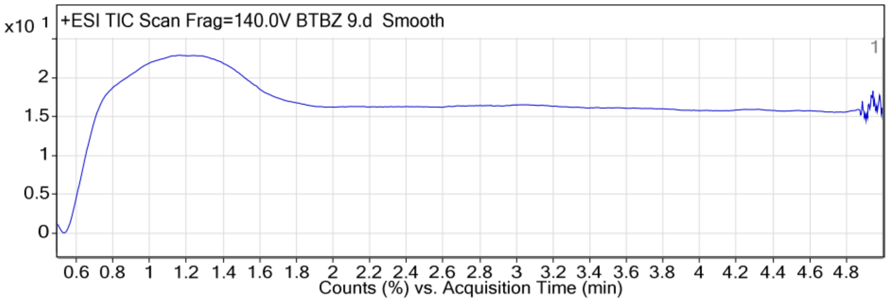

User Spectra

Fragmentor Voltage	Collision Energy	Ionization Mode
140	0	ESI

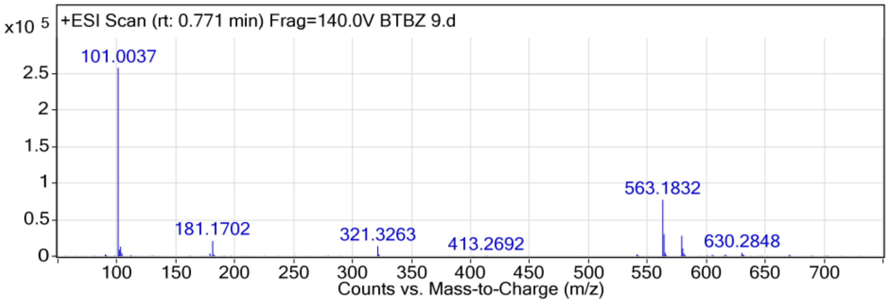

Peak List

m/z	z	Abund
101.0037	1	258011.27
102.006	1	8774.61
102.9991	1	13193.66
181.1702	1	20953.3
321.3263	1	13905.09
563.1832	1	77298.47
564.1861	1	30128.7
579.1568	1	28046.29
580.1599	1	10937.76
630.2848	1	4755.01

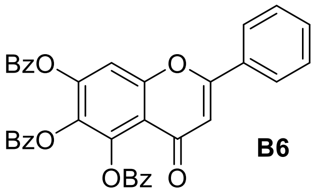

B6

--- End Of Report ---

MS

**Sample Name**	BTBZ 9	**Position**	P2-C2	**Instrument Name**	Instrument 1	**User Name**	
**Inj Vol**	2	**InjPosition**		**SampleType**	Sample	**IRM Calibration Status**	Success
**Data Filename**	BTBZ 9.d	**ACQ Method**	Cot ngan - MSMS_Pos.	**Comment**		**Acquired Time**	04/08/2020 8:07:43 PM

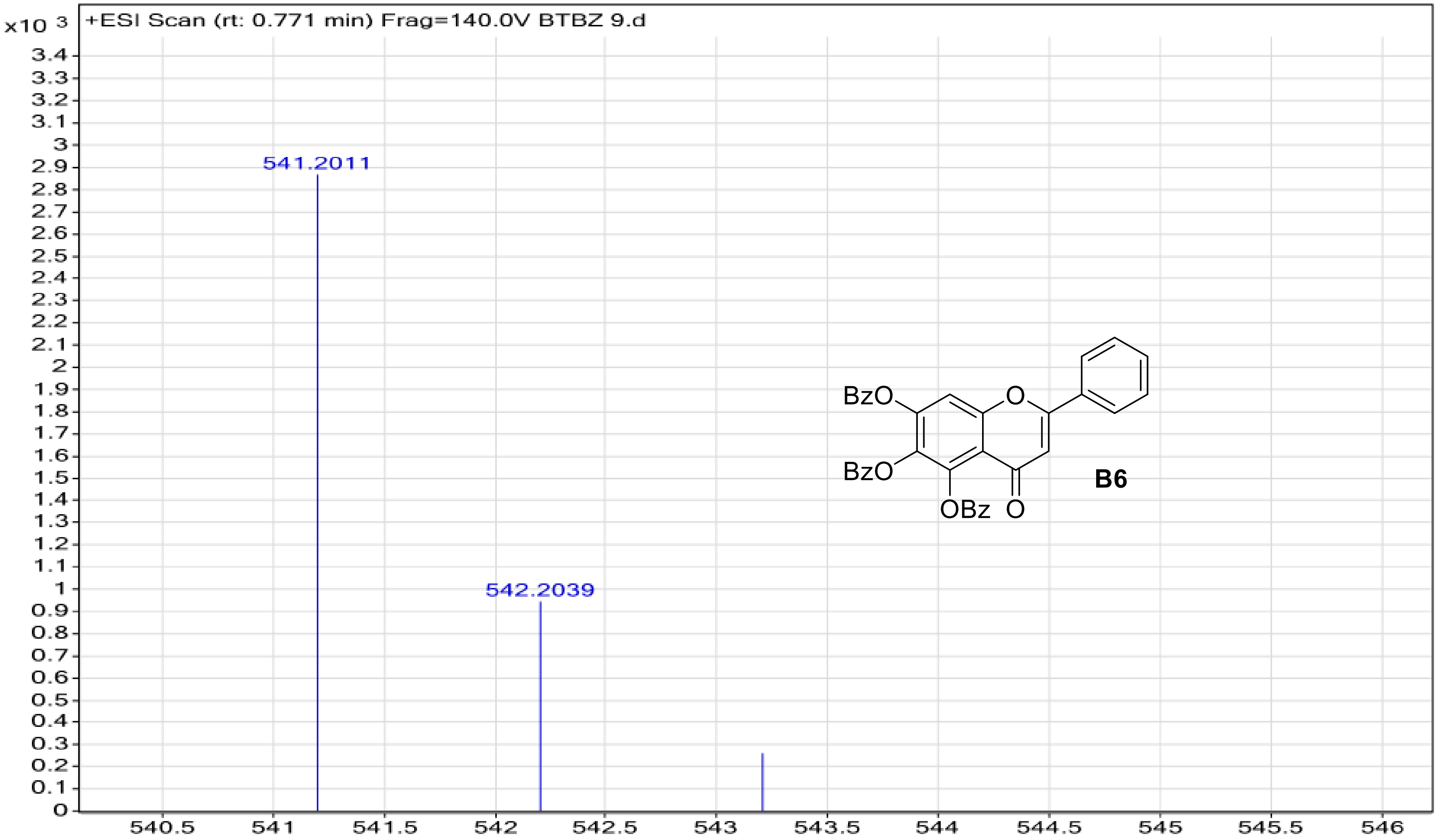

^{1}H-NMR

BTBZ-DMSO-1H

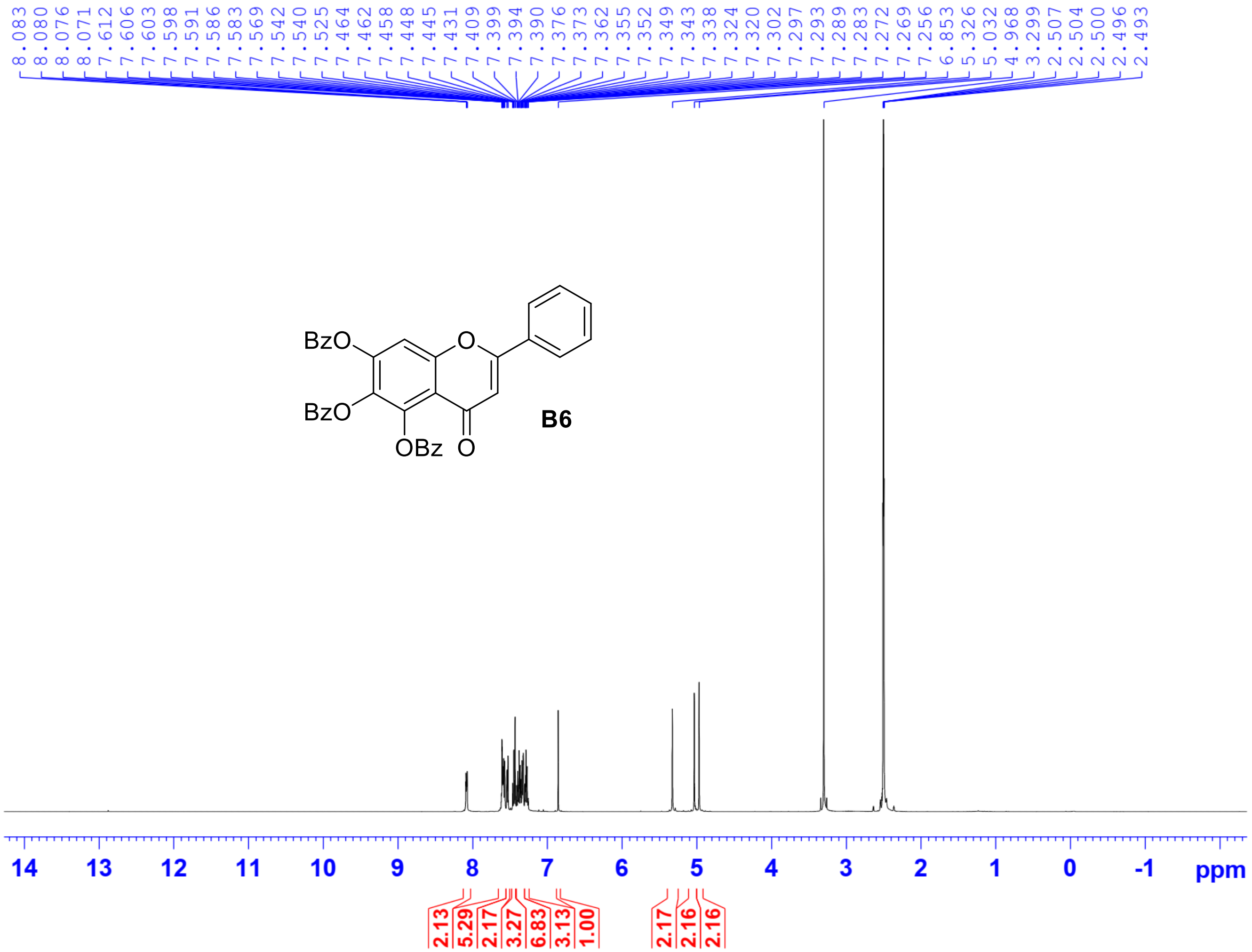


```
Current Data Parameters
NAME          110HUAN_BTBZ
EXPNO                   10
PROCNO                   1

F2 - Acquisition Parameters
Date_             20191126
Time                 11.25
INSTRUM              spect
PROBHD    5 mm PABBO BB/
PULPROG               zg30
TD                   65536
SOLVENT               DMSO
NS                      16
DS                       2
SWH              10000.000 Hz
FIDRES            0.152588 Hz
AQ               3.2767999 sec
RG                  157.35
DW                  50.000 usec
DE                    6.50 usec
TE                   303.9 K
D1              1.00000000 sec
TD0                      1

======== CHANNEL f1 ========
SFO1           500.1920889 MHz
NUC1                    1H
P1                   10.20 usec
PLW1           22.00000000 W

F2 - Processing parameters
SI                   65536
SF             500.1890048 MHz
WDW                     EM
SSB      0
LB                    0.30 Hz
GB       0
PC                    1.00
```

^{1}H-NMR

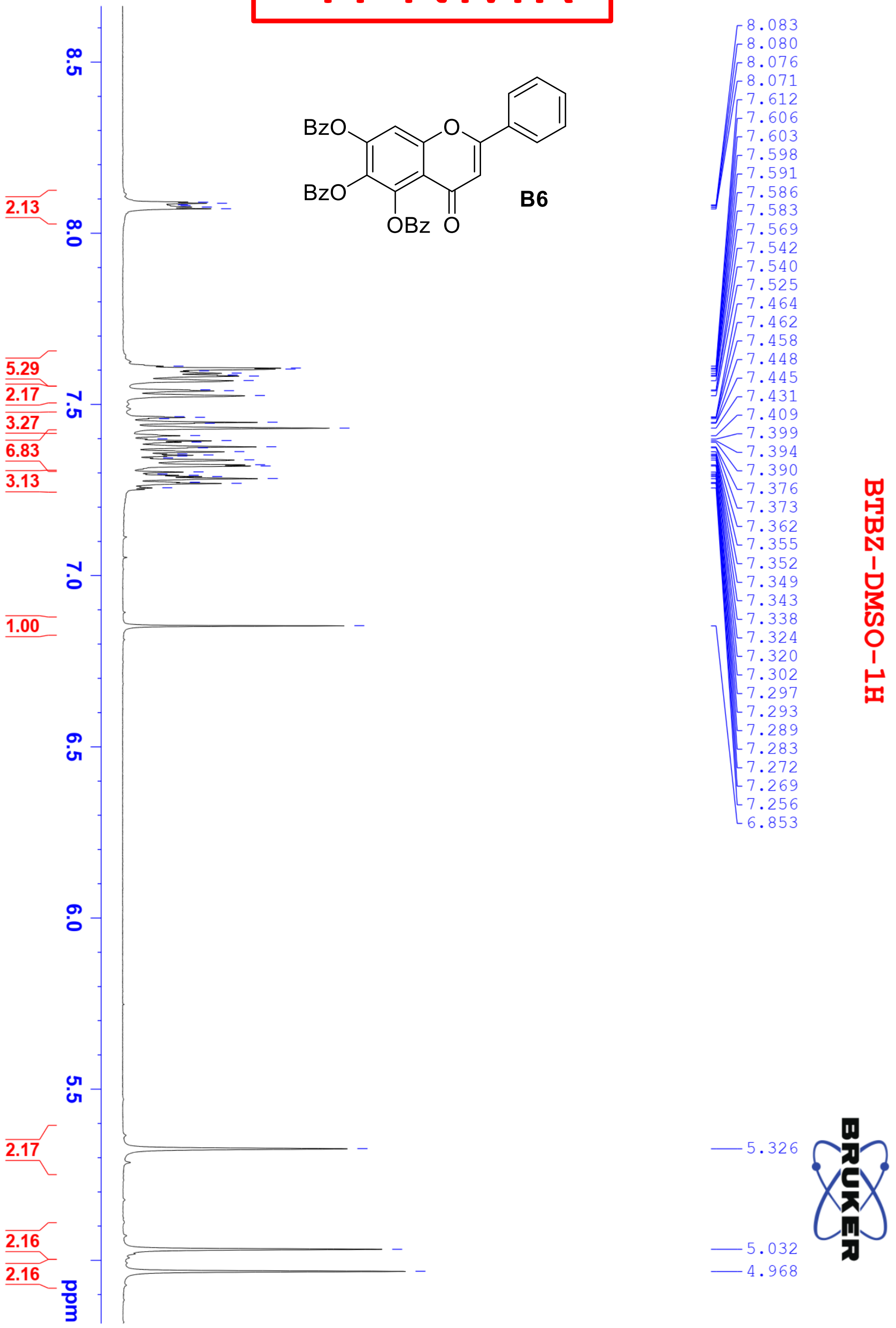

BzO
BzO
OBz
O
O
B6
8.5
8.0
7.5
7.0
6.5
6.0
5.5
ppm
2.13
5.29
2.17
3.27
6.83
3.13
1.00
2.17
2.16
2.16
8.083
8.080
8.076
8.071
7.612
7.606
7.603
7.598
7.591
7.586
7.583
7.569
7.542
7.540
7.525
7.464
7.462
7.458
7.448
7.445
7.431
7.409
7.399
7.394
7.390
7.376
7.373
7.362
7.355
7.352
7.349
7.343
7.338
7.324
7.320
7.302
7.297
7.293
7.289
7.283
7.272
7.269
7.256
6.853
5.326
5.032
4.968
BTBZ-DMSO-1H
BRUKER

^{1}H-NMR

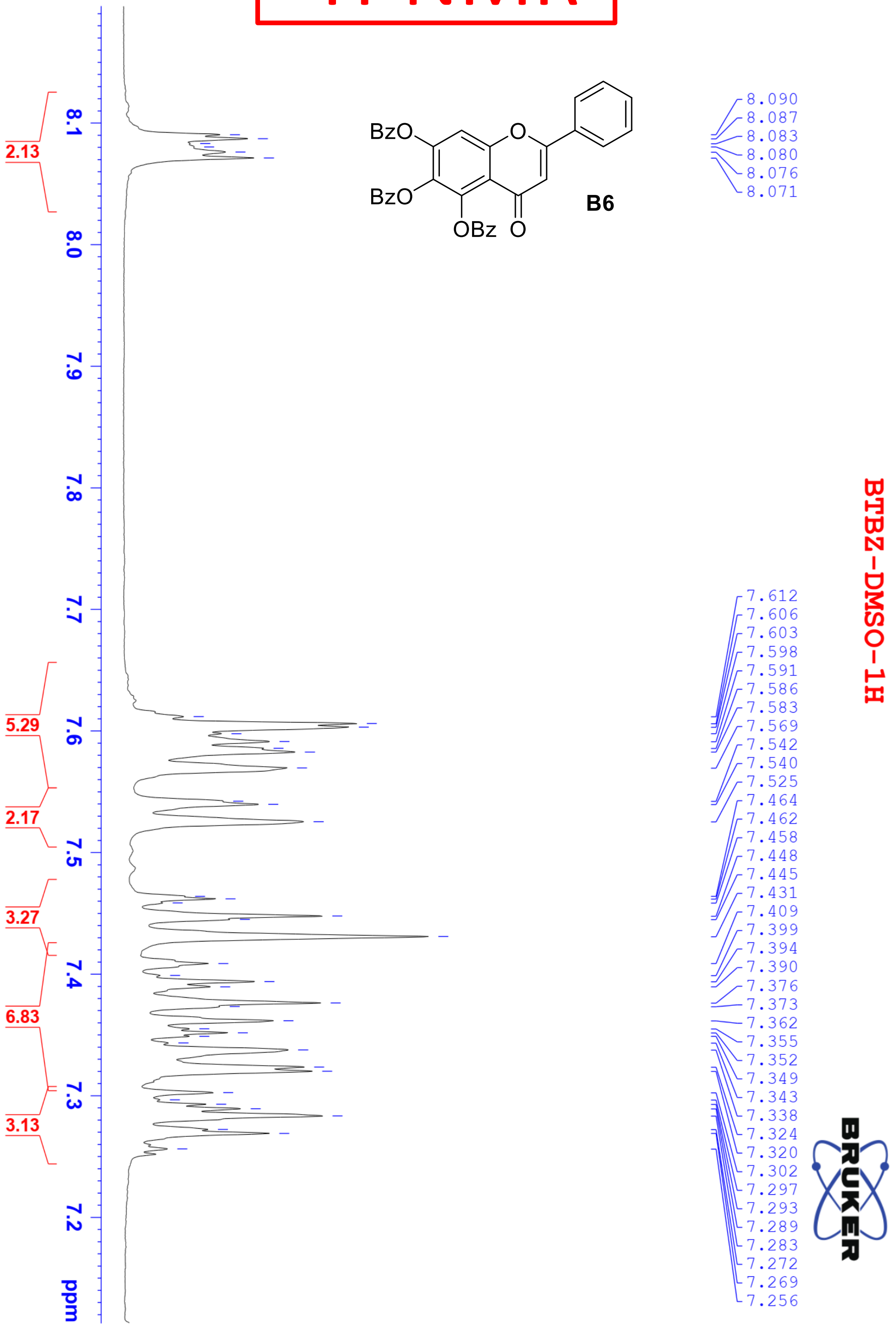

BzO
BzO
OBz
O
O
B6
8.090
8.087
8.083
8.080
8.076
8.071
7.612
7.606
7.603
7.598
7.591
7.586
7.583
7.569
7.542
7.540
7.525
7.464
7.462
7.458
7.448
7.445
7.431
7.409
7.399
7.394
7.390
7.376
7.373
7.362
7.355
7.352
7.349
7.343
7.338
7.324
7.320
7.302
7.297
7.293
7.289
7.283
7.272
7.269
7.256
BTBZ-DMSO-1H
2.13
5.29
2.17
3.27
6.83
3.13
8.1
8.0
7.9
7.8
7.7
7.6
7.5
7.4
7.3
7.2
ppm

^{13}C-NMR

BTBZ-DMSO-C13CPD

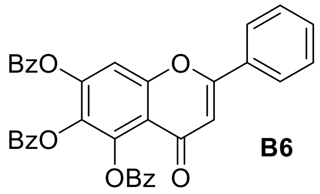

B6

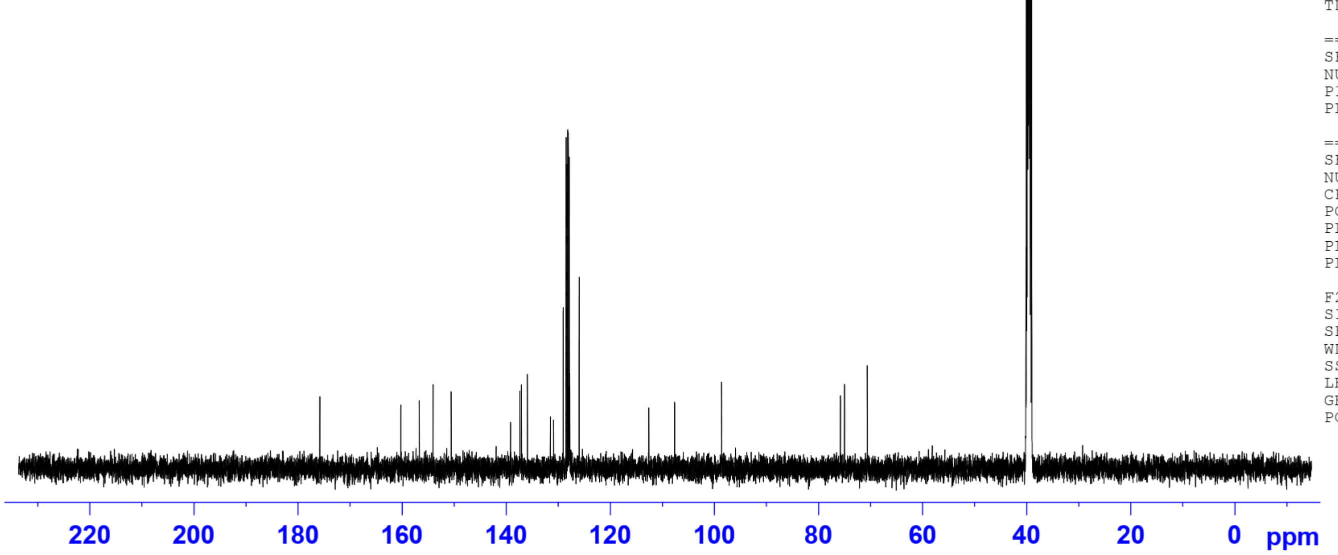

BRUKER

```
Current Data Parameters
NAME          110HUAN_BTBZ
EXPNO                    2
PROCNO                   1

F2 - Acquisition Parameters
Date_             20191126
Time                 12.04
INSTRUM              spect
PROBHD     5 mm PABBO BB/
PULPROG             zgpg30
TD                   65536
SOLVENT               DMSO
NS                    2048
DS                       4
SWH              31250.000 Hz
FIDRES            0.476837 Hz
AQ               1.0485760 sec
RG                  198.57
DW                  16.000 usec
DE                    6.50 usec
TE                   305.2 K
D1              2.00000000 sec
D11             0.03000000 sec
TD0                      1

======== CHANNEL f1 ========
SFO1           125.7864591 MHz
NUC1                   13C
P1                   10.00 usec
PLW1           88.00000000 W

======== CHANNEL f2 ========
SFO2           500.1910008 MHz
NUC2                    1H
CPDPRG[2           waltz16
PCPD2                80.00 usec
PLW2           22.00000000 W
PLW12           0.35764000 W
PLW13           0.17989001 W

F2 - Processing parameters
SI                   32768
SF             125.7726241 MHz
WDW                     EM
SSB      0
LB                    1.00 Hz
GB       0
PC                    1.40
```

^{13}C-NMR

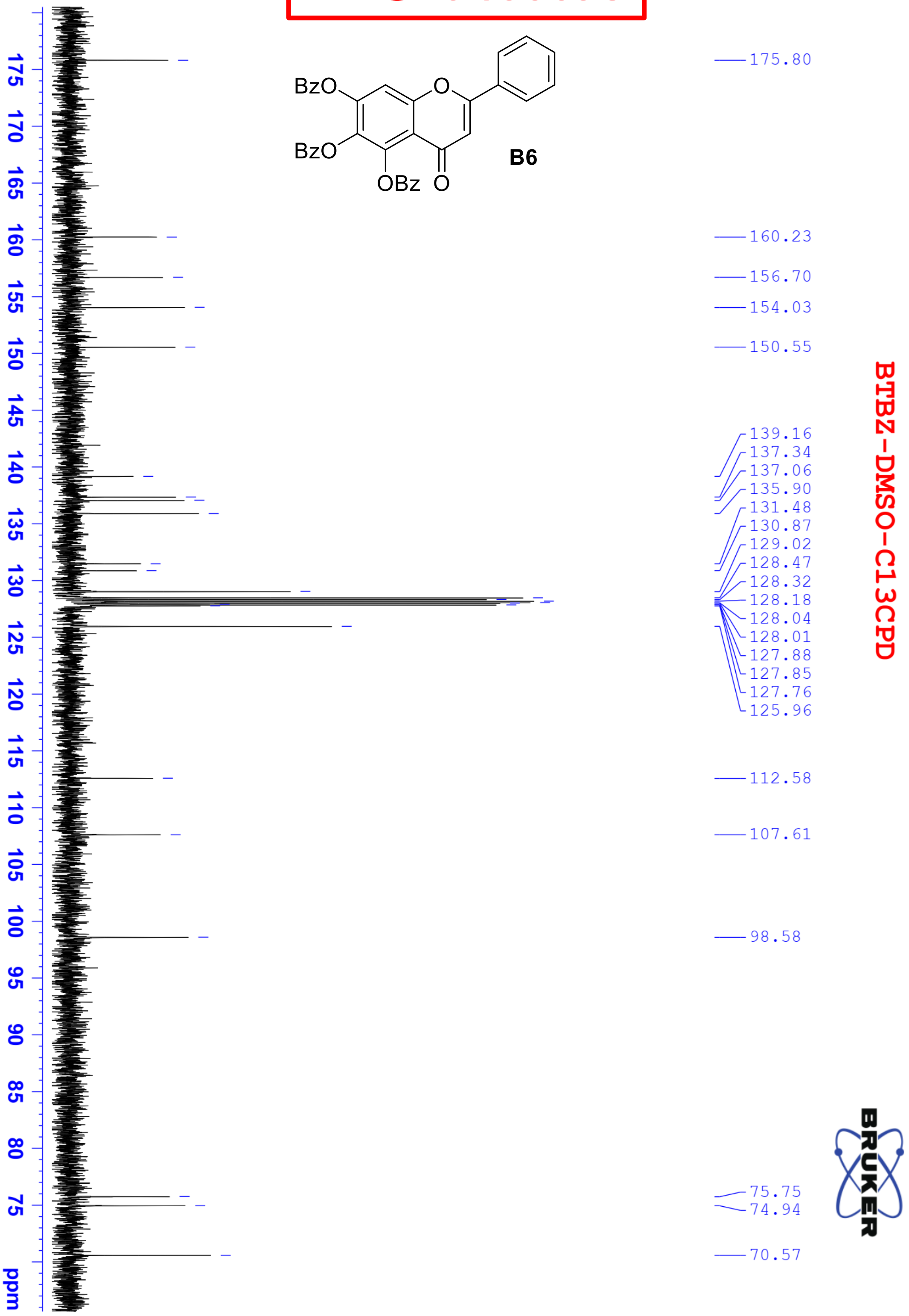

BzO
BzO
OBz
O
O
B6
175.80
160.23
156.70
154.03
150.55
139.16
137.34
137.06
135.90
131.48
130.87
129.02
128.47
128.32
128.18
128.04
128.01
127.88
127.85
127.76
125.96
112.58
107.61
98.58
75.75
74.94
70.57
BTBZ-DMSO-C13CPD
175 170 165 160 155 150 145 140 135 130 125 120 115 110 105 100 95 90 85 80 75 ppm

^{13}C-NMR

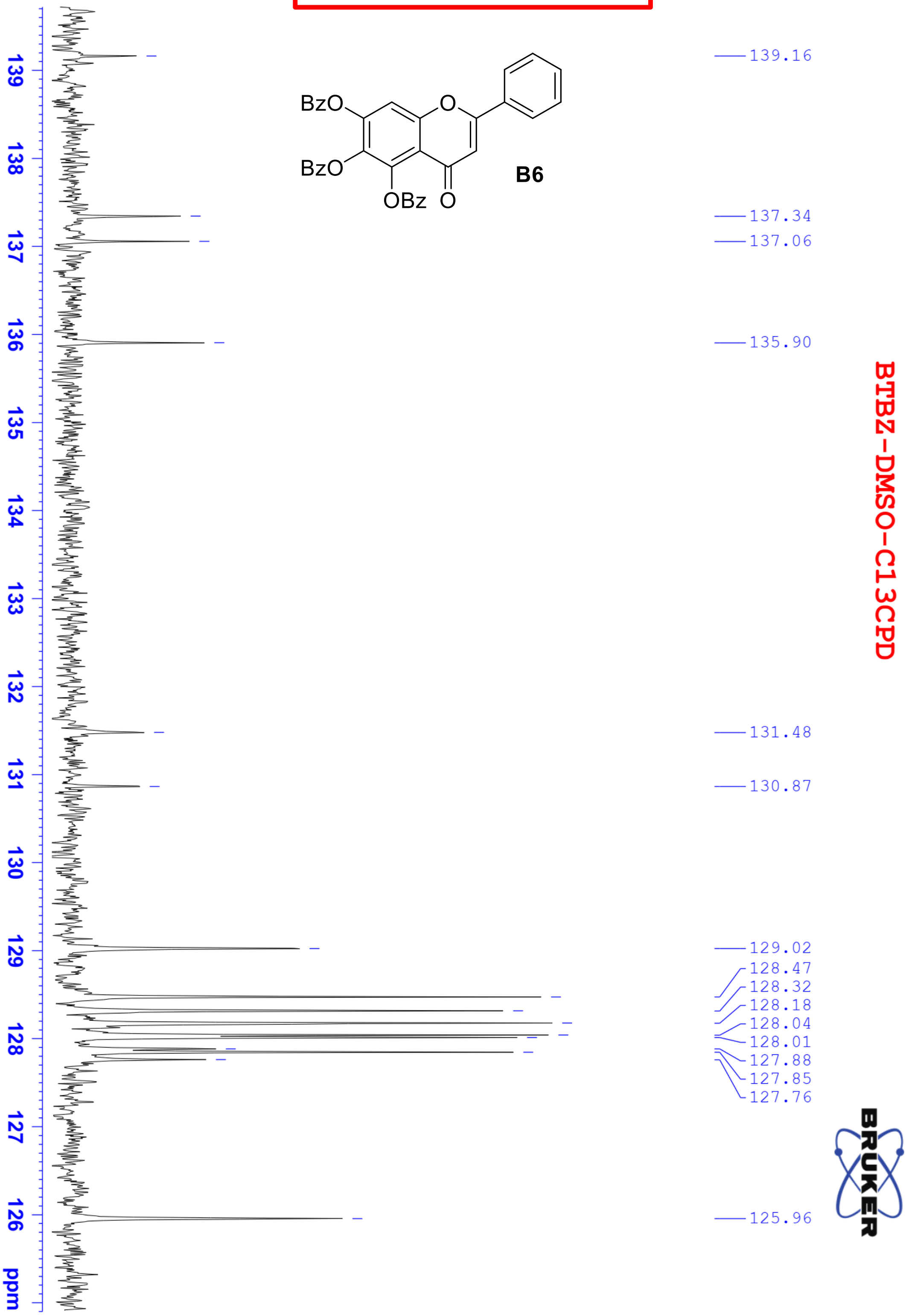

BzO
BzO
OBz
O
O
B6
139.16
137.34
137.06
135.90
131.48
130.87
129.02
128.47
128.32
128.18
128.04
128.01
127.88
127.85
127.76
125.96
139
138
137
136
135
134
133
132
131
130
129
128
127
126
ppm
BTBZ-DMSO-C13CPD
BRUKER

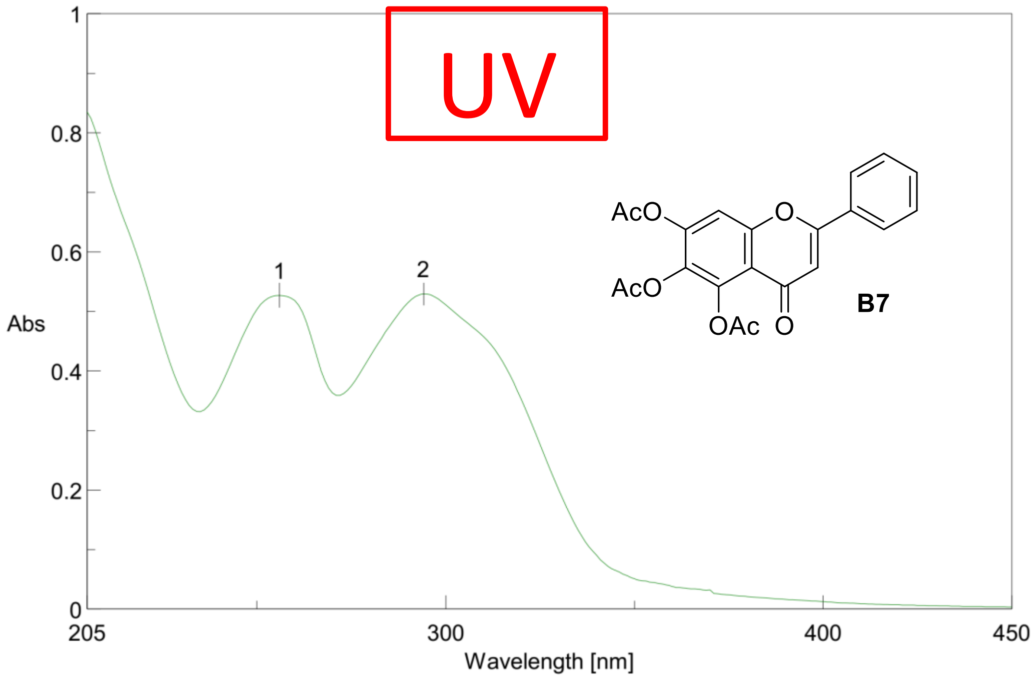

[Result of Peak Picking]

No.	Position	Intensity	No.	Position	Intensity
1	256	0.526452	2	294	0.529178

SHIMADZU

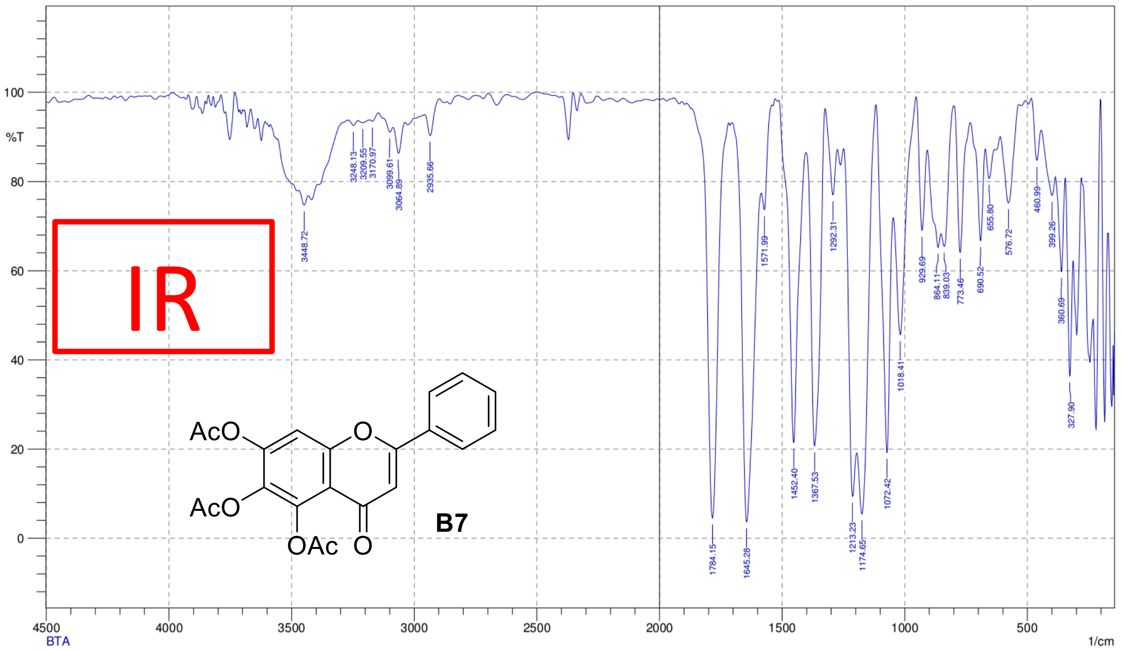

Sample name
BTA

Apodization;
Resolution;
No. of Scans;

Date/Time; 5/11/2020 2:02:26 PM
User; IR-Prestige

MS

Qualitative Analysis Report

**Data Filename**	BTA 10.d	**Sample Name**	BTA 10
**Sample Type**	Sample	**Position**	P2-B1
**Instrument Name**	Instrument 1	**User Name**	
**Acq Method**	Cot ngan - MSMS_Pos.m	**Acquired Time**	04/08/2020 7:12:04 PM
**IRM Calibration Status**	Success	**DA Method**	COTNGAN.M.m
**Comment**			

**Sample Group**		**Info.**	
**Stream Name**	LC 1	**Acquisition SW Version**	6200 series TOF/6500 series Q-TOF B.06.01 (B6172 SP1)

User Chromatograms

Fragmentor Voltage 140 **Collision Energy** 0 **Ionization Mode** ESI

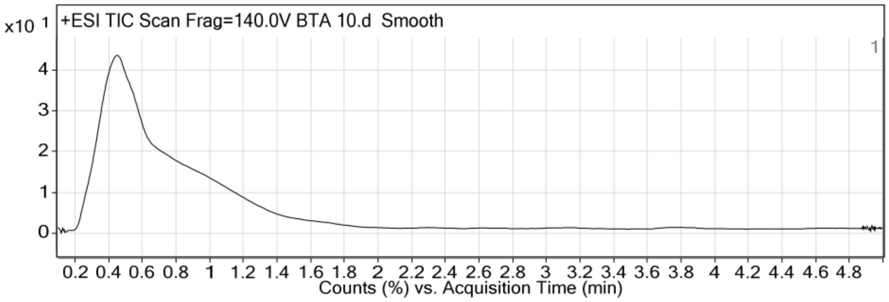

User Spectra

Fragmentor Voltage 140 **Collision Energy** 0 **Ionization Mode** ESI

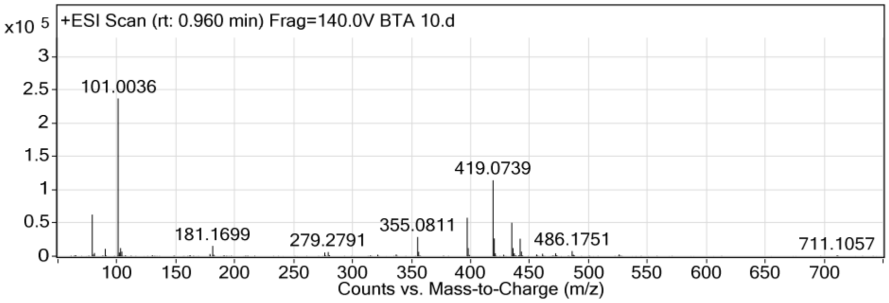

Peak List

m/z	z	Abund
79.0213	1	62825.86
101.0036	1	237001.98
397.0916	1	57811.62
419.0739	1	114020.98
435.0476	1	50508.92
815.1593	1	282822.72
816.1616	1	139684.63
831.133	1	223102.61
832.1356	1	108772.73
833.1357	1	42507.65

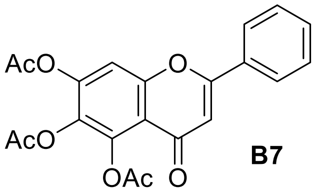

B7

--- End Of Report ---

MS

**Sample Name**	BTA 10	**Position**	P2-B1	**Instrument Name**	Instrument 1	**User Name**	
**Inj Vol**	2	**InjPosition**		**SampleType**	Sample	**IRM Calibration Status**	Success
**Data Filename**	BTA 10.d	**ACQ Method**	Cot ngan - MSMS_Pos.	**Comment**		**Acquired Time**	04/08/2020 7:12:04 PM

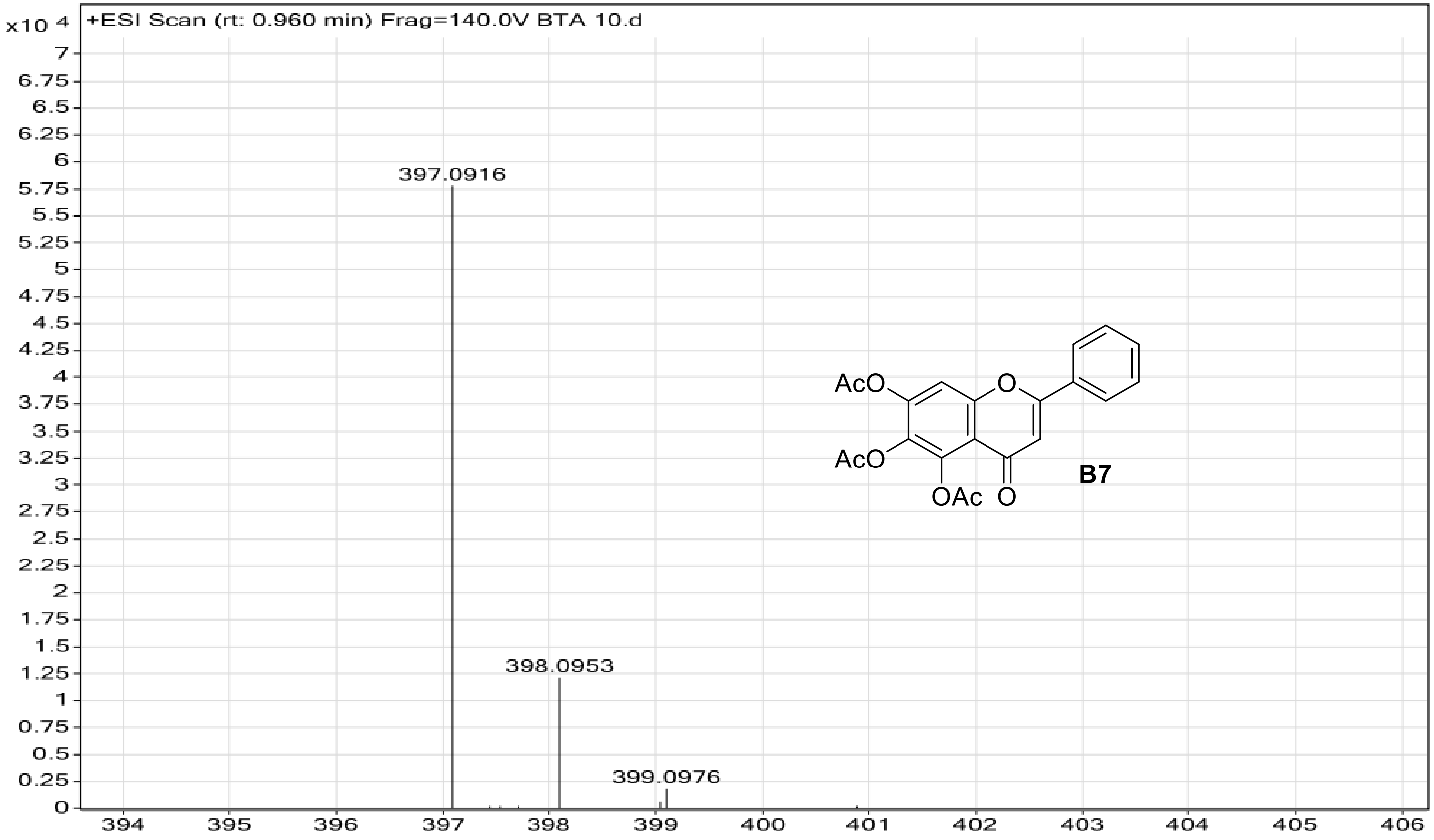

^{1}H-NMR

BTA-DMSO-1H

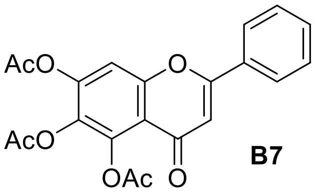

B7

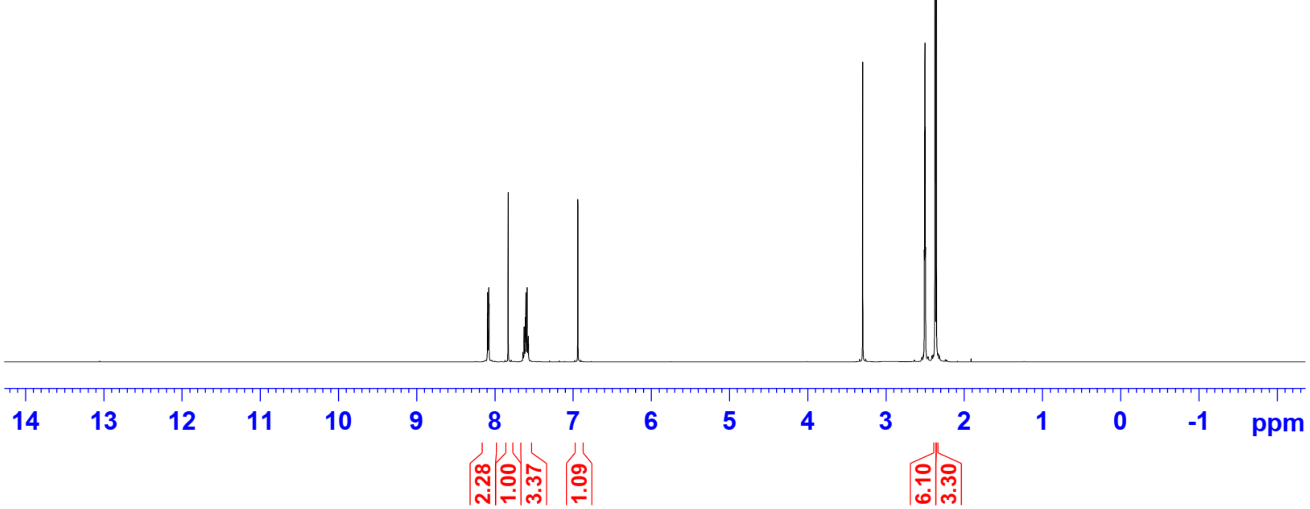


```
Current Data Parameters
NAME          113HUAN_BTA
EXPNO                  10
PROCNO                  1

F2 - Acquisition Parameters
Date_            20191008
Time                12.03
INSTRUM             spect
PROBHD   5 mm PABBO BB/
PULPROG              zg30
TD                  65536
SOLVENT              DMSO
NS                     16
DS                      2
SWH             10000.000 Hz
FIDRES           0.152588 Hz
AQ              3.2767999 sec
RG                 127.68
DW                 50.000 usec
DE                   6.50 usec
TE                  304.7 K
D1             1.00000000 sec
TD0                     1

======== CHANNEL f1 ========
SFO1          500.1920889 MHz
NUC1                   1H
P1                  10.20 usec
PLW1          22.00000000 W

F2 - Processing parameters
SI                  65536
SF            500.1890048 MHz
WDW                    EM
SSB       0
LB                   0.30 Hz
GB        0
PC                   1.00
```

^{1}H-NMR

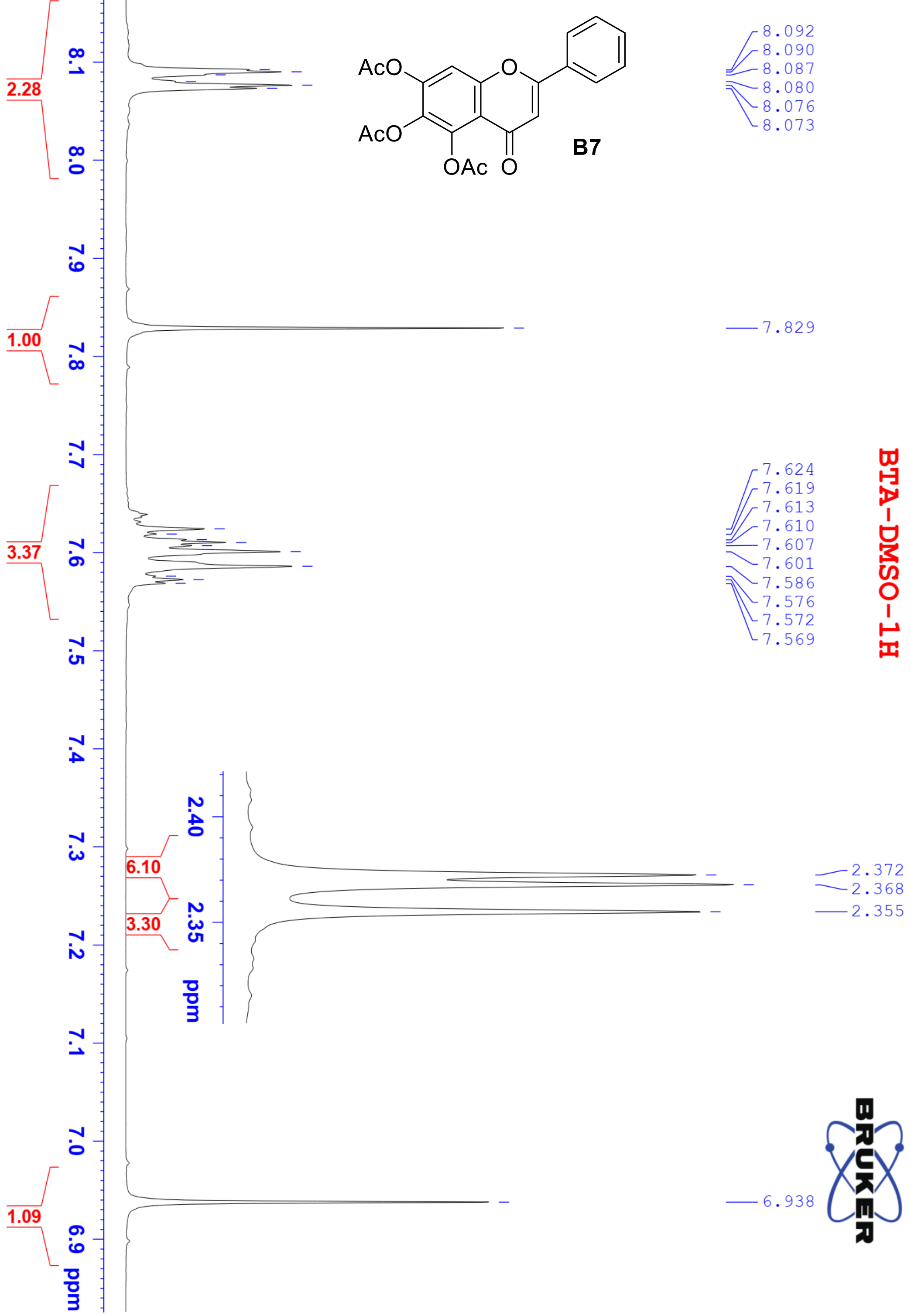

AcO
AcO
OAc
O
O
B7
8.092
8.090
8.087
8.080
8.076
8.073
7.829
7.624
7.619
7.613
7.610
7.607
7.601
7.586
7.576
7.572
7.569
2.372
2.368
2.355
BTA-DMSO-1H
2.28
1.00
3.37
6.10
3.30
1.09
8.1
8.0
7.9
7.8
7.7
7.6
7.5
7.4
7.3
7.2
7.1
7.0
6.9
ppm
2.40
2.35
ppm

^{13}C-NMR

BTA-DMSO-C13CPD

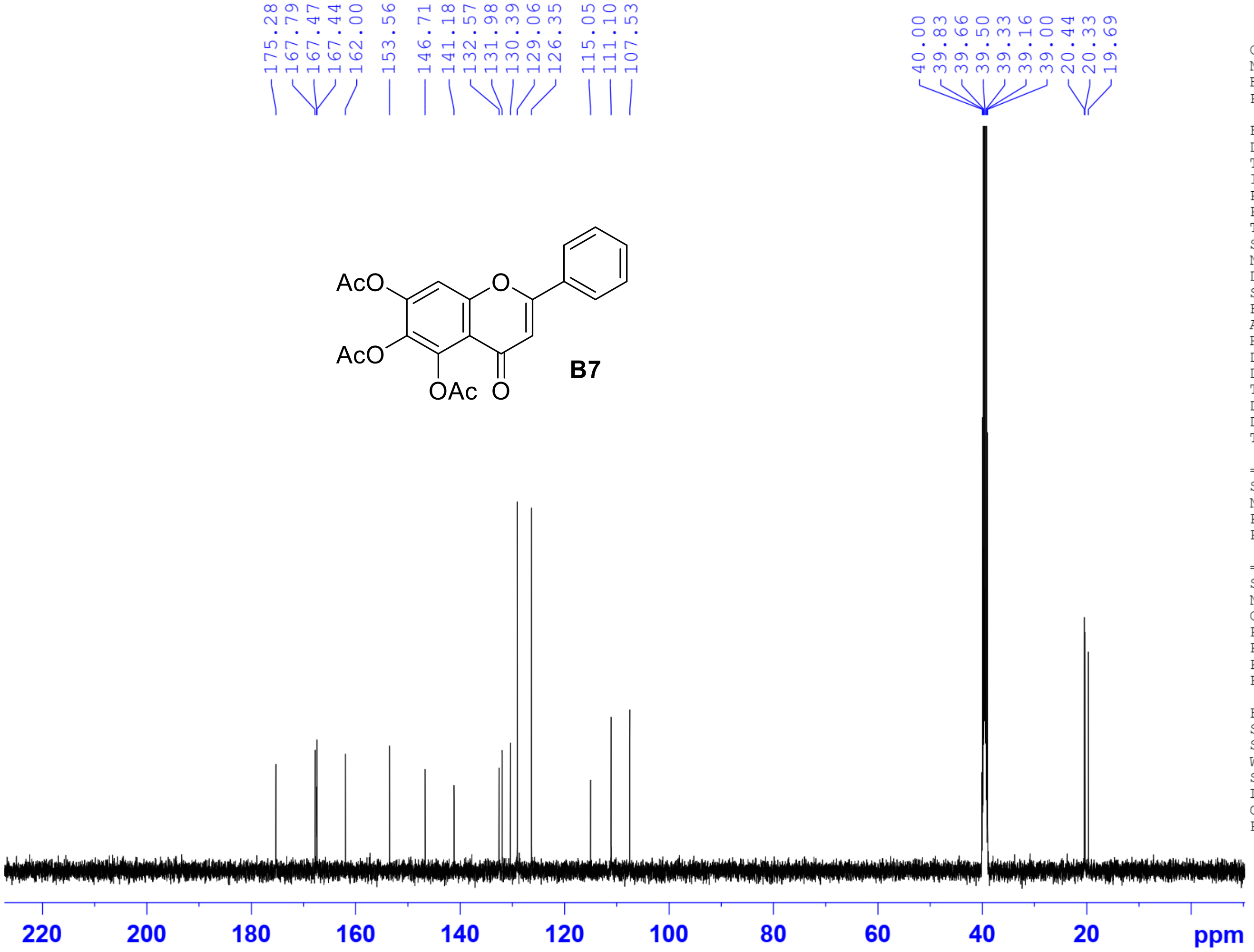


```
Current Data Parameters
NAME          113HUAN_BTA
EXPNO                   2
PROCNO                  1

F2 - Acquisition Parameters
Date_            20191008
Time                16.14
INSTRUM             spect
PROBHD   5 mm PABBO BB/
PULPROG           zgpg30
TD                  65536
SOLVENT              DMSO
NS                    256
DS                      4
SWH             31250.000 Hz
FIDRES           0.476837 Hz
AQ              1.0485760 sec
RG                 198.57
DW                 16.000 usec
DE                   6.50 usec
TE                  304.3 K
D1             2.00000000 sec
D11            0.03000000 sec
TD0                     1

======== CHANNEL f1 ========
SFO1         125.7864591 MHz
NUC1                  13C
P1                  10.00 usec
PLW1          88.00000000 W

======== CHANNEL f2 ========
SFO2         500.1910008 MHz
NUC2                   1H
CPDPRG[2          waltz16
PCPD2               80.00 usec
PLW2          22.00000000 W
PLW12          0.35764000 W
PLW13          0.17989001 W

F2 - Processing parameters
SI                  32768
SF            125.7726931 MHz
WDW                    EM
SSB      0
LB                   1.00 Hz
GB       0
PC                   1.40
```

^{13}C-NMR

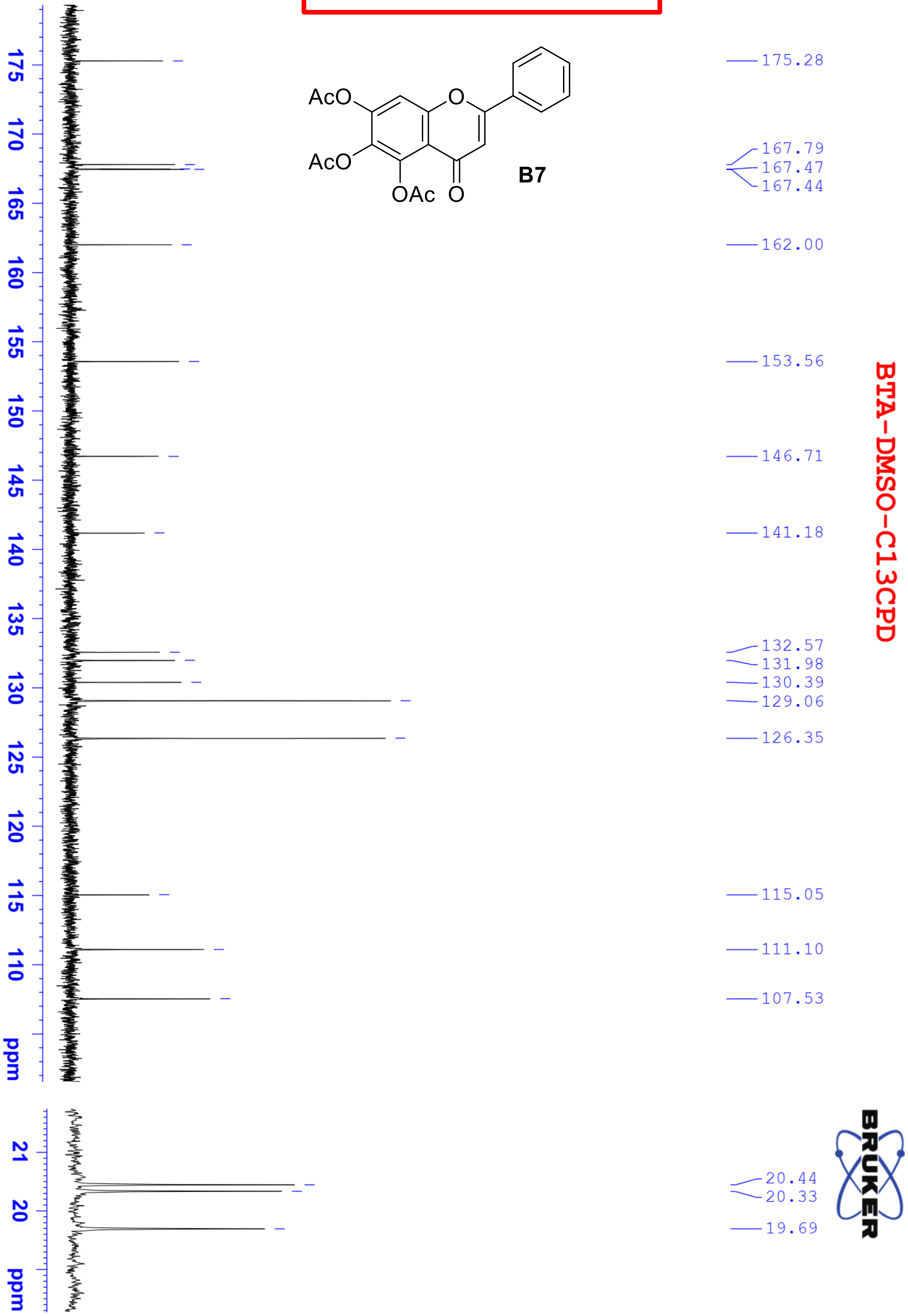

AcO
AcO
OAc
O
O
B7
175.28
167.79
167.47
167.44
162.00
153.56
146.71
141.18
132.57
131.98
130.39
129.06
126.35
115.05
111.10
107.53
20.44
20.33
19.69
BTA-DMSO-C13CPD
ppm

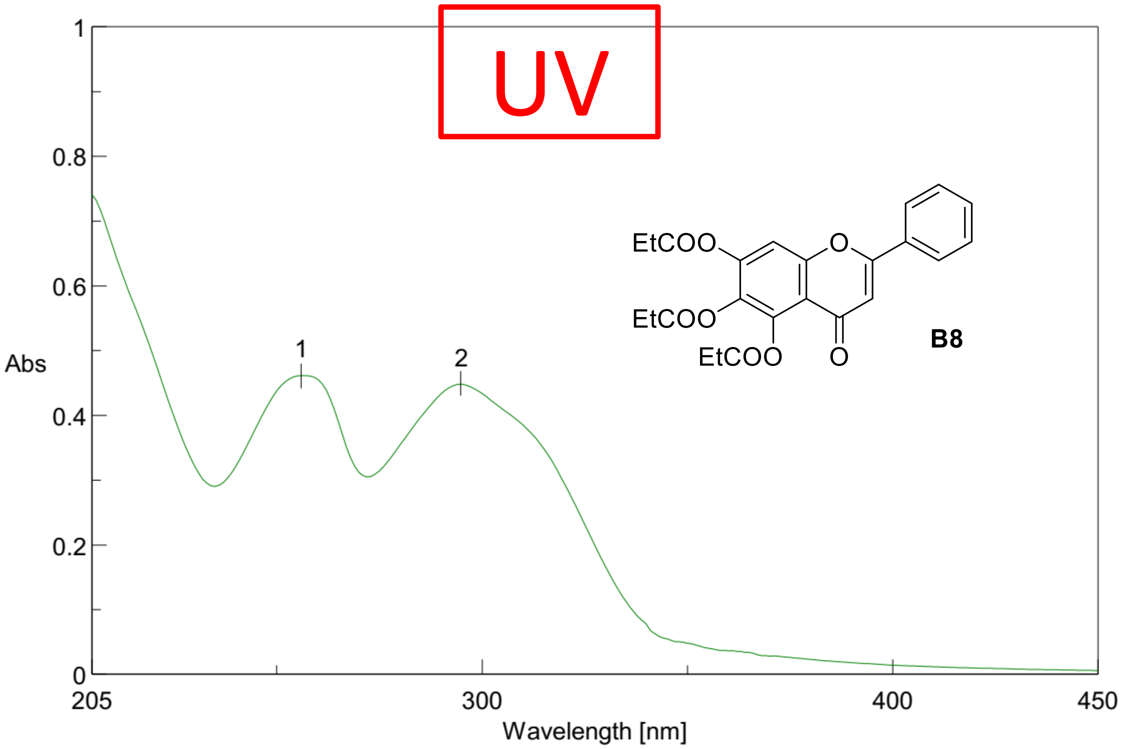

[Result of Peak Picking]

No.	Position	Intensity	No.	Position	Intensity
1	256	0.461275	2	295	0.447673

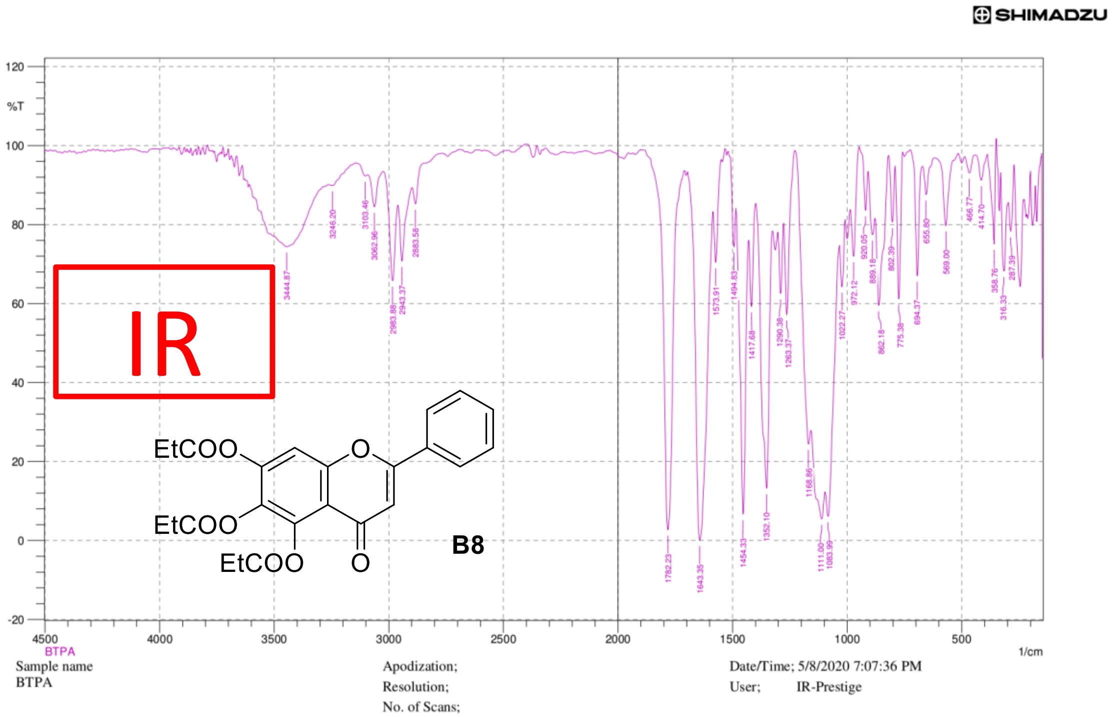

MS

Qualitative Analysis Report

**Data Filename**	BTPA-11.d	**Sample Name**	BTPA-11
**Sample Type**	Sample	**Position**	P2-B6
**Instrument Name**	Instrument 1	**User Name**	
**Acq Method**	Cot ngan - MSMS_Pos.m	**Acquired Time**	04/08/2020 7:39:54 PM
**IRM Calibration Status**	Success	**DA Method**	COTNGAN.M.m
**Comment**			

**Sample Group**		**Info.**	
**Stream Name**	LC 1	**Acquisition SW Version**	6200 series TOF/6500 series Q-TOF B.06.01 (B6172 SP1)

User Chromatograms

Fragmentor Voltage 140 **Collision Energy** 0 **Ionization Mode** ESI

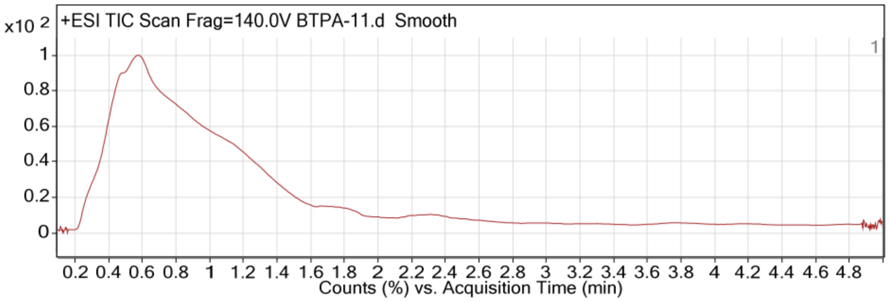

User Spectra

Fragmentor Voltage 140 **Collision Energy** 0 **Ionization Mode** ESI

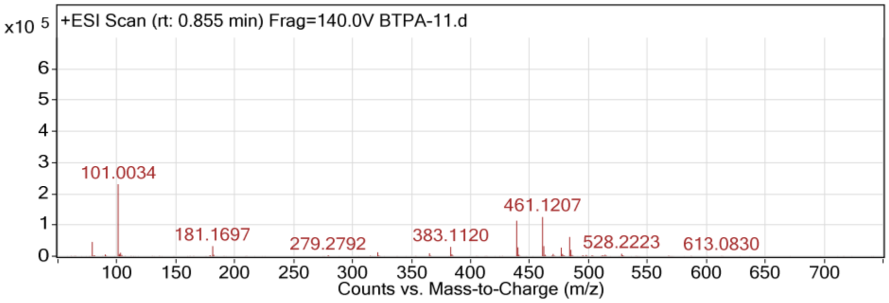

Peak List

m/z	z	Abund
101.0034	1	230474.77
439.1384	1	113750.94
461.1207	1	126043.83
484.1962	1	61900.38
899.2526	1	602931.81
900.2562	1	308889.25
901.258	1	98654.03
915.2266	1	269624.84
916.2292	1	142459.03
917.2297	1	56441.81

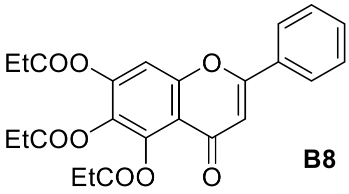

B8

--- End Of Report ---

MS

**Sample Name**	BTPA-11	**Position**	P2-B6	**Instrument Name**	Instrument 1	**User Name**	
**Inj Vol**	2	**InjPosition**		**SampleType**	Sample	**IRM Calibration Status**	Success
**Data Filename**	BTPA-11.d	**ACQ Method**	Cot ngan - MSMS_Pos.	**Comment**		**Acquired Time**	04/08/2020 7:39:54 PM

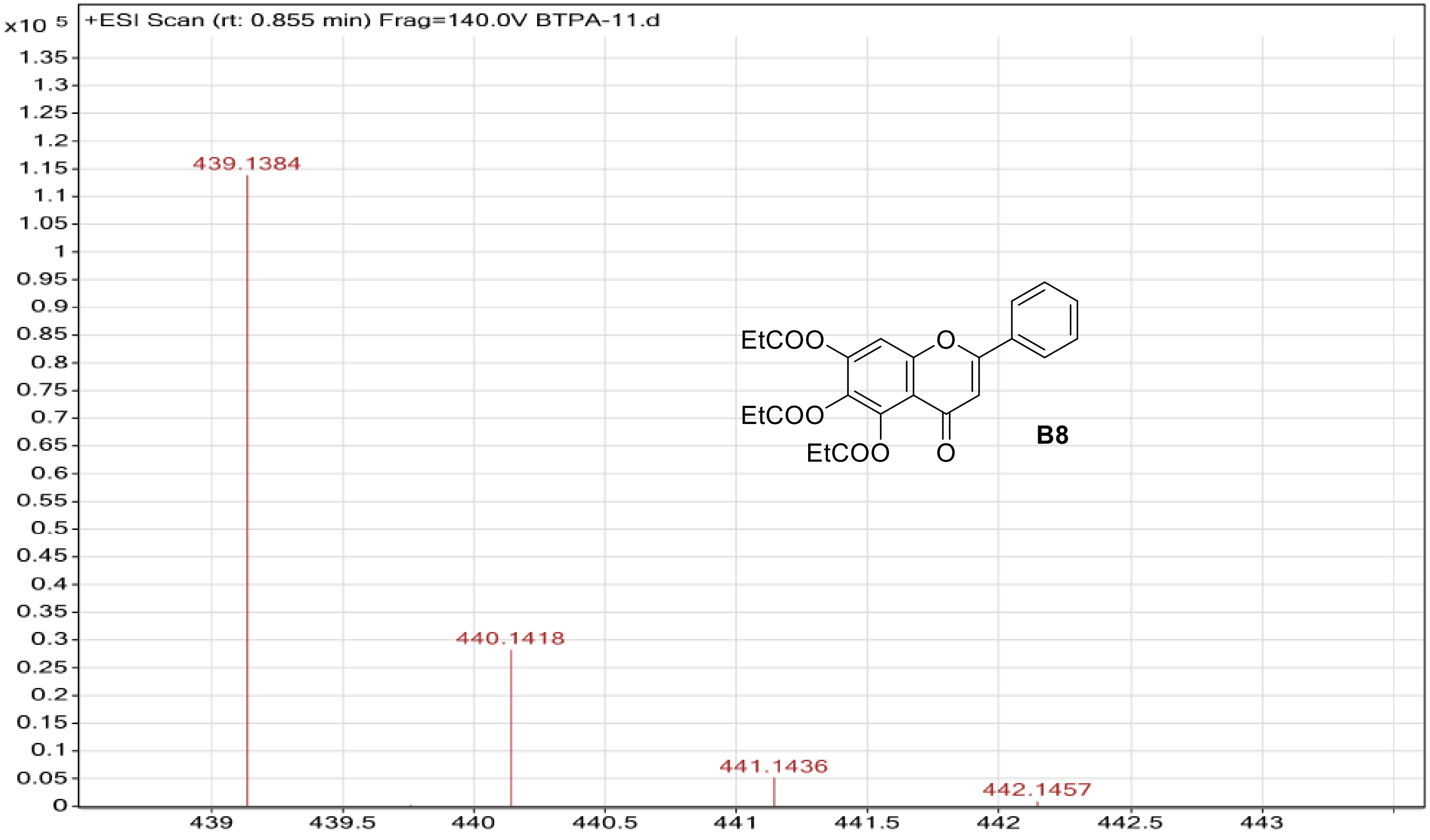

^{1}H-NMR

BTPA-DMSO-1H

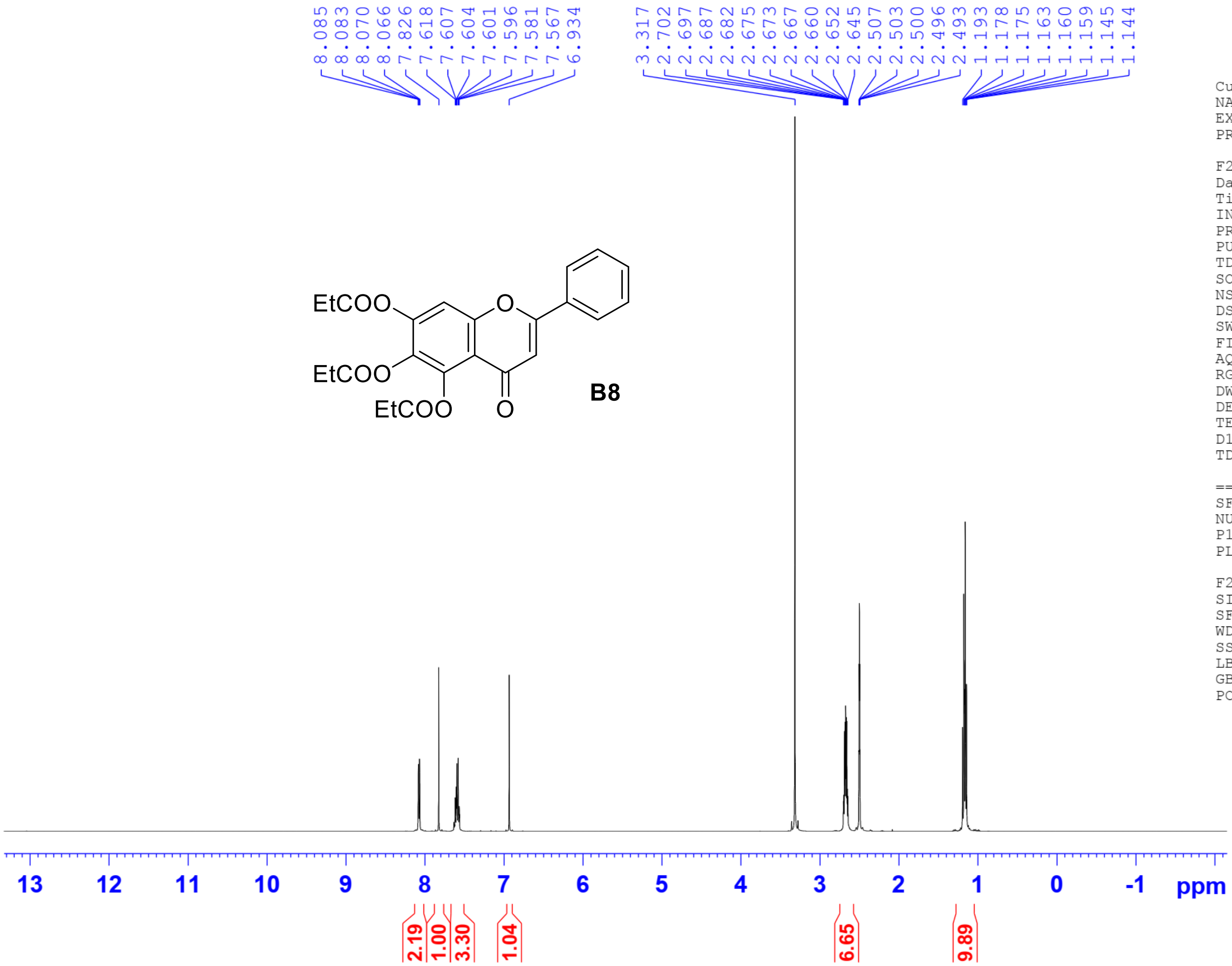

B8

```
Current Data Parameters
NAME        110HUAN_BTPA
EXPNO                 10
PROCNO                 1

F2 - Acquisition Parameters
Date_           20200109
Time               14.49
INSTRUM            spect
PROBHD   5 mm PABBO BB/
PULPROG             zg30
TD                 65536
SOLVENT             DMSO
NS                    16
DS                     2
SWH            10000.000 Hz
FIDRES          0.152588 Hz
AQ             3.2767999 sec
RG                 89.63
DW                50.000 usec
DE                  6.50 usec
TE                 303.0 K
D1            1.00000000 sec
TD0                    1

======== CHANNEL f1 ========
SFO1         500.1920889 MHz
NUC1                  1H
P1                 10.20 usec
PLW1         22.00000000 W

F2 - Processing parameters
SI                 65536
SF           500.1890047 MHz
WDW                   EM
SSB       0
LB                  0.30 Hz
GB        0
PC                  1.00
```

^{1}H-NMR

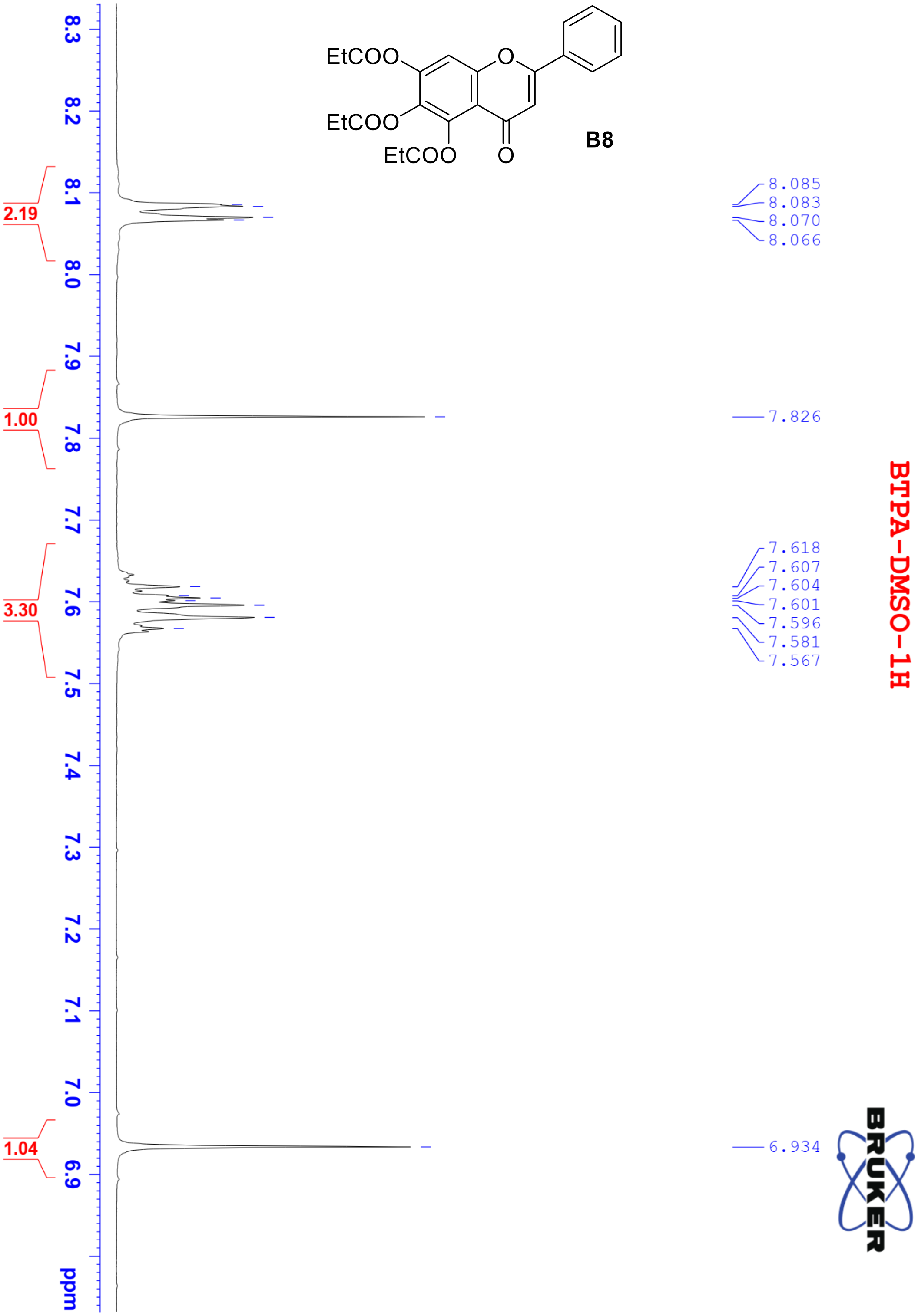

EtCOO
EtCOO
EtCOO
O
O
B8
8.085
8.083
8.070
8.066
7.826
7.618
7.607
7.604
7.601
7.596
7.581
7.567
6.934
2.19
1.00
3.30
1.04
8.3
8.2
8.1
8.0
7.9
7.8
7.7
7.6
7.5
7.4
7.3
7.2
7.1
7.0
6.9
ppm
BTPA-DMSO-1H

^{1}H-NMR

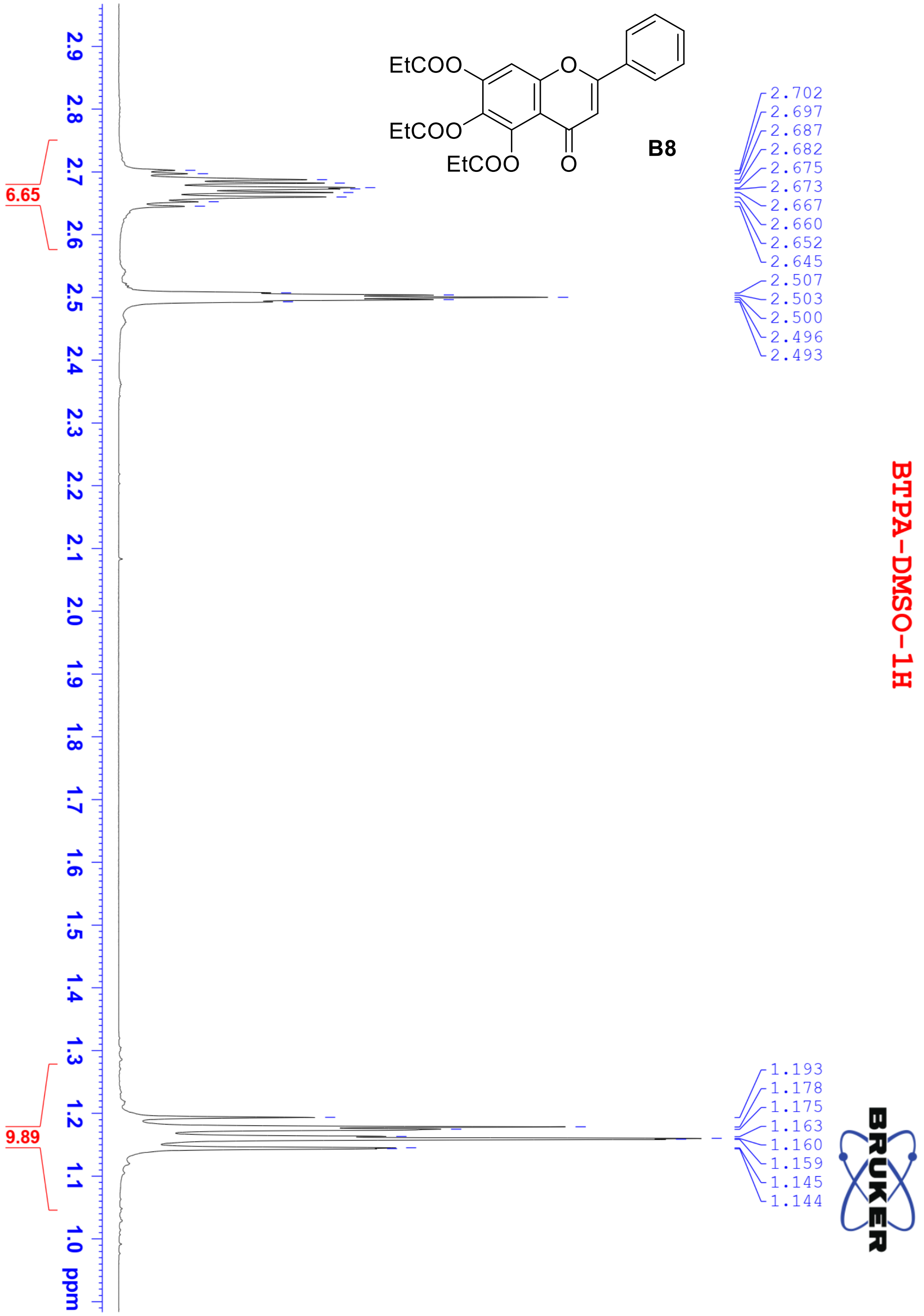

EtCOO
EtCOO
EtCOO
O
O
B8
BTPA-DMSO-1H
2.702
2.697
2.687
2.682
2.675
2.673
2.667
2.660
2.652
2.645
2.507
2.503
2.500
2.496
2.493
1.193
1.178
1.175
1.163
1.160
1.159
1.145
1.144
6.65
9.89
2.9
2.8
2.7
2.6
2.5
2.4
2.3
2.2
2.1
2.0
1.9
1.8
1.7
1.6
1.5
1.4
1.3
1.2
1.1
1.0
ppm

^{13}C-NMR

BTPA-DMSO-C13CPD

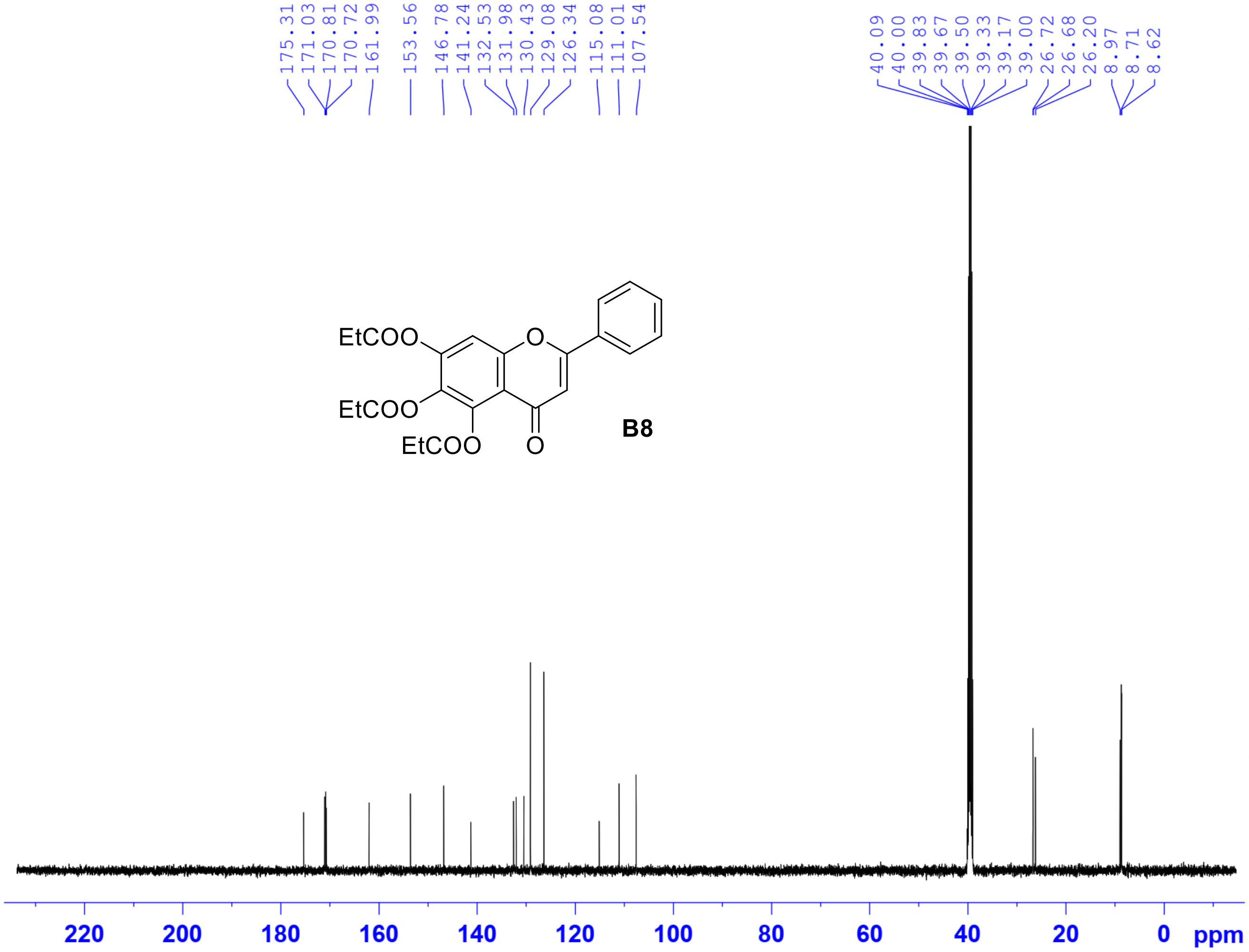

B8

```
Current Data Parameters
NAME        110HUAN_BTPA
EXPNO                  2
PROCNO                 1

F2 - Acquisition Parameters
Date_           20200109
Time               17.18
INSTRUM            spect
PROBHD   5 mm PABBO BB/
PULPROG          zgpg30
TD                 65536
SOLVENT             DMSO
NS                   256
DS                     4
SWH            31250.000 Hz
FIDRES          0.476837 Hz
AQ             1.0485760 sec
RG                198.57
DW                16.000 usec
DE                  6.50 usec
TE                 303.4 K
D1            2.00000000 sec
D11           0.03000000 sec
TD0                    1

======== CHANNEL f1 ========
SFO1         125.7864591 MHz
NUC1                 13C
P1                 10.00 usec
PLW1         88.00000000 W

======== CHANNEL f2 ========
SFO2         500.1910008 MHz
NUC2                  1H
CPDPRG[2         waltz16
PCPD2              80.00 usec
PLW2         22.00000000 W
PLW12         0.35764000 W
PLW13         0.17989001 W

F2 - Processing parameters
SI                 32768
SF           125.7726903 MHz
WDW                   EM
SSB      0
LB                  1.00 Hz
GB       0
PC                  1.40
```

^{13}C-NMR

BTPA-DMSO-C13CPD

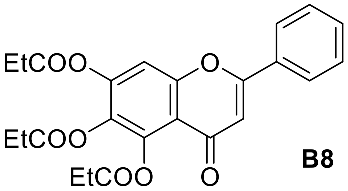

B8

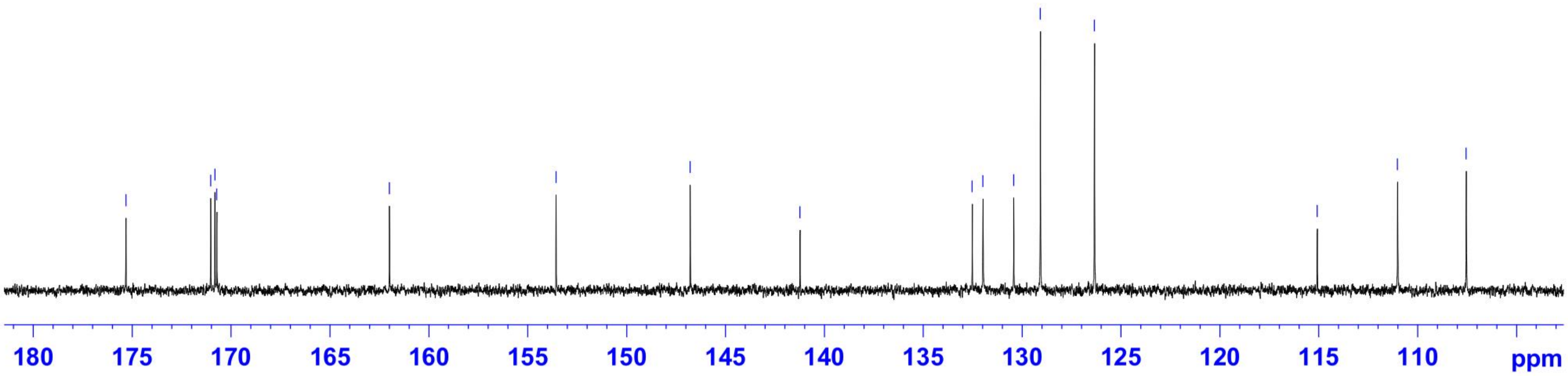

^{13}C-NMR

BTPA-DMSO-C13CPD

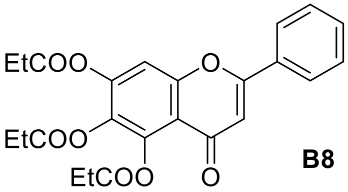

B8

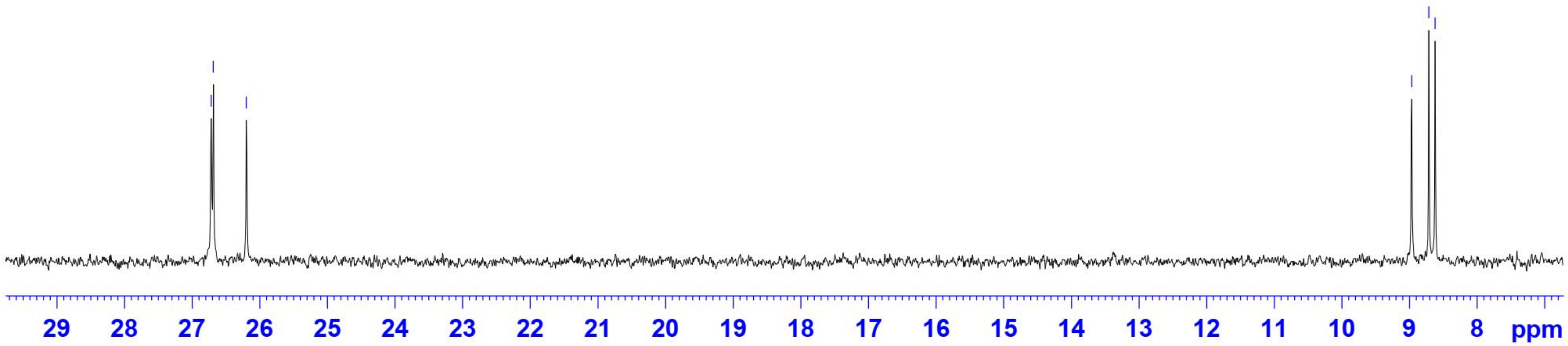

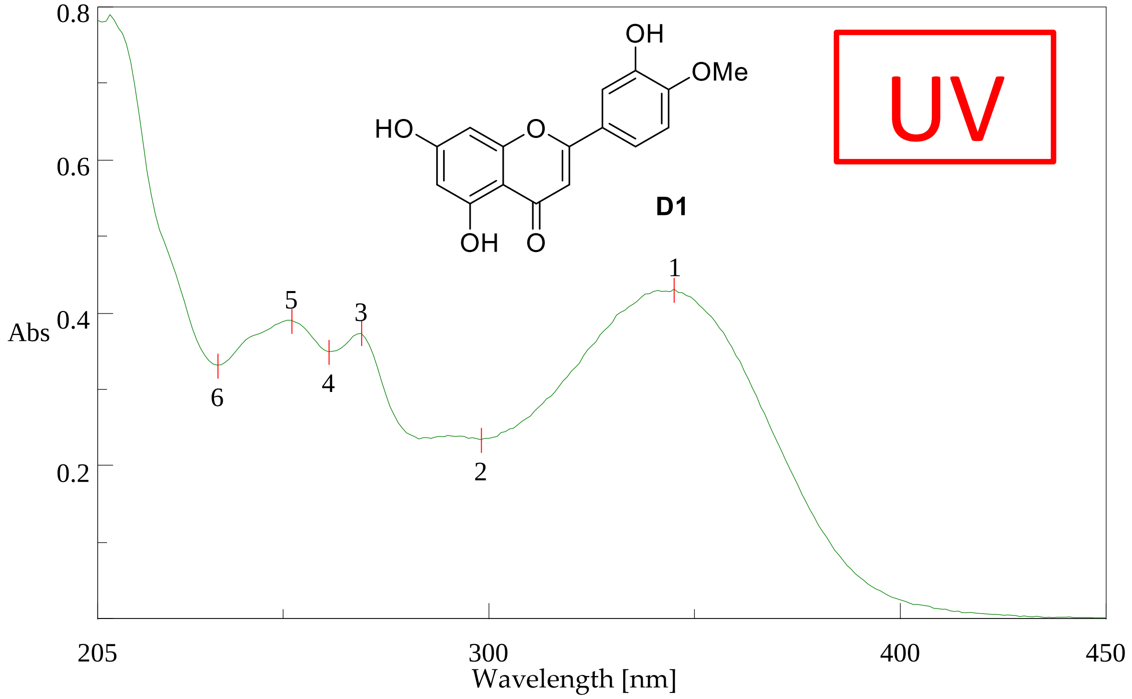

[Result of Peak Picking]

No.	Position	Intensity	No.	Position	Intensity	No.	Position	Intensity
1	345	0.43004	2	298	0.233573	3	269	0.372553
4	261	0.348546	5	252	0.389723	6	234	0.330926

VIEN CONG NGHE HOA HOC
01 Mac Dinh Chi - Q.1 - Tp HCM

Tel: 08.38296127
Fax: 08.38293889

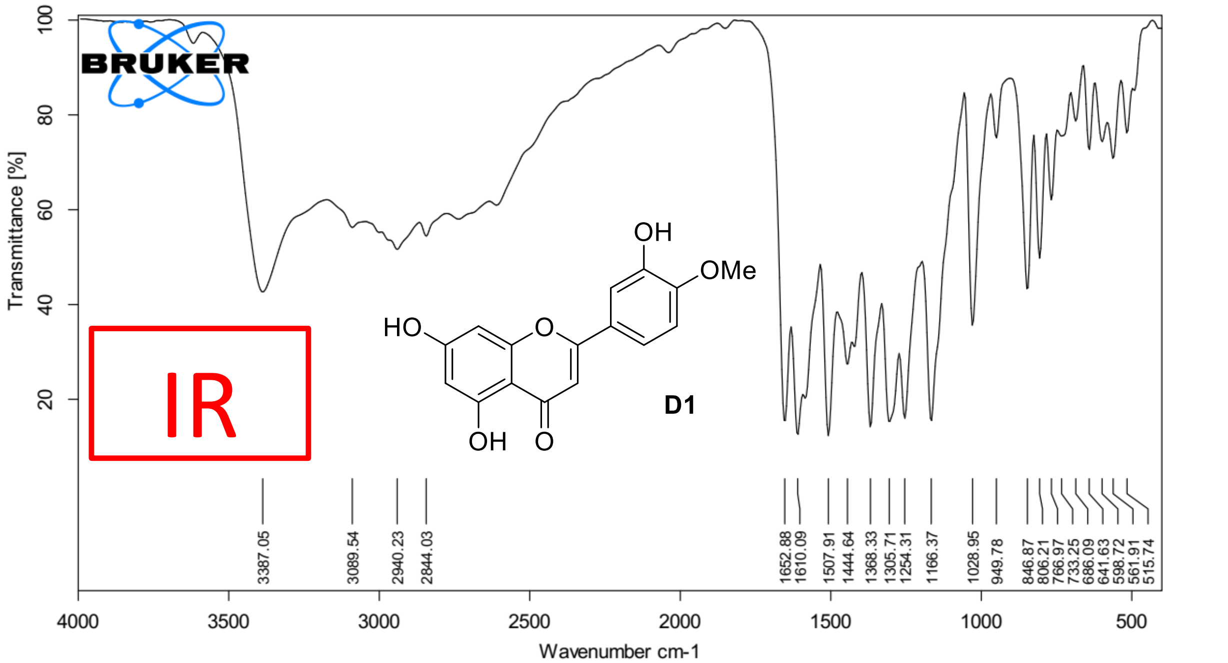

E:\OPUS 7\2016\20160812\HD1.0	HD1	TENSOR 27 - BRUKER - GERMANY	8/12/2016

MS

Qualitative Analysis Report

**Data Filename**	1-DT.d	**Sample Name**	1-DT
**Sample Type**	Sample	**Position**	P2-C6
**Instrument Name**	Instrument 1	**User Name**	
**Acq Method**	Cot ngan - MSMS_Pos.m	**Acquired Time**	04/08/2020 8:29:59 PM
**IRM Calibration Status**	Success	**DA Method**	COTNGAN.M.m
**Comment**			

**Sample Group**		**Info.**	
**Stream Name**	LC 1	**Acquisition SW Version**	6200 series TOF/6500 series Q-TOF B.06.01 (B6172 SP1)

User Chromatograms

Fragmentor Voltage 140 **Collision Energy** 0 **Ionization Mode** ESI

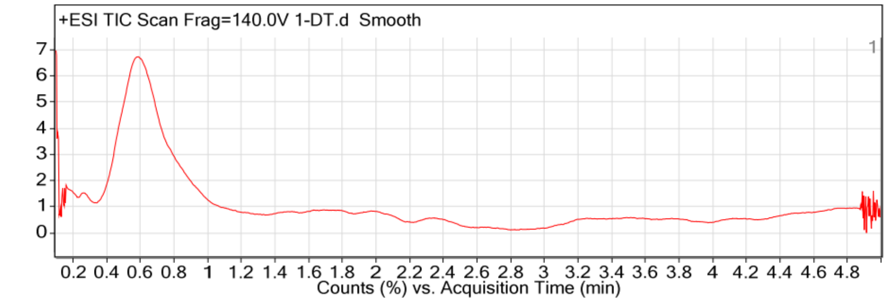

User Spectra

Fragmentor Voltage	Collision Energy	Ionization Mode
140	0	ESI

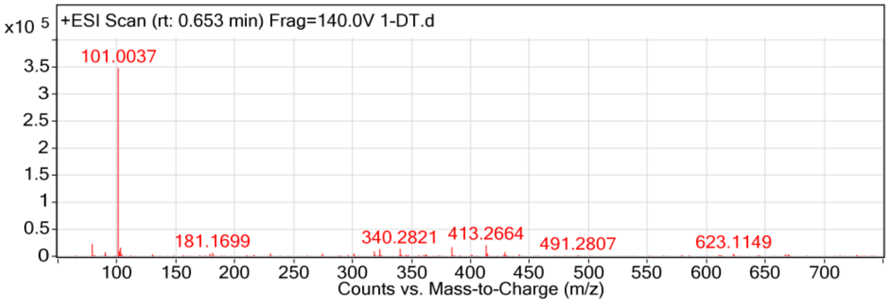

Peak List

m/z	z	Abund
79.0214	1	22762.52
101.0037	1	348713.09
102.0062	1	9852.9
102.9991	1	16236.17
318.3002	1	9053.3
323.0526	1	13376.74
340.2821	1	13906.29
384.3083	1	16981.61
413.2664	1	20418.92
429.2402	1	7832.86

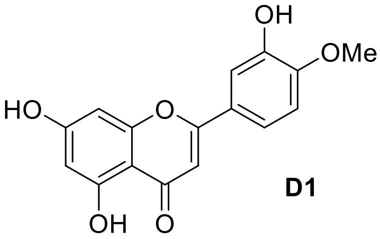

D1

--- End Of Report ---

MS

Sample Name	1-DT	Position	P2-C6	Instrument Name	Instrument 1	User Name	
Inj Vol	2	InjPosition		SampleType	Sample	IRM Calibration Status	Success
Data Filename	1-DT.d	ACQ Method	Cot ngan - MSMS_Pos.	Comment		Acquired Time	04/08/2020 8:29:59 PM

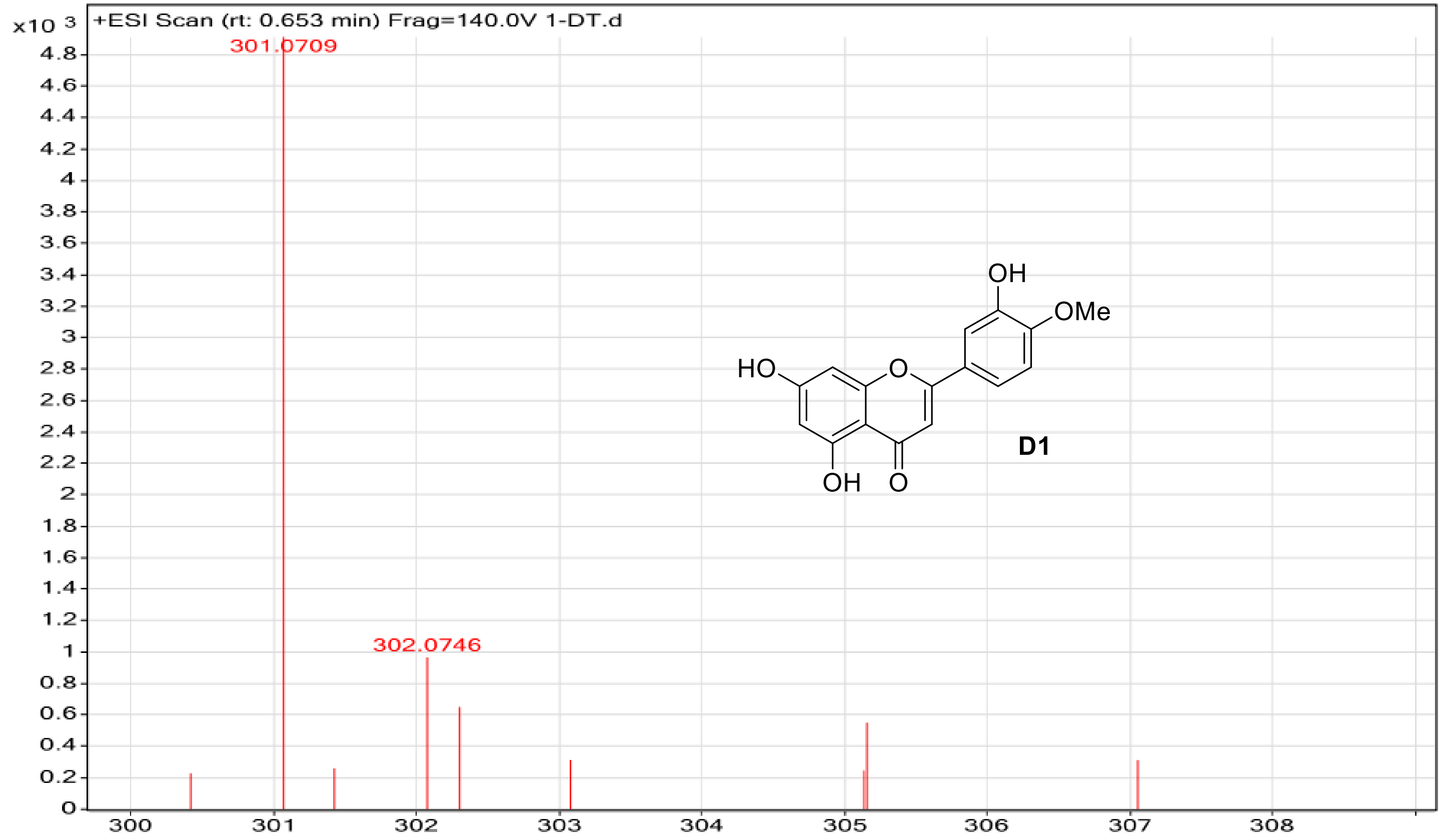

^{1}H-NMR

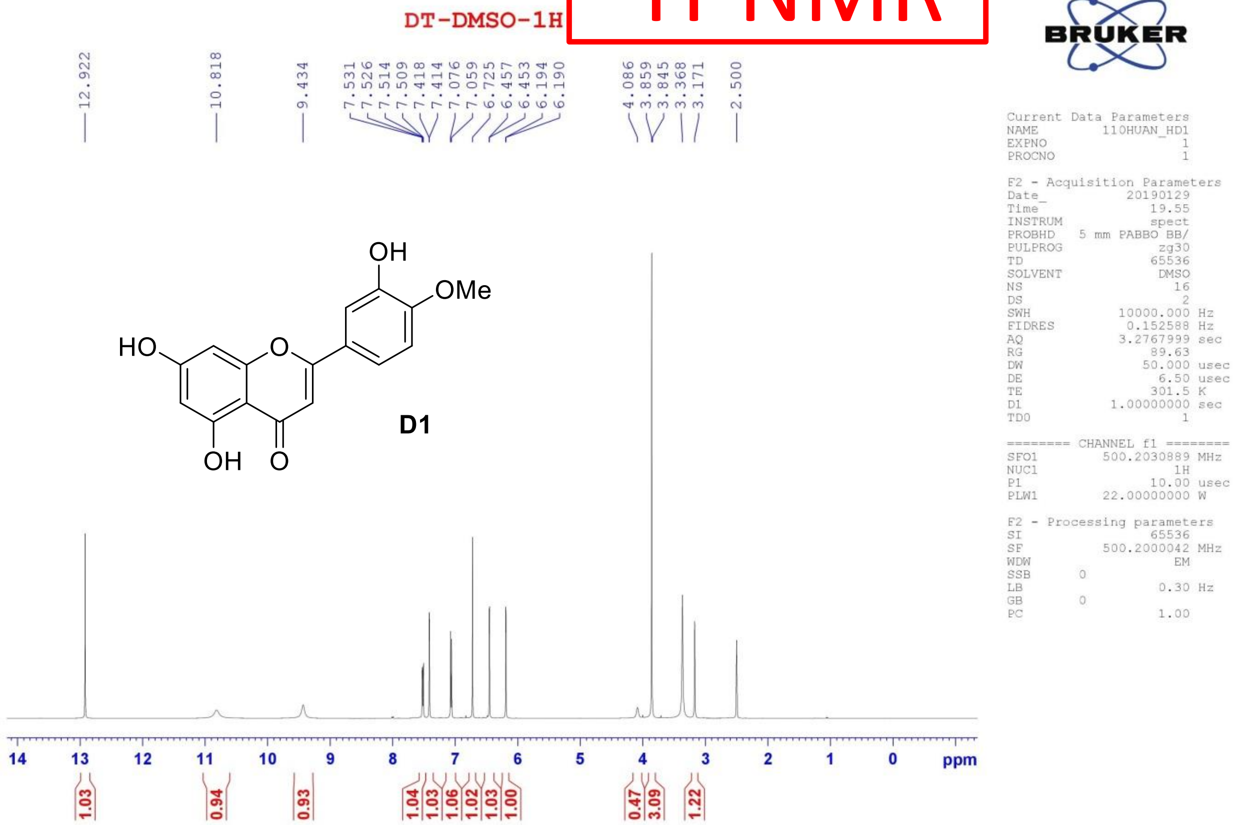
DT-DMSO-1H
12.922
10.818
9.434
7.531
7.526
7.514
7.509
7.418
7.414
7.076
7.059
6.725
6.457
6.453
6.194
6.190
4.086
3.859
3.845
3.368
3.171
2.500
OH
OMe
HO
O
OH O
D1
ppm
1.03
0.94
0.93
1.04
1.03
1.06
1.02
1.03
1.00
0.47
3.09
1.22
Current Data Parameters
NAME 110HUAN_HD1
EXPNO 1
PROCNO 1
F2 - Acquisition Parameters
Date_ 20190129
Time 19.55
INSTRUM spect
PROBHD 5 mm PABBO BB/
PULPROG zg30
TD 65536
SOLVENT DMSO
NS 16
DS 2
SWH 10000.000 Hz
FIDRES 0.152588 Hz
AQ 3.2767999 sec
RG 89.63
DW 50.000 usec
DE 6.50 usec
TE 301.5 K
D1 1.00000000 sec
TD0 1
======== CHANNEL f1 ========
SFO1 500.2030889 MHz
NUC1 1H
P1 10.00 usec
PLW1 22.00000000 W
F2 - Processing parameters
SI 65536
SF 500.2000042 MHz
WDW EM
SSB 0
LB 0.30 Hz
GB 0
PC 1.00

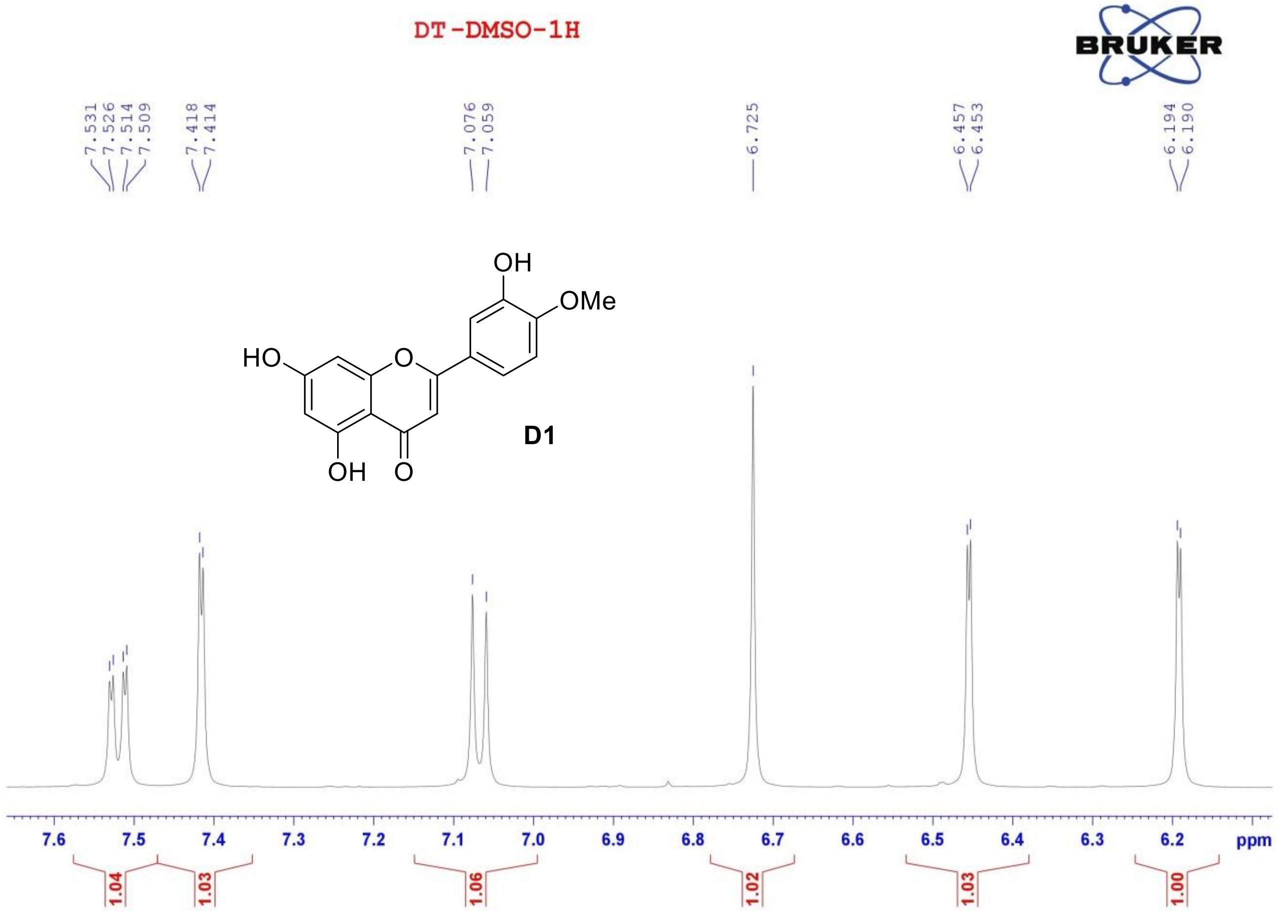
DT-DMSO-1H
7.531
7.526
7.514
7.509
7.418
7.414
7.076
7.059
6.725
6.457
6.453
6.194
6.190
OH
OMe
HO
O
OH O
D1
ppm
1.04
1.03
1.06
1.02
1.03
1.00

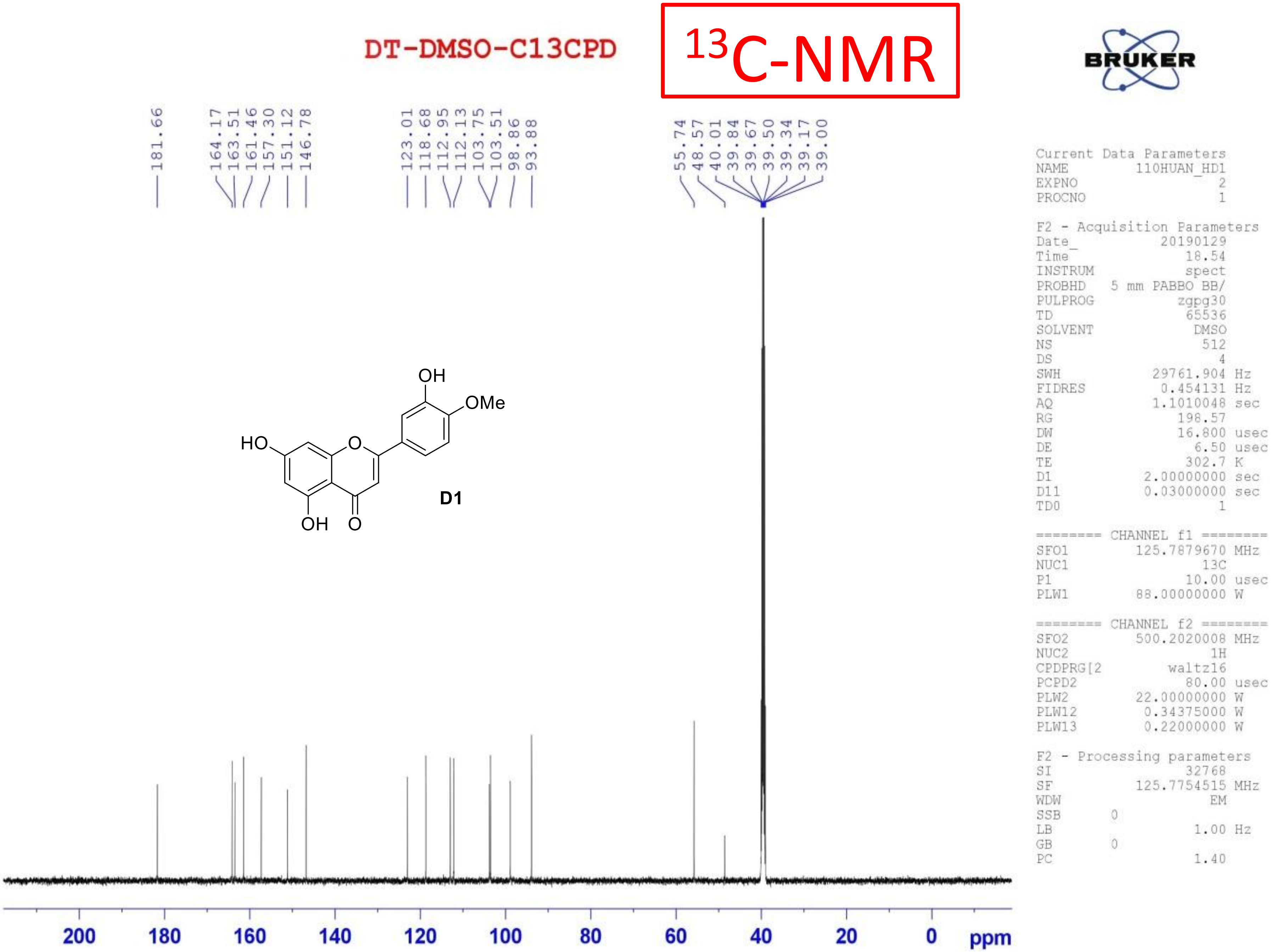
DT-DMSO-C13CPD
13C-NMR
BRUKER
181.66
164.17
163.51
161.46
157.30
151.12
146.78
123.01
118.68
112.95
112.13
103.75
103.51
98.86
93.88
55.74
48.57
40.01
39.84
39.67
39.50
39.34
39.17
39.00
OH
OMe
HO
O
OH
O
D1
Current Data Parameters
NAME 110HUAN_HD1
EXPNO 2
PROCNO 1
F2 - Acquisition Parameters
Date_ 20190129
Time 18.54
INSTRUM spect
PROBHD 5 mm PABBO BB/
PULPROG zgpg30
TD 65536
SOLVENT DMSO
NS 512
DS 4
SWH 29761.904 Hz
FIDRES 0.454131 Hz
AQ 1.1010048 sec
RG 198.57
DW 16.800 usec
DE 6.50 usec
TE 302.7 K
D1 2.00000000 sec
D11 0.03000000 sec
TD0 1
======== CHANNEL f1 ========
SFO1 125.7879670 MHz
NUC1 13C
P1 10.00 usec
PLW1 88.00000000 W
======== CHANNEL f2 ========
SFO2 500.2020008 MHz
NUC2 1H
CPDPRG[2 waltz16
PCPD2 80.00 usec
PLW2 22.00000000 W
PLW12 0.34375000 W
PLW13 0.22000000 W
F2 - Processing parameters
SI 32768
SF 125.7754515 MHz
WDW EM
SSB 0
LB 1.00 Hz
GB 0
PC 1.40
200
180
160
140
120
100
80
60
40
20
0
ppm

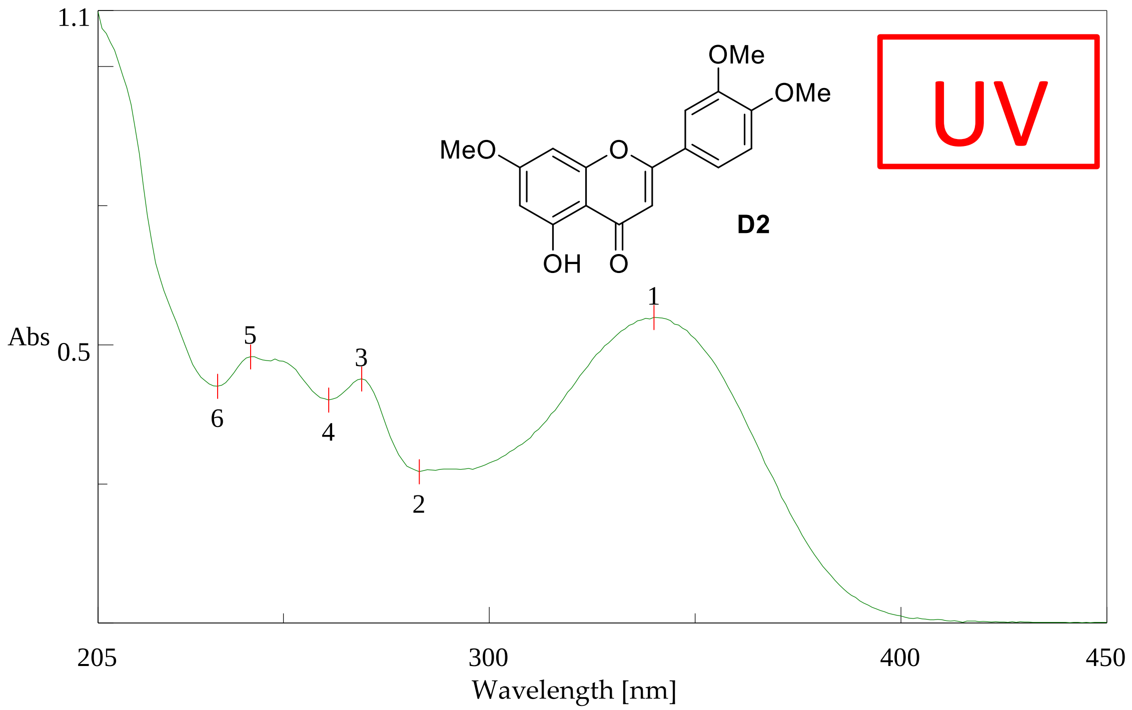

[Result of Peak Picking]

No.	Position	Intensity	No.	Position	Intensity	No.	Position	Intensity
1	340	0.548955	2	283	0.271724	3	269	0.438749
4	261	0.400295	5	242	0.478333	6	234	0.425517

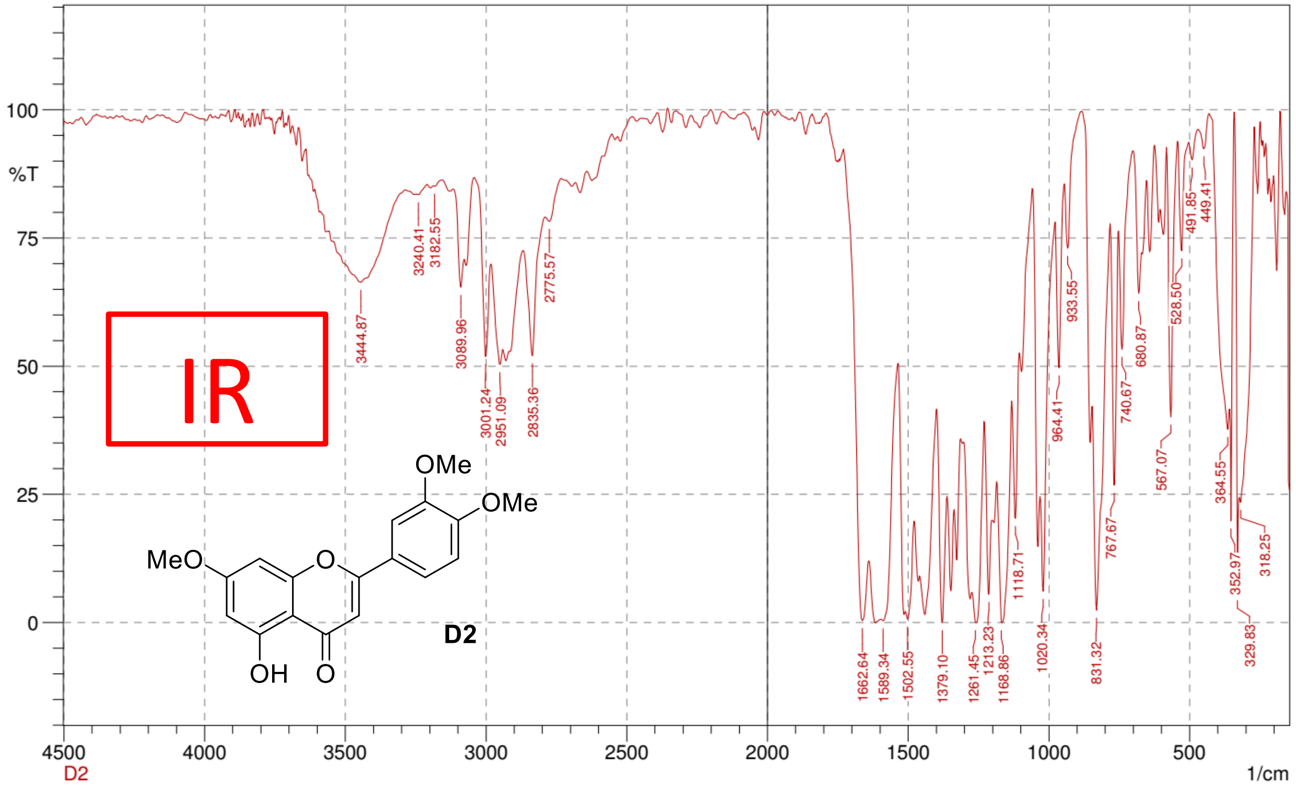

MS

Qualitative Analysis Report

**Data Filename**	4-DDM.d	**Sample Name**	4-DDM
**Sample Type**	Sample	**Position**	P2-B5
**Instrument Name**	Instrument 1	**User Name**	
**Acq Method**	Cot ngan - MSMS_Pos.m	**Acquired Time**	04/08/2020 7:34:20 PM
**IRM Calibration Status**	Success	**DA Method**	COTNGAN.M.m
**Comment**			

**Sample Group**		**Info.**	
**Stream Name**	LC 1	**Acquisition SW Version**	6200 series TOF/6500 series Q-TOF B.06.01 (B6172 SP1)

User Chromatograms

Fragmentor Voltage 140 **Collision Energy** 0 **Ionization Mode** ESI

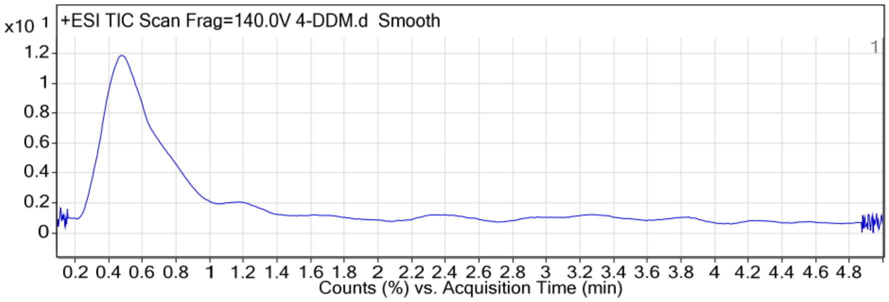

User Spectra

Fragmentor Voltage 140 **Collision Energy** 0 **Ionization Mode** ESI

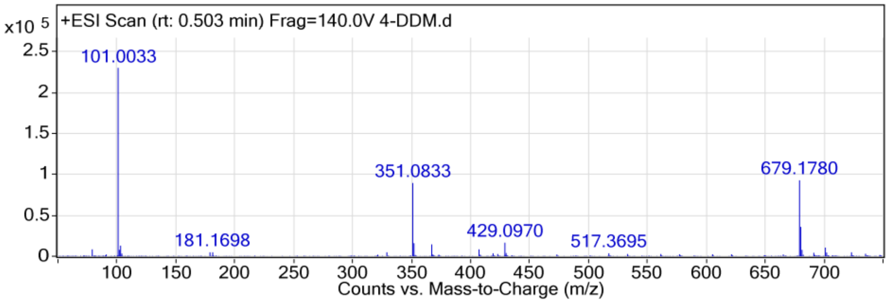

Peak List

m/z	z	Abund
79.0211		8484.25
101.0033	1	230221.81
102.999	1	12995.89
351.0833	1	89560.98
352.0868	1	15988.85
367.0573	1	14627.99
429.097	1	16630.26
679.178	1	92758.16
680.1805	1	36038.07
701.1592	1	10518.93

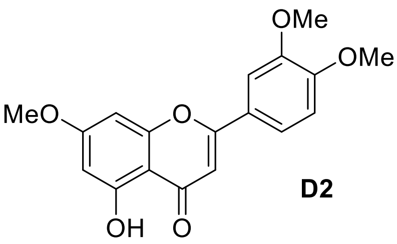

D2

--- End Of Report ---

MS

Sample Name	4-DDM	Position	P2-B5	Instrument Name	Instrument 1	User Name	
Inj Vol	2	InjPosition		SampleType	Sample	IRM Calibration Status	Success
Data Filename	4-DDM.d	ACQ Method	Cot ngan - MSMS_Pos.	Comment		Acquired Time	04/08/2020 7:34:20 PM

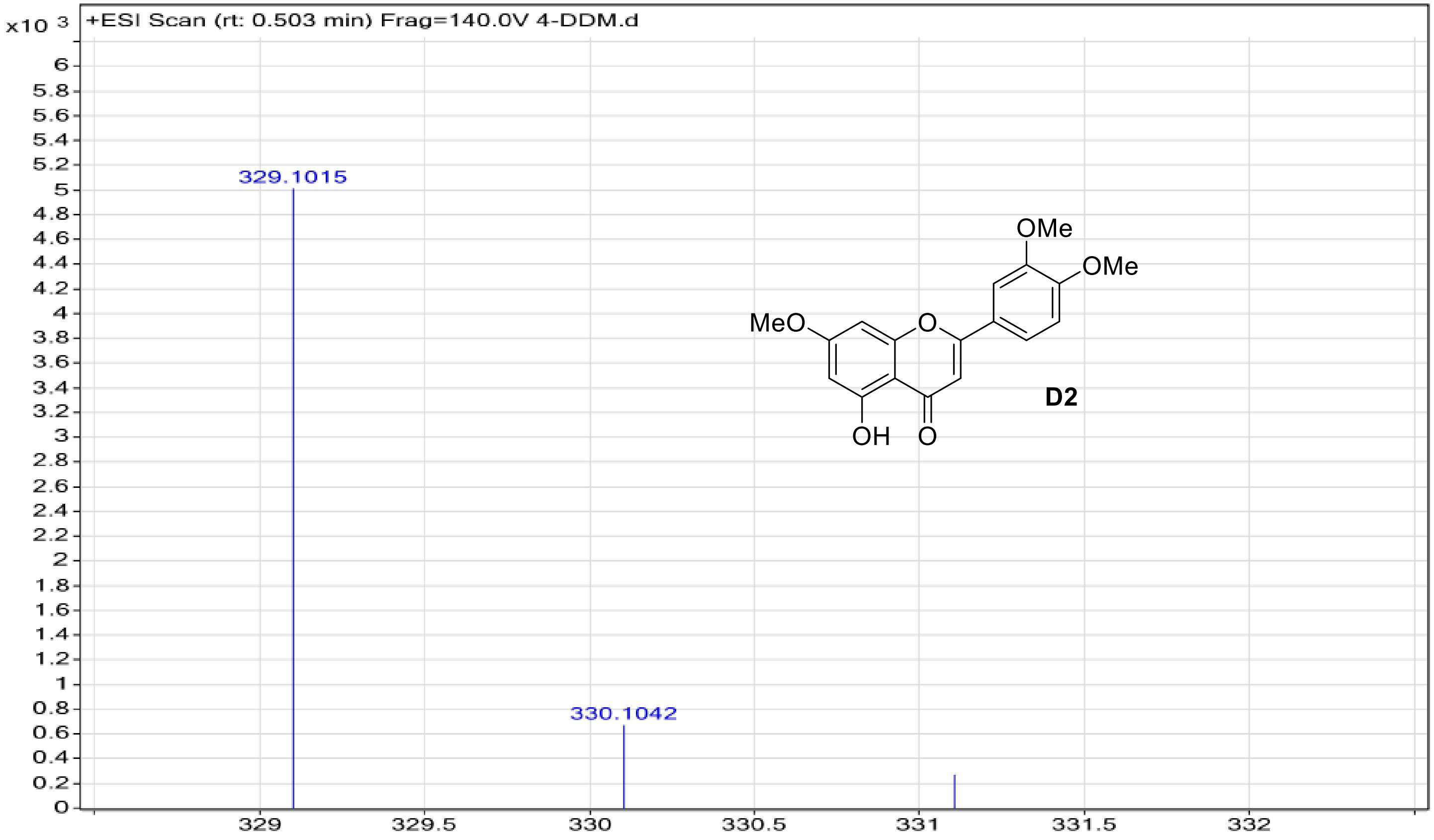

^{1}H-NMR

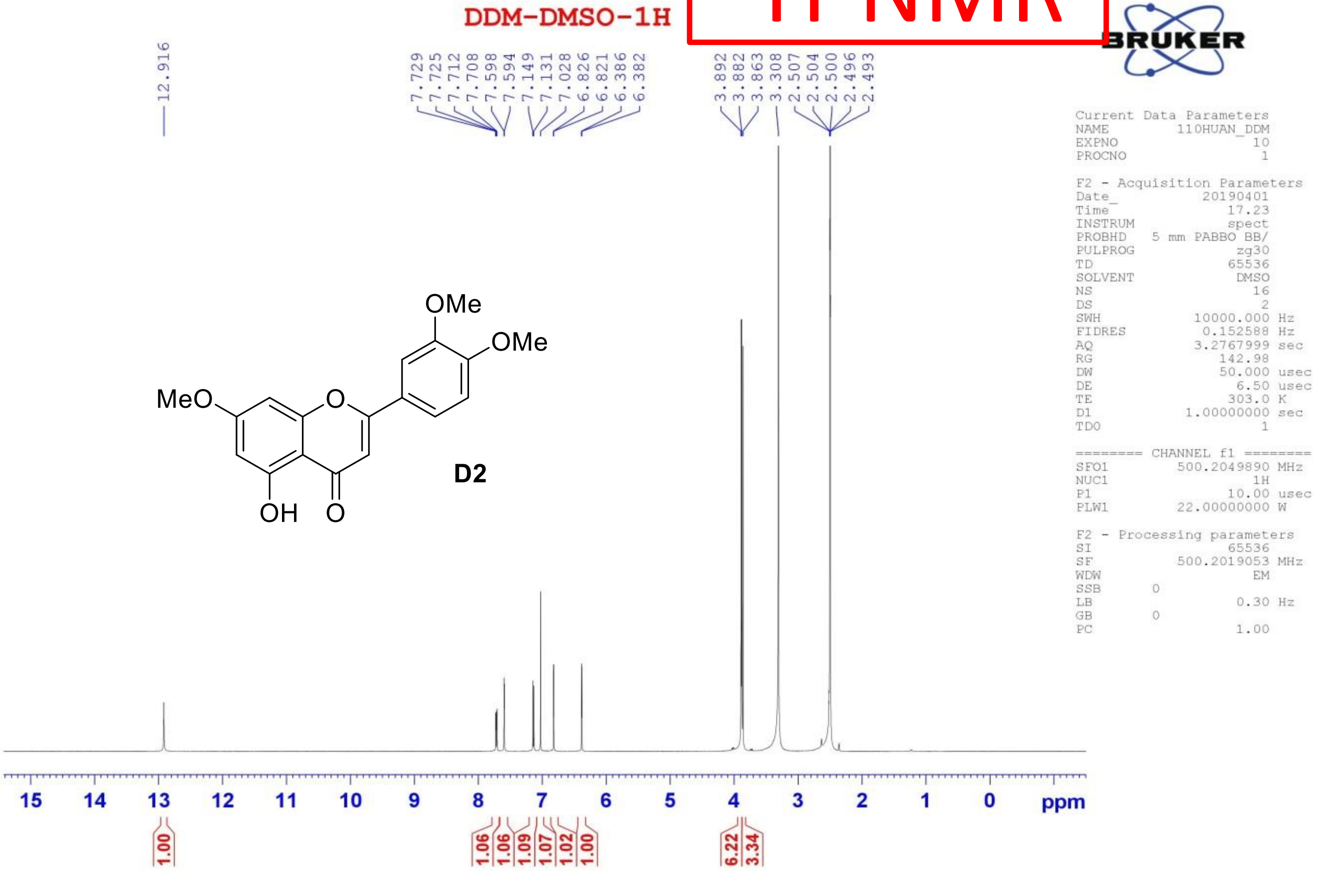
DDM-DMSO-1H
BRUKER
12.916
7.729 7.725 7.712 7.708 7.598 7.594 7.149 7.131 7.028 6.826 6.821 6.386 6.382
3.892 3.882 3.863 3.308 2.507 2.504 2.500 2.496 2.493
OMe
OMe
MeO
O
OH
O
D2
Current Data Parameters
NAME 110HUAN_DDM
EXPNO 10
PROCNO 1
F2 - Acquisition Parameters
Date_ 20190401
Time_ 17.23
INSTRUM spect
PROBHD 5 mm PABBO BB/
PULPROG zg30
TD 65536
SOLVENT DMSO
NS 16
DS 2
SWH 10000.000 Hz
FIDRES 0.152588 Hz
AQ 3.2767999 sec
RG 142.98
DW 50.000 usec
DE 6.50 usec
TE 303.0 K
D1 1.00000000 sec
TD0 1
======== CHANNEL f1 ========
SFO1 500.2049890 MHz
NUC1 1H
P1 10.00 usec
PLW1 22.00000000 W
F2 - Processing parameters
SI 65536
SF 500.2019053 MHz
WDW EM
SSB 0
LB 0.30 Hz
GB 0
PC 1.00
15 14 13 12 11 10 9 8 7 6 5 4 3 2 1 0 ppm
1.00
1.06 1.06 1.09 1.07 1.02 1.00
6.22 3.34

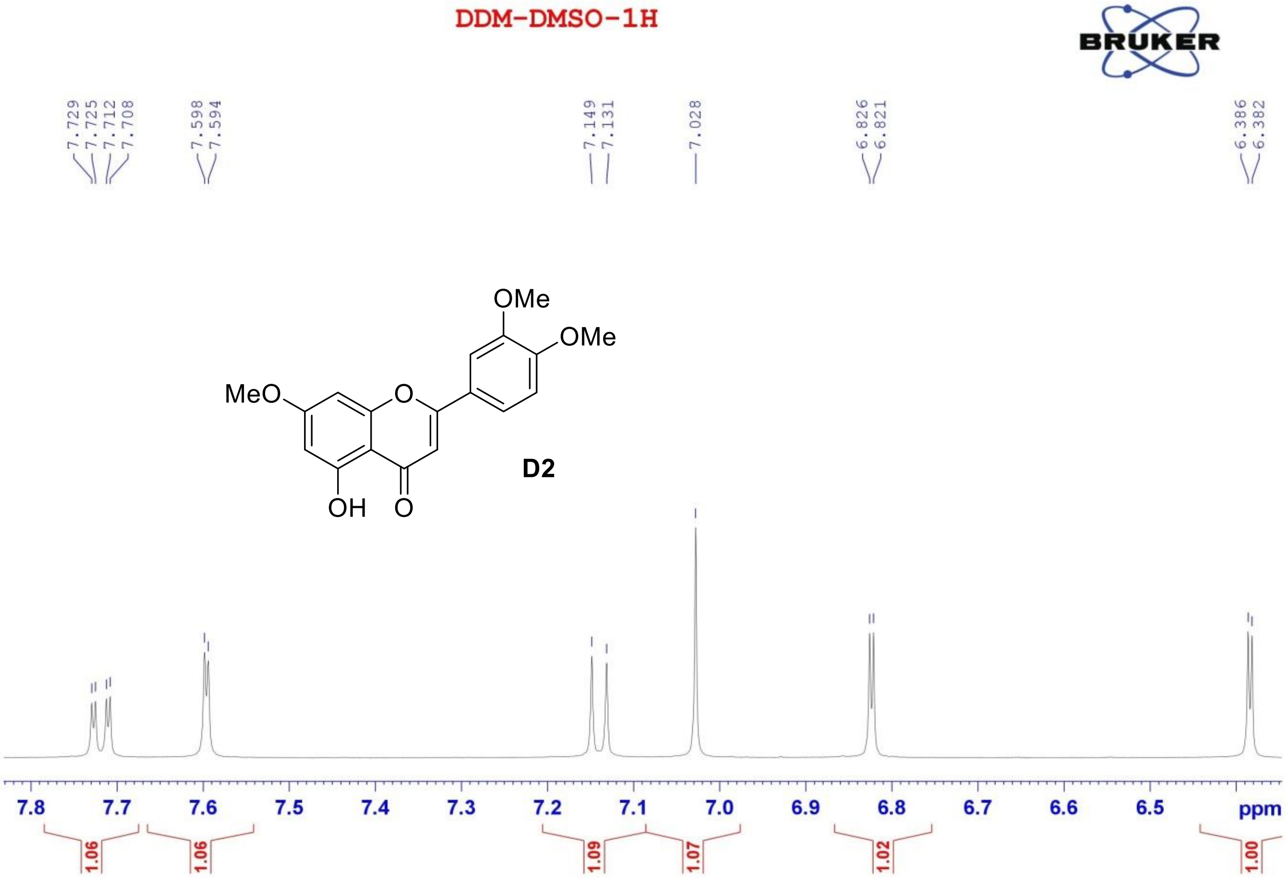
DDM-DMSO-1H
BRUKER
7.729 7.725 7.712 7.708
7.598 7.594
7.149 7.131
7.028
6.826 6.821
6.386 6.382
OMe
OMe
MeO
O
OH
O
D2
7.8 7.7 7.6 7.5 7.4 7.3 7.2 7.1 7.0 6.9 6.8 6.7 6.6 6.5 ppm
1.06 1.06 1.09 1.07 1.02 1.00

DDM-DMSO-C13CPD

^{13}C-NMR

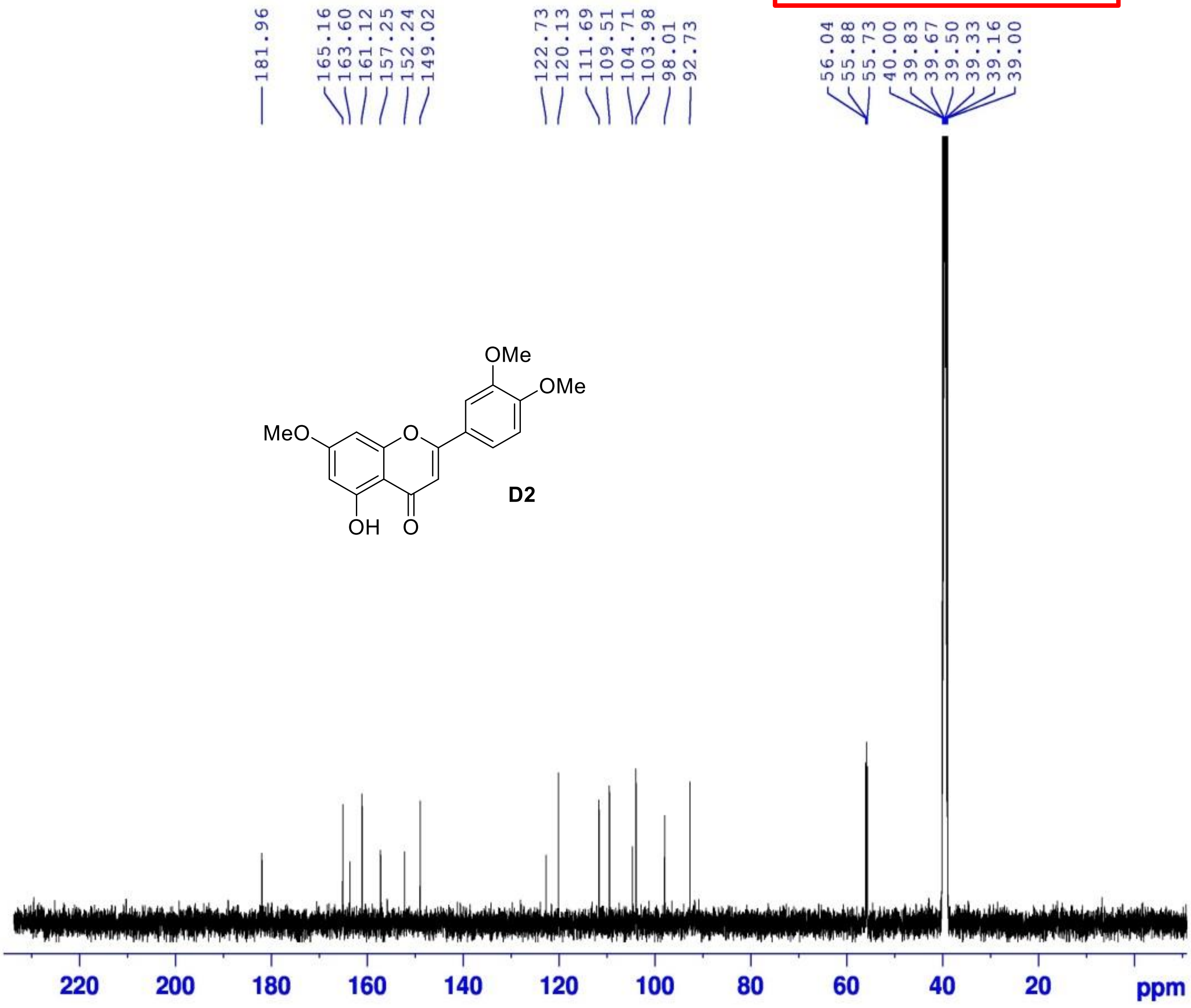

BRUKER

Current Data Parameters
NAME 110HUAN_DDM
EXPNO 2
PROCNO 1

F2 - Acquisition Parameters
Date_ 20190401
Time 19.40
INSTRUM spect
PROBHD 5 mm PABBO BB/
PULPROG zgpg30
TD 65536
SOLVENT DMSO
NS 2048
DS 4
SWH 31250.000 Hz
FIDRES 0.476837 Hz
AQ 1.0485760 sec
RG 198.57
DW 16.000 usec
DE 6.50 usec
TE 303.5 K
D1 2.00000000 sec
D11 0.03000000 sec
TD0 1

======== CHANNEL f1 ========
SFO1 125.7897032 MHz
NUC1 13C
P1 10.00 usec
PLW1 88.00000000 W

======== CHANNEL f2 ========
SFO2 500.2039008 MHz
NUC2 1H
CPDPRG[2 waltz16
PCPD2 80.00 usec
PLW2 22.00000000 W
PLW12 0.34375000 W
PLW13 0.22000000 W

F2 - Processing parameters
SI 32768
SF 125.7759351 MHz
WDW EM
SSB 0
LB 1.00 Hz
GB 0
PC 1.40

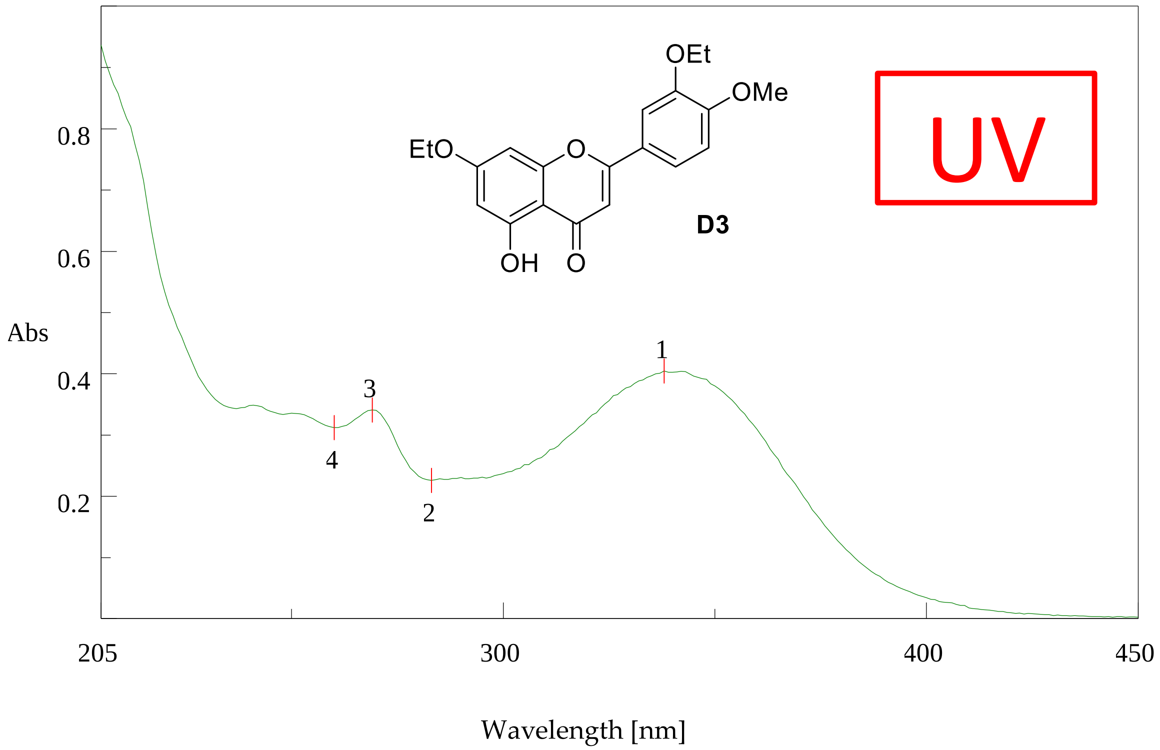

[Result of Peak Picking]

No.	Position	Intensity	No.	Position	Intensity	No.	Position	Intensity
1	338	0.404347	2	283	0.225658	3	269	0.340507
4	260	0.311738						

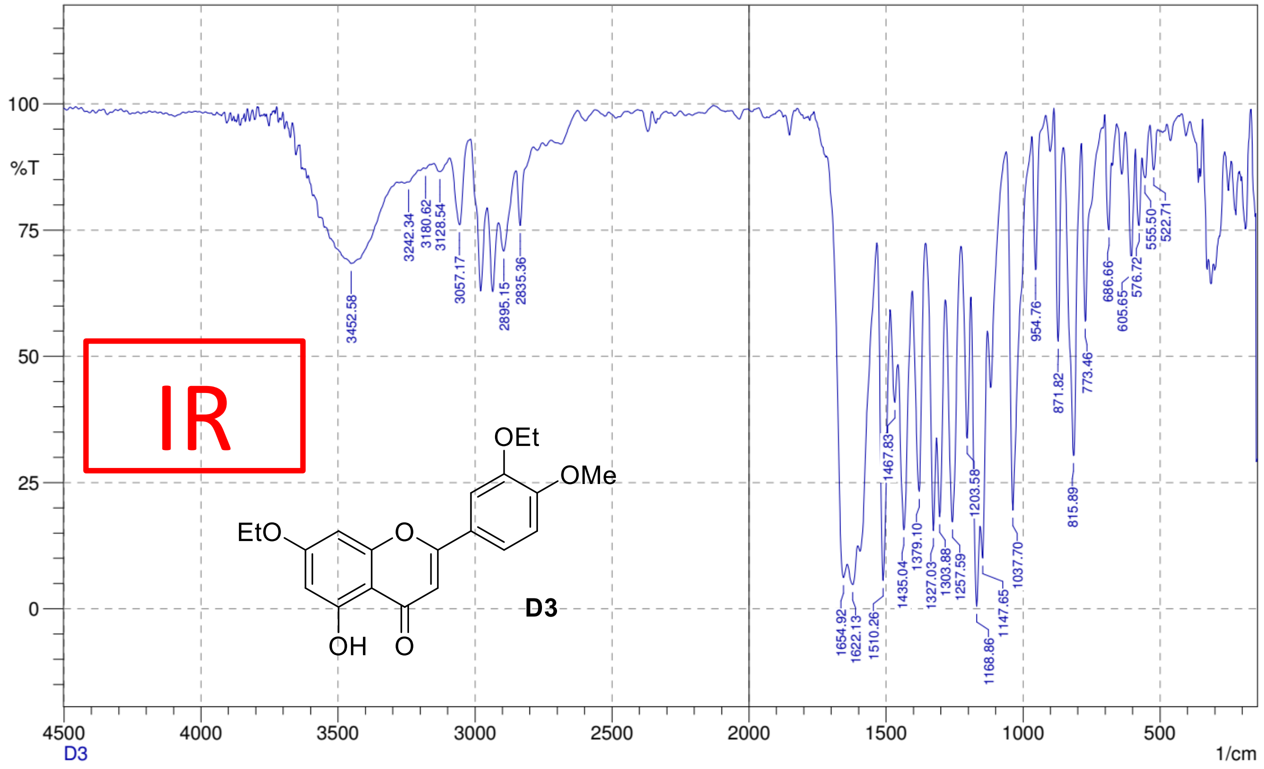

MS

Qualitative Analysis Report

**Data Filename**	6-DDE.d	**Sample Name**	6-DDE
**Sample Type**	Sample	**Position**	P2-B3
**Instrument Name**	Instrument 1	**User Name**	
**Acq Method**	Cot ngan - MSMS_Pos.m	**Acquired Time**	04/08/2020 7:23:11 PM
**IRM Calibration Status**	Success	**DA Method**	COTNGAN.M.m
**Comment**			

**Sample Group**		**Info.**	
**Stream Name**	LC 1	**Acquisition SW Version**	6200 series TOF/6500 series Q-TOF B.06.01 (B6172 SP1)

User Chromatograms

Fragmentor Voltage 140 **Collision Energy** 0 **Ionization Mode** ESI

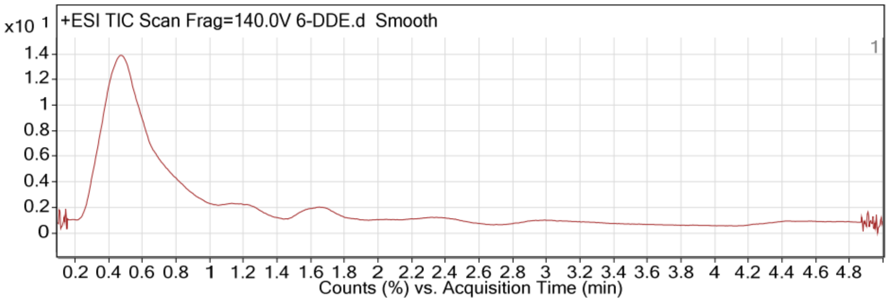

User Spectra

Fragmentor Voltage 140 **Collision Energy** 0 **Ionization Mode** ESI

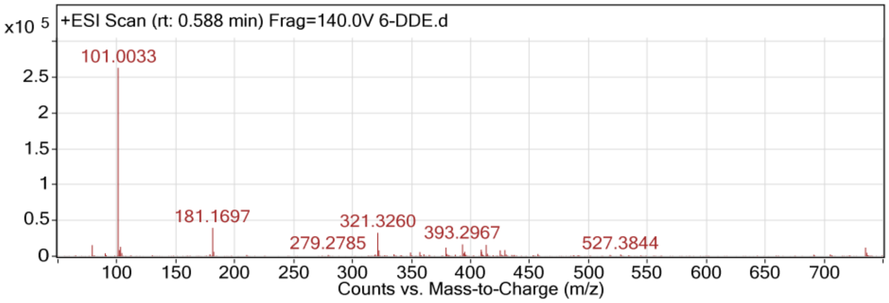

Peak List

m/z	z	Abund
79.0209	1	15483.26
101.0033	1	263178.06
102.9988	1	13295.03
181.1697	1	39590.21
321.326	1	32958.98
379.1142	1	12143.51
393.2967	1	16460.94
409.1613	1	9409.05
413.2654	1	15935.65
735.2404	1	12220.2

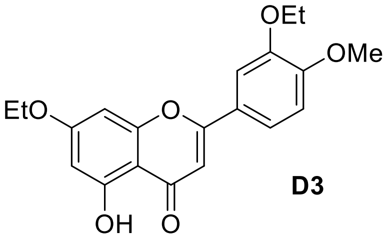

D3

--- End Of Report ---

MS

**Sample Name**	6-DDE	**Position**	P2-B3	**Instrument Name**	Instrument 1	**User Name**	
**Inj Vol**	2	**InjPosition**		**SampleType**	Sample	**IRM Calibration Status**	Success
**Data Filename**	6-DDE.d	**ACQ Method**	Cot ngan - MSMS_Pos.	**Comment**		**Acquired Time**	04/08/2020 7:23:11 PM

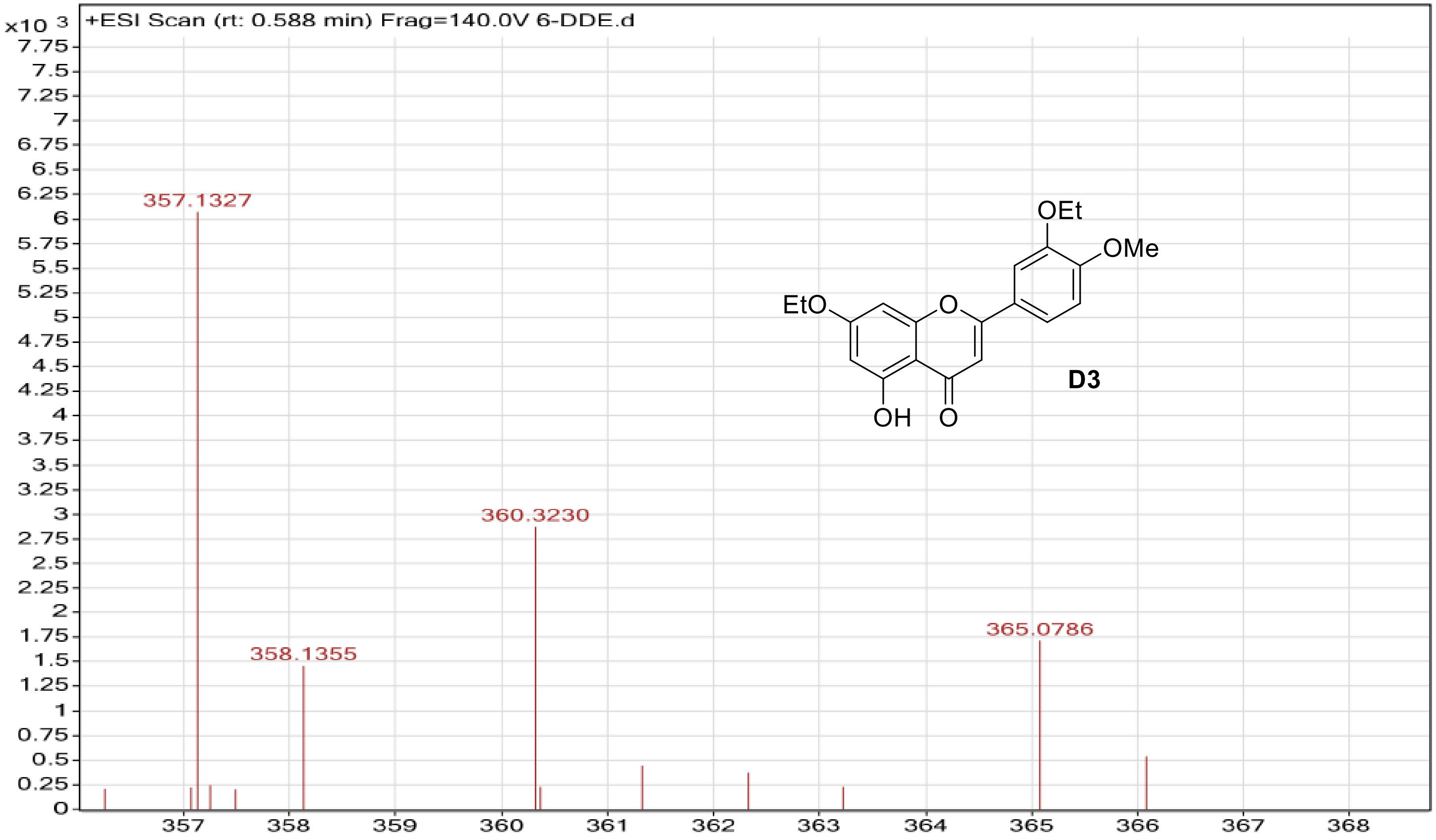

^{1}H-NMR

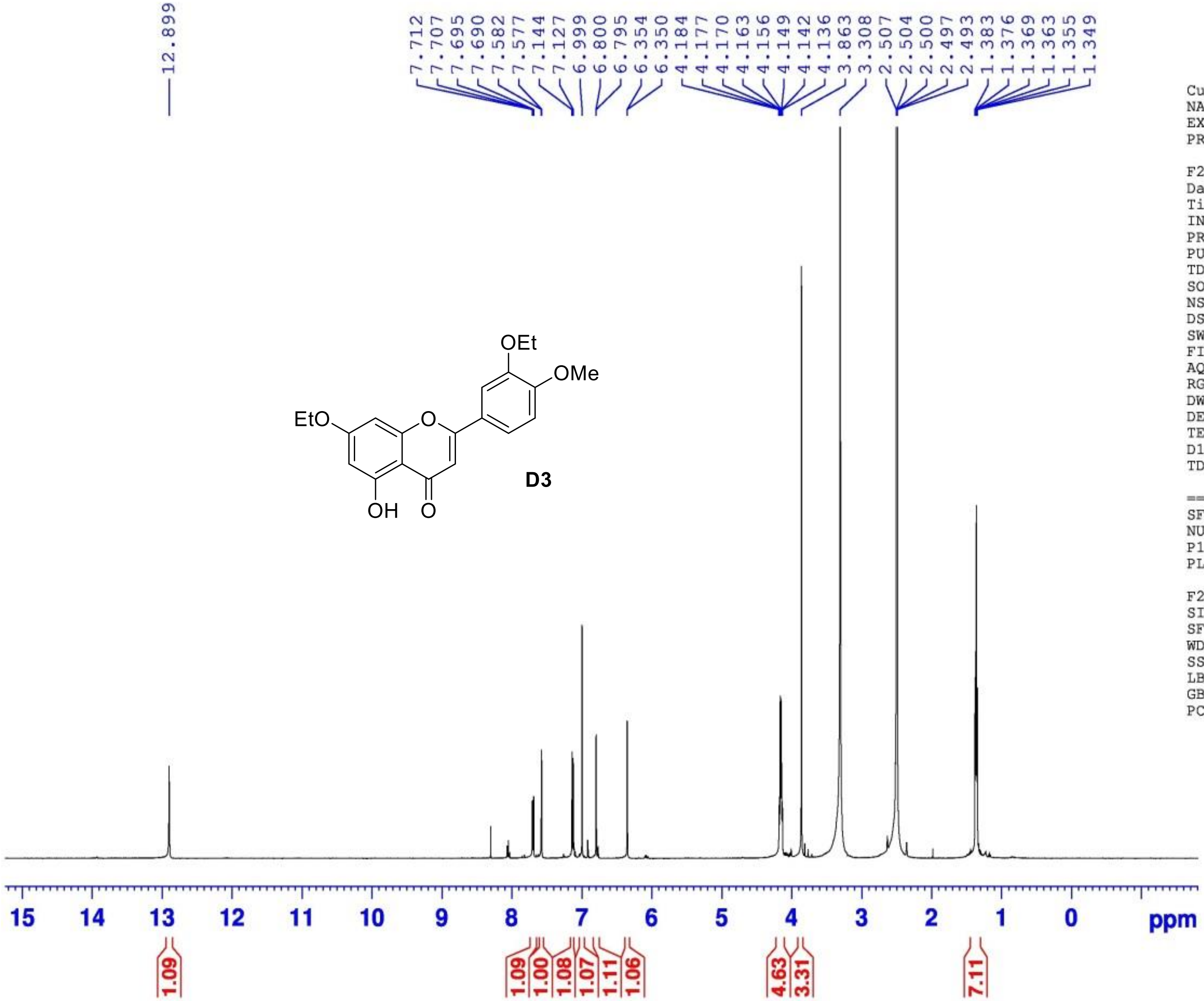


```
Current Data Parameters
NAME              110HUAN_DDE
EXPNO                      10
PROCNO                      1

F2 - Acquisition Parameters
Date_                20190401
Time                    17.13
INSTRUM                 spect
PROBHD     5 mm PABBO BB/
PULPROG                  zg30
TD                      65536
SOLVENT                  DMSO
NS                         16
DS                          2
SWH              10000.000 Hz
FIDRES            0.152588 Hz
AQ               3.2767999 sec
RG                     142.98
DW                 50.000 usec
DE                   6.50 usec
TE                    303.0 K
D1              1.00000000 sec
TD0                         1

======== CHANNEL f1 ========
SFO1           500.2049890 MHz
NUC1                       1H
P1                 10.00 usec
PLW1           22.00000000 W

F2 - Processing parameters
SI                      65536
SF             500.2019051 MHz
WDW                        EM
SSB        0
LB                    0.30 Hz
GB         0
PC                       1.00
```

^{1}H-NMR

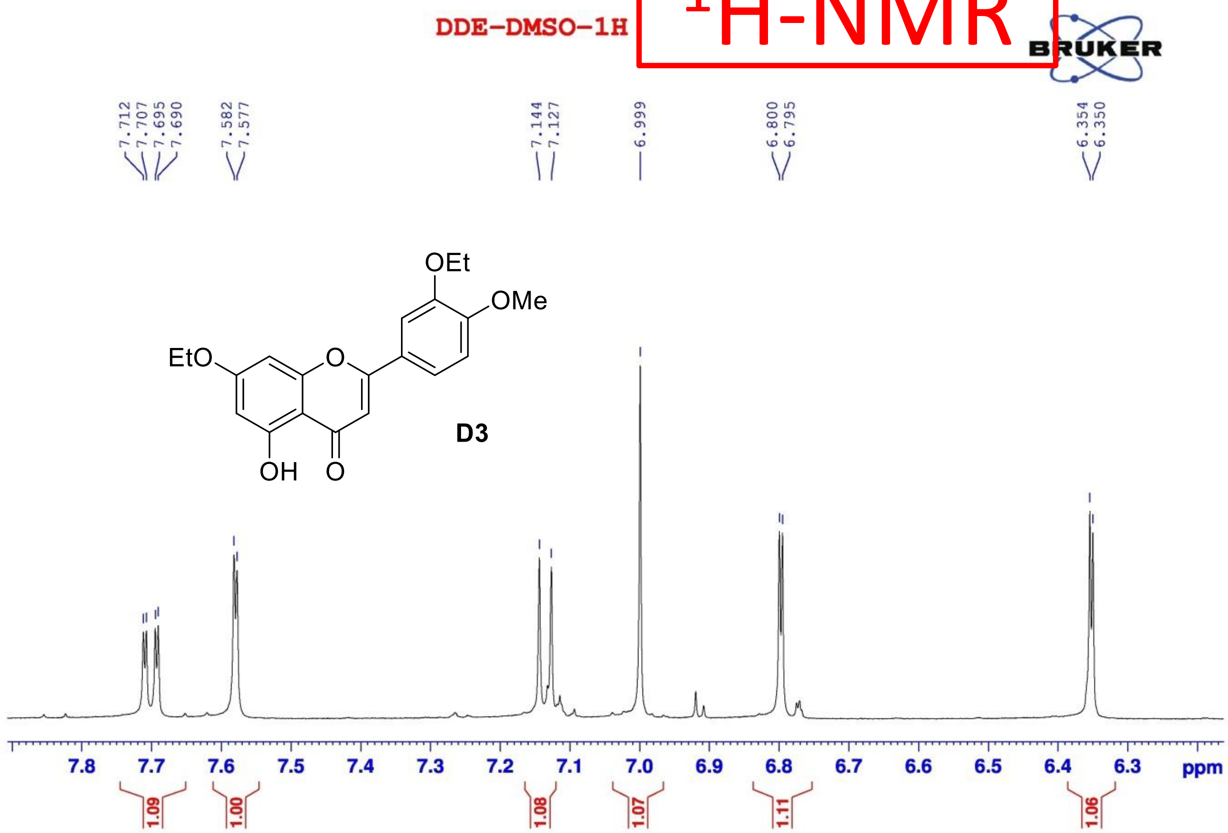
DDE-DMSO-1H
BRUKER
7.712
7.707
7.695
7.690
7.582
7.577
7.144
7.127
6.999
6.800
6.795
6.354
6.350
OEt
OMe
EtO
O
OH
O
D3
7.8
7.7
7.6
7.5
7.4
7.3
7.2
7.1
7.0
6.9
6.8
6.7
6.6
6.5
6.4
6.3
ppm
1.09
1.00
1.08
1.07
1.11
1.06

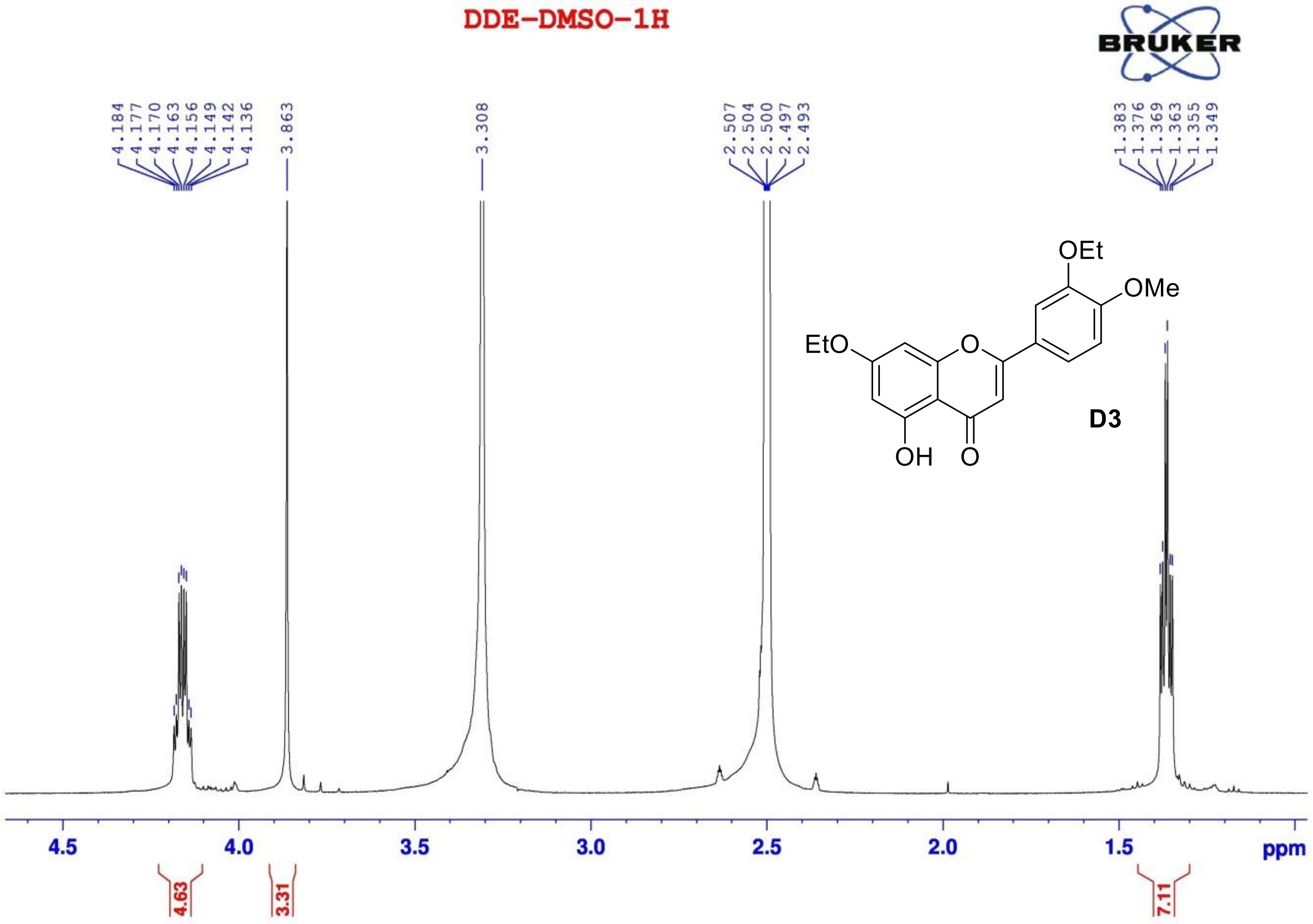
DDE-DMSO-1H
BRUKER
4.184
4.177
4.170
4.163
4.156
4.149
4.142
4.136
3.863
3.308
2.507
2.504
2.500
2.497
2.493
1.383
1.376
1.369
1.363
1.355
1.349
OEt
OMe
EtO
O
OH
O
D3
4.5
4.0
3.5
3.0
2.5
2.0
1.5
ppm
4.63
3.31
7.11

DDE-DMSO-C13CPD

^{13}C-NMR

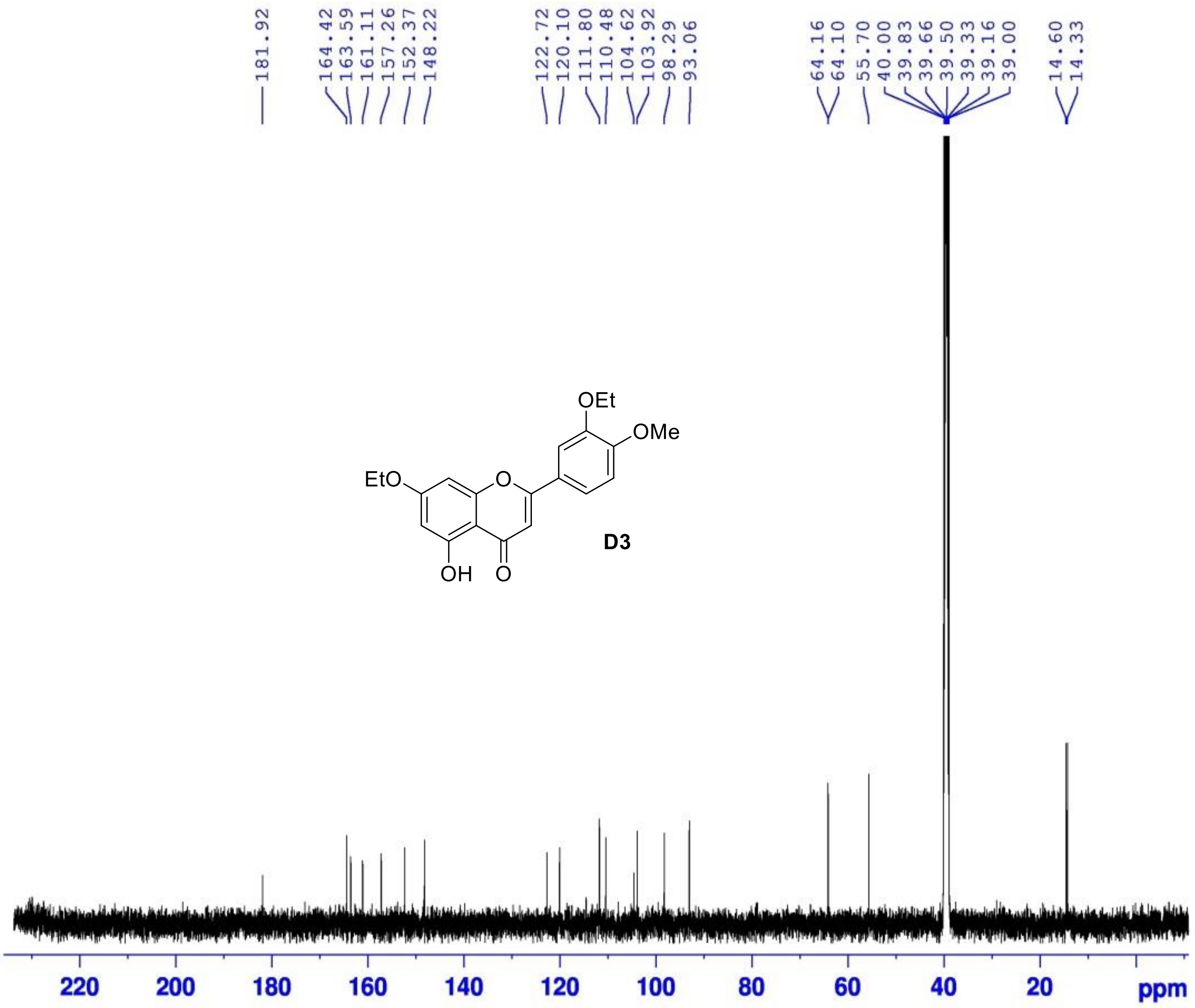

BRUKER

Current Data Parameters
NAME 110HUAN_DDE
EXPNO 2
PROCNO 1

F2 - Acquisition Parameters
Date_ 20190401
Time 18.25
INSTRUM spect
PROBHD 5 mm PABBO BB/
PULPROG zgpg30
TD 65536
SOLVENT DMSO
NS 2048
DS 4
SWH 31250.000 Hz
FIDRES 0.476837 Hz
AQ 1.0485760 sec
RG 198.57
DW 16.000 usec
DE 6.50 usec
TE 303.4 K
D1 2.00000000 sec
D11 0.03000000 sec
TD0 1

======== CHANNEL f1 ========
SFO1 125.7897032 MHz
NUC1 13C
P1 10.00 usec
PLW1 88.00000000 W

======== CHANNEL f2 ========
SFO2 500.2039008 MHz
NUC2 1H
CPDPRG[2 waltz16
PCPD2 80.00 usec
PLW2 22.00000000 W
PLW12 0.34375000 W
PLW13 0.22000000 W

F2 - Processing parameters
SI 32768
SF 125.7759349 MHz
WDW EM
SSB 0
LB 1.00 Hz
GB 0
PC 1.40

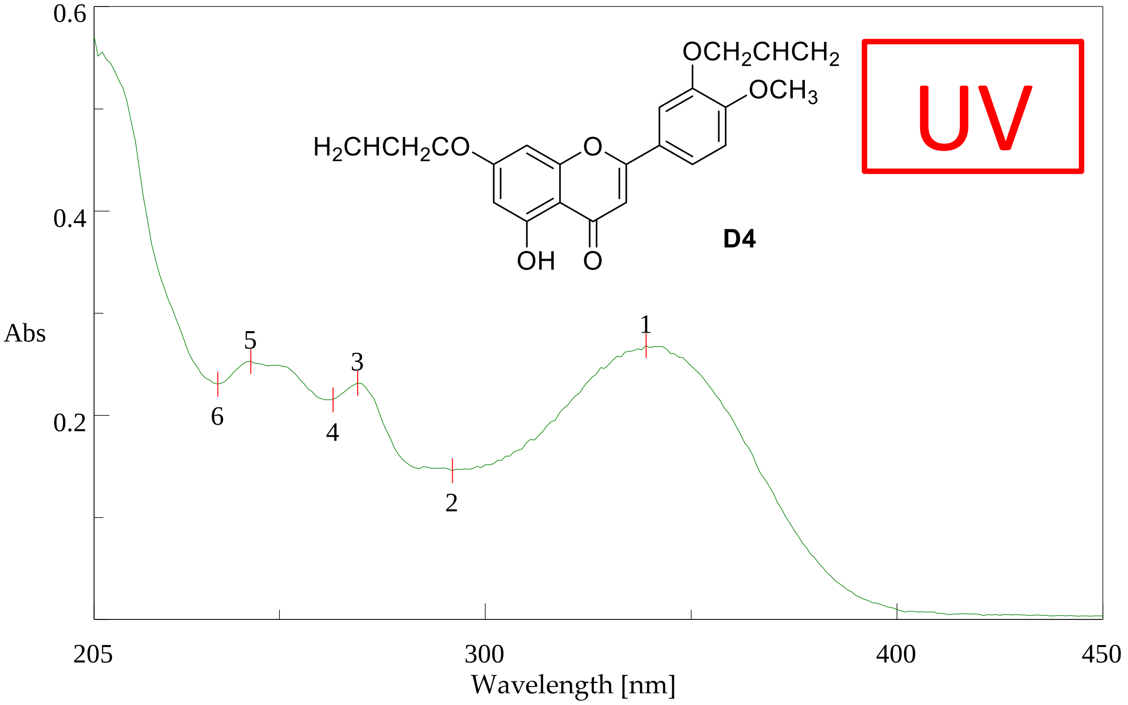

[Result of Peak Picking]

No.	Position	Intensity	No.	Position	Intensity	No.	Position	Intensity
1	339	0.268015	2	292	0.145635	3	269	0.231266
4	263	0.214911	5	243	0.252528	6	235	0.230563

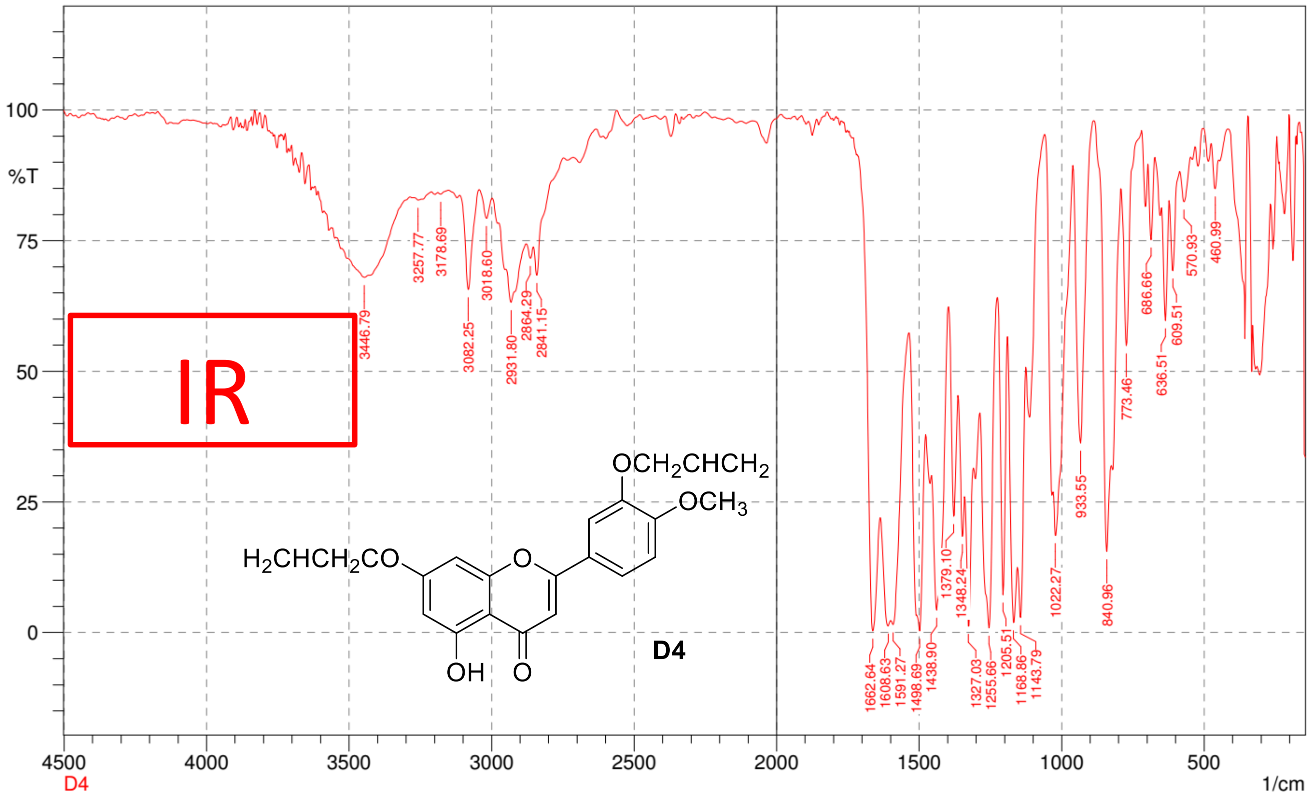

MS

Qualitative Analysis Report

**Data Filename**	0-DDAI.d	**Sample Name**	0-DDAI
**Sample Type**	Sample	**Position**	P2-A9
**Instrument Name**	Instrument 1	**User Name**	
**Acq Method**	Cot ngan - MSMS_Pos.m	**Acquired Time**	04/08/2020 7:06:30 PM
**IRM Calibration Status**	Success	**DA Method**	COTNGAN.M.m
**Comment**			

**Sample Group**		**Info.**	
**Stream Name**	LC 1	**Acquisition SW Version**	6200 series TOF/6500 series Q-TOF B.06.01 (B6172 SP1)

User Chromatograms

Fragmentor Voltage 140 **Collision Energy** 0 **Ionization Mode** ESI

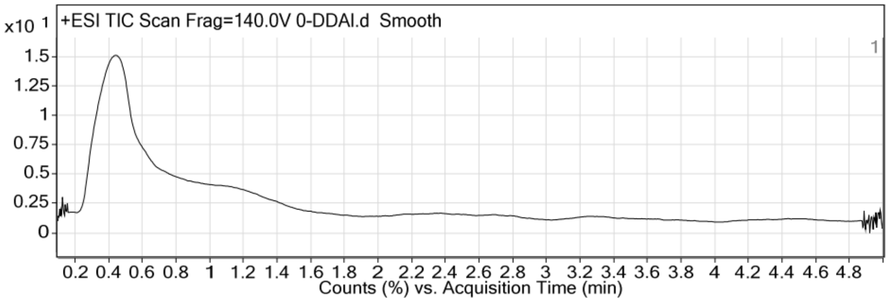

User Spectra

Fragmentor Voltage	Collision Energy	Ionization Mode
140	0	ESI

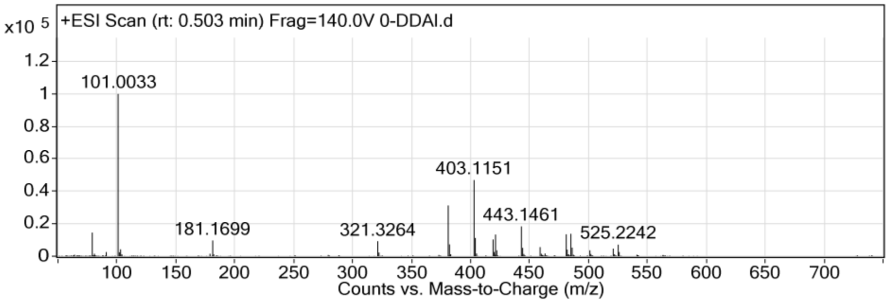

Peak List

m/z	z	Abund
101.0033	1	99821.3
381.1333	1	31223.72
403.1151	1	46981.47
443.1461	1	18364.74
783.2412	1	115923.51
784.2443	1	55489.42
785.2467	1	14720.78
823.2723	1	99211.97
824.2753	1	45365.66
863.3028	1	25991.62

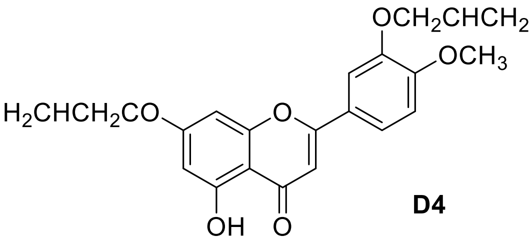

D4

--- End Of Report ---

MS

**Sample Name**	0-DDAI	**Position**	P2-A9	**Instrument Name**	Instrument 1	**User Name**	
**Inj Vol**	2	**InjPosition**		**SampleType**	Sample	**IRM Calibration Status**	Success
**Data Filename**	0-DDAI.d	**ACQ Method**	Cot ngan - MSMS_Pos.	**Comment**		**Acquired Time**	04/08/2020 7:06:30 PM

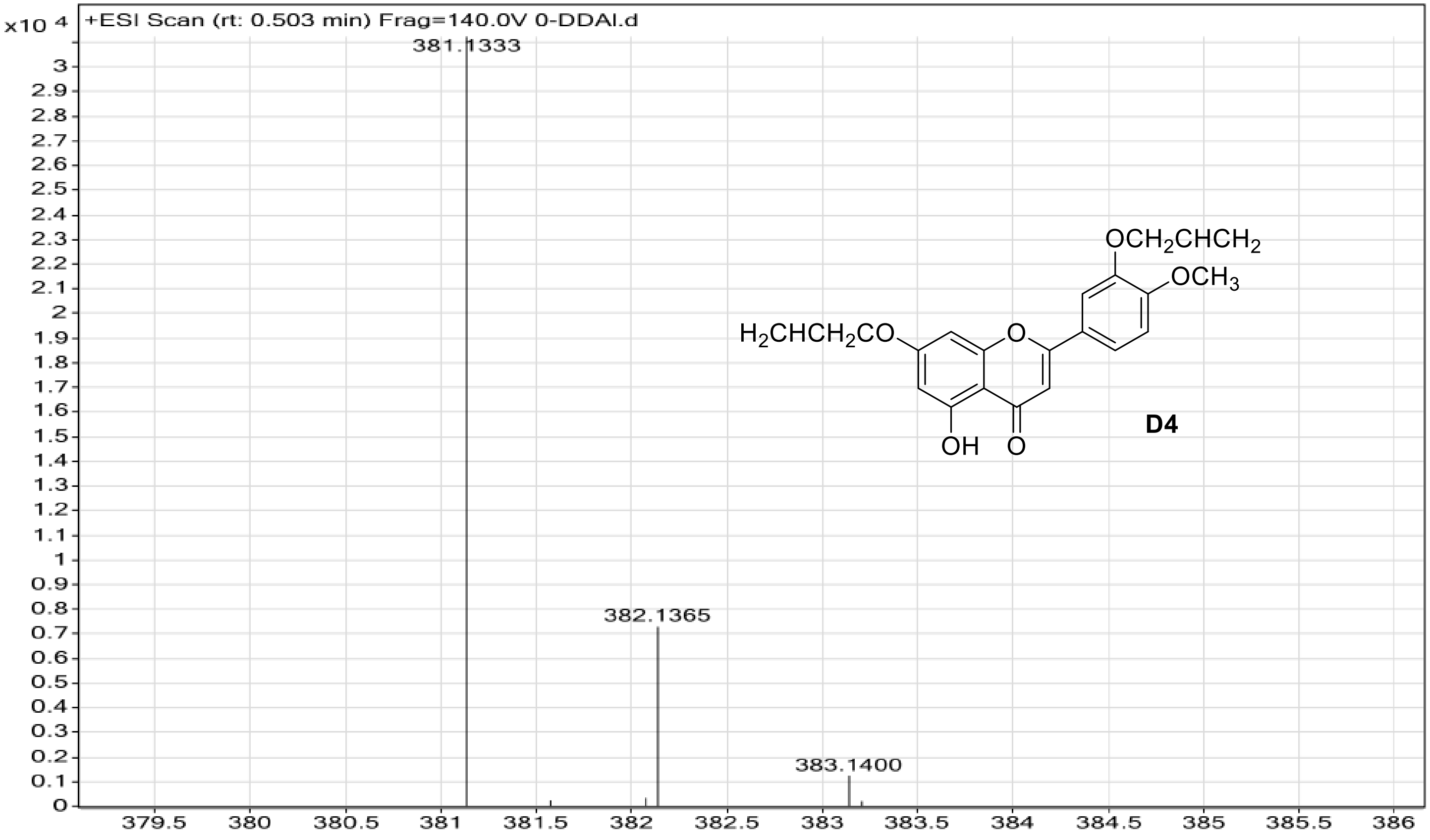

^{1}H-NMR

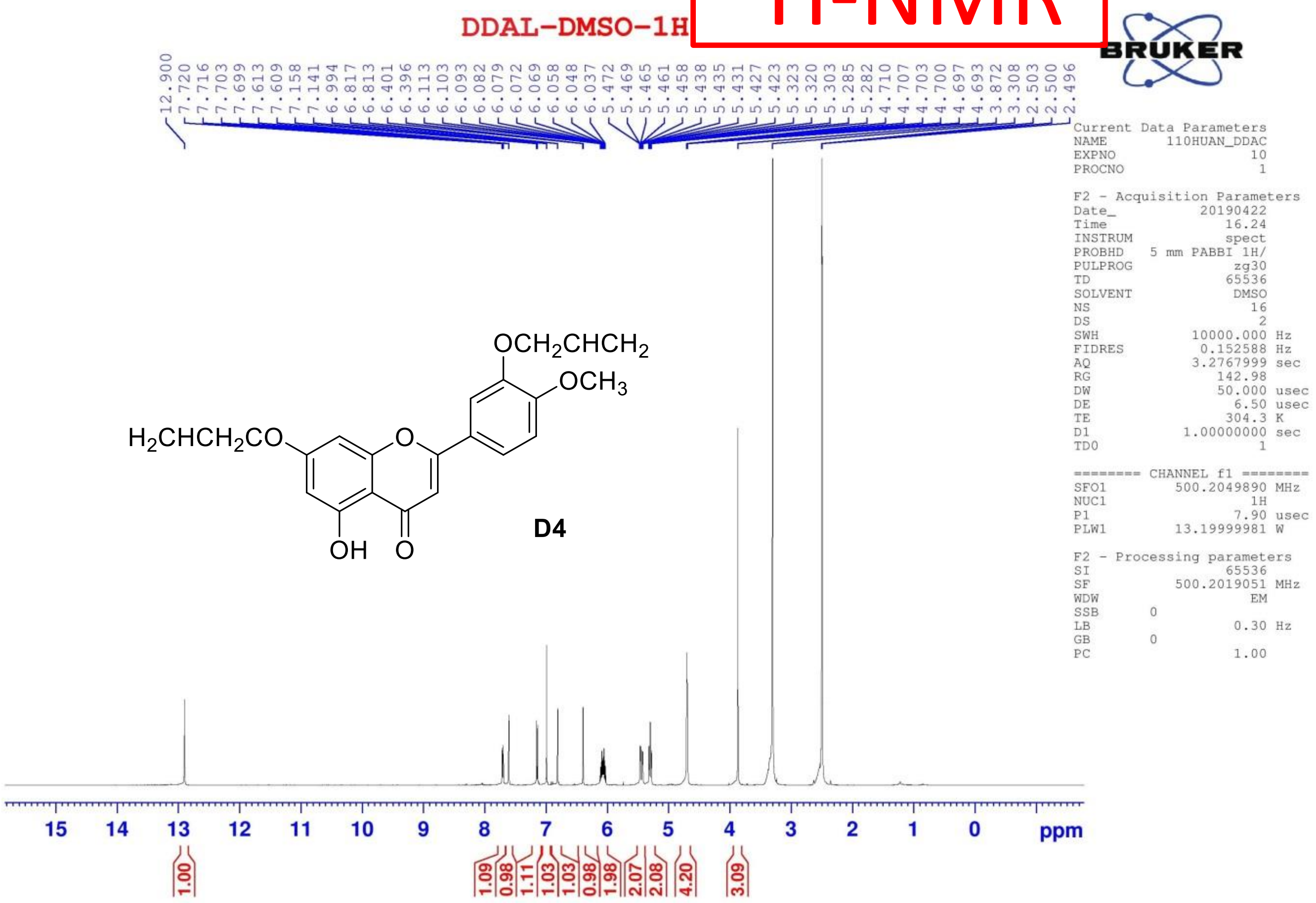
DDAL-DMSO-1H
BRUKER
12.900 7.720 7.716 7.703 7.699 7.613 7.609 7.158 7.141 6.994 6.817 6.813 6.401 6.396 6.113 6.103 6.093 6.082 6.079 6.072 6.069 6.058 6.048 6.037 5.472 5.469 5.465 5.461 5.458 5.438 5.435 5.431 5.427 5.423 5.323 5.320 5.303 5.285 5.282 4.710 4.707 4.703 4.700 4.697 4.693 3.872 3.308 2.503 2.500 2.496
Current Data Parameters
NAME 110HUAN_DDAC
EXPNO 10
PROCNO 1
F2 - Acquisition Parameters
Date_ 20190422
Time 16.24
INSTRUM spect
PROBHD 5 mm PABBI 1H/
PULPROG zg30
TD 65536
SOLVENT DMSO
NS 16
DS 2
SWH 10000.000 Hz
FIDRES 0.152588 Hz
AQ 3.2767999 sec
RG 142.98
DW 50.000 usec
DE 6.50 usec
TE 304.3 K
D1 1.00000000 sec
TD0 1
======== CHANNEL f1 ========
SFO1 500.2049890 MHz
NUC1 1H
P1 7.90 usec
PLW1 13.19999981 W
F2 - Processing parameters
SI 65536
SF 500.2019051 MHz
WDW EM
SSB 0
LB 0.30 Hz
GB 0
PC 1.00
OCH2CHCH2
OCH3
H2CHCH2CO
OH O
D4
15 14 13 12 11 10 9 8 7 6 5 4 3 2 1 0 ppm
1.00 1.09 0.98 1.11 1.03 1.03 0.98 1.98 2.07 2.08 4.20 3.09

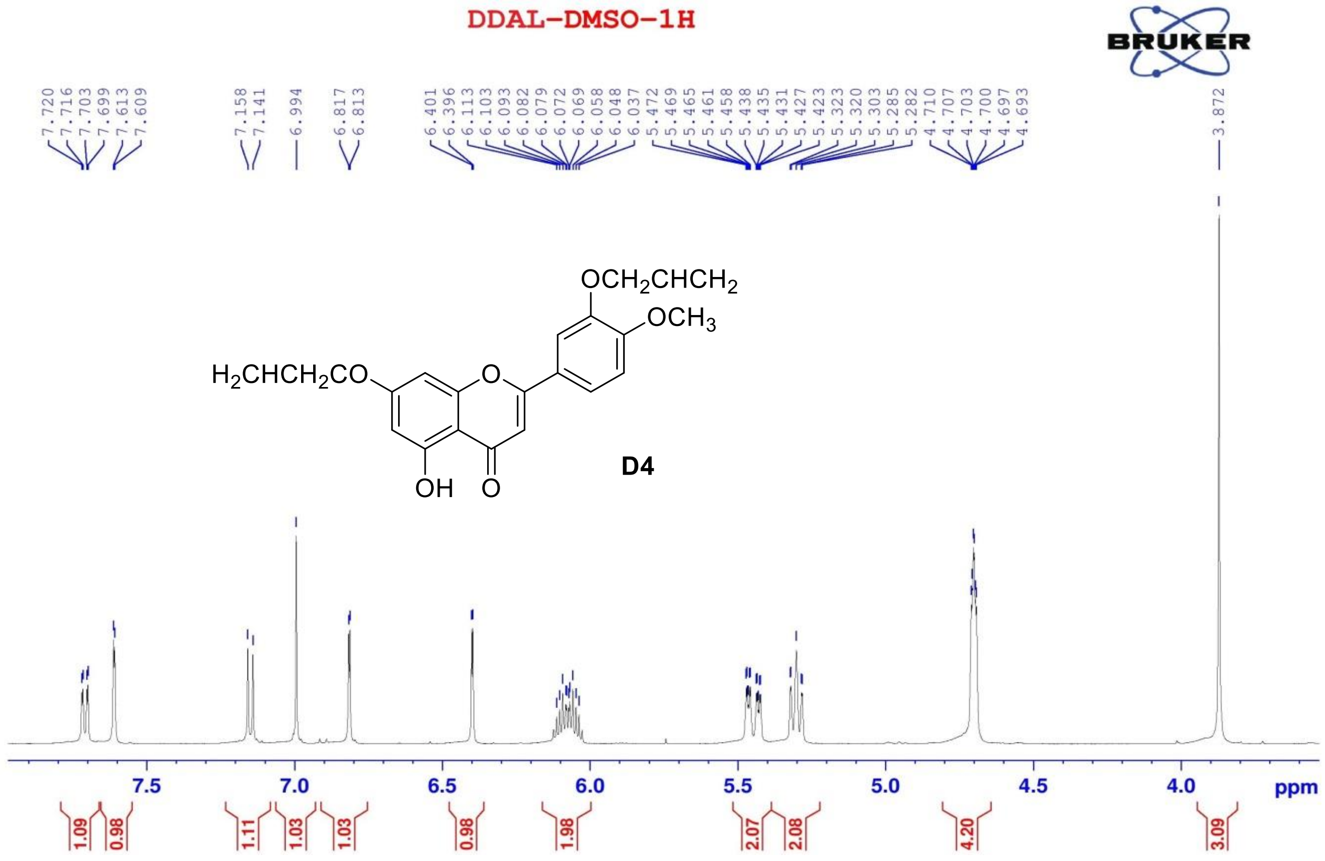
DDAL-DMSO-1H
BRUKER
7.720 7.716 7.703 7.699 7.613 7.609 7.158 7.141 6.994 6.817 6.813 6.401 6.396 6.113 6.103 6.093 6.082 6.079 6.072 6.069 6.058 6.048 6.037 5.472 5.469 5.465 5.461 5.458 5.438 5.435 5.431 5.427 5.423 5.323 5.320 5.303 5.285 5.282 4.710 4.707 4.703 4.700 4.697 4.693 3.872
OCH2CHCH2
OCH3
H2CHCH2CO
OH O
D4
7.5 7.0 6.5 6.0 5.5 5.0 4.5 4.0 ppm
1.09 0.98 1.11 1.03 1.03 0.98 1.98 2.07 2.08 4.20 3.09

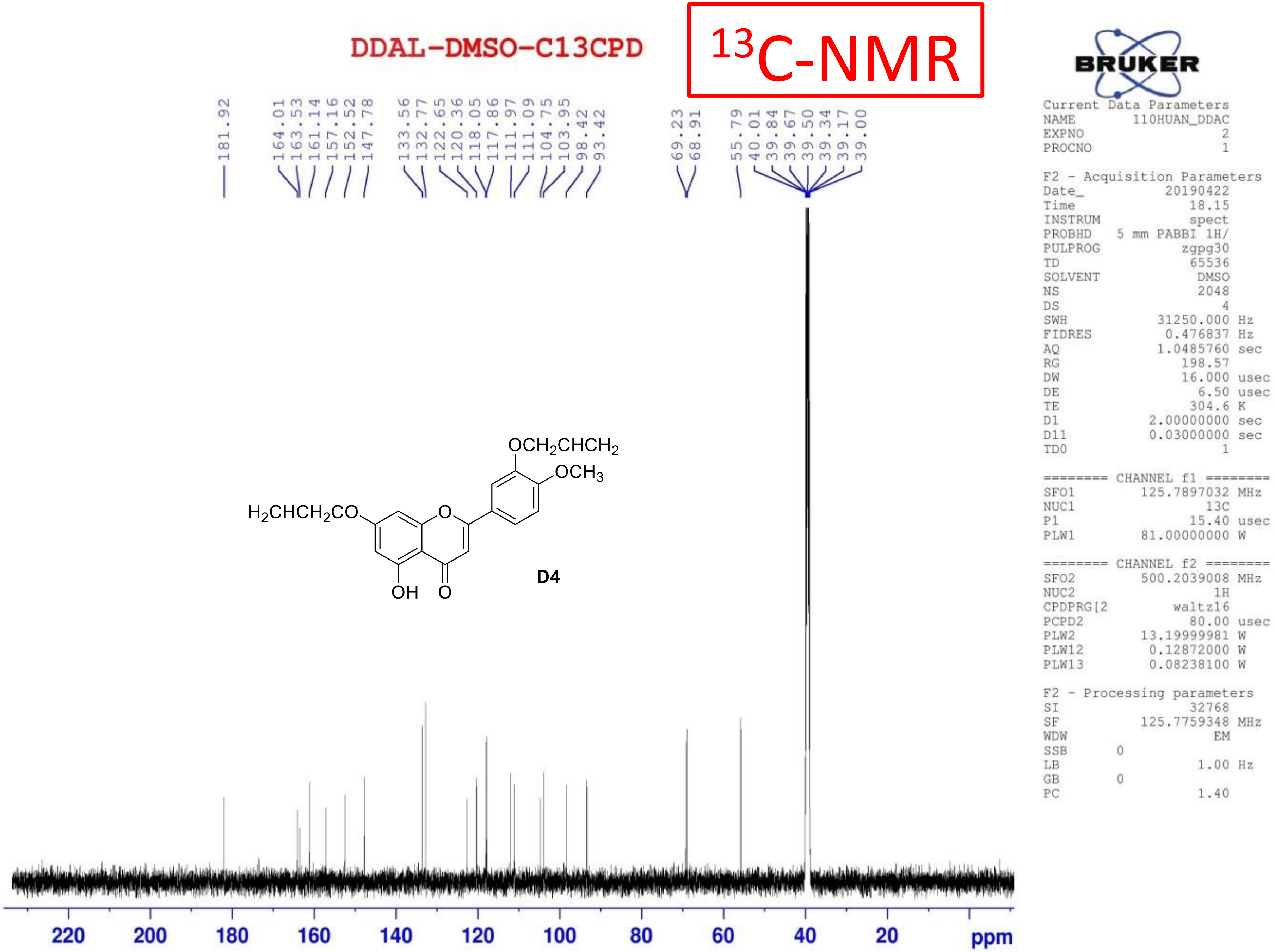

DDAL-DMSO-C13CPD
13C-NMR
BRUKER
181.92
164.01
163.53
161.14
157.16
152.52
147.78
133.56
132.77
122.65
120.36
118.05
117.86
111.97
111.09
104.75
103.95
98.42
93.42
69.23
68.91
55.79
40.01
39.84
39.67
39.50
39.34
39.17
39.00
OCH2CHCH2
OCH3
H2CHCH2CO
O
OH
O
D4
220
200
180
160
140
120
100
80
60
40
20
ppm
Current Data Parameters
NAME 110HUAN_DDAC
EXPNO 2
PROCNO 1
F2 - Acquisition Parameters
Date_ 20190422
Time 18.15
INSTRUM spect
PROBHD 5 mm PABBI 1H/
PULPROG zgpg30
TD 65536
SOLVENT DMSO
NS 2048
DS 4
SWH 31250.000 Hz
FIDRES 0.476837 Hz
AQ 1.0485760 sec
RG 198.57
DW 16.000 usec
DE 6.50 usec
TE 304.6 K
D1 2.00000000 sec
D11 0.03000000 sec
TD0 1
======== CHANNEL f1 ========
SFO1 125.7897032 MHz
NUC1 13C
P1 15.40 usec
PLW1 81.00000000 W
======== CHANNEL f2 ========
SFO2 500.2039008 MHz
NUC2 1H
CPDPRG[2 waltz16
PCPD2 80.00 usec
PLW2 13.19999981 W
PLW12 0.12872000 W
PLW13 0.08238100 W
F2 - Processing parameters
SI 32768
SF 125.7759348 MHz
WDW EM
SSB 0
LB 1.00 Hz
GB 0
PC 1.40

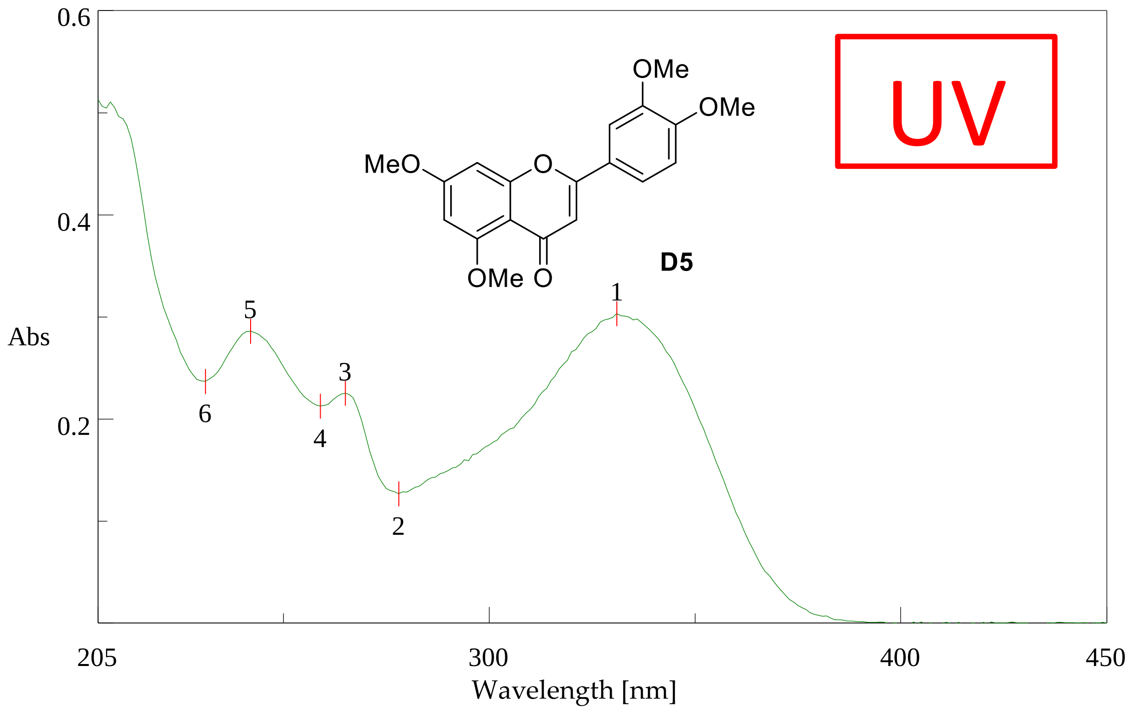

[Result of Peak Picking]

No.	Position	Intensity	No.	Position	Intensity	No.	Position	Intensity
1	331	0.303043	2	278	0.12678	3	265	0.224947
4	259	0.212534	5	242	0.286019	6	231	0.236756

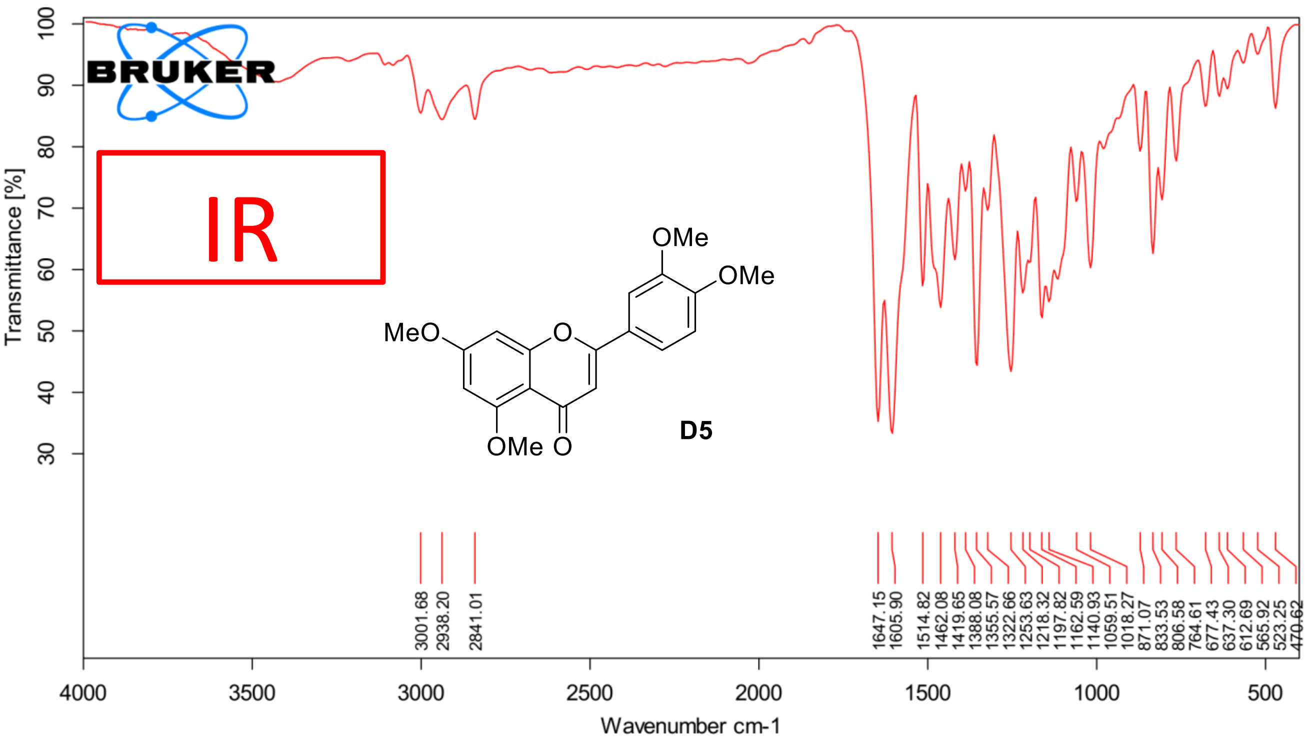

MS

Qualitative Analysis Report

**Data Filename**	3 DTM.d	**Sample Name**	3 DTM
**Sample Type**	Sample	**Position**	P2-C4
**Instrument Name**	Instrument 1	**User Name**	
**Acq Method**	Cot ngan - MSMS_Pos.m	**Acquired Time**	04/08/2020 8:18:51 PM
**IRM Calibration Status**	Success	**DA Method**	COTNGAN.M.m
**Comment**			

**Sample Group**		**Info.**	
**Stream Name**	LC 1	**Acquisition SW Version**	6200 series TOF/6500 series Q-TOF B.06.01 (B6172 SP1)

User Chromatograms

Fragmentor Voltage 140 **Collision Energy** 0 **Ionization Mode** ESI

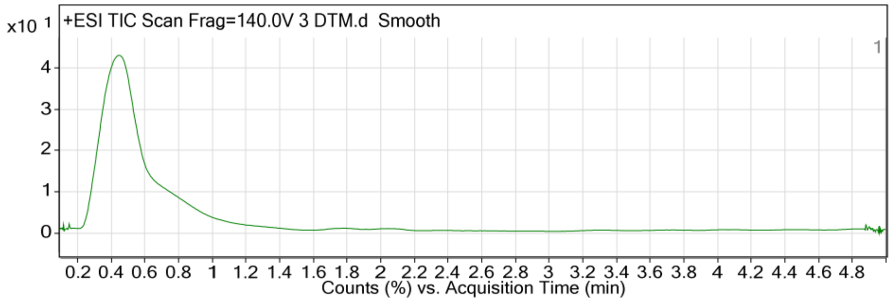

User Spectra

Fragmentor Voltage	Collision Energy	Ionization Mode
140	0	ESI

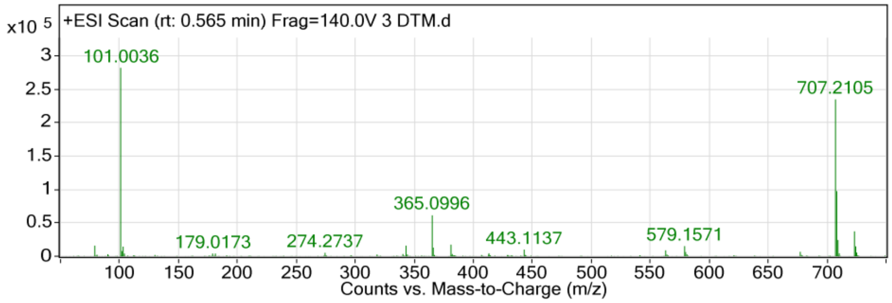

Peak List

m/z	z	Abund
101.0036	1	281893.06
365.0996	1	61498.7
381.0737	1	17344.12
707.2105	1	234806.06
708.2135	1	97507.42
709.2158	1	24530
723.1833	1	37419.95
755.2456	1	17528.59
905.2926	1	62656.96
906.296	1	37812.45

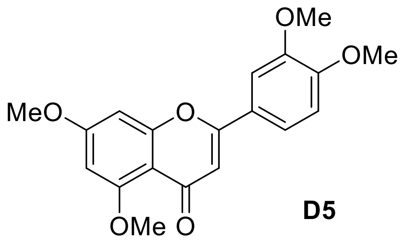

D5

--- End Of Report ---

MS

**Sample Name**	3 DTM	**Position**	P2-C4	**Instrument Name**	Instrument 1	**User Name**	
**Inj Vol**	2	**InjPosition**		**SampleType**	Sample	**IRM Calibration Status**	Success
**Data Filename**	3 DTM.d	**ACQ Method**	Cot ngan - MSMS_Pos.	**Comment**		**Acquired Time**	04/08/2020 8:18:51 PM

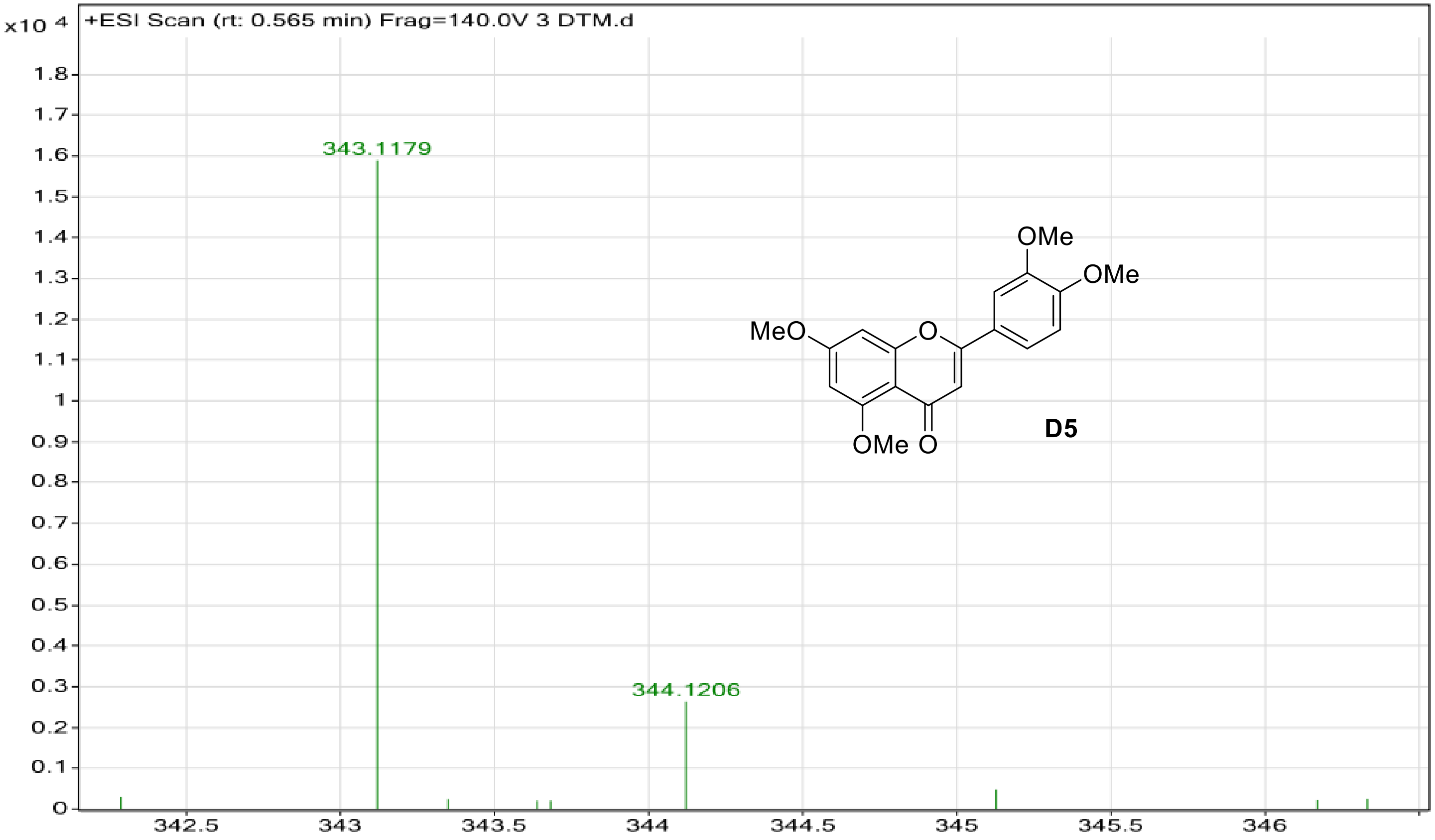

^{1}H-NMR

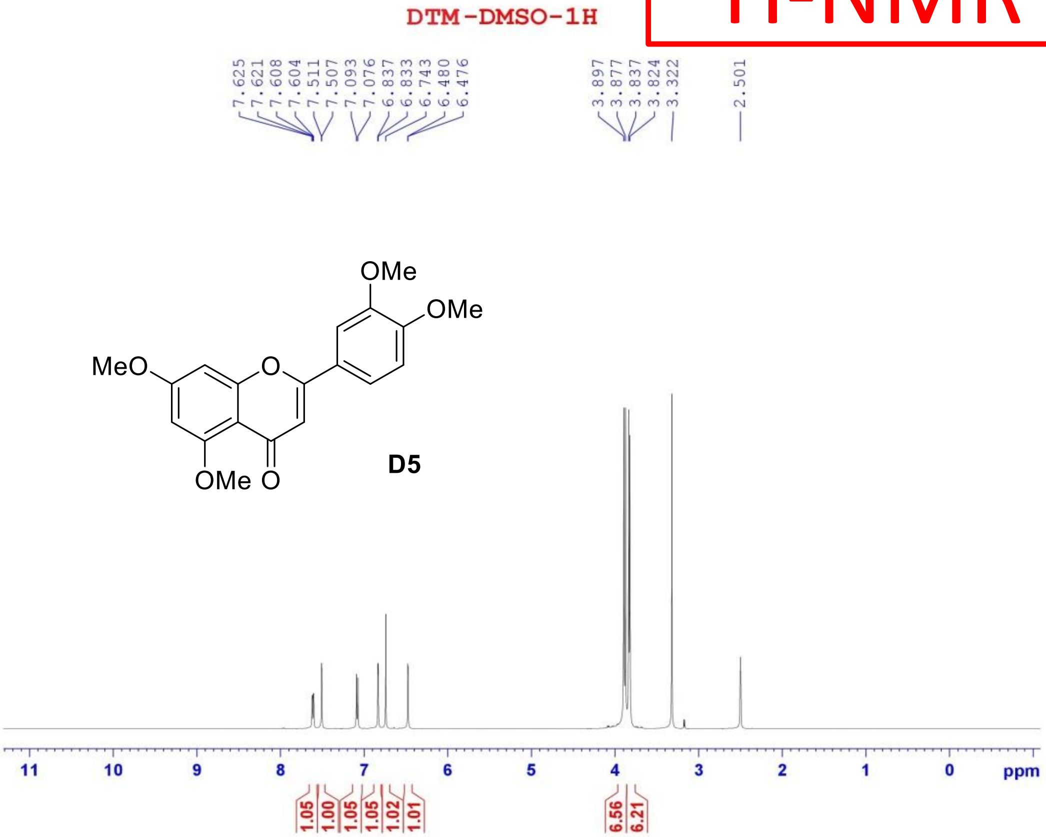

BRUKER

```
Current Data Parameters
NAME        110HUAN_HD4
EXPNO                 1
PROCNO                1

F2 - Acquisition Parameters
Date_          20190129
Time              11.47
INSTRUM           spect
PROBHD   5 mm PABBO BB/
PULPROG            zg30
TD                65536
SOLVENT            DMSO
NS                   16
DS                    2
SWH          10000.000 Hz
FIDRES        0.152588 Hz
AQ           3.2767999 sec
RG                89.63
DW               50.000 usec
DE                 6.50 usec
TE                301.6 K
D1           1.00000000 sec
TD0                   1

======== CHANNEL f1 ========
SFO1        500.2030889 MHz
NUC1                 1H
P1                10.00 usec
PLW1        22.00000000 W

F2 - Processing parameters
SI                65536
SF          500.2000036 MHz
WDW                  EM
SSB      0
LB                 0.30 Hz
GB       0
PC                 1.00
```

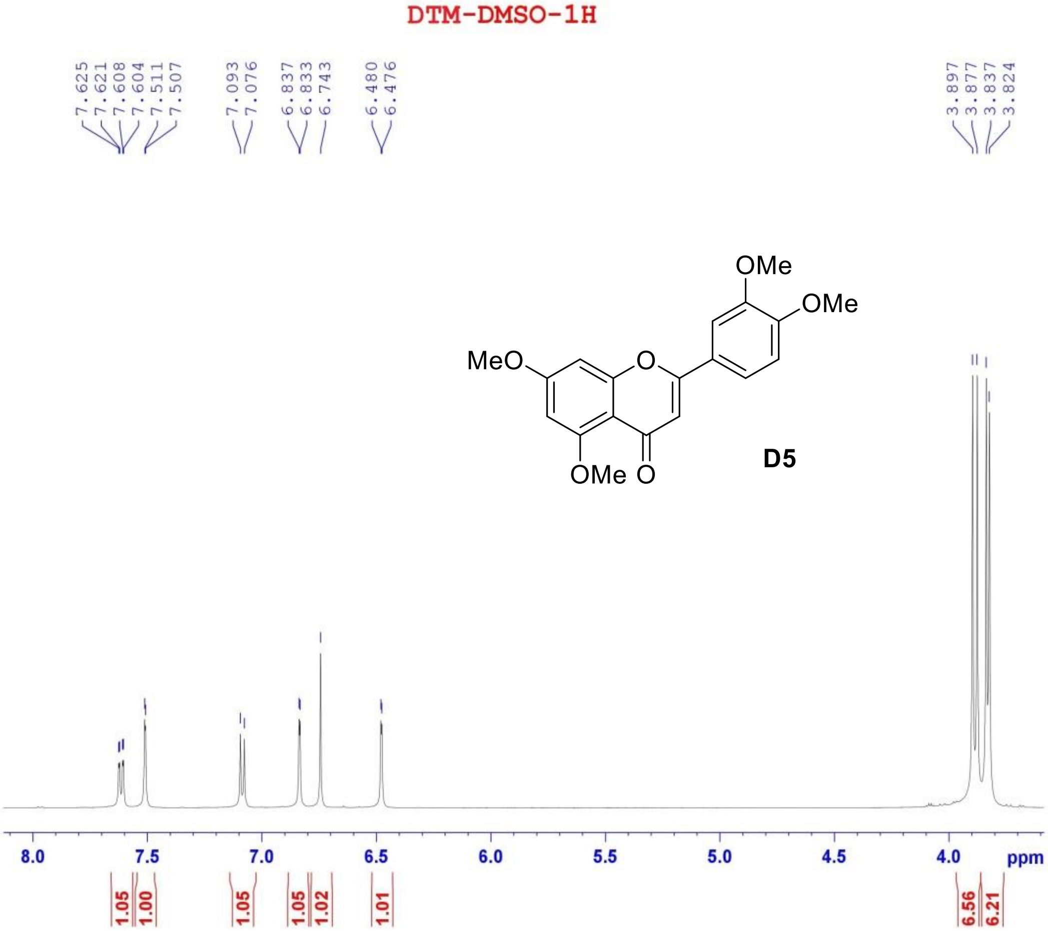

BRUKER

```
Current Data Parameters
NAME        110HUAN_HD4
EXPNO                 1
PROCNO                1

F2 - Acquisition Parameters
Date_          20190129
Time              11.47
INSTRUM           spect
PROBHD   5 mm PABBO BB/
PULPROG            zg30
TD                65536
SOLVENT            DMSO
NS                   16
DS                    2
SWH          10000.000 Hz
FIDRES        0.152588 Hz
AQ           3.2767999 sec
RG                89.63
DW               50.000 usec
DE                 6.50 usec
TE                301.6 K
D1           1.00000000 sec
TD0                   1

======== CHANNEL f1 ========
SFO1        500.2030889 MHz
NUC1                 1H
P1                10.00 usec
PLW1        22.00000000 W

F2 - Processing parameters
SI                65536
SF          500.2000036 MHz
WDW                  EM
SSB      0
LB                 0.30 Hz
GB       0
PC                 1.00
```

^{1}H-NMR

DTM-DMSO-1H

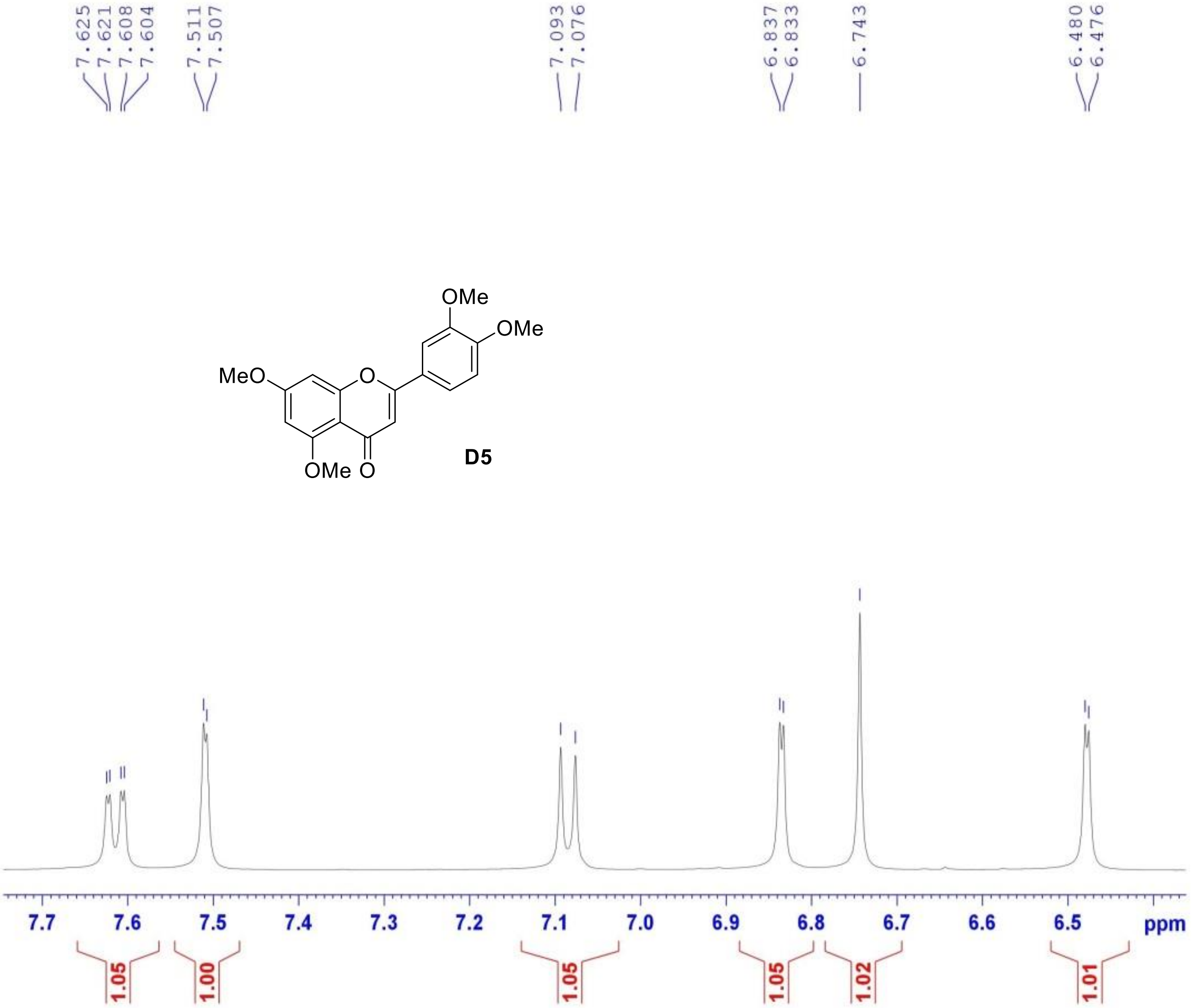

Current Data Parameters
NAME 110HUAN_HD4
EXPNO 1
PROCNO 1

F2 - Acquisition Parameters
Date_ 20190129
Time 11.47
INSTRUM spect
PROBHD 5 mm PABBO BB/
PULPROG zg30
TD 65536
SOLVENT DMSO
NS 16
DS 2
SWH 10000.000 Hz
FIDRES 0.152588 Hz
AQ 3.2767999 sec
RG 89.63
DW 50.000 usec
DE 6.50 usec
TE 301.6 K
D1 1.00000000 sec
TD0 1

======== CHANNEL f1 ========
SFO1 500.2030889 MHz
NUC1 1H
P1 10.00 usec
PLW1 22.00000000 W

F2 - Processing parameters
SI 65536
SF 500.2000036 MHz
WDW EM
SSB 0
LB 0.30 Hz
GB 0
PC 1.00

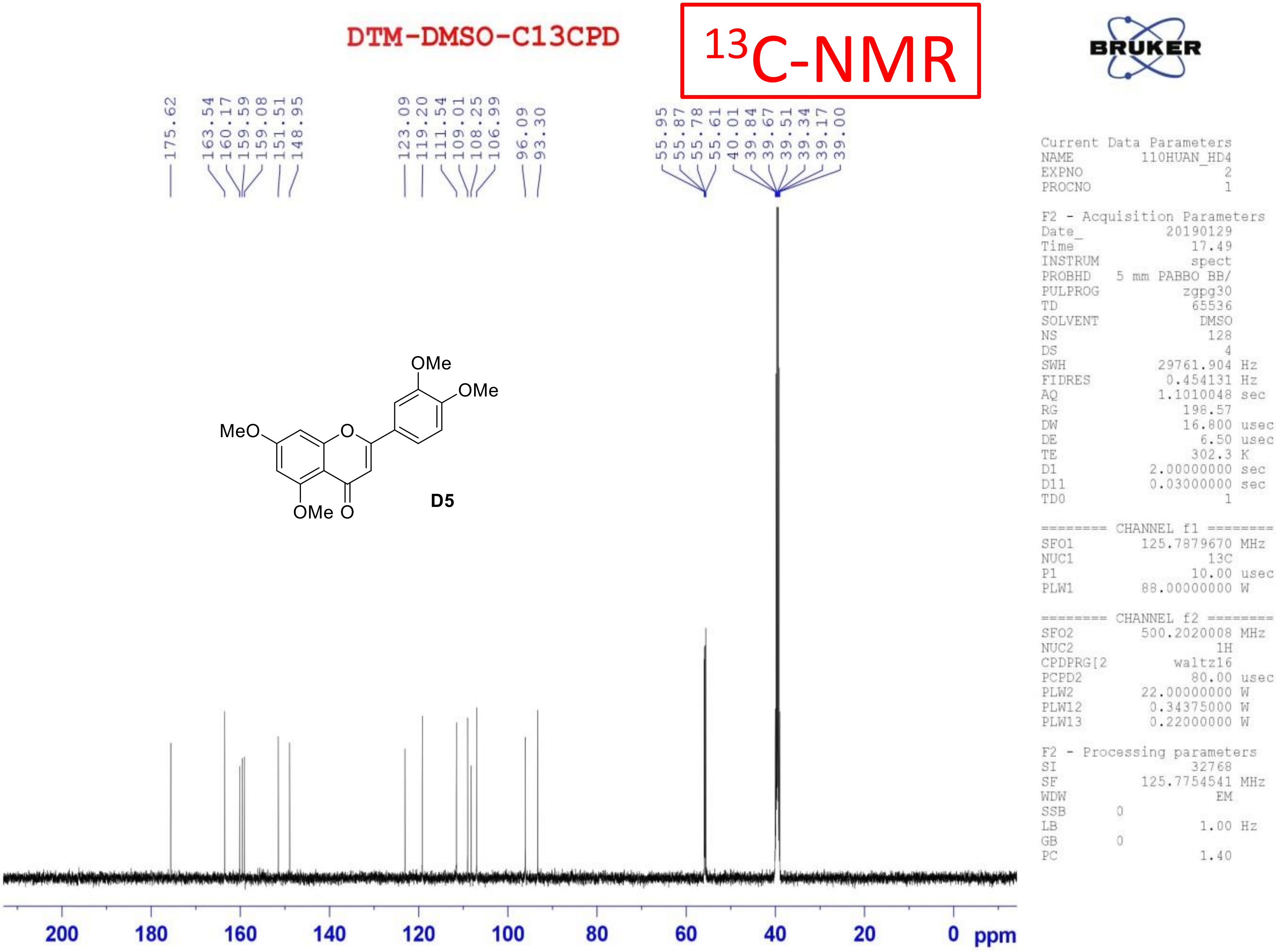
DTM-DMSO-C13CPD
13C-NMR
BRUKER
175.62
163.54
160.17
159.59
159.08
151.51
148.95
123.09
119.20
111.54
109.01
108.25
106.99
96.09
93.30
55.95
55.87
55.78
55.61
40.01
39.84
39.67
39.51
39.34
39.17
39.00
OMe
OMe
MeO
O
OMe O
D5
Current Data Parameters
NAME 110HUAN_HD4
EXPNO 2
PROCNO 1
F2 - Acquisition Parameters
Date_ 20190129
Time 17.49
INSTRUM spect
PROBHD 5 mm PABBO BB/
PULPROG zgpg30
TD 65536
SOLVENT DMSO
NS 128
DS 4
SWH 29761.904 Hz
FIDRES 0.454131 Hz
AQ 1.1010048 sec
RG 198.57
DW 16.800 usec
DE 6.50 usec
TE 302.3 K
D1 2.00000000 sec
D11 0.03000000 sec
TD0 1
======== CHANNEL f1 ========
SFO1 125.7879670 MHz
NUC1 13C
P1 10.00 usec
PLW1 88.00000000 W
======== CHANNEL f2 ========
SFO2 500.2020008 MHz
NUC2 1H
CPDPRG[2 waltz16
PCPD2 80.00 usec
PLW2 22.00000000 W
PLW12 0.34375000 W
PLW13 0.22000000 W
F2 - Processing parameters
SI 32768
SF 125.7754541 MHz
WDW EM
SSB 0
LB 1.00 Hz
GB 0
PC 1.40
200
180
160
140
120
100
80
60
40
20
0
ppm

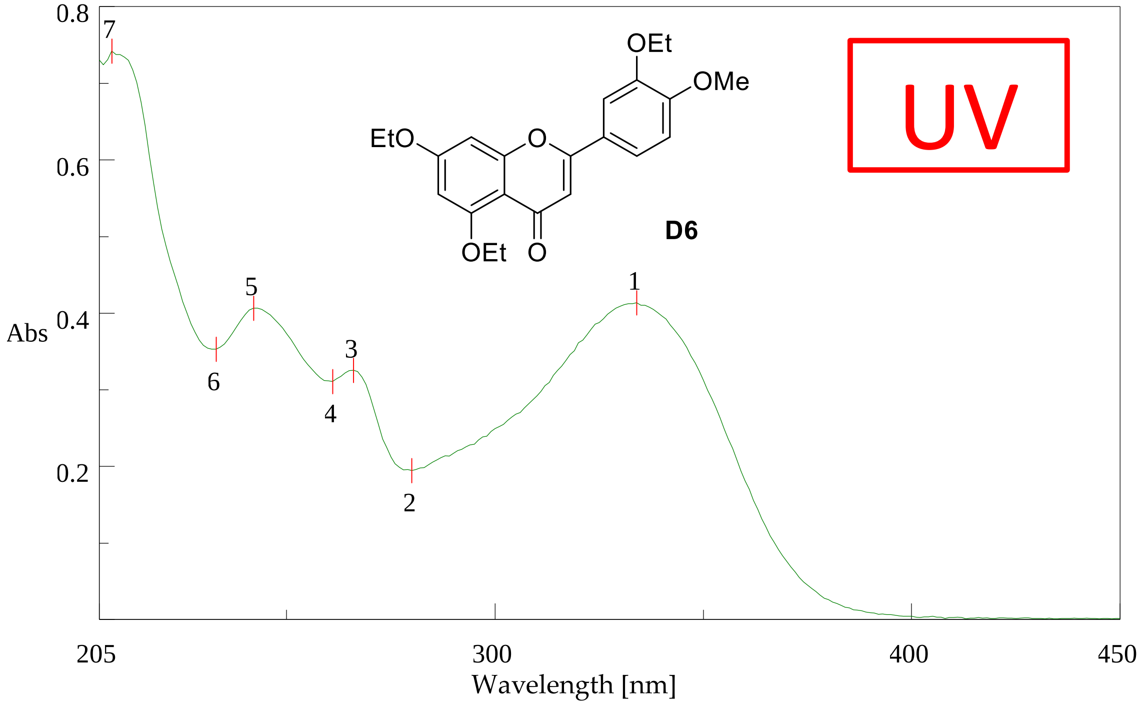

[Result of Peak Picking]

No.	Position	Intensity	No.	Position	Intensity	No.	Position	Intensity
1	334	0.413532	2	280	0.19431	3	266	0.325215
4	261	0.310755	5	242	0.406389	6	233	0.352706
7	208	0.741798						

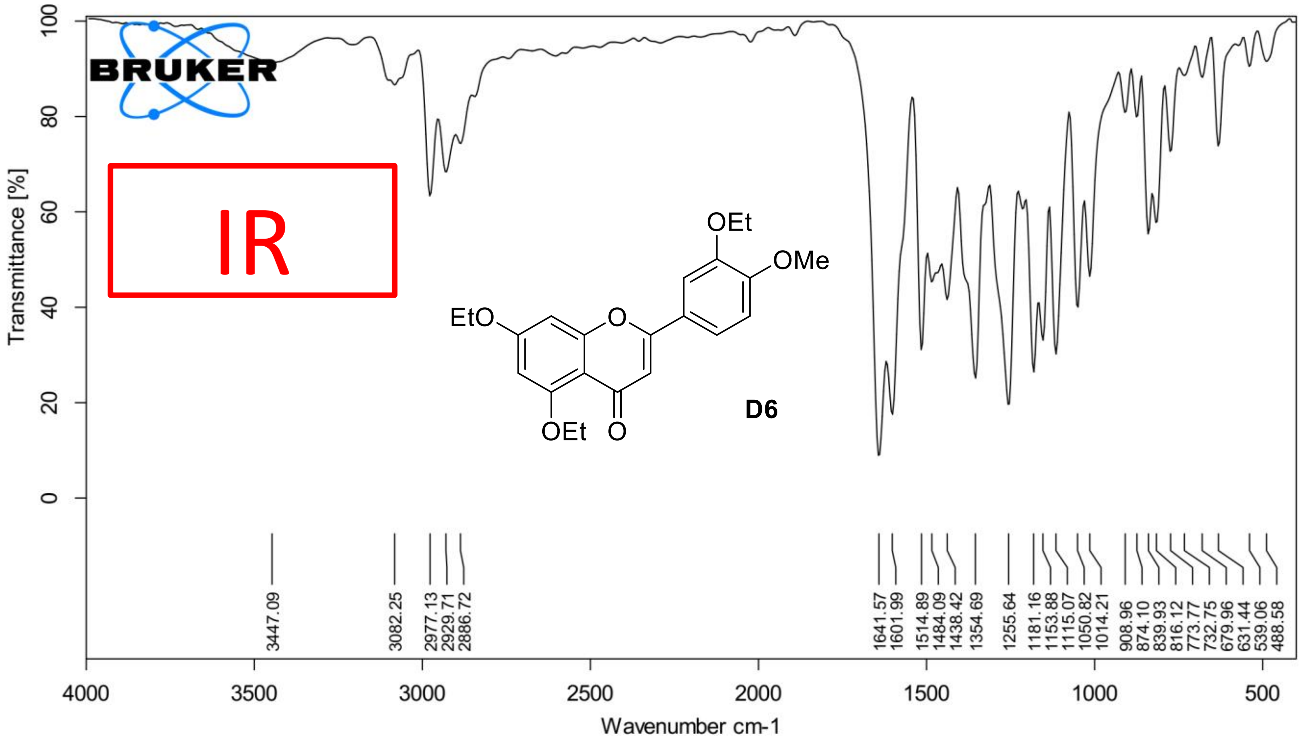

MS

Qualitative Analysis Report

**Data Filename**	5-DTE.d	**Sample Name**	5-DTE
**Sample Type**	Sample	**Position**	P2-B4
**Instrument Name**	Instrument 1	**User Name**	
**Acq Method**	Cot ngan - MSMS_Pos.m	**Acquired Time**	04/08/2020 7:28:45 PM
**IRM Calibration Status**	Success	**DA Method**	COTNGAN.M.m
**Comment**			

**Sample Group**		**Info.**	
**Stream Name**	LC 1	**Acquisition SW Version**	6200 series TOF/6500 series Q-TOF B.06.01 (B6172 SP1)

User Chromatograms

Fragmentor Voltage 140 **Collision Energy** 0 **Ionization Mode** ESI

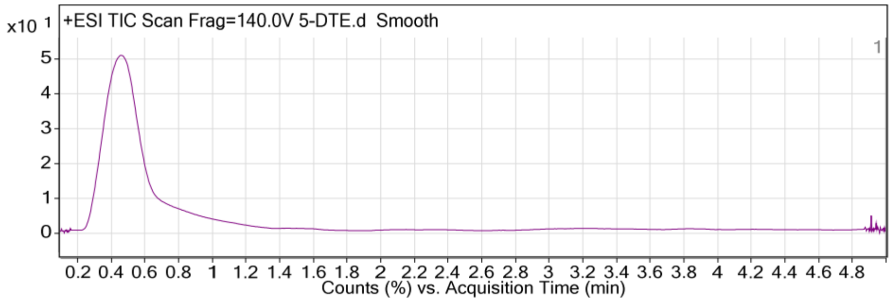

User Spectra

Fragmentor Voltage	Collision Energy	Ionization Mode
140	0	ESI

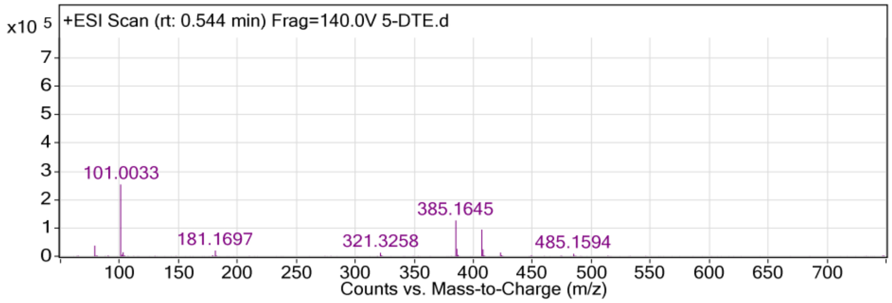

Peak List

m/z	z	Abund
79.0211	1	37639.98
101.0033	1	252987.19
385.1645	1	126322.29
407.1463	1	94535.22
791.3043	1	664562.75
791.4165		43104.09
792.3076	1	319582.63
793.3099	1	90412.51
807.2774	1	135345.67
808.2803	1	61167.45

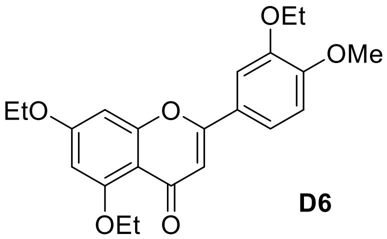

D6

--- End Of Report ---

MS

**Sample Name**	5-DTE	**Position**	P2-B4	**Instrument Name**	Instrument 1	**User Name**	
**Inj Vol**	2	**InjPosition**		**SampleType**	Sample	**IRM Calibration Status**	Success
**Data Filename**	5-DTE.d	**ACQ Method**	Cot ngan - MSMS_Pos.	**Comment**		**Acquired Time**	04/08/2020 7:28:45 PM

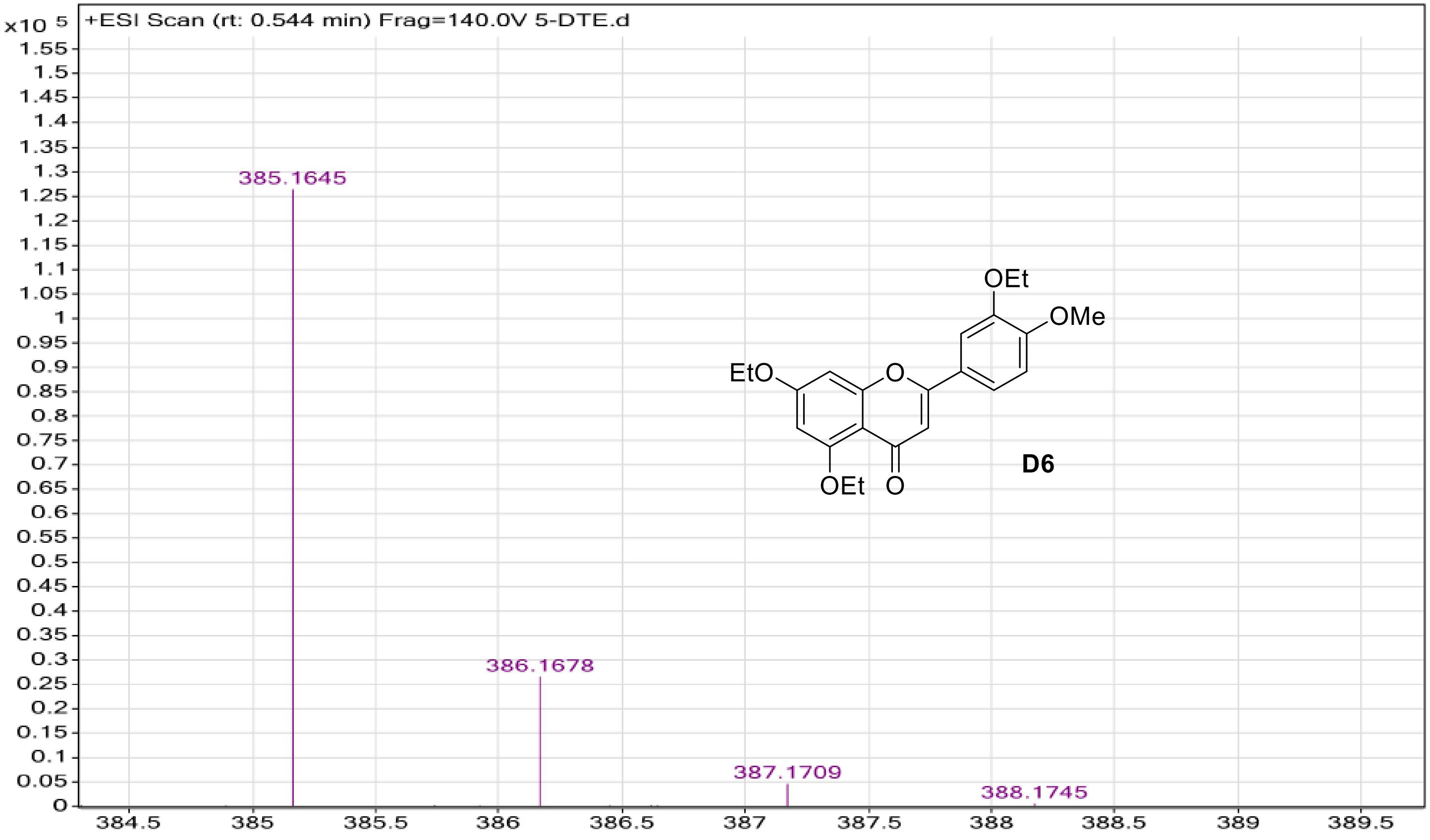

^{1}H-NMR

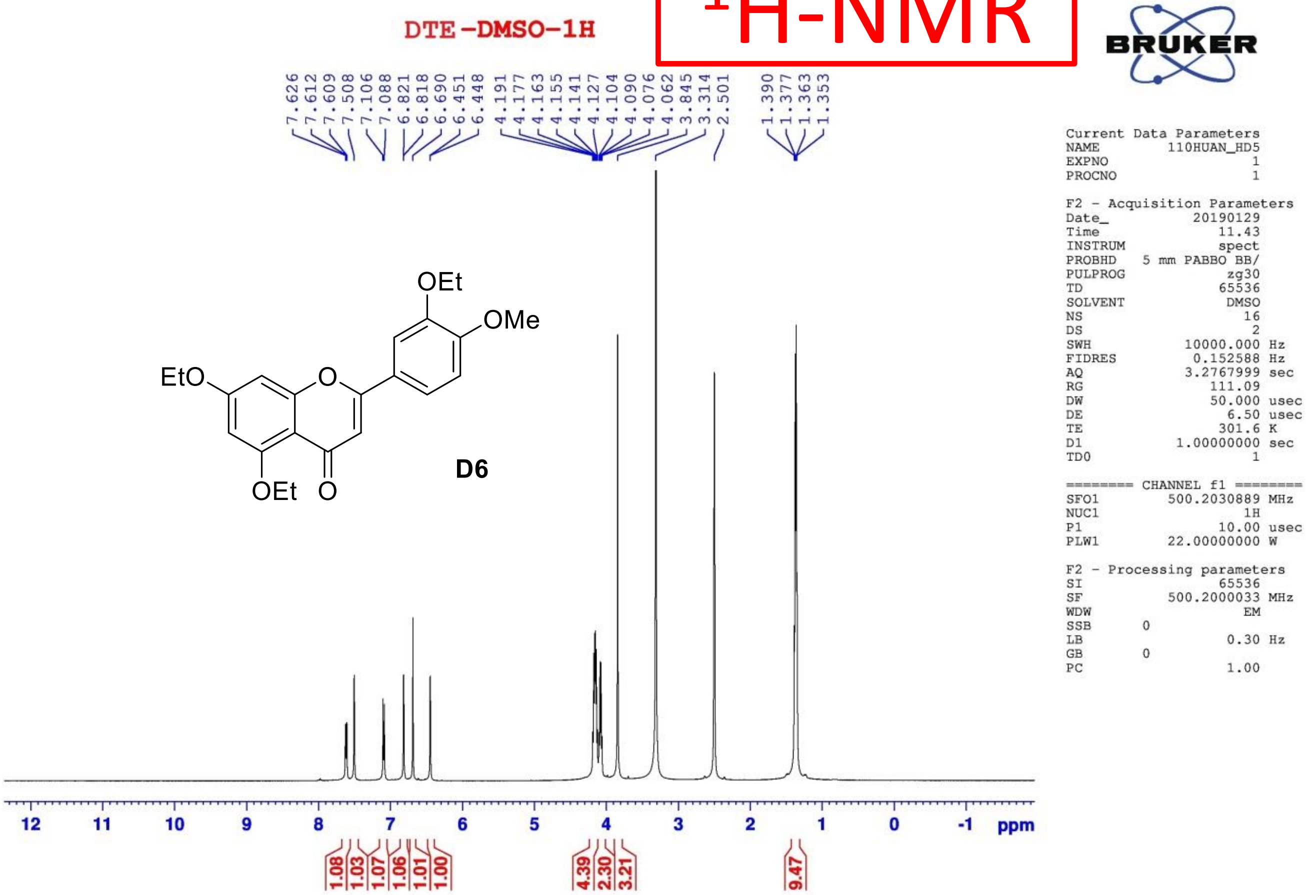
DTE-DMSO-1H
7.626
7.612
7.609
7.508
7.106
7.088
6.821
6.818
6.690
6.451
6.448
4.191
4.177
4.163
4.155
4.141
4.127
4.104
4.090
4.076
4.062
3.845
3.314
2.501
1.390
1.377
1.363
1.353
OEt
OMe
EtO
O
OEt
O
D6
1.08
1.03
1.07
1.06
1.01
1.00
4.39
2.30
3.21
9.47
ppm
BRUKER
Current Data Parameters
NAME 110HUAN_HD5
EXPNO 1
PROCNO 1
F2 - Acquisition Parameters
Date_ 20190129
Time 11.43
INSTRUM spect
PROBHD 5 mm PABBO BB/
PULPROG zg30
TD 65536
SOLVENT DMSO
NS 16
DS 2
SWH 10000.000 Hz
FIDRES 0.152588 Hz
AQ 3.2767999 sec
RG 111.09
DW 50.000 usec
DE 6.50 usec
TE 301.6 K
D1 1.00000000 sec
TD0 1
======== CHANNEL f1 ========
SFO1 500.2030889 MHz
NUC1 1H
P1 10.00 usec
PLW1 22.00000000 W
F2 - Processing parameters
SI 65536
SF 500.2000033 MHz
WDW EM
SSB 0
LB 0.30 Hz
GB 0
PC 1.00

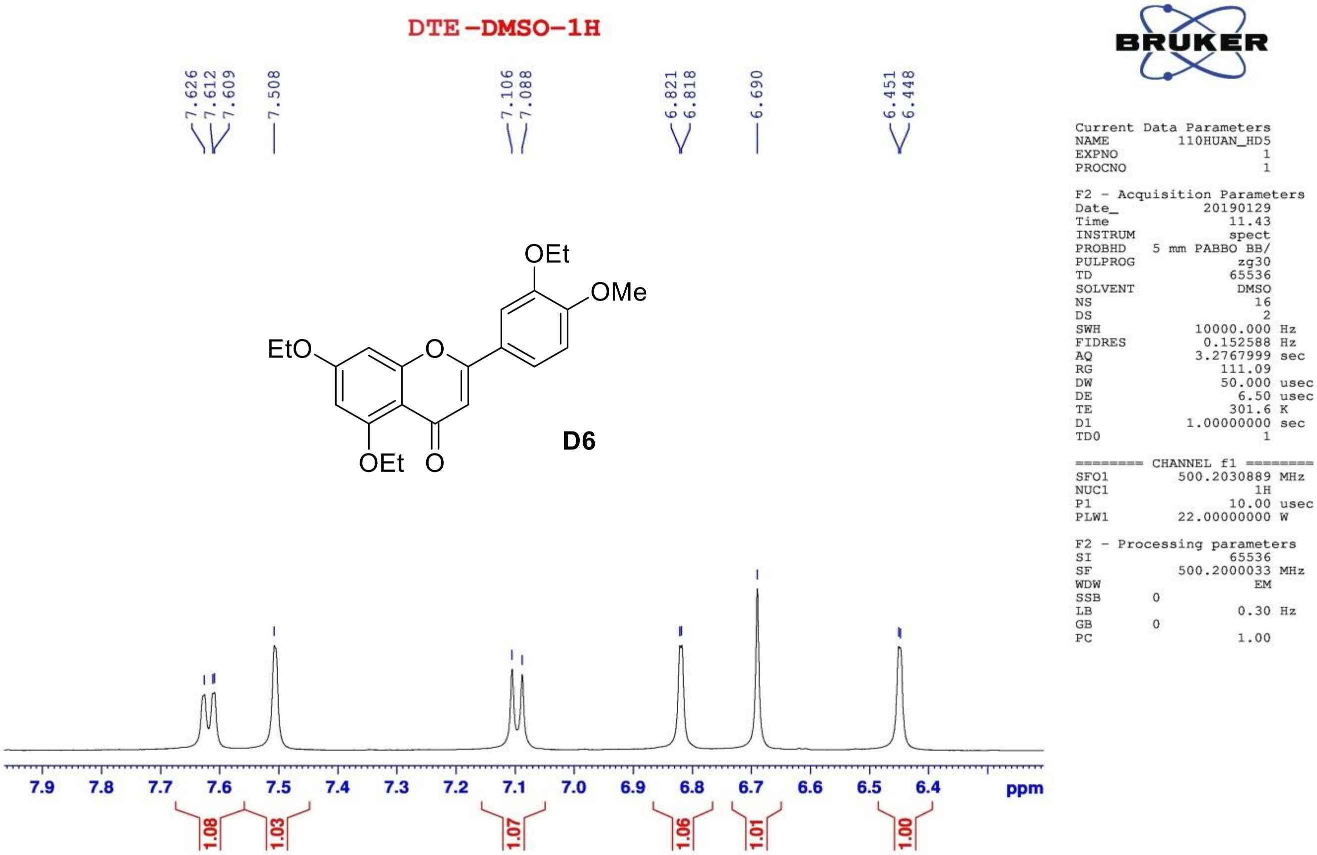
DTE-DMSO-1H
7.626
7.612
7.609
7.508
7.106
7.088
6.821
6.818
6.690
6.451
6.448
OEt
OMe
EtO
O
OEt
O
D6
1.08
1.03
1.07
1.06
1.01
1.00
ppm
BRUKER
Current Data Parameters
NAME 110HUAN_HD5
EXPNO 1
PROCNO 1
F2 - Acquisition Parameters
Date_ 20190129
Time 11.43
INSTRUM spect
PROBHD 5 mm PABBO BB/
PULPROG zg30
TD 65536
SOLVENT DMSO
NS 16
DS 2
SWH 10000.000 Hz
FIDRES 0.152588 Hz
AQ 3.2767999 sec
RG 111.09
DW 50.000 usec
DE 6.50 usec
TE 301.6 K
D1 1.00000000 sec
TD0 1
======== CHANNEL f1 ========
SFO1 500.2030889 MHz
NUC1 1H
P1 10.00 usec
PLW1 22.00000000 W
F2 - Processing parameters
SI 65536
SF 500.2000033 MHz
WDW EM
SSB 0
LB 0.30 Hz
GB 0
PC 1.00

^{1}H-NMR

DTE-DMSO-1H

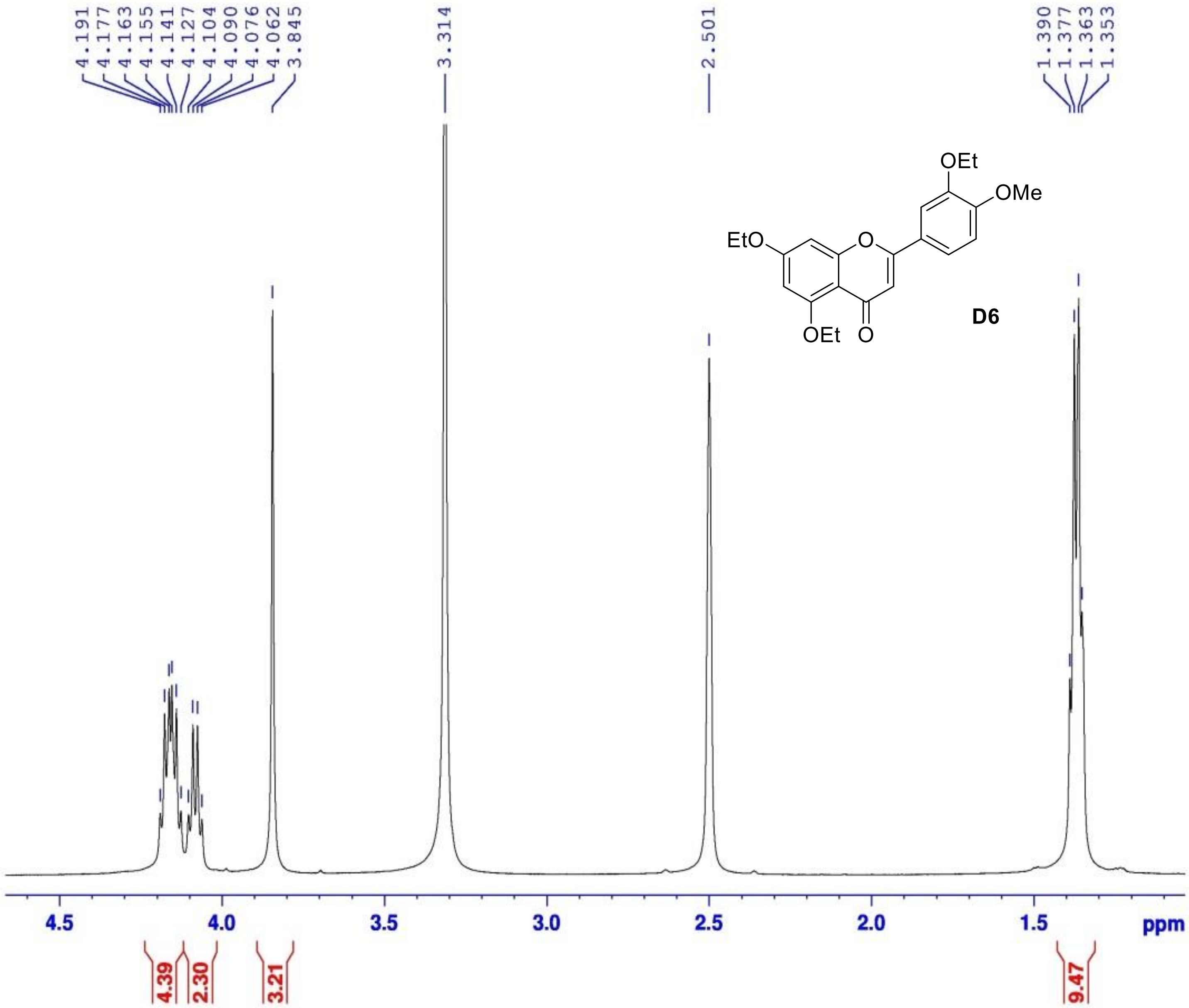

BRUKER

```
Current Data Parameters
NAME          110HUAN_HD5
EXPNO                   1
PROCNO                  1

F2 - Acquisition Parameters
Date_            20190129
Time                11.43
INSTRUM             spect
PROBHD    5 mm PABBO BB/
PULPROG              zg30
TD                  65536
SOLVENT              DMSO
NS                     16
DS                      2
SWH            10000.000 Hz
FIDRES          0.152588 Hz
AQ             3.2767999 sec
RG                 111.09
DW                 50.000 usec
DE                   6.50 usec
TE                  301.6 K
D1            1.00000000 sec
TD0                     1

======== CHANNEL f1 ========
SFO1         500.2030889 MHz
NUC1                   1H
P1                  10.00 usec
PLW1          22.00000000 W

F2 - Processing parameters
SI                  65536
SF            500.2000033 MHz
WDW                    EM
SSB        0
LB                   0.30 Hz
GB         0
PC                   1.00
```

DTE-DMSO-C13CPD

^{13}C-NMR

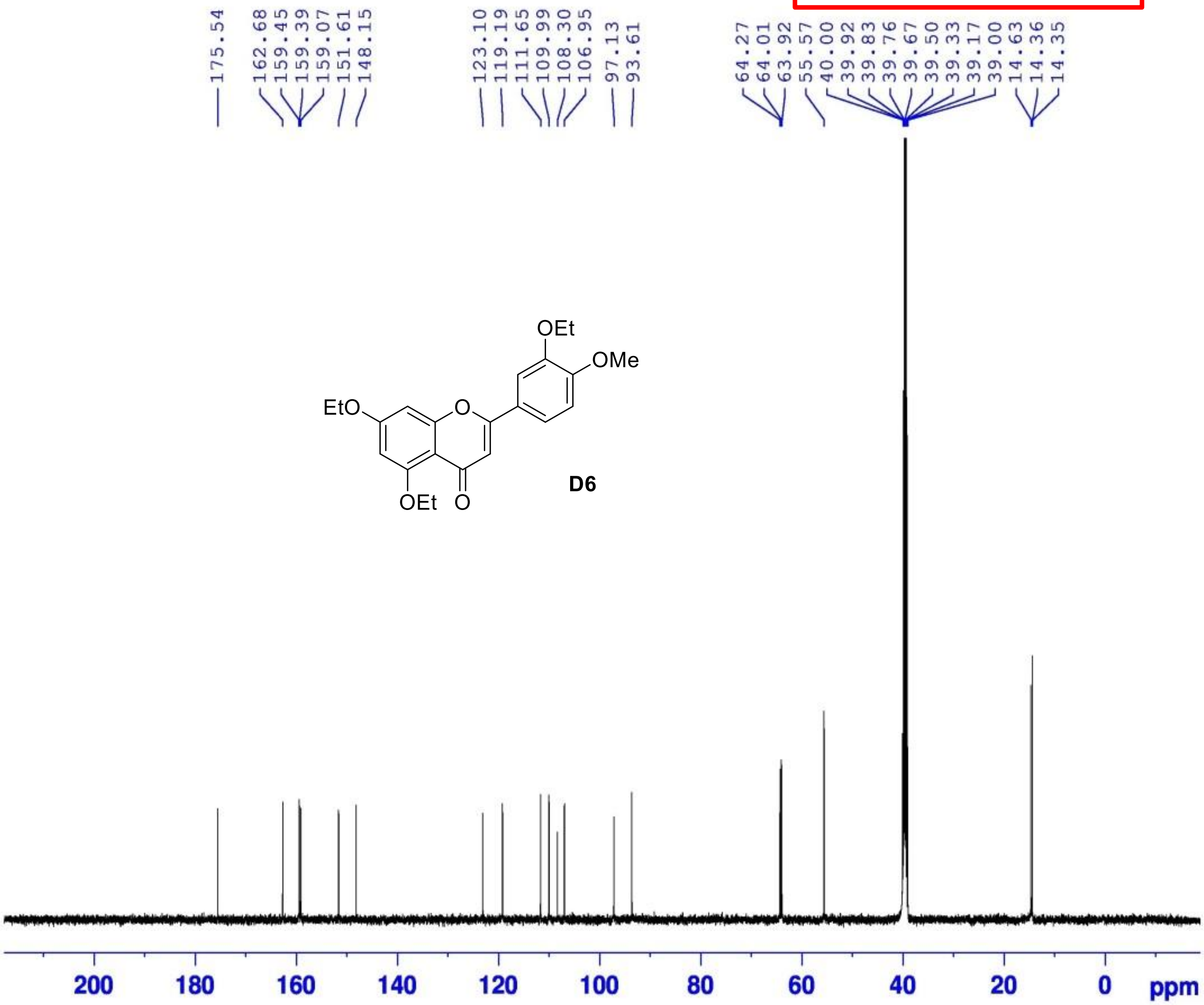

D6

Current Data Parameters	
NAME	110HUAN_HD5
EXPNO	2
PROCNO	1

F2 - Acquisition Parameters	
Date_	20190129
Time	17.07
INSTRUM	spect
PROBHD	5 mm PABBO BB/
PULPROG	zgpg30
TD	65536
SOLVENT	DMSO
NS	256
DS	4
SWH	29761.904 Hz
FIDRES	0.454131 Hz
AQ	1.1010048 sec
RG	198.57
DW	16.800 usec
DE	6.50 usec
TE	302.5 K
D1	2.00000000 sec
D11	0.03000000 sec
TD0	1

======== CHANNEL f1 ========	
SFO1	125.7879670 MHz
NUC1	13C
P1	10.00 usec
PLW1	88.00000000 W

======== CHANNEL f2 ========	
SFO2	500.2020008 MHz
NUC2	1H
CPDPRG[2	waltz16
PCPD2	80.00 usec
PLW2	22.00000000 W
PLW12	0.34375000 W
PLW13	0.22000000 W

F2 - Processing parameters	
SI	32768
SF	125.7754556 MHz
WDW	EM
SSB	0
LB	1.00 Hz
GB	0
PC	1.40

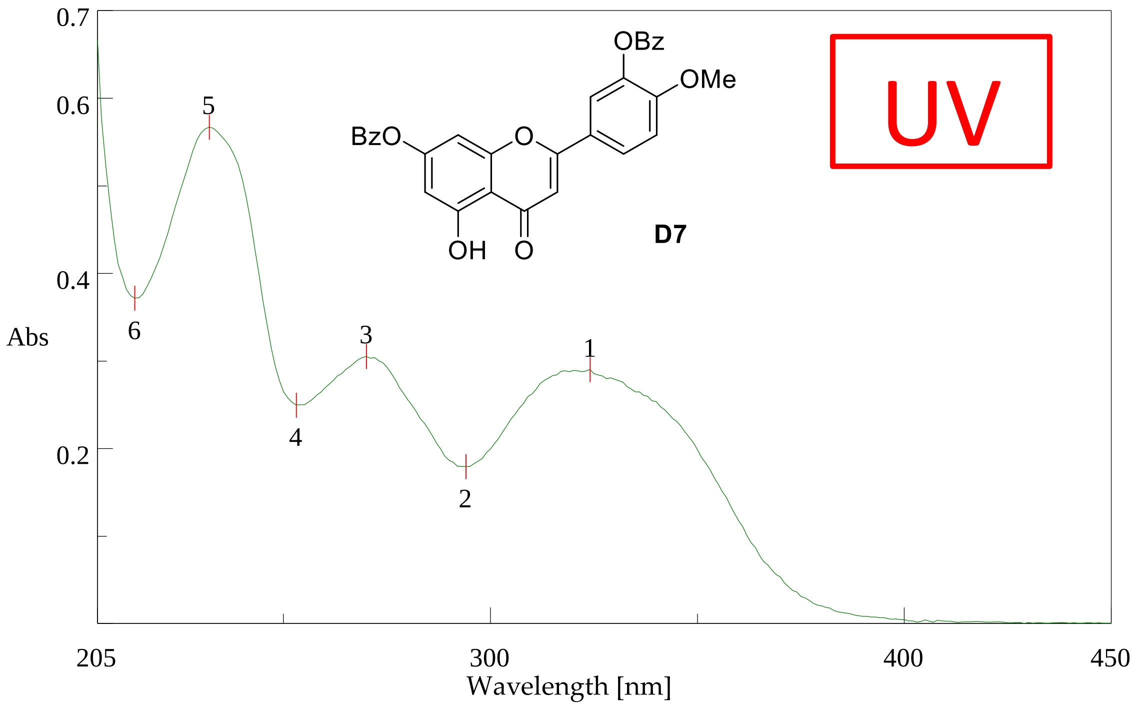

[Result of Peak Picking]

No.	Position	Intensity	No.	Position	Intensity	No.	Position	Intensity
1	324	0.290155	2	294	0.179039	3	270	0.305058
4	253	0.249541	5	232	0.566944	6	214	0.371536

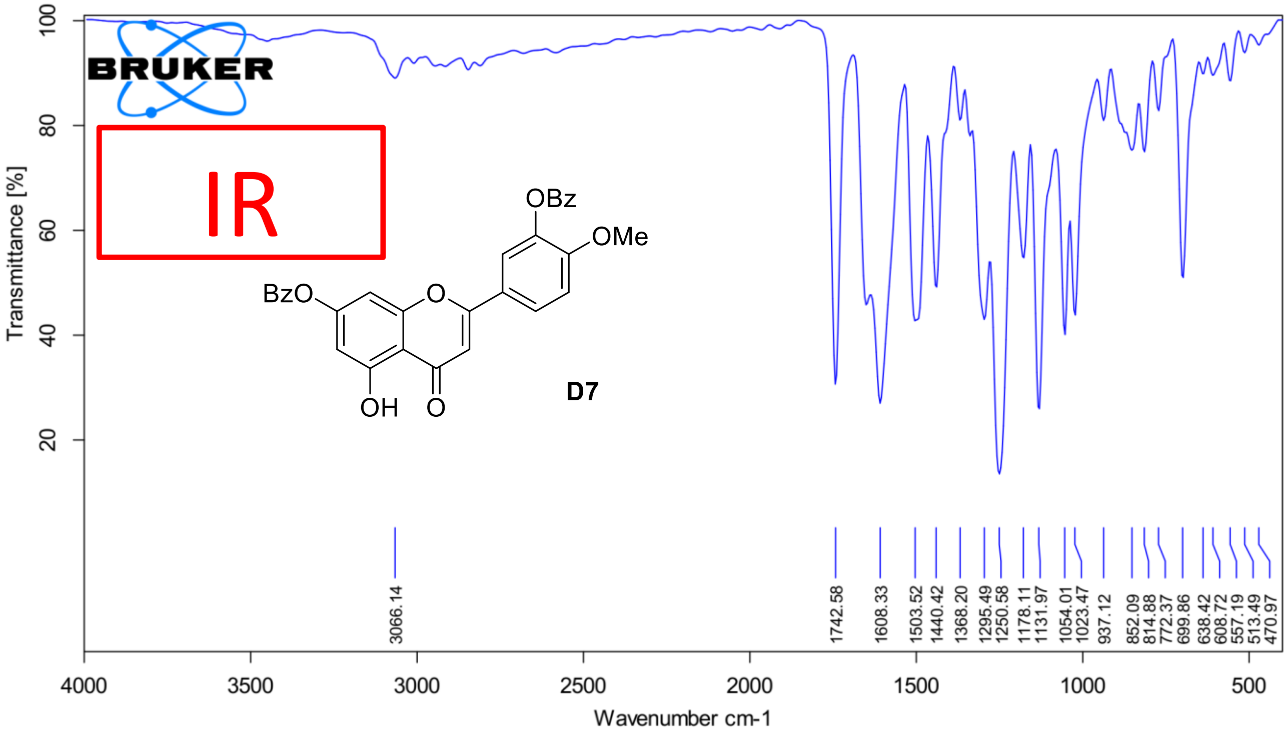

MS

Qualitative Analysis Report

**Data Filename**	8.DDB.d	**Sample Name**	8.DDB
**Sample Type**	Sample	**Position**	P2-B7
**Instrument Name**	Instrument 1	**User Name**	
**Acq Method**	Cot ngan - MSMS_Pos.m	**Acquired Time**	04/08/2020 7:45:28 PM
**IRM Calibration Status**	Success	**DA Method**	COTNGAN.M.m
**Comment**			

**Sample Group**		**Info.**	
**Stream Name**	LC 1	**Acquisition SW Version**	6200 series TOF/6500 series Q-TOF B.06.01 (B6172 SP1)

User Chromatograms

Fragmentor Voltage 140 **Collision Energy** 0 **Ionization Mode** ESI

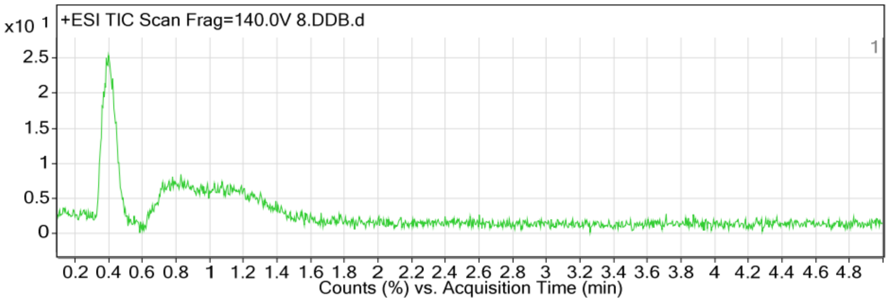

User Spectra

**Fragmentor Voltage**	**Collision Energy**	**Ionization Mode**
140	0	ESI

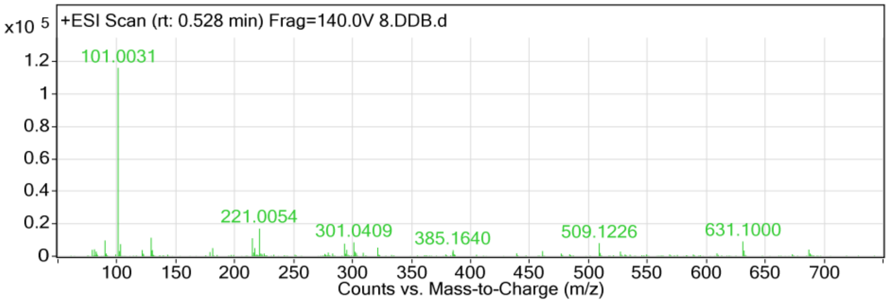

Peak List

m/z	z	Abund
90.0059	2	9788.28
101.0031	1	116298.95
102.9987	1	7500.39
129.0125	2	11382.23
214.9812	1	11176.09
221.0054	1	17083.07
292.9947	1	7780.54
301.0409	1	8574.88
509.1226	1	8061.85
631.1	1	9216.2

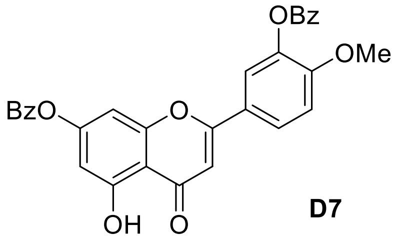

D7

--- End Of Report ---

MS

Sample Name	8.DDB	Position	P2-B7	Instrument Name	Instrument 1	User Name	
Inj Vol	2	InjPosition		SampleType	Sample	IRM Calibration Status	Success
Data Filename	8.DDB.d	ACQ Method	Cot ngan - MSMS_Pos.	Comment		Acquired Time	04/08/2020 7:45:28 PM

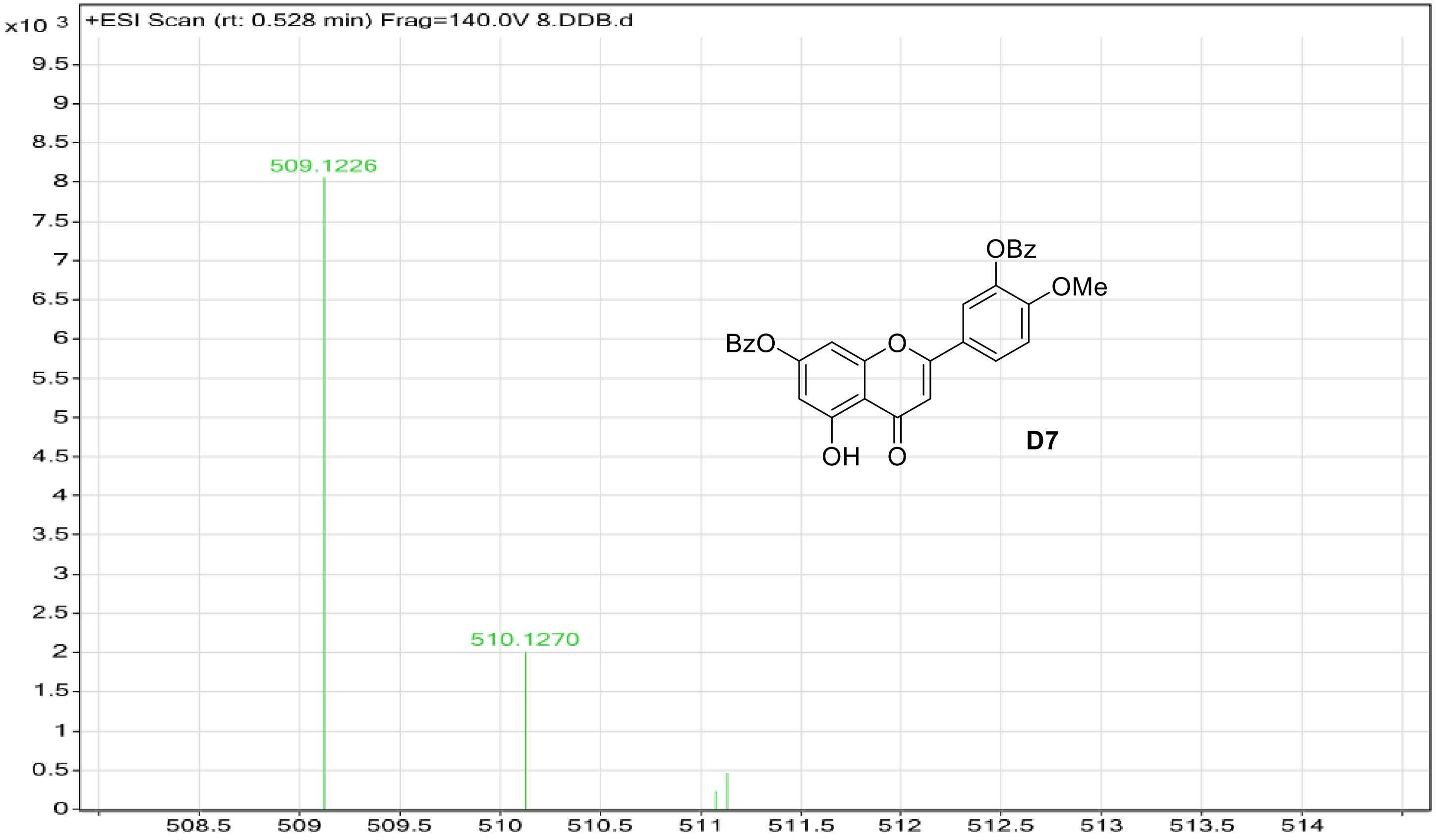

^{1}H-NMR

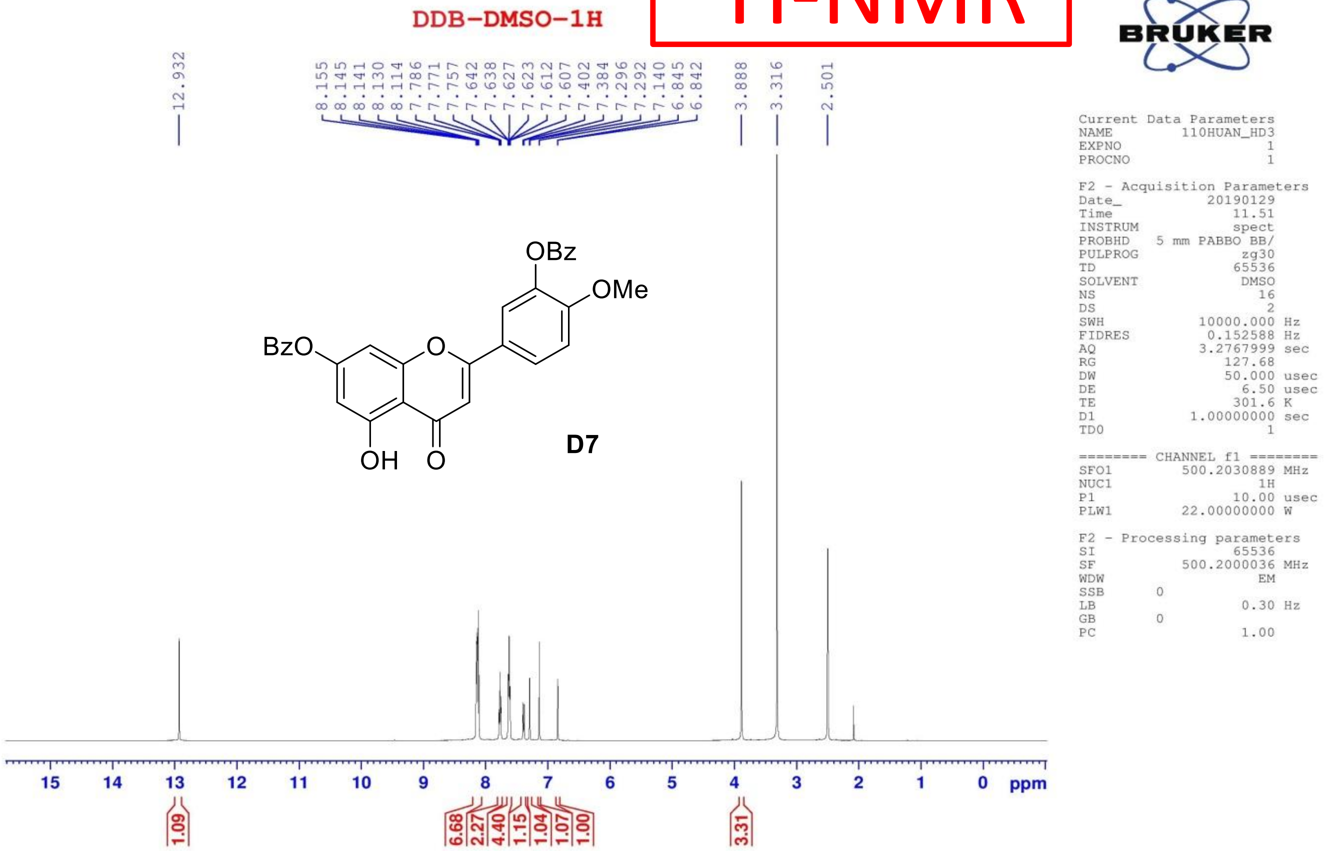
DDB-DMSO-1H
12.932
8.155 8.145 8.141 8.130 8.114 7.786 7.771 7.757 7.642 7.638 7.627 7.623 7.612 7.607 7.402 7.384 7.296 7.292 7.140 6.845 6.842
3.888
3.316
2.501
OBz
OMe
BzO
O
OH
O
D7
15 14 13 12 11 10 9 8 7 6 5 4 3 2 1 0 ppm
1.09
6.68 2.27 4.40 1.15 1.04 1.07 1.00
3.31
BRUKER
Current Data Parameters
NAME 110HUAN_HD3
EXPNO 1
PROCNO 1
F2 - Acquisition Parameters
Date_ 20190129
Time 11.51
INSTRUM spect
PROBHD 5 mm PABBO BB/
PULPROG zg30
TD 65536
SOLVENT DMSO
NS 16
DS 2
SWH 10000.000 Hz
FIDRES 0.152588 Hz
AQ 3.2767999 sec
RG 127.68
DW 50.000 usec
DE 6.50 usec
TE 301.6 K
D1 1.00000000 sec
TD0 1
======== CHANNEL f1 ========
SFO1 500.2030889 MHz
NUC1 1H
P1 10.00 usec
PLW1 22.00000000 W
F2 - Processing parameters
SI 65536
SF 500.2000036 MHz
WDW EM
SSB 0
LB 0.30 Hz
GB 0
PC 1.00

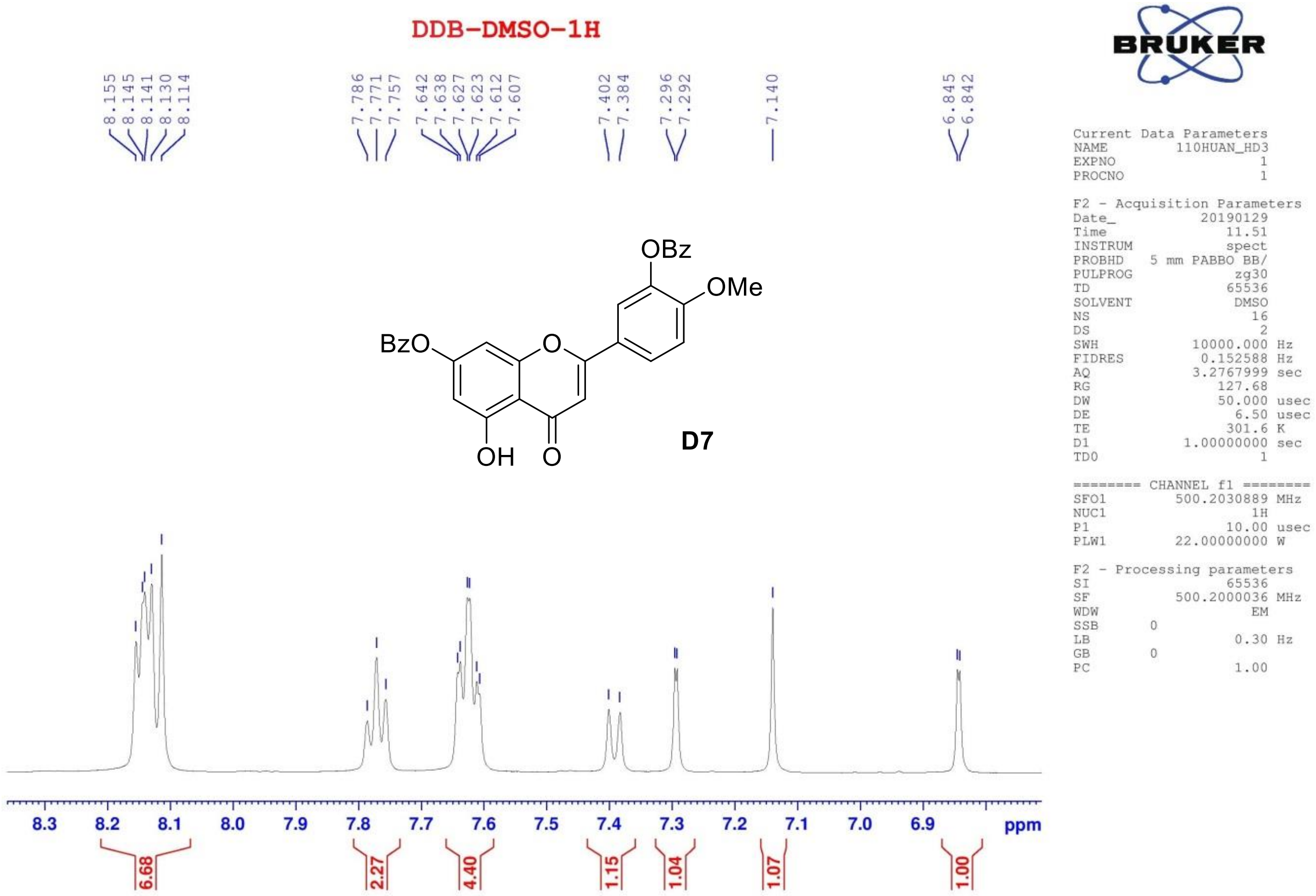
DDB-DMSO-1H
8.155 8.145 8.141 8.130 8.114
7.786 7.771 7.757
7.642 7.638 7.627 7.623 7.612 7.607
7.402 7.384
7.296 7.292
7.140
6.845 6.842
OBz
OMe
BzO
O
OH
O
D7
8.3 8.2 8.1 8.0 7.9 7.8 7.7 7.6 7.5 7.4 7.3 7.2 7.1 7.0 6.9 ppm
6.68 2.27 4.40 1.15 1.04 1.07 1.00
BRUKER
Current Data Parameters
NAME 110HUAN_HD3
EXPNO 1
PROCNO 1
F2 - Acquisition Parameters
Date_ 20190129
Time 11.51
INSTRUM spect
PROBHD 5 mm PABBO BB/
PULPROG zg30
TD 65536
SOLVENT DMSO
NS 16
DS 2
SWH 10000.000 Hz
FIDRES 0.152588 Hz
AQ 3.2767999 sec
RG 127.68
DW 50.000 usec
DE 6.50 usec
TE 301.6 K
D1 1.00000000 sec
TD0 1
======== CHANNEL f1 ========
SFO1 500.2030889 MHz
NUC1 1H
P1 10.00 usec
PLW1 22.00000000 W
F2 - Processing parameters
SI 65536
SF 500.2000036 MHz
WDW EM
SSB 0
LB 0.30 Hz
GB 0
PC 1.00

DDB-DMSO-C13CPD

^{13}C-NMR

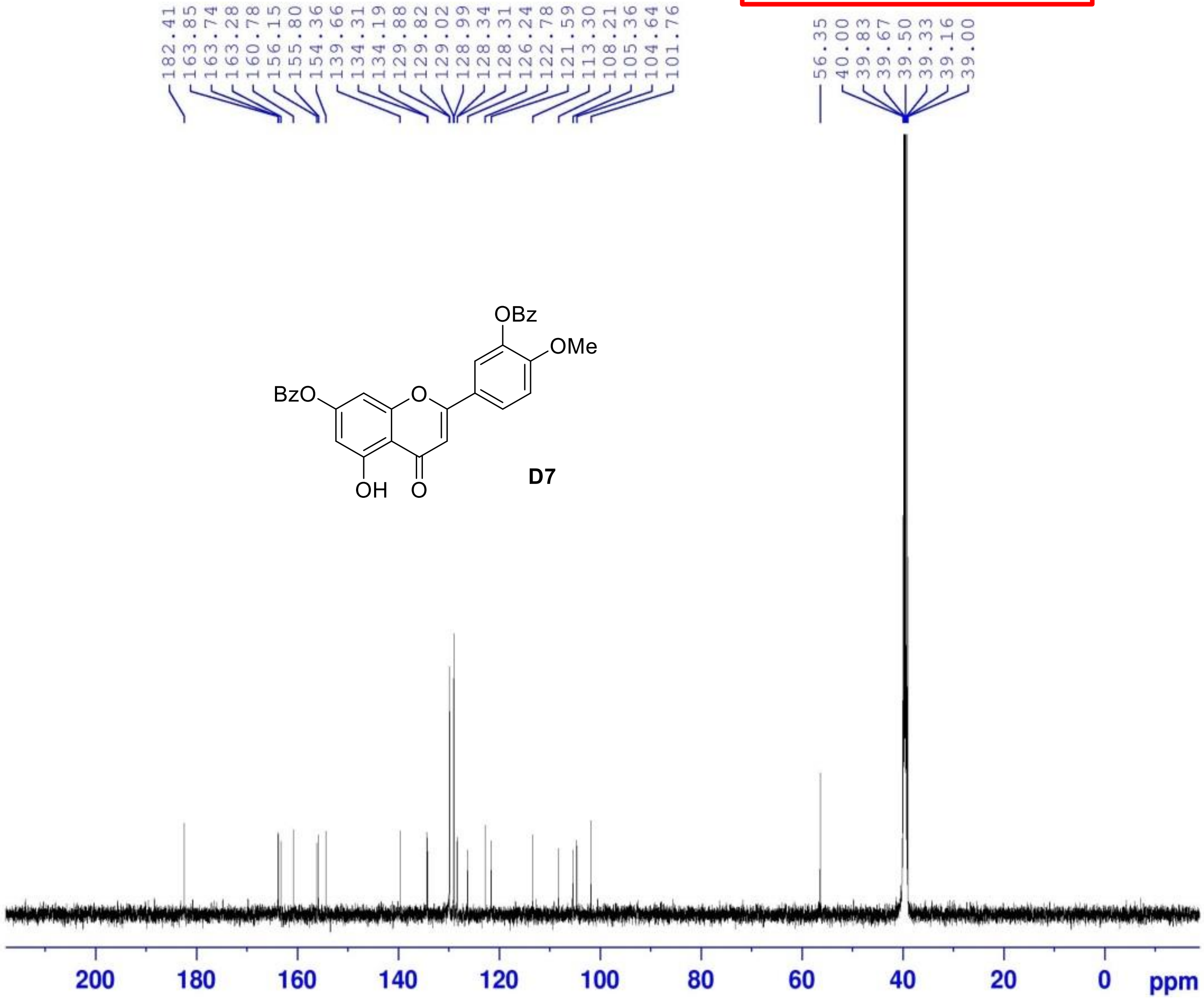

D7

BRUKER

```
Current Data Parameters
NAME          110HUAN_HD3
EXPNO                   2
PROCNO                  1

F2 - Acquisition Parameters
Date_            20190129
Time                12.35
INSTRUM             spect
PROBHD     5 mm PABBO BB/
PULPROG           zgpg30
TD                 65536
SOLVENT             DMSO
NS                   256
DS                     4
SWH            29761.904 Hz
FIDRES          0.454131 Hz
AQ             1.1010048 sec
RG                198.57
DW                16.800 usec
DE                  6.50 usec
TE                 302.3 K
D1            2.00000000 sec
D11           0.03000000 sec
TD0                    1

======== CHANNEL f1 ========
SFO1         125.7879670 MHz
NUC1                 13C
P1                 10.00 usec
PLW1         88.00000000 W

======== CHANNEL f2 ========
SFO2         500.2020008 MHz
NUC2                  1H
CPDPRG[2           waltz16
PCPD2              80.00 usec
PLW2         22.00000000 W
PLW12         0.34375000 W
PLW13         0.22000000 W

F2 - Processing parameters
SI                 32768
SF           125.7754559 MHz
WDW                   EM
SSB      0
LB                  1.00 Hz
GB       0
PC                  1.40
```

Reference

1. Tran, T.-S.; Le, M.-T.; Tran, T.-D.; Tran, T.-H.; Thai, K.-M. Design of Curcumin and Flavonoid Derivatives with Acetylcholinesterase and Beta-Secretase Inhibitory Activities Using in Silico Approaches. *Molecules* **2020**, *25*, 3644.
2. Wildman, S.A.; Crippen, G.M. Prediction of Physicochemical Parameters by Atomic Contributions. *J Chem Inf Comput Sci* **1999**, *39*, 868-873, doi:10.1021/ci990307l.
3. Oprea, T.I. Property distribution of drug-related chemical databases. *Journal of computer-aided molecular design* **2000**, *14*, 251-264, doi:10.1023/a:1008130001697.
4. Neumann, U.; Ufer, M.; Jacobson, L.H.; Rouzade-Dominguez, M.-L.; Huledal, G.; Kolly, C.; Lüönd, R.M.; Machauer, R.; Veenstra, S.J.; Hurth, K., et al. The BACE-1 inhibitor CNP520 for prevention trials in Alzheimer's disease. *EMBO Mol Med* **2018**, *10*, e9316, doi:10.15252/emmm.201809316.
